# Zoanthamine-Type Alkaloids Derived from Cultured *Zoanthus kuroshio* with Therapeutic Potential Against Osteoporosis

**DOI:** 10.1021/acs.jnatprod.5c00457

**Published:** 2025-06-10

**Authors:** Ngoc-Thac Pham, Bo-Rong Peng, Huong-Giang Le, You-Song Cheng, Yun-Shiuan Chen, Thanh Hao Huynh, Lo-Yun Chen, Le Anh Tuan Nguyen, Dang T. Nguyen, Yu-Chia Chang, Jui-Hsin Su, Mohamed El-Shazly, Mei-Hsien Lee, Kuei-Hung Lai

**Affiliations:** † PhD Program in Clinical Drug Development of Herbal Medicine, College of Pharmacy, 38032 Taipei Medical University, Taipei 110301, Taiwan; ‡ Graduate Institute of Pharmacognosy, College of Pharmacy, 38032 Taipei Medical University, Taipei 110301, Taiwan; § Graduate Institute of Healthy Industry Technology, Center for Drug Research and Development, College of Human Ecology, 63113 Chang Gung University of Science and Technology, Taoyuan 333324, Taiwan; ∥ Instrumentation Resource Center, 34914 National Yang Ming Chiao Tung University, Taipei City 112304, Taiwan; ⊥ Faculty of Pharmacy, 599817 Lac Hong University, Bien Hoa City, Dongnai 810000, Vietnam; # Faculty of Applied Sciences, 469882 Ton Duc Thang University, Ho Chi Minh City 700000, Vietnam; 7 Department of Cosmetic Science, 63113 Chang Gung University of Science and Technology, Taoyuan City 33303, Taiwan; 8 63454 National Museum of Marine Biology and Aquarium, Pingtung 944401, Taiwan; 9 Department of Marine Biotechnology and Resources, National Sun Yat-sen University, Kaohsiung 804201, Taiwan; 10 Department of Pharmacognosy, Faculty of Pharmacy, 68791 Ain-Shams University, Organization of African Unity Street, Abassia, Cairo 11566, Egypt; 11 Center for Reproductive Medicine and Sciences, Taipei Medical University Hospital, Taipei 110301, Taiwan; 12 Traditional Herbal Medicine Research Center, Taipei Medical University Hospital, Taipei 110301, Taiwan

## Abstract

Extracts of the zoanthid from *Zoanthus kuroshio* (ZK) exhibited significant *in vitro* antiosteoporotic activity in MG-63 cells, prompting the use of Global Natural Products Social Molecular Networking (GNPS) to identify alkaloid-rich fractions. Subsequently, five new zoanthamine-type alkaloids, norzoabenzaldehyde (**1**), norzoazepanol (**2**), 3-acetoxynorzoanthaminone (**3**), 11-hydroxynorzoanthamide B (**4**), and 11-hydroxyzoanthamide B (**5**), were isolated. Structural elucidation was achieved by spectroscopic analyses, time-dependent density functional theory nuclear magnetic resonance (TDDFT-NMR) calculations, and electronic circular dichroism (ECD). The alkaloid 3-hydroxynorzoanthamine exhibited notable alkaline phosphatase activity and promoted mineralization, with an IC_50_ value of 20 μM, underscoring its potential as a promising lead compound for the development of osteoporosis therapeutics.

## Graphical abstract

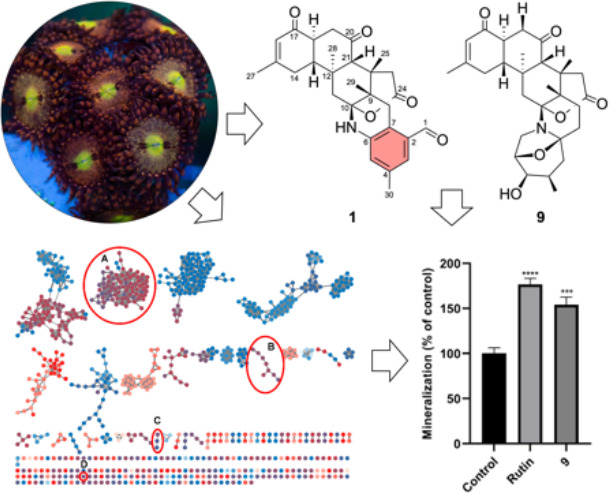

Osteoporosis is characterized as a comprehensive metabolic bone disorder featuring a significant decrease in bone mineral density and progressive deterioration of the bone microarchitectural integrity. This pathological condition arises from an imbalanced shift favoring increased bone resorption mediated by osteoclasts over bone formation facilitated by osteoblasts.^1^ This condition, along with associated fractures, is a prevalent source of morbidity and mortality among adults, particularly the elderly population.^2^ The resultant pain and skeletal deformities severely impact patients’ quality of life while imposing substantial economic burdens on families and healthcare systems. Therapeutic interventions for osteoporosis typically fall into two categories: inhibition of osteoclast activity (antiresorptive) and activation of osteoblast activity (osteoanabolic). However, antiresorptive treatments often exhibit limited efficacy in significantly increasing or restoring bone mass in patients with severe bone loss. Moreover, the sustained inhibition of bone resorption may adversely affect skeletal integrity by disrupting the ongoing remodeling of the osteoid. This issue is endorsed by a growing body of literature indicating a rise in atypical long bone fractures among patients receiving bisphosphonate therapy for periods exceeding five years.^3–5^ As a result, the development of natural products and bioactive compounds that directly promote bone formation while minimizing side effects may represent a more advantageous strategy for the treatment of osteoporosis. Thus, the identification of molecules that specifically augment osteoblast functionality is of critical clinical importance.

Alkaloids classified as zoanthamines, obtained from various zoantharian species within the *Zoanthus* genus, are characterized by their unique azepane ring structure.^6–8^ These alkaloids are characterized by their complexly functionalized molecular architecture, which features a bicyclo[3.2.1]­octane system alongside a β-methylene enone moiety integrated within an octacyclic ring structure. This chemical group can be structurally classified into two distinct categories: type I, which is characterized by the appearance of a methyl group on the carbon located at position 19, and type II, which lacks this methyl substituent.^9^ The importance of zoanthamine-type alkaloids derives not solely from their distinctive structural attributes but also from their potential pharmacological applications. Research has identified a range of pharmacological activities for zoanthamines, including antiplatelet aggregation,^10^ anti-inflammatory,^11^ antibacterial,^12^ and antiosteoporotic effects.^13,14^ However, the mechanisms by which most zoanthamines exert their effects remain largely unknown, with the exception of norzoanthamine, which has been identified as a promising antiosteoporotic compound due to its ability to reduce bone weight loss by inhibiting IL-6 secretion.^13^ Therefore, there is an urgent need to investigate the medicinal potential of these compounds for the management of osteoporosis.

To address the increasing interest in exploring the potential applications of bioactive compounds, *Zoanthus kuroshio* (ZK), a species of zoantharian, has attracted substantial attention from marine natural product researchers. Renowned for its rich repertoire of secondary metabolites, including ecdysteroids, zoanthamine alkaloids, and 2-aminoimidazole alkaloids,^15^ this species has been harvested from ecological environments and subsequently propagated under controlled circumstances to facilitate further investigation. In this research, we maintained our attempts to explore other zoanthamine-type alkaloids from the cultured ZK, including five new alkaloids, as detailed in Figure 1: norzoabenzaldehyde (**1**), norzoazepanol (**2**), 3-acetoxynorzoanthaminone (**3**), 11-hydroxynorzoanthamide B (**4**), and 11-hydroxyzoanthamide B (**5**). Four known compounds were also identified: norzoanthaminone (**6**),^16^ 3-acetoxynorzoanthamine (**7**),^17^ 3-acetoxyzoanthamine (**8**),^17^ and 3-hydroxynorzoanthamine (**9**).^18^

**Figure 1:**
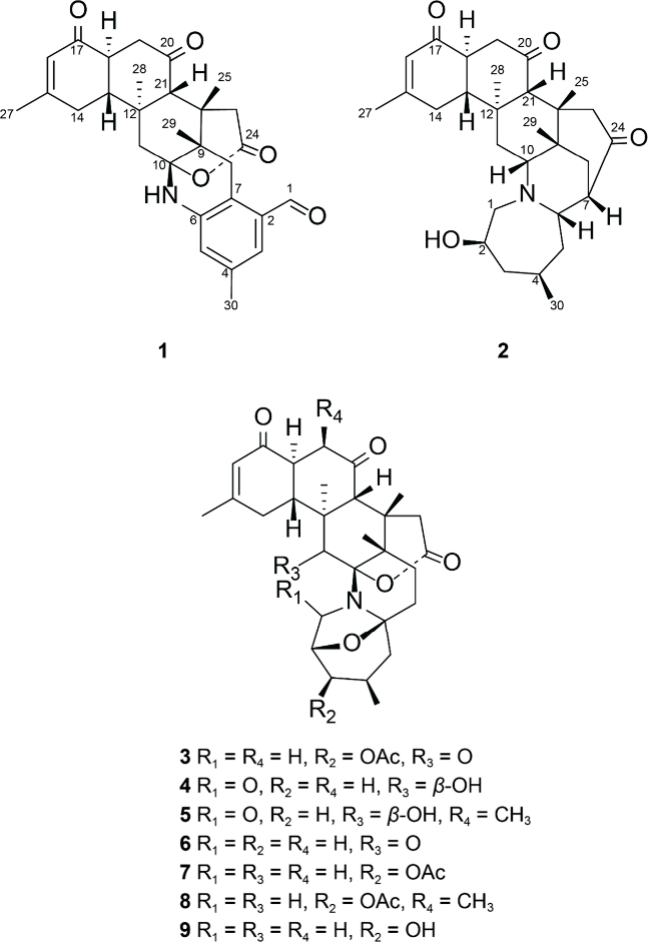
Structures of compounds **1**–**9**.

## Results and Discussion

Alkaline phosphatase (ALP) activity was used as a biomarker to evaluate the antiosteoporotic potential of the ZK extract. Four fractions, including the extract (CR), *n*-hexane fraction (HF), EtOAc fraction (EF), and H_2_O fraction (WF), were assessed through *in vitro* activity assays, with rutin serving as the positive control. The findings indicated that none of the extracts exhibited cytotoxicity toward MG-63 cells (Figure 2A), allowing for continued evaluation of ALP activity and mineralization. With a concentration of 10 μg/mL, those fractions demonstrated ALP activity except for WF, with EF showing the highest activity level at 269.58 ± 26.97%, followed by CR at 164.98 ± 16.12% and HF at 163.00 ± 8.48% (Figure 2B). Subsequently, these fractions were also tested for mineralization, revealing that only EF had significant effects, as indicated by a value of 150.67 ± 11.13%, and it exhibited a darker staining color compared to the other fractions and the control (Figure 2C, D). These findings suggest that the compounds in the EF fraction may possess significant potential as antiosteoporotic agents.

**Figure 2:**
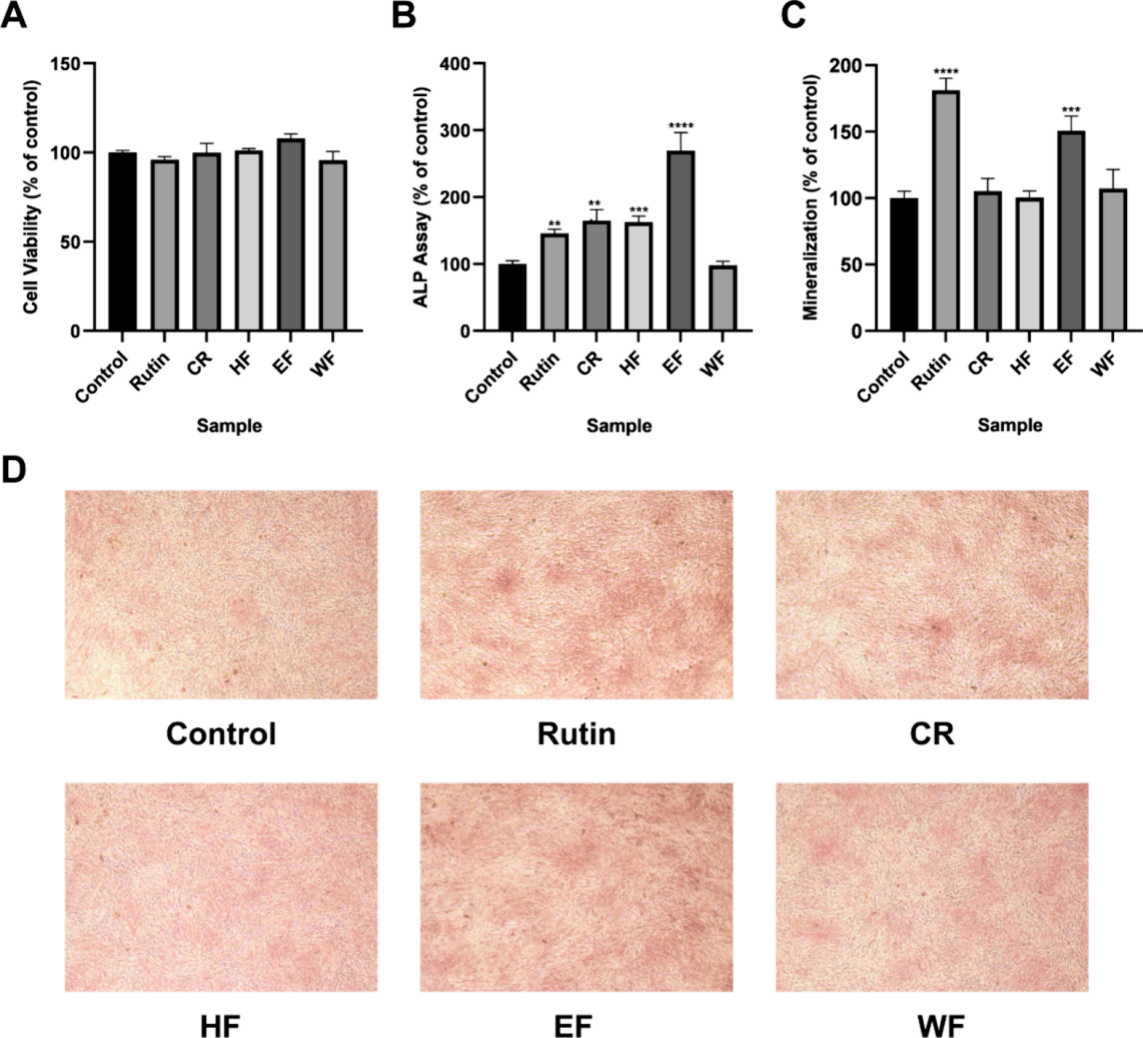
Results of four extracts (CR, HF, EF, and WF) on ALP activity and mineralization in MG-63 cells. (A). Cell viability was evaluated utilizing the MTT assay. (B) ALP activity was assessed in MG-63 cells treated with 10 μg/mL of each extract and 60 μg/mL of rutin. (C) MG-63 cells were treated with 10 μg/mL of each extract and 60 μg/mL of rutin for quantitative analysis of the degree of mineralization. (D) Mineralized nodule formation was evaluated through alizarin red staining. The data are presented as the mean ± SD with *n* = 3. **p < 0.01, ***p < 0.001 and ****p < 0.0001 compared to control cells.

To facilitate the separation of zoanthamine alkaloids, the EF fraction was subjected to ^1^H NMR analysis, as illustrated in Supporting Information Figure S1. The spectrum revealed a characteristic signal corresponding to the sp^2^ methine at C-16, which is a structural hallmark of zoanthamine alkaloids. Based on this observation, a classical molecular networking (MN) approach was subsequently employed to support compound annotation and dereplication. Such a methodology enabled comprehensive analysis and the construction of MN profiles for all fractions through the use of MS/MS analysis performed on the GNPS platform. The acquired profiles were presented derived from the *m*/*z* values of the precursor ions, as illustrated in Figure 3A. The detailed MN analysis highlighted several clusters that exhibited the characteristic molecular weights associated with the anticipated mass range of 400–650 *m*/*z*, determined from the literature review about zoanthamine alkaloids.^15^ Among the identified clusters, four distinct groups (A–D) were annotated as containing 30 compounds previously reported in the literature to belong to the zoanthamine alkaloid family. These compounds include norzoanthamine, kuroshine B, 11β-chloro-11-deoxykuroshine A, zoanthamine, norzoanthamide B, 3-hydroxynorzoanthamine, 3-acetoxynorzoanthamine, and 3-acetoxyzoanthamine, among others,^15^ as detailed in Supporting Information Table S1. These annotated clusters were subsequently utilized as targets for isolation efforts. Furthermore, an in-depth investigation was conducted to determine the specific fractions to which these compounds belonged. The analysis revealed that the preponderance of zoanthamine alkaloids were concentrated in EF, followed by WF, with no nodes detected in HF (Figure 3B). Within the *m*/*z* range of 400–650, and in conjunction with nodes exhibiting the color corresponding to the EF fraction, a substantial number of molecular features remained unannotated despite spectral network analysis. The persistence of these uncharacterized nodes suggests the presence of unidentified metabolites within the sample matrix. Their consistent detection, coupled with distinct fractionation profiles, underscores the potential of these features to represent novel or structurally unique compounds. As such, these unannotated nodes warrant further targeted investigation through advanced dereplication strategies and structural elucidation techniques, potentially contributing to the expansion of the known metabolome. Based on these findings, the EF fraction was prioritized and selected for further purification processes due to its high content of alkaloid-type compounds and its pronounced effect on ALP activity. This strategic decision facilitated the successful identification of new compounds within fraction EF (clusters E, F, G, H, and I). The integration of MN with MS/MS analysis proved to be a powerful approach to the targeted isolation and discovery of zoanthamine alkaloids, demonstrating the utility of this method in the field of natural product research.

**Figure 3:**
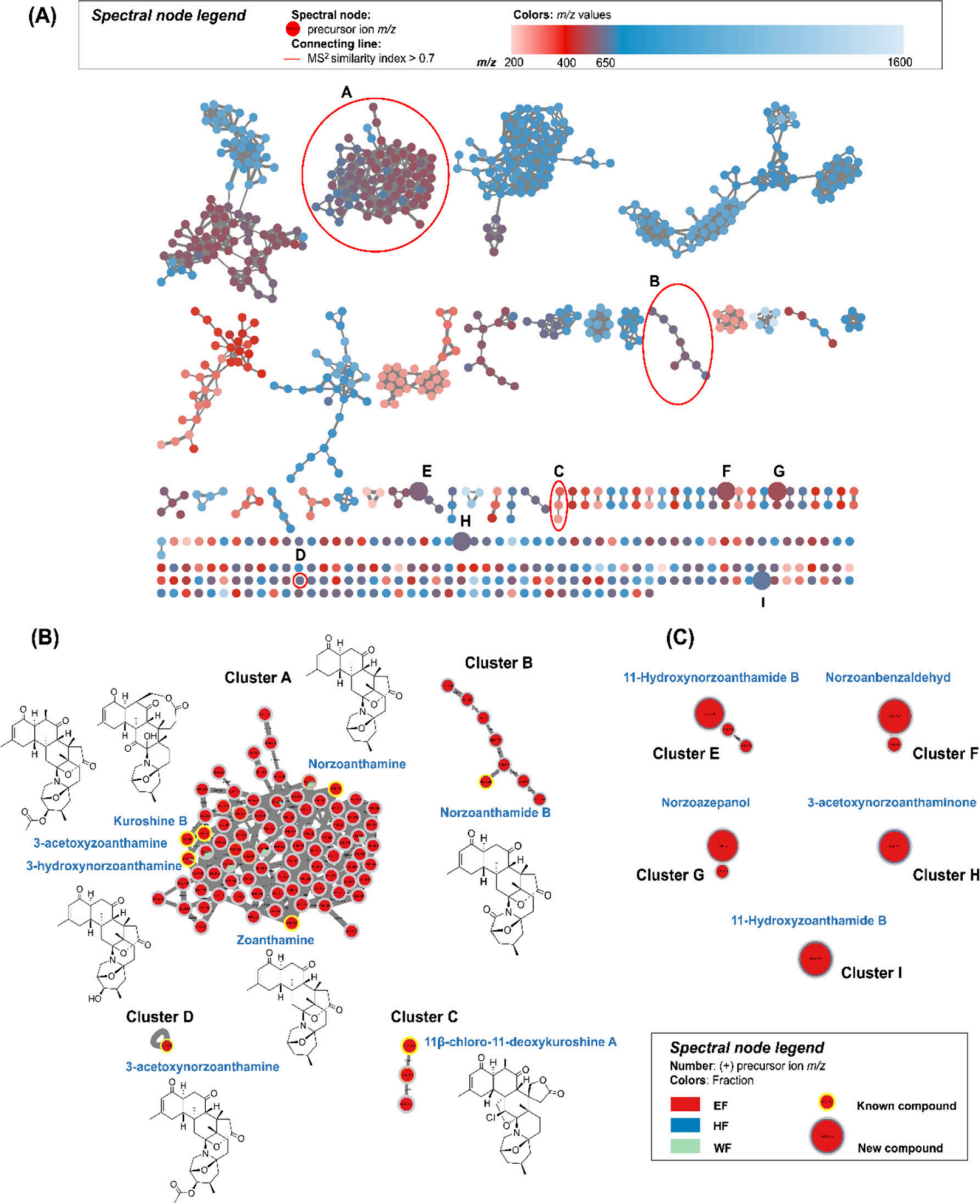
MS/MS molecular networking (MN) reveals the chemical diversity of zoanthamine alkaloids derived from cultured *Zoanthus kuroshio*. (A) The MN profile is color-coded based on *m*/*z* values. (B) The unique zoanthamine alkaloids identified within clusters A–D are highlighted. (C). Cluster E–I containing nodes corresponding to new compounds.

Compound **1** was isolated as a white, amorphous powder. HRESIMS analysis revealed a predominant protonated molecular ion peak [M + H]^+^ at *m*/*z* 476.2444 (calculated for 476.2437, C_29_H_34_NO_5_). Initial assessment of the NMR spectra exhibited specific signals consistent with the zoanthamine family, as suggested by mass profile: 29 signals of carbon were recognized as 12 quaternary carbons (assigned as three carbonyl groups, four sp^3^ carbons, and five sp^2^ carbons) and five methyl groups, five methylenes, six methine groups (including four sp^2^ carbon) (Table 1). The deficiency of a methyl doublet, typically introduced in zoanthamines, indicated the vanishing of CH_3_-26 at C-19; thus, **1** was classified as a norzoanthamine-type. To ensure consistency in the NMR table, we retained the zoanthamine numbering for methyl groups 27, 28, 29, and 30. In accordance with formula of structure, the unsaturations should originate from an aromatic ring and five additional rings. The analysis of the HSQC and COSY profiles exposed the presence of two distinct systems known as ^1^H–^1^H spin. The initial structural moiety was effectively established from methylene proton H-3 (δ_H_ 7.15), which exhibited a *meta* coupling (*J* = 1.8 Hz) with H-5 (δ_H_ 6.97), indicating their connectivity within the aromatic ring system. Constructed through the coupling of H-13 (δ_H_ 2.44) with H_2_-14 (δ_H_ 2.11) and H-18 (δ_H_ 2.74), the second moiety was linked to H_2_-19 (δ_H_ 2.84) (Figure 4). However, given the presence of numerous quaternary carbons, the HMBC correlations were pivotal in the structural elucidation of this molecule. The preliminary analytical connection between rings A and B (Figure 4) was established in a manner analogous to that of norzoanthamine, supported by critical HMBC correlations (H-18/C-17; H-19/C-17, C-20; H-28/C-13) and the previously discussed second fragment. The connection between ring B and ring C was established through the following correlations: the hydrogen of the carbon situated at position 28 with carbon atoms 11, 12, and 21. Rings D and E were attached with ring C as follows: correlations of H_3_-29 with C-8, C-9, C-10, and C-22 and those of H_3_-25 with carbon atoms at positions 9, 21, and 23, where H_2_-23 connected with δ_c_ 172.5 (C-24), which demonstrated that ring D contained a carbonyl group. Moreover, ring E connected to ring C through correlations of C-11 and C-9 with δ_H_ 7.91 (H–N). Ultimately, key HMBC correlations (H_3_-30/C-5, C-4, C-3; H-1/C-2, C-3, C-7), along with the connections of H–N and H_2_-8 to C-6 and C-7, confirmed the linkage between rings E and F. These correlations also established one methyl group and an aldehyde group in the final ring. The stereostructural connection within compound **1** was primarily determined from proton spin coupling constants as well as dipolar interactions detected by NOESY correlations that matched those of norzoanthamine. Thus, the relative configurations within the C-9 to C-29 fragment were assigned as 9S*,10R*,12S*,13R*,18S*,21S*,22S* as determined by NOE correlations of H-21 with H_3_-29, H_3_-25, and H-13, along with the correlation of H-18 with H_3_-28 and H–N with H_3_-29 (Figure 3).

**Table 1:** ^1^H and ^13^C NMR Data for Compounds **1** and **2**

	**1** ^a^		**2** ^b^	
no.	δ_H_, mult (*J* in Hz)	δ_C_	δ_H_, mult (*J* in Hz)	δ_C_
1	10.28, *s*	194.3, CH	3.17, *m*	62.1, CH_2_
			2.17, *m*	
2		141.6, C	3.95, *m*	65.8, CH
3	7.15, br *s* (1.8)	127.0, CH	2.07, *m*	42.9, CH_2_
			1.02, *m*	
4		137.6, C	1.62, *m*	25.0, CH
5	6.97, br *s* (1.8)	121.3, CH	1.67, *m*	48.5, CH_2_
6		136.7, C	2.63, *m*	69.2, CH
7		118.3, C	2.22, *m*	54.9, CH
8	3.64, *d* (17.9)	28.4, CH_2_	2.17 (α), *m*	39.1, CH_2_
	3.29, *d* (17.8)		1.47 (β), *m*	
9		38.1, C		36.4, C
10		93.7, C	2.41, *m*	69.6, CH
11	2.40, *d* (14.4)	43.8, CH_2_	2.13, *m*	34.9, CH_2_
	2.27, *d* (14.5)		1.80, *m*	
12		40.1, C		40.5, C
13	2.44, *m*	53.1, CH	2.10, *m*	53.5, CH
14	2.11, *m*	31.9, CH_2_	2.28, *m*	31.8, CH_2_
			2.38, *m*	
15		161.1, C		160.6, C
16	6.04, *s*	125.9, CH	5.90, *s*	125.6, CH
17		198.8, C		199.2, C
18	2.74, *m*	46.7, CH	2.58, *m*	46.1, CH
19	2.84, *m*	43.5, CH_2_	2.39, *m*	42.8, CH_2_
			2.68, *m*	
20		210.2, C		209.5, C
21	3.13, *s*	59.9, CH	2.61, *s*	61.8, CH
22		37.0, C		41.0, C
23	3.96, *d* (20.3)	38.2, CH_2_	3.45, *d* (15.4)	49.5, CH_2_
	2.91, *d* (20.3)		3.15, *d* (14.2)	
24		172.5, C		213.8, C
25	1.26, *s*	21.3, CH_3_	0.99, *s*	23.2, CH_3_
26				
27	1.82, *s*	24.3, CH_3_	2.02, *s*	24.5, CH_3_
28	1.10, *s*	17.7, CH_3_	1.32, *s*	19.8, CH_3_
29	1.19, *s*	18.1, CH_3_	0.95, *s*	24.5, CH_3_
30	2.21, *s*	21.1, CH_3_	0.86, *d* (6.5)	24.1, CH_3_
H–N	7.91, *s*			

a Spectra recorded at 600 and 150 MHz in C_5_D_5_N.

b Spectra recorded at 600 and 150 MHz in CDCl_3_.

**Figure 4:**
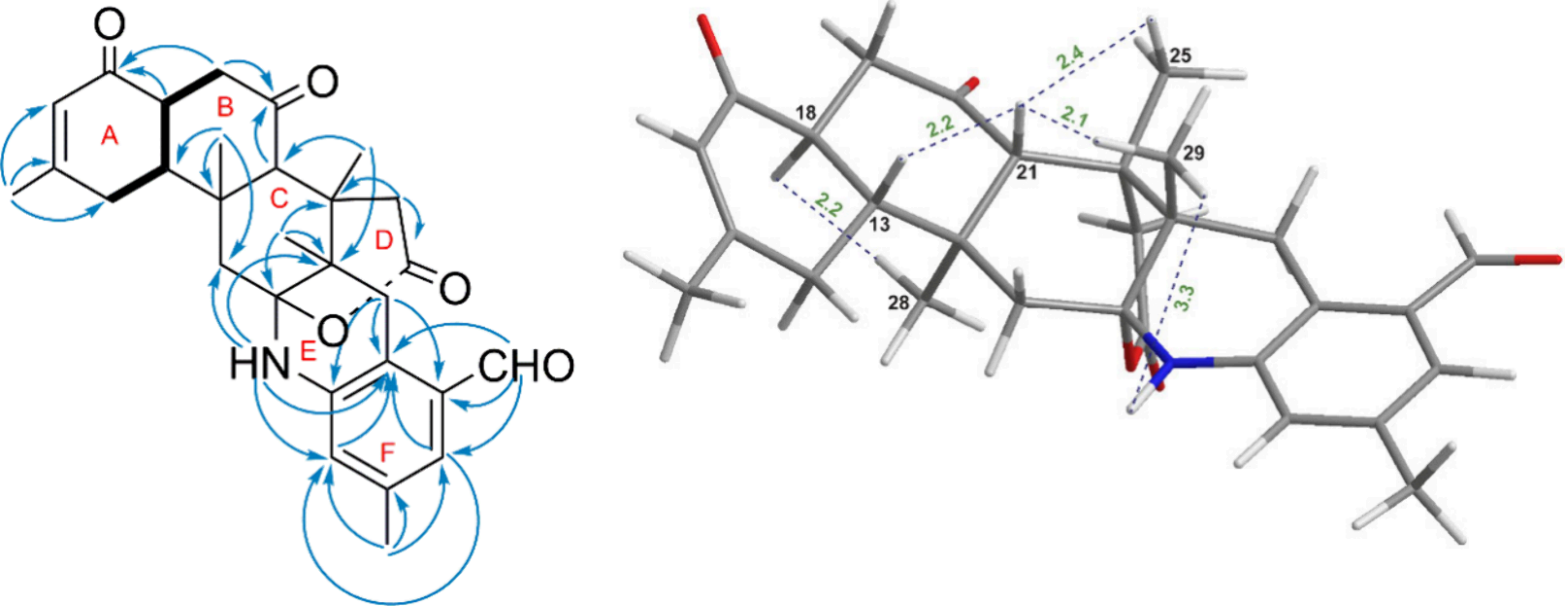
Key COSY, HMBC, and NOESY correlations of compound **1** (ring nomenclature is shown using red letters).

Compound **2** was isolated as a white, amorphous powder. HRESIMS analysis indicated a protonated molecular ion peak [M + H]^+^ at *m*/*z* 468.3120 (calculated for 468.3114, C_29_H_42_NO_4_,). From the analysis of the NMR data of compound **2**, 29 carbon signals were classified into five methyl groups, eight methylenes, nine methine groups (including one sp^2^ carbon), and seven quaternary carbons (assigned as three carbonyl groups, three sp^3^ carbons, and one sp^2^ carbon) (Table 1); the remaining unsaturations were attributed to six rings. Analysis of HSQC and COSY data indicated three distinct proton–proton spin systems. The first moiety was built on the key COSY correlations (H_2_-1/H-2/H_2_-3/H-4/H_2_-5/H-6/H-7/H-8; H-4/H_3_-30). The second fragment (H_2_-14/H-13/H-18/H_2_-19) was established similarly to compound **1**; the third fragment was constructed through the coupling of methylene H_2_-11 with H-10 (Figure 5). Specifically, the absence of an oxygenated substituent at C-10 is supported by the significant upfield shift of its carbon signal to δ_C_ 69.6 compared to the corresponding signal in compound **1**. The connectivity among rings B, C, D, and E in **2** exhibited characteristics similar to those observed in compound **1**; however, a significant difference in ring D was noted at C-24, where the carbonyl group was positioned at a higher chemical shift of 213.8 ppm compared to compound **1**. The relative configurations of the carbon atoms at positions 9, 13, 18, 21, and 22 in compound **2** were established via the NOESY experiment and further validated through comparison with the NMR shift values. The NOE correlation of H_3_-29 (δ_H_ 0.95) with H-10 (δ_H_ 2.41), along with the correlations of H-8_β_ (δ_H_ 1.47) with H-10 and H-6 (δ_H_ 2.63), which in turn correlated with H-7 (δ_H_ 2.22), indicated that these protons were predominantly β-oriented (Figure 5). The stereochemistry at C-2 was determined by calculating the ^13^C NMR chemical shifts for various configurations by using Gaussian 16 software. These calculations generated direct correlations between the predicted values and the experimental data (Figure 6). The linear regression analysis between the computed values for 2*S*-**2** and the experimental data for compound **2** yielded a correlation coefficient (*R* ^2^) of 0.9985, while for 2*R*-**2**, the R^2^ value was 0.9990. To further verify the configuration of C-2, the 2*R*-**2** and 2*S*-**2** configurations were analyzed using the GIAO NMR method. The results were cross-validated with the sDP4+, uDP4+, and DP4+ methods, all showing a perfect match ratio of 100.00% for the 2*R*-**2** configuration (Supporting Information Figure S53). Additionally, the absolute configurations of the stereogenic centers in compound **2** were confirmed through ECD experiments, with the predicted ECD spectrum for the 2*R*-**2** enantiomer closely matching the experimentally obtained spectrum (Figure 7). The configuration was assigned to C-2, thereby establishing the structure of compound **2** as 2*R*,4*R*,6*S*,7*S*,9*S*,10*R*,12*S*,13*R*,18*S*,21*S*,22*S*.

**Figure 5:**
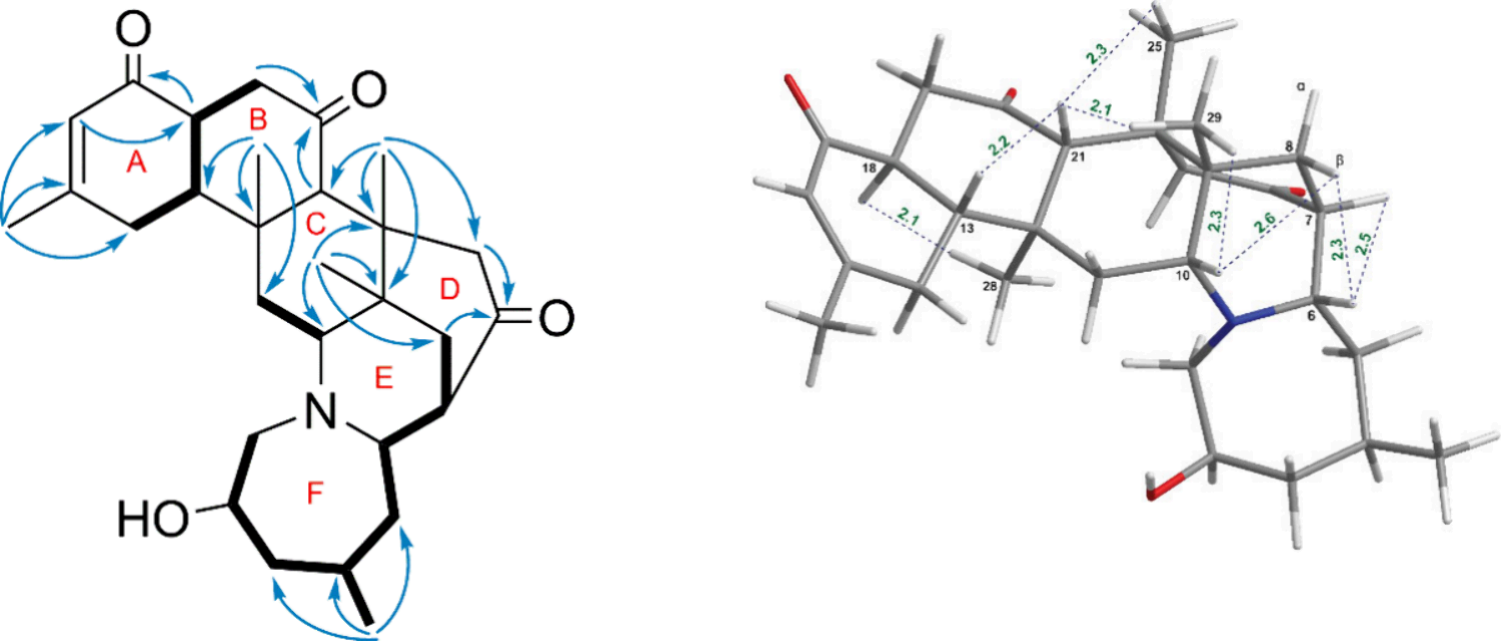
Key COSY, HMBC and NOESY correlations of compound **2**.

**Figure 6:**
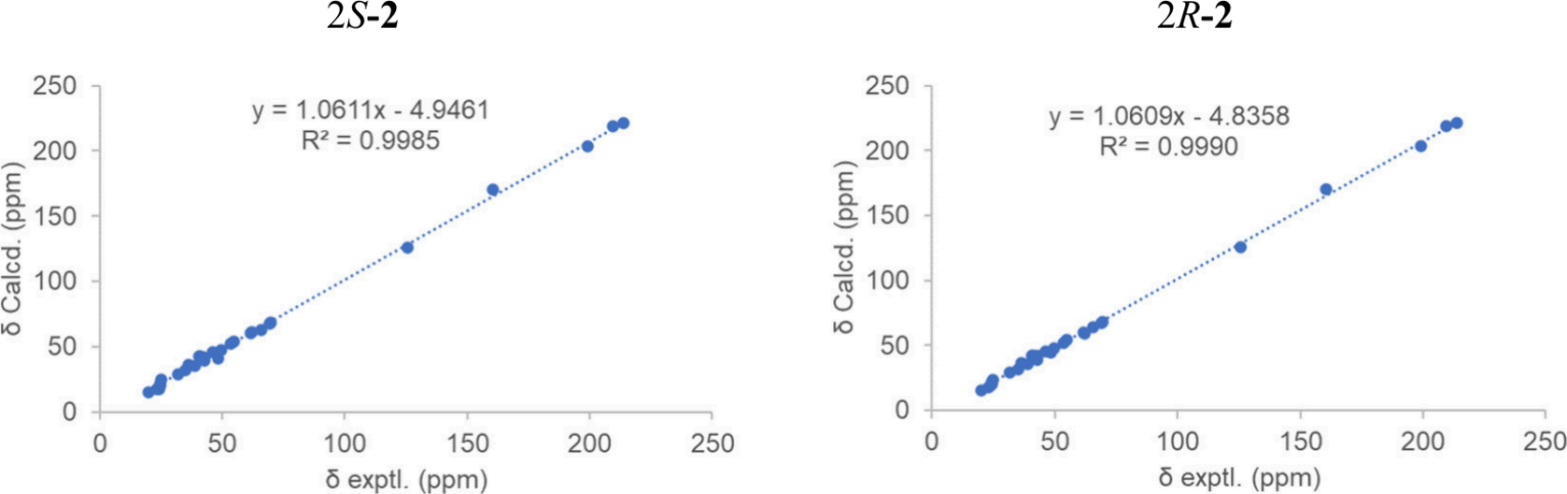
Linear correlations of calculated 2*S*-**2** and 2*R*-**2** with the experimental ^13^C NMR chemical shifts for compound **2**.

**Figure 7:**
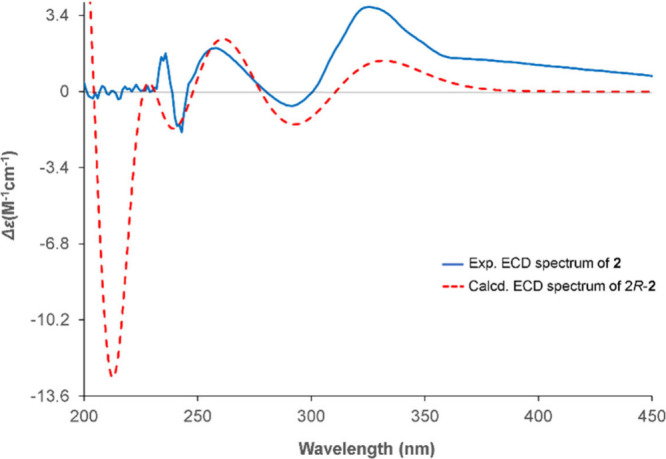
Experimental ECD spectra of **2** vs calculated values.

Compound **3** was isolated as a white, amorphous powder. HRESIMS analysis revealed a protonated molecular ion peak [M + H]^+^ at *m*/*z* 554.2757 (calculated for 554.2754, C_31_H_40_NO_8_). According to MS and NMR data, five methyl groups (four methyl singlets and a methyl doublet), seven methylene and methine groups, and 11 quaternary carbons (comprising two carboxylic, three carbonyl, and two olefinic carbons) were identified (Table 2). The spectrum profile for compound **3** exhibited a high degree of similarity to that of norzoanthamine, with the primary distinction being the appearance of a methylcarbonyl group at C-3. This feature was corroborated by a significant increase in the chemical shift of the methine signal (H-3, δ_H_ 4.66) and the emergence of a methyl singlet at δ_H_ 2.13, further supported by key HMBC correlations between H-3/C-1′ and H_3_-2′/C-1′. Additionally, the deficiency of H_2_ signals at C-11 suggested the introduction of an additional ketone signal at δ_C_ 202.5, corroborated by the HMBC correlation between H_3_-28 and C-11 (Figure 8). The NOESY cross-peaks at H-21 and H-18 closely resembled those observed in compounds **1** and **2**.

**Table 2:** ^1^H and ^13^C NMR Data for Compounds **3**–**5**

	**3** [Table-fn t2fn1]		**4** [Table-fn t2fn1]		**5** [Table-fn t2fn2]	
no.	δ_H_, mult (*J* in Hz)	δ_C_	δ_H_, mult (*J* in Hz)	δ_C_	δ_H_, mult (*J* in Hz)	δ_C_
1	(β) 4.03, *m*	47.0, CH_2_		175.9, C		175.8, C
	(α) 2.99, d (9.3)					
2	4.57, *m*	76.0, CH	4.32, *q* (2.1, 1.8)	77.4, CH	4.33, *q* (3.8, 2.1)	77.4, CH
3	4.66, *m*	72.5, CH	1.89, *m*	32.3, CH_2_	1.90, *m*	32.6, CH_2_
			1.47, *m*		1.47, *m*	
4	2.48, *m*	25.9, CH	2.23, *m*	24.1, CH	2.21, *m*	24.1, CH
5	1.83, *m*	40.0, CH_2_	2.20, *m*	40.6, CH_2_	2.22, *m*	40.6, CH_2_
	1.40, *m*		1.20, *m*		1.20, *m*	
6		90.9, C		95.2, C		95.2, C
7	1.85, *m*	29.7, CH_2_	1.88, *m*	30.0, CH_2_	1.88, *m*	30.1, CH_2_
	1.25, *m*		2.14, *td* (5.0, 13.7)		2.14, td (4.9, 13.8)	
8	1.89, *m*	24.2, CH_2_	1.69, *m*	24.5, CH_2_	1.66, *m*	24.6, CH_2_
	1.62, *m*		1.57, *m*		1.58, *m*	
9		43.4, C		39.6, C		39.8, C
10		103.9, C		97.3, C		97.3, C
11		202.5, C	4.77, *d* (6.1)	73.5, CH	4.76, *d* (6.1)	73.4, CH
12		53.5, C		44.0, C		43.6, C
13	2.59, *m*	48.3, CH	2.95, *m*	46.6, CH	3.17, *m*	41.2, CH
14	3.36, *dd* (3.4, 14.7)	34.5, CH_2_	2.27, *m*	32.6, CH_2_	2.19, *m*	31.0, CH_2_
	2.26, *m*		2.50, *dd* (4.1, 17.9)		2.47, *dd* (4.2, 17.6)	
15		162.4, C		161.7, C		161.9, C
16	5.91, *s*	124.7, CH	5.89, *s*	125.1, CH	5.89, *s*	126.4, CH
17		198.1, C		199.0, C		197.9, C
18	2.71, *m*	47.0, CH	2.63, *m*	45.8, CH	2.58, *dd* (5.2, 8.72)	47.6, CH
19	2.60, *m*	42.8, CH_2_	2.69, *dd* (5.8, 13.6)	43.0, CH_2_	3.04, *m*	46.1, CH
	2.52, *m*		2.44, *m*			
20		207.9, C		210.3, C		213.3, C
21	2.97, *s*	59.6, CH	3.06, *s*	56.0, CH	3.43, *s*	50.5, CH
22		37.4, C		36.5, C		36.0, C
23	4.14, *d* (20.5)	35.1, CH_2_	3.77, *d* (20.6)	37.0, CH_2_	3.78, *d* (20.6)	37.0, CH_2_
	2.55, *m*		2.43, *d* (20.7)		2.43, *d* (20.6)	
24		170.9, C		170.1, C		170.1, C
25	1.03, *s*	21.4, CH_3_	1.04, *s*	20.8, CH_3_	1.00, *s*	20.5, CH_3_
26					1.18, *d* (7.0)	13.4, CH_3_
27	2.04, *s*	24.6, CH_3_	1.99, *s*	24.6, CH_3_	1.98, *s*	24.8, CH_3_
28	1.28, *s*	16.6, CH_3_	1.04, *s*	18.0, CH_3_	1.02, *s*	17.7, CH_3_
29	1.08, *s*	17.5, CH_3_	1.38, *s*	17.4, CH_3_	1.41, *s*	17.4, CH_3_
30	0.88, *d* (6.7)	16.3, CH_3_	1.00, *d* (6.1)	21.4, CH_3_	1.00, *d* (5.5)	21.4, CH_3_
1′		171.2, C				
2′	2.13, *s*	21.2, CH_3_				

c Spectra recorded at 600 and 150 MHz in CDCl_3_.

d Spectra recorded at 800 and 200 MHz in CDCl_3_.

**Figure 8:**
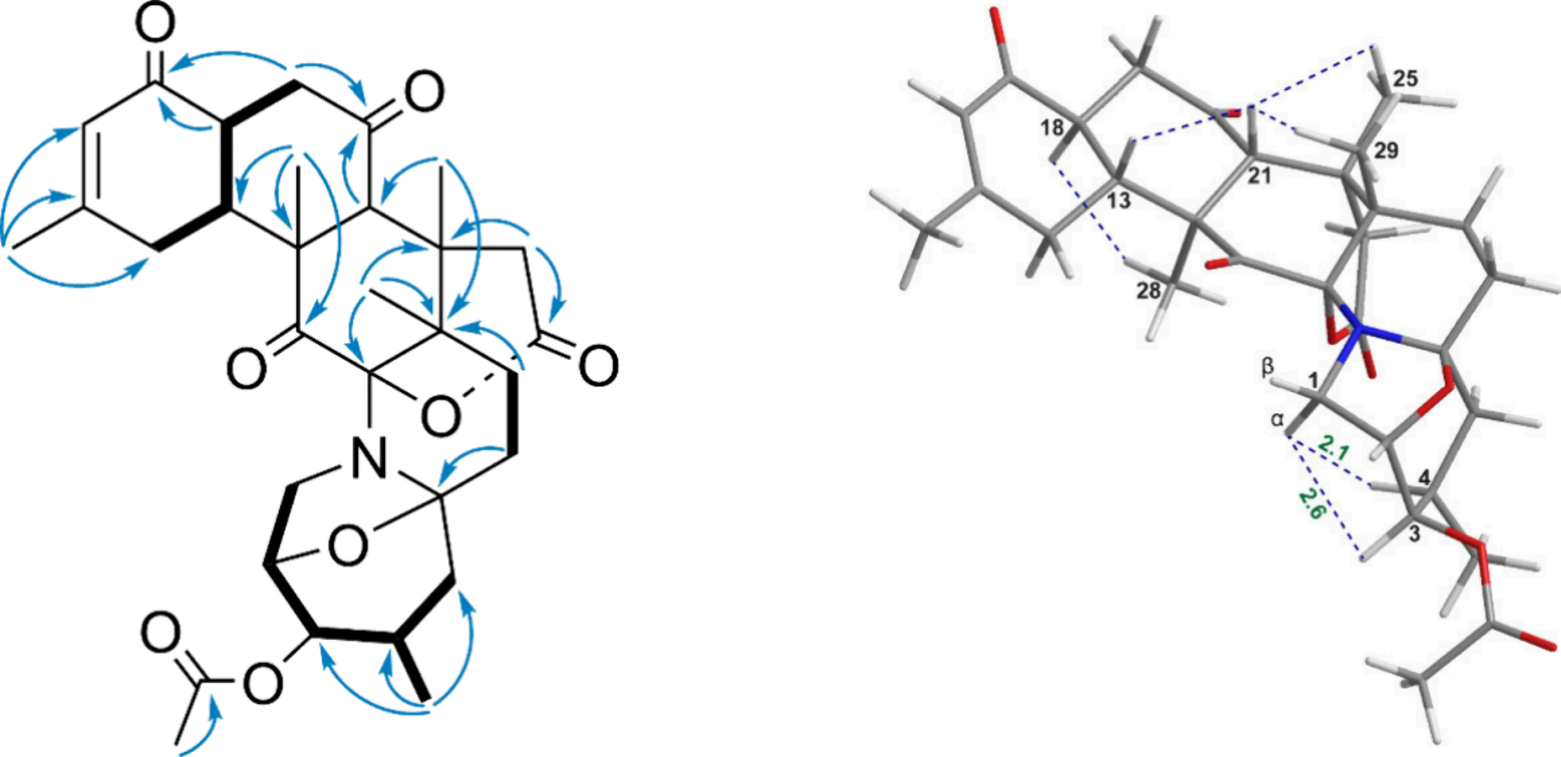
Key COSY, HMBC, and NOESY correlations of compound **3**.

Moreover, the NOE correlation between H-3 (δ_H_ 4.66) and the proton signal at δ_H_ 2.99, identified as H-1_α_ due to its connection with H-4 (δ_H_ 2.48), suggested a relatively α-oriented H-3. As described above, the relative configuration of **3** was 2S*,3R*,4R*,6S*,9S*,10S*,12S*,13R*,18S*,21R*,22S* (Figure 3).

Compound **4** was isolated as a white, amorphous powder. HRESIMS analysis showed a protonated molecular ion peak [M + H]^+^ at *m*/*z* 512.2653 (calculated for 512.2648, C_29_H_38_NO_7_). A comparable evaluation of the NMR spectra for **4**, relative to norzoanthamine, revealed a carboxylic signal at δ_C_ 175.9 for C-1, along with the lack of distinctive proton signals associated with the isolated methylene group of carbon atom at position 11 in norzoanthamine, which was replaced by a newly identified methine group (δ_H_ 4.77, δ_C_ 73.5). This proton signal exhibited COSY connectivity exclusively with the hydroxyl proton, displaying a coupling constant of 6.1 Hz. The proton at C-11 exhibited interactions with carbons at positions 9, 10, 28, 12, and 21, while the carbon at C-11 demonstrated connectivity with H_3_-28, as evidenced by the HMBC correlations (Figure 9). The relative stereochemistry at C-11 was verified as S* based on NOE correlations of H-11 with H_3_-28. Consequently, the relative configuration of **4** was assigned as 2R*,4S*,6S*,9S*,10S*,11S*,12S*,13R*,18S*,21R*,22S* based upon analysis of NMR data recorded for compound **3** and by comparison with data of norzoanthamine.^13^

**Figure 9:**
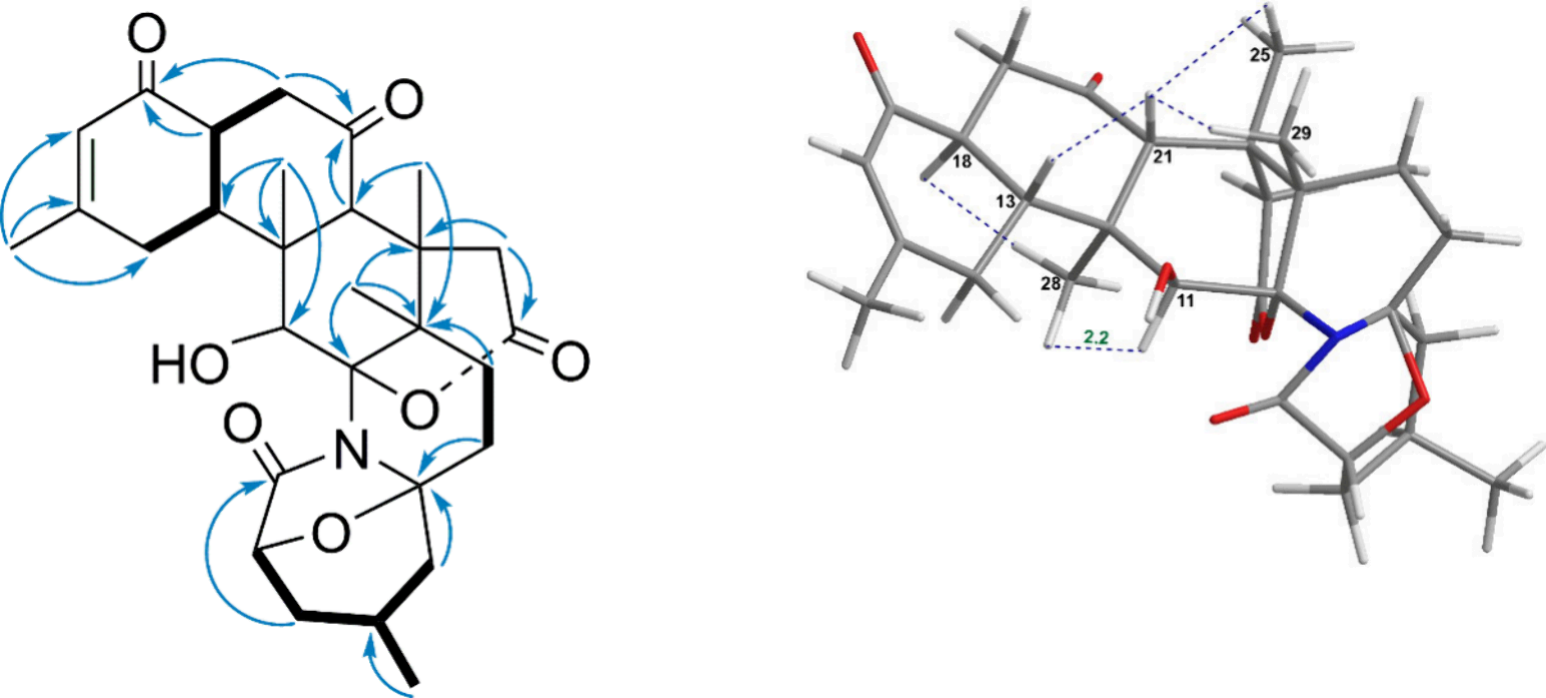
Key COSY, HMBC ,and NOESY correlations of compound **4**.

Compound **5** was isolated as a white, amorphous powder. Its HRESIMS analysis indicated a protonated molecular ion peak [M + H]^+^ at *m*/*z* 526.2818 (calculated for 526.2805, C_30_H_40_NO_7_). Therefore, compound **5** is a homologue of compound **4** showing closely related NMR signals, as well as the appearance of a ketone group at C-1 and a hydroxy group at C-1 (Table 2). An extra signal corresponding to a proton at 1.18 (*d*, *J* = 7.0 Hz, H_3_-26), identified as a methyl group, indicated that compound **5** was classified as a type I zoanthamine. The positioning of the proton at C-19 was further corroborated by observing key HMBC correlations (H-19/C-26; H_3_-26/C-18/C-19) (Figure 10). The NOE correlation between H_3_-26 and H-21 indicates that the relative configuration at C-19 is R*, while the configuration at the other chiral center is consistent with that of compound **4**, supported by similar observations.

**Figure 10:**
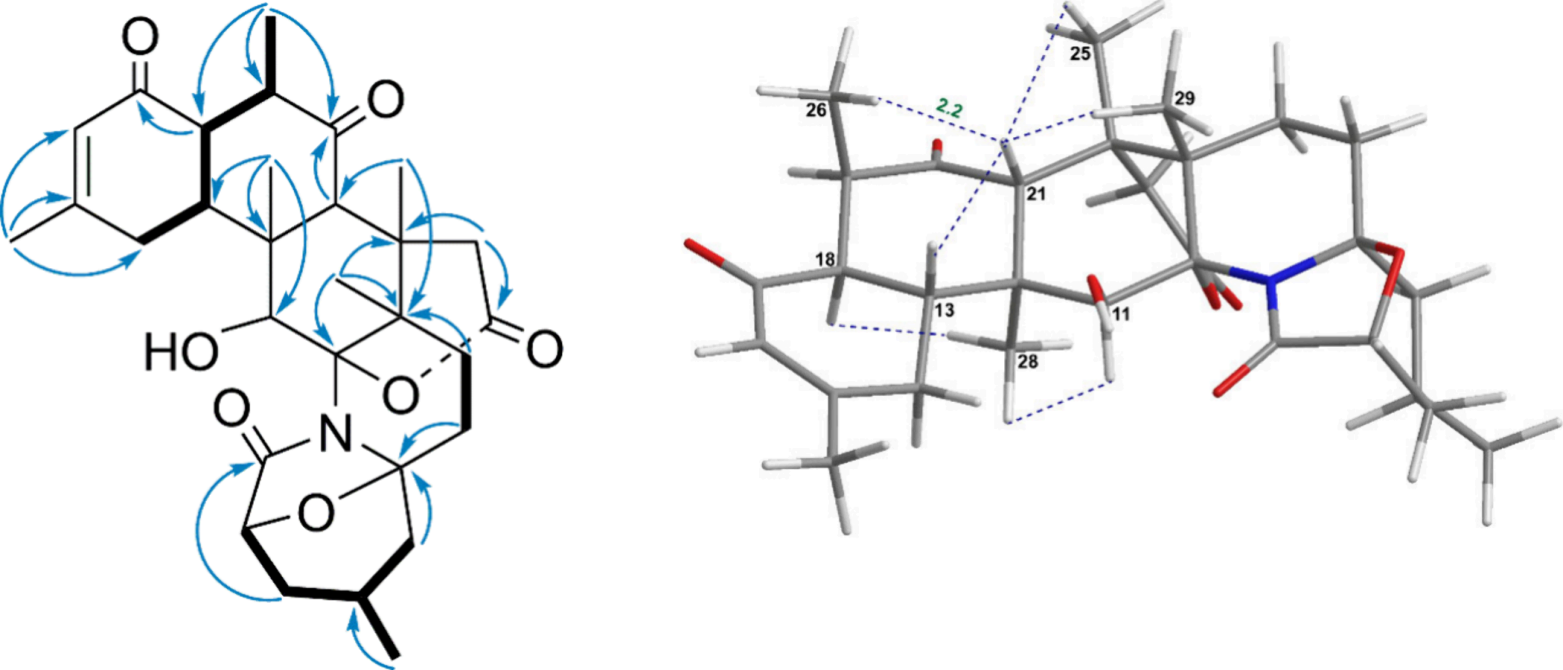
Key COSY, HMBC, and NOESY correlations of compound **5**.

In *in vitro* bioassays, the potential toxic effects of nine compounds on the growth of MG-63 cells were assessed using a cell viability assay based on the MTT method. As shown in Figure 11A, none of the compounds inhibited MG-63 cell proliferation at 20 μM. Following this, the nine isolated compounds were assessed for their impact on ALP activity, with results indicating that compound **9** significantly enhanced ALP levels, reaching a value of 197.38 ± 23.58% compared to the control group, while the other compounds demonstrated no significant effect on ALP activity (Figure 11B). Further investigations into mineralization demonstrated that a concentration of 20 μM of compound **9** resulted in an increase in the calcium levels in the mineralized matrix by 154.15 ± 8.43%, as illustrated in Figure 11C. This increase was further supported by the darker coloration observed relative to the control groups, signifying elevated calcium content, as detailed in Figure 11D. Notably, norzoanthamine hydrochloride remains the only compound in this class reported to prevent bone fractures, presumably through the inhibition of interleukin-6 production and osteoclastogenesis *in vivo*.^13,14^

**Figure 11:**
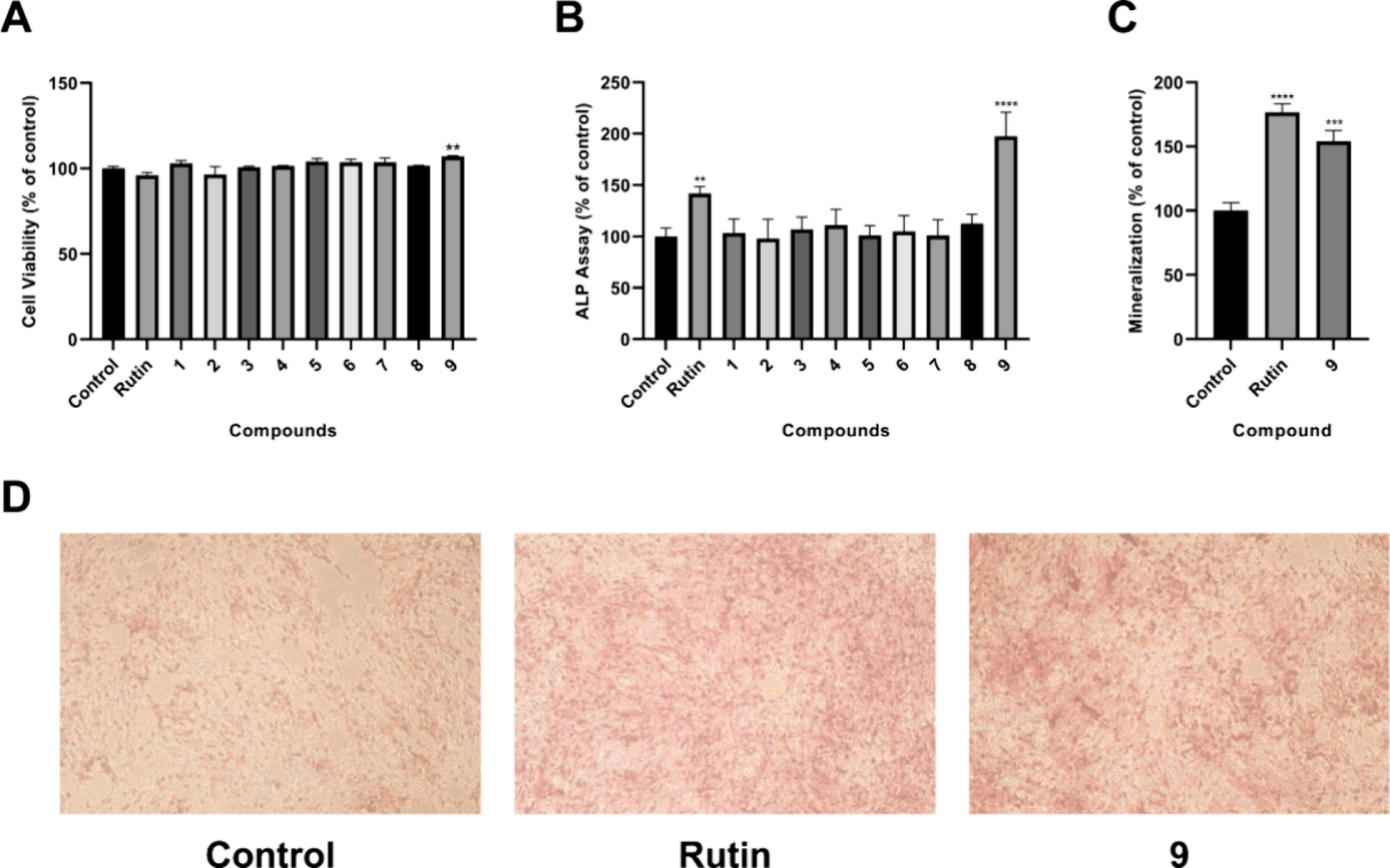
Results of isolated compounds **1**–**9** in terms of cell viability, ALP activity, and mineralization in MG-63 cells. (A) Cell viability was evaluated by utilizing the MTT assay. (B) ALP activity was assessed in MG-63 cells treated with 10 μg/mL of each compound. (C) MG-63 cells were treated with 10 μg/mL of compound **9** for the quantitative analysis of mineralization levels. (D) Mineralized nodule formation was evaluated through alizarin red staining. Rutin (60 μg/mL) was used as a positive control. The data are presented as the mean ± SD with *n* = 3. **p < 0.01, ***p < 0.001 and ****p < 0.0001 compared to control cells.

## Conclusion

In summary, the investigation of *Zoanthus kuroshio* through *in vitro* bioactivity assays using MG-63 cells, combined with GNPS-based molecular networking analysis, provided valuable insights into its potential as a source of bioactive compounds for osteoporosis treatment. Five new zoanthamine-type alkaloids were discovered, namely, norzoabenzaldehyde (**1**), norzoazepanol (**2**), 3-acetoxynorzoanthaminone (**3**), 11-hydroxynorzoanthamide B (**4**), and 11-hydroxyzoanthamide B (**5**). The structures of these compounds were elucidated through comprehensive analyses of spectral data alongside computational calculations. Compound **9** demonstrated significant antiosteoporotic activity, as evidenced by assays measuring ALP activity and mineralization. These discoveries highlight the therapeutic potential of *Zoanthus kuroshio* and enhance our understanding of marine natural products as promising candidates for osteoporosis treatment, underscoring the importance of cultivating this species for its exploitation in both general bioassays and specific pathological studies related to osteoporosis.

## Experimental Section

### General Experimental Procedures

A JASCO P-2000 polarimeter (JASCO, Tokyo, Japan) was used to perform optical rotation measurements in methanol. Ultraviolet (UV) spectra were acquired using a a UV-2600 UV–vis spectrophotometer (Shimadzu, Tokyo, Japan), and electronic circular dichroism (ECD) spectra were recorded with a JASCO J-715 spectropolarimeter (JASCO, Tokyo, Japan). Infrared spectra (IR) were recorded with an IRAffinity-1S FTIR Spectrometer (Shimadzu, Tokyo, Japan). Nuclear magnetic resonance (NMR) experiments were administered by an Agilent 600 MHz NMR spectrometer (Agilent, Santa Clara, CA, USA), with chloroform-*d* and pyridine-*d*_*5*_ employed as internal locks. High-resolution electrospray ionization mass spectrometry (HRESIMS) data were obtained by utilizing a system known as Waters LC/QTOF SYNAPT G2 (Waters Corporation, Milford, MA, USA). A SepaBean machine 2 (China), fitted with either a normal-phase silica gel or a reverse-phase C_18_ column, was used to conduct fast column chromatography (FCS). Using a semipreparative Shimadzu LC-2050 HPLC instrument (Shimadzu, Kyoto, Japan), the separation of extracts was accomplished by a reverse-phase C_18_ column (Galaksil 250 × 10 mm, 5 μm) sourced from Galak Chromatography (Wuxi, Jiangsu, China), in addition to a Shim-pack GIST Phenyl reverse-phase column (250 × 10 mm, 5 μm), also produced by Shimadzu. Bioassay activity was assessed using the Synergy HT multidetection microplate reader (BioTek, Winooski, USA) and a microscopy system (Nikon, Tokyo, Japan).

### Extraction

Seagrass adhesive was utilized to attach the sea anemones to tiles, which were subsequently placed in a 0.6 ton tank containing a continuous flow of natural seawater for the cultivation of *Zoanthus Kuroshio* (ZK). The freeze-dried cultured ZK was subjected to exhaustive extraction using a mixture of MeOH and DCM in a 1:1 ratio at room temperature. The resulting extracts were evaporated at reduced pressure, yielding an extract (CR) weighing 21.5 g. The CR extract underwent liquid–liquid partitioning between *n*-hexane and H_2_O to yield two distinct layers. The H_2_O layer was further partitioned with EtOAc to generate two additional layers. Concentration of the Hex, EtOAc, and H_2_O layers under reduced pressure resulted in the Hex fraction (HF, 2.5 g), the EtOAc fraction (EF, 11.5 g), and the H_2_O fraction (WF, 3.4 g).

### Utilization of Ultraperformance Liquid Chromatography-Tandem Mass Spectrometry (UPLC-MS/MS) for the Collection of Fragment Ions

MS^2^ profile was acquired with a Waters SYNAPT G2 LC/Q-TOF system (Waters Corporation, Milford, MA, USA). Prior to MS spectral assessment, chromatography was conducted by utilizing a Waters Acquity UPLC BEH C18 column (Waters, 1.7 μm, 2.1 mm × 100 mm). Acetonitrile (CH_3_CN) (designated as A) and water, both containing 0.1% formic acid, constituted the mobile phases. The gradient elution was as follows: 0.01–7 min, 5–10% A; 7–15 min, 10–50% A; 15–17.5 min, 50–60% A; 17.5–25 min, 60% A; 25–27.5 min, 60–70% A; 27.5–28.5 min, 70–100% A; 28.5–30 min, 100% A. The flow velocity was established at 0.5 mL/min, and the column was kept at 40 °C. The extract (4.0 mg) was diluted in 1 mL of MeOH and processed through a 0.45 μm membrane filter, employing an automated sample injection system programmed for a syringe volume of 5 μL. MS^1^ and MS^2^ data were acquired in the *m*/*z* domain of 100–2000, and MS^2^ scans were performed employing an automatic data-dependent acquisition (DDA) method, which involved gradually increasing the collision energy from 10 to 50 eV to fragment five nontargeted precursor ions. The collected MS profiles were processed applying the Waters MassFragment program (MassLynx4.1, Waters, MA, USA).

### The Global Natural Product Social Molecular Networking (GNPS) Classical Molecular Networking Analysis

The Global Natural Product Social Molecular Networking (GNPS) classical molecular networking analysis was conducted using the GNPS web interface (job ID: 45912123d2d140fc98cd32d9ce0d01ac, from https://gnps.ucsd.edu/). The analysis of the MS/MS spectra included window filtering to capture the top five most intense ion peaks within an ±50 Da range throughout the spectrum. A network was subsequently created, requiring a minimum of four matched peaks, with connections between nodes assessed based on a cosine value greater than 0.70. The resulting molecular network visualization, which featured annotated nodes derived from the isolated MS^2^ fragmentations, was generated and organized using Cytoscape 3.8.2 software (NRNB, USA). Compound annotation was conducted through the integration of data from both the GNPS and Reaxys databases in conjunction with information obtained from relevant published literature.

### Isolation

FCS was performed on Si-60 silica gel for the EF material (11.5 g) utilizing gradual elution at a velocity of 100 mL/min with a mixture of *n*-hexane and EtOAc, changing from a ratio of 1:0 to 0:1 over the course of 100 min, resulting in separation of nine fractions (Fr.K1 to Fr.K9). Fr.K7 (661.6 mg) was subjected to FCS on Si-60 silica gel, employing a series of combinations of *n*-hexane, EtOAc, and MeOH with increasing eluting power (*n*-hexane/EtOAc/MeOH, 100:0:0, 80:20:0, 60:40:0, 40:60:0, 20:80:0, 0:100:0, 0:0:100) at 30 mL/min, resulting in seven subfractions (Fr.K7-1 to Fr.K7-7). Fr.K7-1 (65.7 mg) was purified using an EF–C18-H column with a flow velocity of 2 mL/min and isocratic elution of 59% CH_3_CN in H_2_O, yielding compound **6** (2.76 mg). Fraction K7-4 (226 mg) was purified using the same column, but with a gradient elution (35–57% CH_3_CN/H_2_O in 30 min) at a flow speed of 2 mL/min, leading to two subfractions (Fr.K7-4-1 and Fr.K7-4-2). Fr.K7-4-1 (120.4 mg) was further purified under the similar conditions mentioned above, with a minor modification to use isocratic elution (54% CH_3_CN/H_2_O) instead of gradient elution to isolate compound **7** (55.9 mg), compound **8** (18.0 mg) and compound **1** (1.57 mg). Additional purification was performed on subfractions Fr.K7-4-2 (70.6 mg) and Fr.K7-3 (17.49 mg) utilizing a Shim-pack GIST phenyl reverse phase column (flow rate of 2 mL/min, 49% CH_3_CN/H_2_O), which yielded isolated compound **2** (4.37 mg) and compound **3** (1.86 mg), respectively.

Fr.K8 (1265 mg) was subjected to a chromatographic separation on C18-reversed phase silica gel using FC with a MeOH/H_2_O gradient (1:0 to 0:1) in 90 min, maintaining a flow velocity of 35 mL/min. This process generated separation into six fractions (Fr.K8-1 to Fr.K8-6), with 48.8 mg of Fr. K8-2 was identified as compound **9**. Furthermore, RP-HPLC was utilized to separate fraction Fr.K8-5 into eight subfractions with a gradient elution condition of 54–75% CH_3_CN/H_2_O in 25 min. Fr.K8-5-5 (4.38 mg) and Fr.K8-5-6 (2.65 mg) were further purifiedwith a mobile phase composed of acetonitrile and water (57:43) to generate compounds **4** (1.84 mg) and **5** (0.58 mg).

#### Norzoanbenzaldehyd (**1**)

White amorphous powder; [α]_D_^25^ +42 (*c* 0.7, MeOH); UV λ_max_ (MeOH) 204 (1.51), 212 (1.50), 235 (1.26) nm; IR (ATR) υ_max_ (CHCl_3_) 2924, 2360, 1720, 1674, 1458, 1242 cm^–1^; ESI-HRMS *m*/*z* 476.2444 (calculated for C_29_H_34_NO_5_, 476.2437, [M + H]^+^); ^1^H (600 MHz, C_5_D_5_N) and ^13^C (150 MHz, C_5_D_5_N) NMR chemical shifts are reported in Table 1.

#### Norzoazepanol (**2**)

White amorphous powder; [α]_D_^25^ −17 (*c* 0.7, MeOH); UV λ_max_ (CHCl_3_) 202 (0.78), 233 (1.16) nm; IR (ATR) υ_max_ (CHCl_3_) 3418, 2924, 1705, 1659, 1458, 1381, 1250, 1211, 1034 cm^–1^; ESI-HRMS *m*/*z* 468.3120 (calculated for C_29_H_42_NO_4_, 468.3114, [M + H]^+^); ^1^H (600 MHz, CDCl_3_) and ^13^C (150 MHz, CDCl_3_) NMR chemical shifts are reported in Table 1.

#### 3-Acetoxynorzoanthaminone (**3**)

White amorphous powder; [α]_D_^25^ +81 (*c* 0.7, MeOH); UV λ_max_ (CHCl_3_) 204 (1.60), 233 (2.07) nm; IR (ATR) υ_max_ (CHCl_3_) 2970, 2199, 1721, 1667, 1435, 1373, 1234, 1119, 1026 cm^–1^; ESI-HRMS *m*/*z* 554.2757 (calculated for C_31_H_40_NO_8_, 554.2754, [M + H]^+^); ^1^H (600 MHz, CDCl_3_) and ^13^C (150 MHz, CDCl_3_) NMR chemical shifts are reported in Table 2.

#### 11-Hydroxynorzoanthamide B (**4**)

White amorphous powder; [α]_D_^25^ +27 (*c* 0.7, MeOH); UV λ_max_ (CHCl_3_) 205 (2.81), 234 (3.54) nm ; IR (ATR) υ_max_ (CHCl_3_) 3464, 3433, 3395, 3017, 2955, 2924, 1736, 1705, 1667, 1427, 1389, 1342, 1312, 1242, 1219, 1150, 1103 cm^–1^; ESI-HRMS *m*/*z* 512.2653 (calculated for C_29_H_38_NO_7_, 512.2648, [M + H]^+^); ^1^H (600 MHz, CDCl_3_) and ^13^C (150 MHz, CDCl_3_) NMR chemical shifts are reported in Table 2.

#### 11-Hydroxyzoanthamide B (**5**)

White amorphous powder; [α]_D_^25^ +64 (*c* 0.7, MeOH); UV λ_max_ (CHCl_3_) 205 (2.54), 235 (2.51) nm; IR (ATR) υ_max_ (CHCl_3_) 3379, 3341, 3202, 2924, 2361, 1705, 1659, 1458, 1381, 1219, 1150 cm^–1^; ESI-HRMS *m*/*z* 526.2818 (calculated for C_30_H_40_NO_7_, 526.2805, [M + H]^+^); ^1^H (800 MHz, CDCl_3_) and ^13^C (200 MHz, CDCl_3_) NMR chemical shifts are reported in Table 2.

### NMR and ECD Calculations

Compound **2** was analyzed by generating possible conformers within a 3.5 kcal/mol energy window for each configuration utilizing the GMMX package from GaussView 6.1 (Gaussian Inc., Wallingford, CT, USA). All obtained conformers were subsequently analyzed for geometry optimization and frequency using DFT at the B3LYP/6-31G­(d,p) level in the solvent phase (CHCl_3_), applying Gaussian 16 software. All conformers used for property calculations in this study were confirmed as stable points on the potential energy surface (PES) by the nonexistence of imaginary frequencies. The Gibbs free energy of each conformer was then employed to calculate the Boltzmann distribution, and conformers with a Boltzmann population below 1% were excluded from further analysis. Subsequently, the remaining conformers were subjected to GIAO–DFT calculations for NMR under the mpw1pw91/6-311+G­(d,p) level with IEFPCM-CHCl_3_, following DP4+ methodology. The unscaled chemical shifts (δ_u_) were calculated by utilizing tetramethylsilane (TMS) as a reference standard, following the equation δ_u_ = σ_0_ – σ_*x*_. In this context, σ_*x*_ represents the Boltzmann-averaged shielding tensor derived from all significantly populated conformations, while σ_0_ denotes the shielding tensor of TMS calculated at the same theoretical level as σ_*x*_. The theoretical NMR predictions were subsequently averaged, taking into account the relative proportions of each conformer and were analyzed using an Excel spreadsheet in conjunction with the DP4+ method.^19^

Excitation energies of the first 30 energy levels for ECD calculations were determined using the DFT/B3LYP/6-311G­(d,p) level with the aforementioned solvation model, as implemented in Gaussian 16 software. The calculated ECD spectra of the conformers were integrated by weighting according to their Boltzmann distribution rates, using SpecDis 1.71 software with a σ value of 0.30 eV. The resulting spectra were subsequently compared with the experimental data, where the CD values are reported in millidegrees (millimeters) across the 200–500 nm wavelength range, with a total of 301 data points collected.

### Cell Viability Assay

The viability of the cells was examined using the MTT assay as previously detailed in the literature.^20^ In brief, MG-63 cells were seeded in 96-well culture plates at a concentration of 4 × 10^3^ cells/well. Following a 24 h period, freshly formulated medium incorporating the test samples was used to replace the culture medium. After a 72 h of incubation, the medium was removed, combining the addition of MTT for for 4 h, after which it was substituted with 100 μL of DMSO, and the wells were gently mixed to dissolve the resulting dark-blue precipitate. A microplate reader (Bio Tek Instruments, Winooski, VT, USA) was used to obtain optical density (OD) measurements at 600 nm, following the manufacturer’s guidelines.

### ALP Activity Assay

Using a colorimetric assay with *p*-nitrophenyl phosphate as the substrate, the assessment of ALP activity was conducted in MG-63 cells.^21^ Initially, the protocols for culturing MG-63 cells mirrored those employed for the cell viability assessment; however, after a 72 h incubation, the medium was eliminated, and the cells were rinsed with 200 μL of normal saline before being treated with 0.1% Triton X-100 lysis buffer. Using a BCA assay, the quantification of protein levels in the supernatant was conducted, with the supernatants mixed with BCA reagent for 30 min at room temperature, and absorbance was gauged at 560 nm. Subsequently, ALP activity in the supernatants was assessed by incubating them for 1 h at room temperature in a buffer solution composed of 0.2 M Tris-HCl (pH 9.5) supplemented with 0.1% Triton X-100, 1 mM MgCl_2_, and 6 mM *p*-nitrophenyl phosphate. Through the addition of 0.5 M NaOH, the reaction was halted, and absorbance was evaluated at 405 nm, with ALP function subsequently adjusted to the protein levels of each sample.

### Mineralized Matrix Formation Assay

The mineralization of MG-63 cells was assessed by quantifying calcium deposits through alizarin red-S staining, following the initial plating of the cells in multiwell plates at a density of 2 × 10^4^ cells per well.^22^ After 3 days, mineralization medium, consisting of a mixture of 50 μg/mL ascorbic acid, 10 mM β-glycerophosphate, and the test samples, was used to replace the growth medium. The cells were incubated for 12 days and subsequently treated with 70% ethanol, and calcium deposits were stained for 1 h during incubation at 37 °C using a 40 mM alizarin red-S solution (pH 4.2). A mixture of 10% (w/v) cetylpyridinium chloride in 10 mM sodium phosphate (pH 7.0) was used to elute the bound stain, followed by measurement of the absorbance at 550 nm.

### Statistical Analysis

The data are presented as the mean ± the standard deviation (SD). One-way analysis of variance (ANOVA) followed by the Student–Newman–Keuls test was used to evaluate significant differences (*p* < 0.05) among the corresponding mean values. The term “*n*” denotes the number of biological replicates.

## Supplementary Material





## Data Availability

The NMR data for the following compounds have been deposited in the Natural Products Magnetic Resonance Database (NP-MRD; https://np-mrd.org): norzoabenzaldehyde, norzoazepanol, 3-acetoxynorzoanthaminone, 11-hydroxynorzoanthamide B, and 11-hydroxyzoanthamide B.

## Notes

The annotated zoanthamine alkaloids originating from the extract of ZK; IR, HRESIMS, UV, and 1D/2D NMR spectra for **1**–**5**; and NMR and ECD calculations of compound **2** (PDF)
Raw NMR data (ZIP)
