# Supplementary material for: Zoanthamine-Type Alkaloids Derived from Cultured Zoanthus kuroshio with Therapeutic Potential Against Osteoporosis
Source: J Nat Prod. 2025 Jun 10;88(9):2053–64. doi: 10.1021/acs.jnatprod.5c00457 (PMC12481557; doi:10.1021/acs.jnatprod.5c00457)

Supporting Information

Zoanthamine-Type Alkaloids Derived from Cultured *Zoanthus kuroshio* with Therapeutic Potential Against Osteoporosis

Ngoc-Thac Pham[1] · Bo-Rong Peng[1,2,3,4] · Huong-Giang Le[1] · You-Song Cheng[5] · Yun-Shiuan Chen[1] · Thanh Hao Huynh[3] · Lo-Yun Chen[1] · Le Anh Tuan Nguyen[6] · Dang T. Nguyen[7] · Yu-Chia Chang[3,4] · Jui-Hsin Su[8,9] · Mohamed El-Shazly[10] · Mei-Hsien Lee[1,2,11] · Kuei-Hung Lai[1,2,12,*]

[1] *PhD Program in Clinical Drug Development of Herbal Medicine, College of Pharmacy, Taipei Medical University, Taipei 110301, Taiwan*

[2] *Graduate Institute of Pharmacognosy, College of Pharmacy, Taipei Medical University, Taipei 110301, Taiwan*

[3] *Graduate Institute of Healthy Industry Technology, Center for Drug Research and Development, College of Human Ecology, Chang Gung University of Science and Technology, Taoyuan 333324, Taiwan*

[4] *Department of Cosmetic Science, Chang Gung University of Science and Technology, Taoyuan City 33303, Taiwan*

[5] *Instrumentation Resource Center at National Yang Ming Chiao Tung University*

[6] *Faculty of Pharmacy, Lac Hong University, Bien Hoa city, Dongnai, Vietnam*

[7] *Faculty of Applied Sciences, Ton Duc Thang University, Ho Chi Minh City, Vietnam*

[8] *National Museum of Marine Biology and Aquarium, Pingtung 944401, Taiwan*

[9] *Department of Marine Biotechnology and Resources, National Sun Yat-sen University, Kaohsiung 804201, Taiwan*

[10] *Department of Pharmacognosy, Faculty of Pharmacy, Ain-Shams University, Organization of African Unity Street, Abassia, Cairo 11566, Egypt*

[11] *Center for Reproductive Medicine and Sciences, Taipei Medical University Hospital, Taipei 110301, Taiwan*

[12] *Traditional Herbal Medicine Research Center, Taipei Medical University Hospital, Taipei 110301, Taiwan*

* Corresponding authors. E-mail addresses: kueihunglai@tmu.edu.tw (K.-H. Lai); TEL: +886-2-2736-1661 ext. 6157 (K.-H. Lai)

Authors’ E-mail addresses: d339112005@tmu.edu.tw (N.-T. Pham), peng_br@tmu.edu.tw (B.-R. Peng), d339111003@tmu.edu.tw (H.-G. Le), gnoosuy@nycu.edu.tw (Y.-S Cheng), d339112006@tmu.edu.tw (Y.-S. Chen), a03881@tmu.edu.tw (T.H. Huynh), d339112001@tmu.edu.tw (L.-Y. Chen), tuannguyen@lhu.edu.vn (L.A.T. Nguyen), nguyenthuongdang@tdtu.edu.vn (D.T. Nguyen), ycchang03@mail.cgust.edu.tw (Y.-C. Chang), x2219@nmmba.gov.tw (J.-H. Su), mohamed.elshazly@pharma.asu.edu.eg (M. El-Shazly), lmh@tmu.edu.tw (M.-H. Lee) , kueihunglai@tmu.edu.tw (K.-H. Lai)

Table legend

Table S1. The annotated zoanthamine alkaloids originating from the extract of ZK.

Table S2. NMR data for norzoabenzaldehyde (**1**) in $CDCl_3$.

Table S3. NMR data for norzoazepanol (**2**) in $CDCl_3$.

Table S4. NMR data for 3-acetoxynorzoanthaminone (**3**) in $CDCl_3$.

Table S5. NMR data for 11-hydroxynorzoanthamide B (**4**) in $CDCl_3$.

Table S6. NMR data for 11-hydroxyzoanthamide B (**5**) in $CDCl_3$.

Table S7. ^{1}H and ^{13}C NMR data in ppm for norzoanthaminone (**6**) and 3-hydroxynorzoanthamine (**9**) in $CDCl_3$.

Table S8. ^{1}H and ^{13}C NMR data in ppm for 3-acetoxynorzoanthamine (**7**) and 3-acetoxyzoanthamine (**8**) in $CDCl_3$.

Table S9. Gibbs free energy of the conformers of compound **2** and the Boltzmann distribution of each conformer.

Table S10. The cartesian coordinates of the dominant conformers for 2*S*-**2** and 2*R*-**2.**

Figure legend

Figure S1. ^{1}H-NMR spectrum of fraction EF at 600 MHz in C_5D_5N

Figure S2. ^{1}H-NMR spectrum of norzoabenzaldehyde (**1**) at 600 MHz in C_5D_5N

Figure S3. ^{1}H-NMR spectrum of norzoabenzaldehyde (6-11 ppm) at 600 MHz in C_5D_5N

Figure S4. ^{1}H-NMR spectrum of norzoabenzaldehyde (3-4 ppm) at 600 MHz in C_5D_5N

Figure S5. ^{1}H-NMR spectrum of norzoabenzaldehyde (1-3 ppm) at 600 MHz in C_5D_5N

Figure S6. ^{13}C-NMR spectrum of norzoabenzaldehyde (**1**) at 150 MHz in C_5D_5N

Figure S7. ^{13}C-NMR spectrum of norzoabenzaldehyde (115-210 ppm) at 150 MHz in C_5D_5N

Figure S8. ^{13}C-NMR spectrum of norzoabenzaldehyde (15-105 ppm) at 150 MHz in C_5D_5N

Figure S9. COSY spectrum of norzoabenzaldehyde (**1**) at 600 MHz in C_5D_5N

Figure S10. COSY spectrum of norzoabenzaldehyde (2.25-2.66 ppm) at 600 MHz in C_5D_5N

Figure S11. COSY spectrum of norzoabenzaldehyde (2.65-2.97 ppm) at 600 MHz in C_5D_5N

Figure S12. HSQC spectrum of norzoabenzaldehyde (**1**) at 600 MHz and 150 MHz in C_5D_5N

Figure S13. HSQC spectrum of norzoabenzaldehyde (9.75-10.40 ppm) at 600 and 150 MHz in C_5D_5N

Figure S14. HSQC spectrum of norzoabenzaldehyde (6.0-7.6 ppm) at 600 and 150 MHz in C_5D_5N

Figure S15. HSQC spectrum of norzoabenzaldehyde (0.9-4.0 ppm) at 600 and 150 MHz in C_5D_5N

Figure S16. HMBC spectrum of norzoabenzaldehyde (**1**) at 600 and 150 MHz in C_5D_5N

Figure S17. HMBC spectrum of norzoabenzaldehyde (10.10-10.50 ppm) at 600 and 150 MHz in C_5D_5N

Figure S18. HMBC spectrum of norzoabenzaldehyde (6.85-7.95 ppm) at 600 and 150 MHz in C_5D_5N

Figure S19. HMBC spectrum of norzoabenzaldehyde (2.40-4.20 ppm) at 600 and 150 MHz in C_5D_5N

Figure S20. HMBC spectrum of norzoabenzaldehyde (0.90-2.30 ppm) at 600 and 150 MHz in C_5D_5N

Figure S21. NOESY spectrum of norzoabenzaldehyde (**1**) at 600 MHz in C_5D_5N

Figure S22. NOESY spectrum of norzoabenzaldehyde (7.82-8.00 ppm) at 600 MHz in C_5D_5N

Figure S23. NOESY spectrum of norzoabenzaldehyde (3.02-3.22 ppm) at 600 MHz in C_5D_5N

Figure S24. NOESY spectrum of norzoabenzaldehyde (0.98-1.24 ppm) at 600 MHz in C_5D_5N

Figure S25. MS spectrum of norzoabenzaldehyde (**1**)

Figure S26. UV spectrum of norzoabenzaldehyde (**1**)

Figure S27. IR (ATR) spectrum of norzoabenzaldehyde (**1**)

Figure S28. ^{1}H-NMR spectrum of norzoazepanol (**2**) at 600 MHz in $CDCl_3$

Figure S29. ^{1}H-NMR spectrum of norzoazepanol (3.0-6.1 ppm) at 600 MHz in $CDCl_3$

Figure S30. ^{1}H-NMR spectrum of norzoazepanol (0.8-2.9 ppm) at 600 MHz in $CDCl_3$

Figure S31. ^{13}C-NMR spectrum of norzoazepanol (**2**) at 150 MHz in $CDCl_3$

Figure S32. ^{13}C-NMR spectrum of norzoazepanol (120-220 ppm) at 150 MHz in $CDCl_3$

Figure S33. ^{13}C-NMR spectrum of norzoazepanol (20-74 ppm) at 150 MHz in $CDCl_3$

Figure S34. COSY spectrum of norzoazepanol (**2**) at 600 MHz in $CDCl_3$

Figure S35. COSY spectrum of norzoazepanol (2.9-4.3 ppm) at 600 MHz in $CDCl_3$

Figure S36. COSY spectrum of norzoazepanol (0.9-2.8 ppm) at 600 MHz in $CDCl_3$

Figure S37. HSQC spectrum of norzoazepanol (**2**) at 600 and 150 MHz in $CDCl_3$

Figure S38. HSQC spectrum of norzoazepanol (3.80-6.10 ppm) at 600 and 150 MHz in $CDCl_3$

Figure S39. HSQC spectrum of norzoazepanol (0.80-3.50 ppm) at 600 and 150 MHz in $CDCl_3$

Figure S40. HMBC spectrum of norzoazepanol (**2**) at 600 and 150 MHz in $CDCl_3$

Figure S41. HMBC spectrum of norzoazepanol (5.78-6.03 ppm) at 600 and 150 MHz in $CDCl_3$

Figure S42. HMBC spectrum of norzoazepanol (2.0-3.2 ppm) at 600 and 150 MHz in $CDCl_3$

Figure S43. HMBC spectrum of norzoazepanol (0.85-1.45 ppm) at 600 and 150 MHz in $CDCl_3$

Figure S44. NOESY spectrum of norzoazepanol (**2**) at 600 MHz in $CDCl_3$

Figure S45. NOESY spectrum of norzoazepanol (2.03-2.31 ppm) at 600 MHz in $CDCl_3$

Figure S46. NOESY spectrum of norzoazepanol (1.4-1.56 ppm) at 600 MHz in $CDCl_3$

Figure S47. NOESY spectrum of norzoazepanol (1.4-1.56 ppm) at 600 MHz in $CDCl_3$

Figure S48. NOESY spectrum of norzoazepanol (0.85-1.06 ppm) at 600 MHz in $CDCl_3$

Figure S49. NOESY spectrum of norzoazepanol (2.46-2.73 ppm) at 600 MHz in $CDCl_3$

Figure S50. MS spectrum of norzoazepanol (**2**)

Figure S51. UV spectrum of norzoazepanol (**2**)

Figure S52. IR (ATR) spectrum of norzoazepanol (**2**)

Figure S53. DP4+ results obtained using experimental data of compound **2** *versus* isomers 2*S*-**2** (isomer 1) and 2*R*-**2** (isomer 2)

Figure S54. The cartesian coordinates of the dominant conformers for 2*S*-**2** and 2*R*-**2.**

Figure S55. ^{1}H-NMR spectrum of 3-acetoxynorzoanthaminone (**3**) at 600 MHz in $CDCl_3$

Figure S56. ^{1}H-NMR spectrum of 3-acetoxynorzoanthaminone (2.8-6.2 ppm) at 600 MHz in $CDCl_3$

Figure S57. ^{1}H-NMR spectrum of 3-acetoxynorzoanthaminone (0.8-2.9 ppm) at 600 MHz in $CDCl_3$

Figure S58. ^{13}C-NMR spectrum of 3-acetoxynorzoanthaminone (**3**) at 150 MHz in $CDCl_3$

Figure S59. ^{13}C-NMR spectrum of 3-acetoxynorzoanthaminone (90-215 ppm) at 150 MHz in $CDCl_3$

Figure S60. ^{13}C-NMR spectrum of 3-acetoxynorzoanthaminone (15-80 ppm) at 150 MHz in $CDCl_3$

Figure S61. COSY spectrum of 3-acetoxynorzoanthaminone (**3**) at 600 MHz in $CDCl_3$

Figure S62. COSY spectrum of 3-acetoxynorzoanthaminone (0.8-4.8 ppm) at 600 MHz in $CDCl_3$

Figure S63. HSQC spectrum of 3-acetoxynorzoanthaminone (**3**) at 600 and 150 MHz in $CDCl_3$

Figure S64. HSQC spectrum of 3-acetoxynorzoanthaminone (4.4-6.1 ppm) at 600 and 150 MHz in $CDCl_3$

Figure S65. HSQC spectrum of 3-acetoxynorzoanthaminone (0.8-4.2 ppm) at 600 and 150 MHz in $CDCl_3$

Figure S66. HMBC spectrum of 3-acetoxynorzoanthaminone (**3**) at 600 and 150 MHz in $CDCl_3$

Figure S67. HMBC spectrum of 3-acetoxynorzoanthaminone (3.90-4.28 ppm) at 600 and 150 MHz in $CDCl_3$

Figure S68. HMBC spectrum of 3-acetoxynorzoanthaminone **(**0.7-3.2 ppm) at 600 and 150 MHz in $CDCl_3$

Figure S69. NOESY spectrum of 3-acetoxynorzoanthaminone (**3**) at 600 MHz in $CDCl_3$

Figure S70. NOESY spectrum of 3-acetoxynorzoanthaminone (2.65-2.77 ppm) at 600 MHz in $CDCl_3$

Figure S71. NOESY spectrum of 3-acetoxynorzoanthaminone (2.65-2.77 ppm) at 600 MHz in $CDCl_3$

Figure S72. NOESY spectrum of 3-acetoxynorzoanthaminone (2.953.06 ppm) at 600 MHz in $CDCl_3$

Figure S73. MS spectrum of 3-acetoxynorzoanthaminone (**3**)

Figure S74. UV spectrum of 3-acetoxynorzoanthaminone (**3**)

Figure S75. IR (ATR) spectrum of 3-acetoxynorzoanthaminone (**3**)

Figure S76. ^{1}H-NMR spectrum of 11-hydroxynorzoanthamide B (**4**) at 600 MHz in $CDCl_3$

Figure S77. ^{1}H-NMR spectrum of 11-hydroxynorzoanthamide B (3.60-6.30 ppm) at 600 MHz in $CDCl_3$

Figure S78. ^{1}H-NMR spectrum of 11-hydroxynorzoanthamide B (0.80-3.20 ppm) at 600 MHz in $CDCl_3$

Figure S79. ^{13}C-NMR spectrum of 11-hydroxynorzoanthamide B (**4**) at 150 MHz in $CDCl_3$

Figure S80. ^{13}C-NMR spectrum of 11-hydroxynorzoanthamide B (105-225 ppm) at 150 MHz in $CDCl_3$

Figure S81. ^{1}H-NMR spectrum of 11-hydroxynorzoanthamide B (15-100 ppm) at 600 MHz in $CDCl_3$

Figure S82. COSY spectrum of 11-hydroxynorzoanthamide B (**4**) at 600 MHz in $CDCl_3$

Figure S83. COSY spectrum of 11-hydroxynorzoanthamide B (3.70-4.90 ppm) at 600 MHz in $CDCl_3$

Figure S84. COSY spectrum of 11-hydroxynorzoanthamide B (3.70-4.90 ppm) at 600 MHz in $CDCl_3$

Figure S85. HSQC spectrum of 11-hydroxynorzoanthamide B (**4**) at 600 and 150 MHz in $CDCl_3$

Figure S86. HSQC spectrum of 11-hydroxynorzoanthamide B (4.2-6.1 ppm) at 600 and 150 MHz in $CDCl_3$

Figure S87. HSQC spectrum of 11-hydroxynorzoanthamide B (0.9-4.0 ppm) at 600 and 150 MHz in $CDCl_3$

Figure S88. HMBC spectrum of 11-hydroxynorzoanthamide B (**4**) at 600 and 150 MHz in $CDCl_3$

Figure S89. HMBC spectrum of 11-hydroxynorzoanthamide B (4.3-6.0 ppm) at 600 and 150 MHz in $CDCl_3$

Figure S90. HMBC spectrum of 11-hydroxynorzoanthamide B (1.0-4.0 ppm) at 600 and 150 MHz in $CDCl_3$

Figure S91. NOESY spectrum of 11-hydroxynorzoanthamide B (**4**) at 600 MHz in $CDCl_3$

Figure S92. NOESY spectrum of 11-hydroxynorzoanthamide B (4.7-4.8 ppm) at 600 MHz in $CDCl_3$

Figure S93. NOESY spectrum of 11-hydroxynorzoanthamide B (3.0-3.2 ppm) at 600 MHz in $CDCl_3$

Figure S94. NOESY spectrum of 11-hydroxynorzoanthamide B (1.0-1.17 ppm) at 600 MHz in $CDCl_3$

Figure S95. MS spectrum of 11-hydroxynorzoanthamide B (**4**)

Figure S96. UV spectrum of 11-hydroxynorzoanthamide B (**4**)

Figure S97. IR (ATR) spectrum of 11-hydroxynorzoanthamide B (**4**)

Figure S98. ^{1}H-NMR spectrum of 11-hydroxyzoanthamide B (**5**) at 800 MHz in $CDCl_3$

Figure S99. ^{1}H-NMR spectrum of 11-hydroxyzoanthamide B (3.0-6.2 ppm) at 800 MHz in $CDCl_3$

Figure S100. ^{1}H-NMR spectrum of 11-hydroxyzoanthamide B (1.0-2.7 ppm) at 800 MHz in $CDCl_3$

Figure S101. ^{13}C-NMR spectrum of 11-hydroxyzoanthamide B (**5**) at 200 MHz in $CDCl_3$

Figure S102. ^{13}C-NMR spectrum of 11-hydroxyzoanthamide B (95-220 ppm) at 200 MHz in $CDCl_3$

Figure S103. ^{13}C-NMR spectrum of 11-hydroxyzoanthamide B (10-80 ppm) at 200 MHz in $CDCl_3$

Figure S104. COSY spectrum of 11-hydroxyzoanthamide B (**5**) at 800 MHz in $CDCl_3$

Figure S105. COSY spectrum of 11-hydroxyzoanthamide B (2.9-4.9 ppm) at 800 MHz in $CDCl_3$

Figure S106. COSY spectrum of 11-hydroxyzoanthamide B (0.6-2.6 ppm) at 800 MHz in $CDCl_3$

Figure S107. HSQC spectrum of 11-hydroxyzoanthamide B (**5**) at 800 and 200 MHz in $CDCl_3$

Figure S108. HSQC spectrum of 11-hydroxyzoanthamide B (4.3-5.9 ppm) at 800 and 200 MHz in $CDCl_3$

Figure S109. HSQC spectrum of 11-hydroxyzoanthamide B (4.3-5.9 ppm) at 800 and 200 MHz in $CDCl_3$

Figure S110. HMBC spectrum of 11-hydroxyzoanthamide B (**5**) at 800 and 200 MHz in $CDCl_3$

Figure S111. HMBC spectrum of 11-hydroxyzoanthamide B (4.5-5.9 ppm) at 800 and 200 MHz in $CDCl_3$

Figure S112. HMBC spectrum of 11-hydroxyzoanthamide B (1.8-3.8 ppm) at 800 and 200 MHz in $CDCl_3$

Figure S113. HMBC spectrum of 11-hydroxyzoanthamide B (0.96-1.46 ppm) at 800 and 200 MHz in $CDCl_3$

Figure S114. NOESY spectrum of 11-hydroxyzoanthamide B (**5**) at 800 MHz in $CDCl_3$

Figure S115. NOESY spectrum of 11-hydroxyzoanthamide B (4.68-4.84 ppm) at 800 MHz in $CDCl_3$

Figure S116. NOESY spectrum of 11-hydroxyzoanthamide B (3.38-3.48 ppm) at 800 MHz in $CDCl_3$

Figure S117. NOESY spectrum of 11-hydroxyzoanthamide B (2.54-2.63 ppm) at 800 MHz in $CDCl_3$

Figure S118. MS spectrum of 11-hydroxyzoanthamide B (**5**)

Figure S119. UV spectrum of 11-hydroxyzoanthamide B (**5**)

Figure S120. IR (ATR) spectrum of 11-hydroxyzoanthamide B (**5**)

Figure S121. ^{1}H-NMR spectrum of norzoanthaminone (**6**)

Figure S122. ^{13}C-NMR spectrum of norzoanthaminone (**6**)

Figure S123. ^{1}H-NMR spectrum of 3-acetoxynorzoanthamine (**7**) at 600 MHz in $CDCl_3$

Figure S124. ^{13}C-NMR spectrum of 3-acetoxynorzoanthamine (**7**) at 150 MHz in $CDCl_3$

Figure S125. ^{1}H-NMR spectrum of 3-acetoxyzoanthamine (**8**) at 600 MHz in $CDCl_3$

Figure S126. ^{13}C-NMR spectrum of 3-acetoxyzoanthamine (**8**) at 150 MHz in $CDCl_3$

Figure S127. ^{1}H-NMR spectrum of 3-hydroxynorzoanthamine (**9**) in $CDCl_3$ at 600 MHz in $CDCl_3$

Figure S128. ^{13}C-NMR spectrum of 3-hydroxynorzoanthamine (**9**) at 150 MHz in $CDCl_3$

Figure S129. ^{1}H-NMR spectrum of norzoabenzaldehyde (**1**) at 600 MHz in C_5D_5N after repurification

Figure S130. ^{13}C-NMR spectrum of norzoabenzaldehyde (**1**) at 150 MHz in C_5D_5N after repurification

Table S1. The annotated zoanthamine alkaloids originating from the extract of ZK

No	t_R (min)	Compound name	Ion adduct	Precursor ion (m/z)	Product ion (m/z)	Molecular formula (error in ppm)	Reference
**Known compounds**							
1	0.75	15-hydroxynorzoanthamine	$[M+H]^+$	500.297	208.1687, 426.2616	$C_{29}H_{41}NO_6$ (-8.00)	[1]
2	1.15	oxyzoanthamine	$[M+H]^+$	512.301	208.1688, 482.2892	$C_{30}H_{41}NO_6$ (0.00)	GNPS libraries
3	-6.02	11-Hydroxynorzoanthamine	$[M+H]^+$	498.283	208.1688, 424.2768	$C_{29}H_{39}NO_6$ (-6.02)	GNPS libraries
4	1.47	3-hydroxynorzoanthamine	$[M+H]^+$	498.283	208.1688, 424.2768	$C_{29}H_{39}NO_6$ (-6.02)	GNPS libraries
5	1.57	zoanthamine	$[M+H]^+$	496.303	208.1692, 271.1322, 340.2263, 422.2683	$C_{30}H_{41}NO_5$ (-6.04)	GNPS libraries
6	1.61	kuroshine B	$[M+H]^+$	540.292	206.1534, 266.1745, 466.2577	$C_{31}H_{41}NO_7$ (-7.40)	GNPS libraries
7	1.95	3-acetoxyzoanthamine	$[M+H]^+$	554.310	208.1694, 266.1746, 482.2902	$C_{32}H_{43}NO_7$	[2]

						(-3.61)	
8	1.99	22-epi-28-deoxyzoanthenamine	$[M+HCOO]^+$	538.278	208.1681, 239.1058, 408.2473	$C_{30}H_{39}NO_5$ (-3.72)	GNPS libraries
9	2.29	11β-chloro-11-deoxykuroshine A	$[M+H]^+$	544.252	396.1792, 508.2327, 526.2441	$C_{30}H_{38}ClNO_6$ (9.19)	[3]
10	2.31	zoanthenol	$[M+H]^+$	494.290	208.1688, 420.2522	$C_{30}H_{39}NO_5$ (-2.02)	[4]
11	2.31	28-deoxyzoanthenamine	$[M+H]^+$	494.293	208.1691, 269.1162, 420.2527	$C_{30}H_{39}NO_5$ (4.05)	GNPS libraries
12	2.59	norzoanthamine	$[M+H]^+$	482.290	208.1687, 408.2896	$C_{29}H_{39}NO_5$ (-2.07)	[5]
13	2.79	kuroshine C	$[M+H]^+$	542.274	208.1334, 271.1300, 478.2619, 524.2658	$C_{30}H_{39}NO_8$ (-1.84)	GNPS libraries
14	2.99	3-acetoxynorzoanthamine	$[M+H]^+$	540.295	246.0770, 458.1970, 494.2150, 522.2099	$C_{31}H_{41}NO_7$ (-1.85)	[2]
15	2.99	18-epi-kuroshine E	$[M+H]^+$	524.265	434.2329, 464.2077, 480.2372, 506.2179	$C_{30}H_{37}NO_7$ (0.00)	[3]
16	3.04	norzoanthaminone	$[M+H]^+$	496.268	208.1676, 323.1627, 450.2263	$C_{29}H_{37}NO_6$	[5]

						(-4.03)	
17	3.04	kuroshine D	$[M+HCOO]^+$	586.263	266.1384, 476.2403, 518.2510, 568.2563	$C_{30}H_{39}NO_8$ (-3.41)	[3]
18	3.14	11-hydroxyzoanthamine	$[M+HCOO]^+$	556.290	240.1234, 271.1327, 464.2388, 524.2608	$C_{30}H_{41}NO_6$ (-1.80)	GNPS libraries
19	3.23	28-deoxyzoanthenamide	$[M+H]^+$	496.304	208.1669, 271.1309	$C_{30}H_{41}NO_5$ (-4.03)	[3]
20	3.25	kuroshine E	$[M+H]^+$	524.264	208.1354, 432.2151, 478.2200, 506.2542	$C_{30}H_{37}NO_7$ (-1.91)	[3]
21	3.29	kuroshine F	$[M+H]^+$	540.258	266.1731, 322.1784, 434.1964, 486.1892	$C_{30}H_{37}NO_8$ (-3.70)	GNPS libraries
22	3.39	7α-hydroxykuroshine E	$[M+H]^+$	540.259	254.1558, 300.1580, 382.1644, 442.1879, 494.2521	$C_{30}H_{37}NO_8$ (-1.85)	[3]
23	3.40	kuroshine G	$[M+HCOO]^+$	584.247	264.1225, 436.2087, 489.2292, 524.2274	$C_{30}H_{37}NO_8$ (-5.13)	GNPS libraries
24	3.72	2-hydroxy-11-ketonorzoanthamide B	$[M+H]^+$	526.242	420.2158, 480.2360	$C_{29}H_{35}NO_8$ (-3.80)	[1]
25	4.09	3β-hydroxyzoanthenamide	$[M+H]^+$	526.280	210.1489, 317.1383, 450.2652	$C_{30}H_{39}NO_7$	[3]

						(0.00)	
26	4.18	7α-hydroxyzoanthenamide	$[M+HCOO]^+$	570.268	464.2424, 510.2497	$C_{30}H_{39}NO_7$ (-3.51)	GNPS libraries
27	4.36	norzoanthamide B	$[M+H]^+$	496.268	222.1475, 354.2062, 450.2646	$C_{29}H_{37}NO_6$ (-4.03)	[1]
28	4.76	zoanthenamine	$[M+H]^+$	510.284	352.1889, 464.2401, 482.2530, 492.2723	$C_{30}H_{39}NO_6$ (-3.92)	[3]
29	4.81	kuroshine A	$[M+H]^+$	526.278	210.1482, 317.1371, 410.1966, 508.2305	$C_{30}H_{39}NO_7$ (-3.80)	[3]
30	4.92	zoanthamide	$[M+H]^+$	524.263	408.2149, 478.2572	$C_{30}H_{37}NO_7$ (-3.81)	[5]
**New compounds**							
1	8.09	norzoanbenzaldehyd	$[M+H]^+$	476.242	135.0797, 236.1436, 271.1319, 416.2204	$C_{29}H_{33}NO_5$ (-4.20)	
2	4.98	norzoazepanol	$[M+H]^+$	468.310	110.0959, 128.1057, 173.0955	$C_{29}H_{41}NO_4$ (-2.14)	

3	4.56	3-acetoxynorzoanthaminone	$[M+H]^+$	554.274	121.0631, 287.1266, 494.2558, 524.3062, 536.1881	$C_{31}H_{39}NO_8$ (-1.80)
4	3.12	11-hydroxynorzoanthamide B	$[M+H]^+$	512.263	121.0646, 210.1488, 364.2111	$C_{29}H_{37}NO_7$ (-3.90)
5	6.84	11-hydroxyzoanthamide B	$[M+H]^+$	526.279	104.1067, 184.0732, 239.1038	$C_{30}H_{39}NO_7$ (-1.90)

Table S2. NMR data for norzoabenzaldehyde (**1**) in $CDCl_3$

Position	δ_H, mult (*J* inHz)	δ_C	COSY	HMBC	NOESY
1	10.28, s	194.3, CH		C-2, C-3, C-4, C-7	
2		141.6, C			
3	7.15, s	127.0, CH		C-1, C-5, C-7, C-30	
4		137.6, C			
5	6.97, s	121.3, CH		C-7, C-3, C-30	
6		136.7, C			
7		118.3, C			
8	3.64, d (17.88)<br>3.29, d (17.76)	28.4, CH_2		C-29, C-9, C-10, C-22, C-2, C-7, C-6	
9		38.0, C			
10		93.6, C			
11	2.40, d (14.34)<br>2.27, d (14.40)	43.7, CH_2		C-28, C-13, C-10<br>C-28, C-9, C-21, C-10	
12		40.1, C			
13	2.44, m	53.1, CH	H-14, H-18	C-28, C-14, C-21	
14	2.11, m	31.9, CH_2	H-13	C-15, C-16	
15		161.1, C			
16	6.04, s	126.0, CH		C-27, C-14, C-18	
17		198.8, C			
18	2.74, m	46.7, CH	H-19	C-17, C-13	H-28
19	2.84, m	43.4, CH_2	H-18	C-20, C-18, C-13, C-17	
20		210.2, C			
21	3.13, s	59.9, CH		C-20, C-13, C-22, C-12, C-9	H-29, H-25, H-13
22		37.0, C			
23	3.96, d (20.34)<br>2.91, d (20.34)	38.2, CH_2		C-24, C-9, C-21<br>C-24, C-21, C-9, C-25, C-22	
24		172.5, C			
25	1.26, s	21.3, CH_3		C-21, C-23, C-9	H-21, H-29
26					
27	1.82, s	24.3, CH_3		C-14, C-16, C-15	
28	1.10, s	17.6, CH_3		C-11, C-13, C-21, C-12	H-18
29	1.19, s	18.1, CH_3		C-8, C-9, C-10, C-22	
30	2.21, s	21.1, CH_3		C-3, C-5, C-4	
NH	7.91, s			C-9, C-11, C-5, C-7	H-5, H-29

Table S3. NMR data for norzoazepanol (**2**) in $CDCl_3$

Position	δ_H, mult (*J* inHz)	δ_C	COSY	HMBC	NOESY
1	3.17, m<br>2.17, m	62.1, CH_2	H-2		
2	3.95, m	65.8, CH	H-3, H-1		
3	2.07, m<br>1.02, m	42.9, CH_2	H-4, H-2		
4	1.62, m	25.0, CH	H-5, H-3, H-30		
5	1.67, m	48.5, CH_2	H-6, H-4		
6	2.63, m	69.2, CH	H-7, H-5		H-7
7	2.22, m	54.9, CH	H-8, H-6		H-6
8	2.17 (α), m<br>1.47 (β), m	39.1, CH_2	H-7	C-22, C-7, C-24	
9		36.4, C			
10	2.41, m	69.6, CH	H-11		H-29
11	2.13, m<br>1.80, m	34.9, CH_2	H-10	C-13	
12		40.5, C			
13	2.10, m	53.5, CH	H-14, H-18	C-14	
14	2.28, m<br>2.38, m	31.8, CH_2	H-13	C-18, C-15	
15		160.6, C			
16	5.90, s	125.6, CH		C-18, C-14, C-27	
17		199.2, C			
18	2.58, m	46.1, CH	H-13, H-19	C-13, C-17, C-20	
19	2.39, m<br>2.68, m	42.8, CH_2	H-18	C-18, C-17, C-20<br>C-18, C-13, C-21, C-20	
20		209.5, C			
21	2.61, s	61.8, CH		C-20, C-13, C-28, C-22, C-12, C-23, C-25	H-25, H-29, H-13
22		41.0, C			
23	3.45, d (15.4)<br>3.15, d (14.2)	49.5, CH_2		C-24, C-9, C-25, C-22	
24		213.8, C			
25	0.99, s	23.2, CH_3		C-21, C-23, C-22, C-9	H-21
26					
27	2.02, s	24.5, CH_3		C-14, C-16, C-15	
28	1.32, s	19.8, CH_3		C-11, C-12, C-13, C-21	H-18, H-6
29	0.95, s	24.5, CH_3		C-9, C-8, C-22, C-10	H-10, H-21
30	0.86, d (6.5)	24.1, CH_3	H-4	C-5, C-3, C-4	

Table S4. NMR data for 3-acetoxynorzoanthaminone (**3**) in $CDCl_3$

Position	δ_H, mult (*J* inHz)	δ_C	COSY	HMBC	NOESY
1	(β) 4.03, m	47.0, CH_2	H-2	C-3	
	(α) 2.99, d (9.3)				H-4
2	4.57, m	76.0, CH	H-1, H-3	C-6	
3	4.66, m	72.5, CH	H-2, H-4	C-1'	
4	2.48, m	25.9, CH	H-3, H-30, H-5	C-30	H-1α
5	1.83, m	40.0, CH_2	H-4	C-4, C-30	
	1.40, m				
6		90.9, C			
7	1.85, m	29.7, CH_2	H-8	C-9, C-6	
	1.25, m				
8	1.89, m	24.2, CH_2	H-7	C-9	
	1.62, m				
9		43.4, C			
10		103.9, C			
11		202.5, C			
12		53.5, C			
13	2.59, m	48.3, CH	H-14, H-18	C-21	H-21
14	3.36, dd (3.4, 14.7)	34.5, CH_2	H-13		
	2.26, m				
15		162.4, C			
16	5.91, s	124.7, CH		C-27	
17		198.1, C			
18	2.71, m	47.0, CH	H-13, H-19	C-17	H-28
19	2.60, m	42.8, CH_2	H-18	C-20, C-17, C-21,	
	2.52, m			C-13	
20		207.9, C			
21	2.97, s	59.6, CH		C-20, C-12, C-13, C-22, C-25, C-28	H-13, H-25, H-29
22		37.4, C			
23	4.14, d (20.5)	35.1, CH_2		C-9, C-22, C-21,	
	2.55, m			C-24	
24		170.9, C			
25	1.03, s	21.4, CH_3		C-23, C-22, C-9, C-21	H-21, H-29
26					
27	2.04, s	24.6, CH_3		C-14, C-16, C-15	
28	1.28, s	16.6, CH_3		C-13, C-12, C-21, C-11	H-18
29	1.08, s	17.5, CH_3		C-9, C-22, C-8, C-10	H-21, H-25
30	0.88, d (6.7)	16.3, CH_3		C-4, C-5, C-3	
1'		171.2, C			
2'	2.13, s	21.2, CH_3		C=O	

Table S5. NMR data for 11-hydroxynorzoanthamide B (**4**) in $CDCl_3$

**Position**	**δ_H, mult (*J* inHz)**	**δ_C**	**COSY**	**HMBC**	**NOESY**
1		175.9, C			
2	4.32, q (2.1, 1.8)	77.4, CH	H-3	C-4	
3	1.89, m	32.3, CH_2	H-2, H-4	C-4, C-5	
	1.47, m			C-2, C-4, C-1	
4	2.23, m	24.1, CH	H-3, H-30, H-5	C-30, C-5, C-3	
5	2.20, m	40.6, CH_2	H-4		
	1.20, m			C-30, C-4, C-6	
6		95.2, C			
7	1.88, m	30.0, CH_2	H-8	C-6, C-5, C-8	
	2.14, td (5.0, 13.7)			C-8, C-5	
8	1.69, m	24.5, CH_2	H-7	C-29, C-7, C-9	
	1.57, m			C-6, C-10	
9		39.6, C			
10		97.3, C			
11	4.77, d (6.1)	73.5, CH		C-10, C-21, C-12, C-9, C-28	H-28
12		44.0, C			
13	2.95, m	46.6, CH	H-14, H-18	C-28, C-14, C-12, C-18	
14	2.27, m	32.6, CH_2	H-13	C-13	
	2.50, dd (4.1, 17.9)			C-15, C-16, C-27, C-18, C-13	
15		161.7, C			
16	5.89, s	125.1, CH		C-27, C-18, C-14	
17		199.0, C			
18	2.63, m	45.8, CH	H-13, H-19	C-17, C-14, C-19, C-13	H-28
19	2.69, dd (5.8, 13.6)	43.0, CH_2	H-18	C-18, C-13, C-21, C-20	
	2.44, m			C-20, C-17, C-18, C-21	
20		210.3, C			
21	3.06, s	56.0, CH		C-20, C-28, C-25, C-22, C-12, C-13	H-25, H-29, H-13,
22		36.5, C			
23	3.77, d (20.6)	37.0, CH_2		C-24, C-22, C-9, C-21	
	2.43, d (20.7)			C-24, C-25, C-22, C-21	
24		170.1, C			
25	1.04, s	20.8, CH_3		C-21, C-9, C-22	H-29
26					
27	1.99, s	24.6, CH_3		C-15, C-16, C-14	
28	1.04, s	18.0, CH_3		C-11, C-21, C-13, C-12	H-18
29	1.38, s	17.4, CH_3		C-10, C-8, C-22, C-9	H-25
30	1.00, d (6.1)	21.4, CH_3	H-4	C-4, C-3, C-5	
OH-11	4.54, d (6.2)				

Table S6. NMR data for 11-hydroxyzoanthamide B (**5**) in $CDCl_3$

**Position**	**δ_H, mult (*J* inHz)**	**δ_C**	**COSY**	**HMBC**	**NOESY**
1		175.8, C			
2	4.33, q (3.8, 2.1)	77.4, CH	H-3		
3	1.90, m<br>1.47, m	32.6, CH_2	H-2, H-4	C-4, C-5<br>C-1	
4	2.21, m	24.1, CH	H-3, H-30, H-5	C-30, C-3	
5	2.22, m<br>1.20, m	40.6, CH_2	H-4	 C-30, C-4, C-6	
6		95.2, C			
7	1.88, m<br>2.14, td (4.88, 13.76)	30.1, CH_2	H-8	C-5<br>C-6, C-, C-8	
8	1.66, m<br>1.58, m	24.6, CH_2	H-7	C-29	
9		39.8, C			
10		97.3, C			
11	4.76, d (6.1)	73.4, CH		C-28, C-9, C-21, C-10	H-28, H-14, H-8,
12		43.6, C			
13	3.17, m	41.2, CH	H-18, H-13		H-21, H-26, H-29
14	2.19, m<br>2.47, dd (4.24, 17.60)	31.0, CH_2	H-13	C-18, C-27	
15		161.9, C			
16	5.89, s	126.4, CH			
17		197.9, C			
18	2.58, dd (5.2, 8.72)	47.6, CH	H-13, H-19	C-26, C-14, C-13, C-19, C-17	H-28
19	3.04, m	46.1, CH	H-18	C-20, C-26, C-13, C-18	H-28
20		213.3, C			
21	3.43, s	50.5, CH		C-25, C-12, C-13, C-23, C-22	H-26, H-25, H-29, H-13
22		36.0, C			
23	3.78, d (20.64)<br>2.43, d (20.64)	37.0, CH_2		C-24, C-21, C-9, C-22<br>C-25, C-21	
24		170.1, C			
25	1.00, s	20.5, CH_3		C-21, C-9, C-22, C-23	
26	1.18, d (7.0)	13.4, CH_3	H-19	C-20, C-19, C-18	H-21
27	1.98, s	24.8, CH_3		C-15, C-16, C-14	
28	1.02, s	17.7, CH_3		C-21, C-13, C-12	
29	1.41, s	17.4, CH_3		C-8, C-9, C-22, C-10	H-21, H-25
30	1.00, d (5.5)	21.4, CH_3	H-4	C-4, C-3, C-5	

Table S7. ^{1}H and ^{13}C NMR data in ppm for norzoanthaminone (**6**) and 3-hydroxynorzoanthamine (**9**) in $CDCl_3$.

No.	**6**[a]		**9**[a]	
	δ_H, mult (J in Hz)	δ_C	δ_H, mult (J in Hz)	δ_C
1	3.03, d (8.6)<br>3.99, t (7.6)	48.4, CH_2	3.17, br d (7.3)<br>3.25, t (7.12)	45.1, CH_2
2	4.53, m	74.6, CH	4.51, br dd (2.5, 6.5)	78.2, CH
3	1.44, m<br>1.55, m	38.5, CH_2	3.34, t (3.28)	71.1, CH
4	2.28, m	22.8, CH	2.19, m	27.3, CH
5	1.98, dd (13.4, 5.0)<br>1.09, d (11.4)	44.0, CH_2	1.90, m<br>1.23, dd (7.2, 8)	39.8, CH_2
6		90.7, C		90.1, C
7	1.86, m<br>1.78, m	29.7, CH_2	1.75, dt (3.6, 3.6, 12.7)<br>1.91, m	29.8, CH_2
8	1.86, m<br>1.60, m	24.2, CH_2	1.64, ddd (4.2, 13.8)<br>1.55, dt (3.8, 3.9, 13.8)	23.7, CH_2
9		43.0, C		39.9, C
10		103.3, C		101.5, C
11		202.7, C	1.92, m<br>2.10, d (13.9)	41.9, CH_2
12		53.3, C		36.5, C
13	2.61, dd (3.6, 13.6)	42.6, CH	2.29, ddd (5.9, 13.2, 13.8)	53.1, CH
14	2.28, dd (10.2, 19.7)<br>3.36, dd (18.3, 3.8)	34.3, CH_2	2.26, br d (4.5)<br>2.21, dd (4.5)	32.0, CH_2
15		162.2, C		160.1, C
16	5.91, s	124.6, CH	5.89, s	125.6, CH
17		198.1, C		198.6, C
18	2.71, ddd (13.0, 6.5, 12.1)	48.1, CH	2.69, ddd (6.4, 11.5, 11.4)	46.4, CH
19	2.52, m<br>2.59, m	46.9, CH_2	2.61, dd (6.3, 6.4)<br>2.49, dd (11.4, 14.9)	42.5, CH_2
20		207.9, C		209.1, C
21	2.97, s	59.5, CH	2.83, s	59.1, CH
22		37.3, C		40.0, C
23	4.13, d (20.6)<br>2.54, d (20.1)	35.0, CH_2	3.63, d (20.3)<br>2.34, d (20.3)	35.9, CH_2
24		171.1, C		172.4, C
25	1.02, s	21.3, CH_3	0.96, s	21.1, CH_3
26				
27	2.03, s	24.4, CH_3	1.99, s	24.4, CH_3
28	1.28, s	16.5, CH_3	0.98, s	18.5, CH_3
29	1.07, s	17.4, CH_3	1.15, s	18.5, CH_3
30	0.89, d (6.5)	21.8, CH_3	0.94, d (6.8)	16.8, CH_3

[a] Spectra recorded at 800 and 200 MHz

Table S8. ^{1}H and ^{13}C NMR data in ppm for 3-acetoxynorzoanthamine (**7**) and 3-acetoxyzoanthamine (**8**) in $CDCl_3$.

No.	**2**[b]		**3**[a]	
	δ_H, mult (J in Hz)	δ_C	δ_H, mult (J in Hz)	δ_C
1	3.31, m<br>3.27, m	45.6, CH_2	3.25, t (7.0)<br>3.14, d (6.4)	45.8, CH_2
2	4.61, br d (5.3)	75.3, CH	4.78, td (2.2, 6.3)	76.3, CH
3	4.65, br t (3.6)	72.4, CH	4.85, t (3.96)	73.3, CH
4	2.45, m	26.2, CH	2.52, br sext (4.6)	26.9, CH
5	1.95, m<br>1.40, t (12.7)	40.3, CH_2	1.67, dd (14.1, 6.3)<br>1.53, t (12.5)	40.98, CH_2
6		90.9, C		90.7, C
7	1.92, dd (4.4, 13.3)<br>1.83, dt (12.6, 3.5)	29.7, CH_2	2.39, dd (13.3, 4.5)<br>1.44, dt (12.1, 4.7)	30.7, CH_2
8	1.71, td (13.9, 3.9)<br>1.60, dt (13.9, 4.0)	23.9, CH_2	1.78, td (12.7, 4.1)<br>2.05, dt (13.4, 4.7)	24.3, CH_2
9		40.3, C		41.3
10		100.5, C		101.5
11	2.16, d (13.1)<br>1.97, d (13.3)	42.1, CH_2	2.27, d (13.9)<br>2.08, d (14.0)	42.2, CH_2
12		40.4, C		37.2, CH
13	2.21, m	53.1, CH	2.23, td (12.4, 5.3)	48.3, CH
14	2.29, br s<br>2.30, br s	32.1, CH_2	2.10, br s<br>1.97, br s	31.0, CH_2
15		160.2, C		161.6, C
16	5.9, s	125.7, CH	6.05, s	127.2
17		198.4, C		197.9
18	2.71, td (12.2, 6.3)	46.5, CH	2.63, dd (12.2, 5.3)	48.7, CH
19	2.66, dd (14.7, 6.4)<br>2.52, dd (14.6, 11.3)	42.6, CH_2	2.82, q (8.0, 5.5)	47.0, CH
20		209.1, C		213.2
21	2.85, s	59.3, CH	3.35, s	54.6
22		36.3, C		40.4
23	3.62, d (20.0)<br>2.40, d (20.2)	36.3, CH_2	3.97, d (20.0)<br>2.61, d (20.1)	36.8, CH_2
24		172.4, C		172.2, C
25	1.02, s	21.2, CH_3	1.02, s	21.3, CH_3
26			1.30, d (6.9)	14.2, CH_3
27	2.02, s	24.5, CH_3	1.90, s	24.6, CH_3
28	1.00, s	18.7, CH_3	1.15, s	18.7, CH_3
29	1.18, s	18.7, CH_3	1.23, s	18.6, CH_3
30	0.90, d (6.7)	16.4, CH_3	0.98, d (6.8)	16.9, CH_3
1'		171.3, C		171.3
2'	2.13, s	21.1, CH_3	2.12, s	21.4

[a] Spectra recorded at 800 and 200 MHz, [b] Spectra recorded at 600 and 150 MHz

Table S9. Gibbs free energy of the conformers of compound **2** and the Boltzmann distribution of each conformer.

Conformer	Gibbs free energy (Hartree)	Boltzmann population (%)
2*S*-**2**-1	-1484.861874	30.13
2*S*-**2**-2	-1484.861138	13.82
2*S*-**2**-3	-1484.859064	1.54
2*S*-**2**-4	-1484.861391	18.07
2*S*-**2**-5	-1484.859134	1.65
2*S*-**2**-6	-1484.859029	1.48
2*S*-**2**-7	-1484.861609	22.76
2*S*-**2**-8	-1484.858845	1.22
2*S*-**2**-9	-1484.85895	1.36
2*S*-**2**-10	-1484.860619	7.98
2*R*-**2**-1	-1484.862356	6.15
2*R*-**2**-2	-1484.863834	29.42
2*R*-**2**-3	-1484.862068	4.53
2*R*-**2**-4	-1484.864016	35.68
2*R*-**2**-5	-1484.862672	8.59
2*R*-**2**-6	-1484.863186	14.81
2*R*-**2**-7	-1484.859878	0.45
2*R*-**2**-8	-1484.859682	0.36

Table S10. The cartesian coordinates of the dominant conformers for 2*S*-**2** and 2*R*-**2**

	Coordinates (Å)				Coordinates (Å)		
Conformer 2*S*-**2**-1	X	Y	Z		X	Y	Z
C	5.63573	1.374238	0.00123	H	-2.67771	4.058025	0.005073
C	4.712552	2.265324	0.416802	H	-4.1198	-1.8513	0.822069
C	3.233648	1.976392	0.314313	H	-3.80566	0.409503	1.294413
C	2.905811	0.475426	0.204373	H	6.699046	1.580832	0.091604
C	3.798928	-0.17583	-0.87391	H	2.849967	2.528517	-0.55567
C	5.283319	0.08149	-0.59584	H	2.733744	2.415707	1.184193
C	1.38282	0.144094	-0.01062	H	3.995053	-2.05671	-1.97081
C	1.265418	-1.42804	0.095919	H	3.996144	-2.22375	-0.21758
C	2.05828	-2.0347	-1.06424	H	1.136034	0.672281	2.069713
C	3.535504	-1.68206	-1.05521	H	0.534325	1.86987	0.966997
C	0.571481	0.792431	1.13809	H	-2.62971	-1.27802	2.761548
C	-0.84918	0.242776	1.427045	H	-2.2421	-2.86138	2.111971
C	-0.78729	-1.30432	1.630617	H	4.746808	3.683258	2.029208
C	-0.14229	-2.0447	0.382875	H	6.173638	3.760683	0.970391
C	-2.22449	-1.79188	1.879967	H	4.607481	4.409727	0.435456
O	6.140734	-0.74782	-0.88792	H	-2.60469	2.303521	1.72059
O	1.589053	-2.75016	-1.93504	H	-1.11242	2.646583	0.852868
C	5.093798	3.598689	0.991792	H	-4.70403	2.845824	0.73131
N	-1.88226	0.747799	0.45028	H	-4.82879	3.672684	-0.80636
C	-3.09912	-1.48814	0.667647	H	-4.97797	1.740071	-2.06254
C	-2.09128	2.180914	0.74846	H	-3.21736	0.330023	-1.68225
C	-2.84492	3.01612	-0.29959	H	-4.57507	-0.58407	-1.09821
C	-4.3655	2.809903	-0.31351	H	-1.97954	1.971429	-1.68187
C	-4.89319	1.529673	-0.98938	H	1.281348	0.116502	-2.22578
C	-3.96418	0.298896	-0.88111	H	-0.18071	0.705433	-1.42646
C	-3.20188	0.041873	0.443762	H	1.23014	1.729703	-1.51164
O	-2.2489	2.896542	-1.59272	H	-6.76139	0.392231	-1.03531
H	3.185404	0.029011	1.170694	H	-6.96252	2.086765	-0.56145
H	3.593358	0.330047	-1.83007	H	-6.28528	0.925078	0.585263
C	0.905993	0.69398	-1.37638	H	-0.3991	-0.96381	3.748649
C	-6.30605	1.214128	-0.47277	H	-0.1327	-2.62825	3.244009
C	-0.00532	-1.58687	2.938978	H	1.067175	-1.39403	2.872442
C	0.067585	-3.54974	0.691623	H	-0.86875	-4.04214	0.968982
C	-1.09112	-1.96517	-0.85328	H	0.450351	-4.05866	-0.19422
C	-2.5405	-2.23964	-0.53195	H	0.781183	-3.7076	1.504793
O	-3.23042	-3.00185	-1.19262	H	-1.07678	-0.955	-1.26757
H	1.868501	-1.68167	0.978112	H	-0.75723	-2.64888	-1.63243
H	-1.12578	0.642115	2.420185				

	Coordinates (Å)		
Conformer 2*S*-**2**-2	X	Y	Z
C	5.494466	1.268827	0.08001
C	4.572214	2.15093	0.516526
C	3.092933	1.87247	0.393444
C	2.760257	0.376518	0.239194
C	3.657282	-0.24885	-0.85082
C	5.1413	-0.00312	-0.55926
C	1.237006	0.05765	0.009269
C	1.111446	-1.51585	0.068365
C	1.910842	-2.09097	-1.10342
C	3.390231	-1.74954	-1.07032
C	0.423552	0.686052	1.170975
C	-0.98613	0.115323	1.465801
C	-0.94145	-1.43839	1.598456
C	-0.30301	-2.12985	0.320671
C	-2.38596	-1.92141	1.820761
O	5.99787	-0.82455	-0.87525
O	1.443019	-2.77032	-2.00348
C	4.95492	3.463846	1.135612
N	-2.01093	0.673703	0.518886
C	-3.25582	-1.55569	0.618627
C	-2.24726	2.094265	0.873627
C	-2.39474	3.094642	-0.28111
C	-3.24407	2.626644	-1.47063
C	-4.49338	1.783001	-1.15636
C	-4.13778	0.323607	-0.79556
C	-3.33302	-0.01333	0.479233
O	-1.11591	3.553263	-0.74336
H	3.032442	-0.09726	1.19446
H	3.457973	0.280778	-1.79557
C	0.771158	0.639725	-1.34875
C	-5.48153	2.455219	-0.18885
C	-0.16424	-1.78368	2.893707
C	-0.1127	-3.65015	0.559569
C	-1.24808	-1.97923	-0.9127
C	-2.69868	-2.26518	-0.60613
O	-3.38477	-3.01001	-1.28999
H	1.703928	-1.80042	0.948235
H	-1.24756	0.469614	2.47967

	Coordinates (Å)		
	X	Y	Z
H	-2.85282	3.988366	0.160306
H	-4.28009	-1.91558	0.756517
H	-3.92209	0.306553	1.359788
H	6.558019	1.46774	0.184037
H	2.720388	2.451835	-0.4636
H	2.586008	2.289975	1.269694
H	3.857425	-2.10425	-1.98993
H	3.83933	-2.31267	-0.24066
H	1.002627	0.576953	2.095398
H	0.34965	1.762221	0.993628
H	-2.7898	-1.44179	2.721903
H	-2.41518	-2.99966	2.006559
H	4.598943	3.517852	2.172005
H	6.035691	3.620879	1.128611
H	4.477057	4.29401	0.600629
H	-3.13467	2.165825	1.521999
H	-1.41875	2.467914	1.477232
H	-3.5294	3.513619	-2.0483
H	-2.59959	2.0286	-2.12948
H	-5.02703	1.696137	-2.11309
H	-3.59641	-0.09328	-1.65383
H	-5.0754	-0.24321	-0.72774
H	-0.74034	2.844529	-1.28326
H	1.113221	0.055321	-2.20726
H	-0.31656	0.690605	-1.38943
H	1.152727	1.657063	-1.48003
H	-6.39858	1.862078	-0.10511
H	-5.76186	3.449361	-0.55409
H	-5.08055	2.578002	0.82115
H	-0.57754	-1.21762	3.734783
H	-0.26951	-2.84362	3.136812
H	0.903735	-1.56086	2.846814
H	-1.05311	-4.14051	0.826135
H	0.252992	-4.12496	-0.35192
H	0.607229	-3.85436	1.356816
H	-1.23146	-0.94739	-1.26878
H	-0.91494	-2.62228	-1.7262

	Coordinates (Å)		
Conformer 2*S*-**2**-3	X	Y	Z
C	5.545647	1.242295	-0.16529
C	4.66793	2.135481	0.335782
C	3.179873	1.878749	0.311555
C	2.814588	0.389229	0.170739
C	3.628031	-0.24111	-0.9806
C	5.13139	-0.02142	-0.78475
C	1.275107	0.096583	0.03736
C	1.129881	-1.47842	0.097839
C	1.839266	-2.05954	-1.12663
C	3.321601	-1.73463	-1.19629
C	0.546642	0.713187	1.257123
C	-0.87223	0.181787	1.571872
C	-0.82578	-1.36753	1.750573
C	-0.26856	-2.08649	0.451864
C	-2.25582	-1.83217	2.072538
O	5.952907	-0.85478	-1.15789
O	1.30455	-2.73876	-1.9888
C	5.110959	3.439895	0.932548
N	-1.91078	0.698597	0.621639
C	-3.19497	-1.45009	0.934023
C	-1.92931	2.173588	0.762511
C	-3.14968	2.937819	0.245614
C	-3.47152	2.706079	-1.23021
C	-3.67364	1.219841	-1.58965
C	-4.24573	0.42529	-0.40084
C	-3.25922	0.093004	0.777794
O	-2.8173	4.310353	0.495004
H	3.142153	-0.09547	1.103004
H	3.376703	0.301538	-1.90515
C	0.728264	0.700086	-1.27896
C	-4.56649	1.073031	-2.82908
C	0.022237	-1.69284	3.006519
C	-0.05864	-3.59993	0.715019
C	-1.29048	-1.96922	-0.72132
C	-2.73629	-2.16646	-0.32847
O	-3.50589	-2.84179	-0.99779
H	1.78073	-1.77162	0.932649
H	-1.12335	0.57949	2.575285

	Coordinates (Å)		
	X	Y	Z
H	-4.02732	2.670935	0.850347
H	-4.21585	-1.78843	1.137912
H	-3.71304	0.449748	1.721605
H	6.616539	1.425431	-0.13198
H	2.757899	2.467997	-0.51535
H	2.740368	2.296692	1.223477
H	3.71757	-2.08634	-2.1499
H	3.820054	-2.31399	-0.40694
H	1.148797	0.521417	2.151921
H	0.528622	1.799433	1.155098
H	-2.58932	-1.34756	2.999595
H	-2.28796	-2.91038	2.259673
H	4.825133	3.495598	1.990364
H	6.19123	3.58065	0.854586
H	4.611978	4.279504	0.432636
H	-1.82128	2.459325	1.824506
H	-1.06774	2.592514	0.234793
H	-4.38965	3.2653	-1.46476
H	-2.67264	3.145944	-1.83934
H	-2.6944	0.793995	-1.83196
H	-4.63378	-0.52103	-0.78904
H	-5.11973	0.954083	-0.00182
H	-3.57875	4.843245	0.22927
H	1.056866	0.152055	-2.16673
H	-0.36036	0.716694	-1.27324
H	1.057481	1.736934	-1.39511
H	-4.17522	1.660454	-3.66702
H	-5.58518	1.424892	-2.62472
H	-4.63017	0.028421	-3.15105
H	-0.28779	-1.05389	3.840023
H	-0.13866	-2.72793	3.317805
H	1.097077	-1.55571	2.873535
H	-0.98691	-4.08958	1.023434
H	0.277603	-4.09141	-0.19935
H	0.689345	-3.784	1.491064
H	-1.25487	-0.96262	-1.14301
H	-1.03927	-2.66943	-1.51695

	Coordinates (Å)		
Conformer 2*S*-**2**-4	X	Y	Z
C	5.486245	1.240926	0.062074
C	4.571075	2.12078	0.517867
C	3.090152	1.84652	0.41092
C	2.750342	0.353751	0.244152
C	3.634539	-0.26274	-0.86102
C	5.12201	-0.02453	-0.58419
C	1.223529	0.046635	0.021806
C	1.089532	-1.5275	0.066177
C	1.87821	-2.09703	-1.11498
C	3.35914	-1.75979	-1.09198
C	0.426287	0.661849	1.200354
C	-0.98947	0.104106	1.486294
C	-0.96175	-1.45063	1.603272
C	-0.32751	-2.13656	0.319788
C	-2.41282	-1.91696	1.820106
O	5.973382	-0.84535	-0.9166
O	1.404428	-2.77356	-2.0144
C	4.964896	3.427285	1.143943
N	-2.00402	0.679732	0.541869
C	-3.27564	-1.53481	0.6177
C	-2.22125	2.099973	0.899934
C	-2.35316	3.110294	-0.24216
C	-3.12126	2.621832	-1.47423
C	-4.40457	1.810453	-1.21286
C	-4.09881	0.348044	-0.82274
C	-3.33079	0.008637	0.475323
O	-1.02362	3.512726	-0.60524
H	3.029904	-0.13211	1.191573
H	3.425553	0.277221	-1.79769
C	0.747506	0.651855	-1.32126
C	-5.41641	2.511206	-0.29079
C	-0.18982	-1.81841	2.895459
C	-0.14524	-3.65917	0.549776
C	-1.2724	-1.97321	-0.91225
C	-2.72291	-2.25303	-0.60406
O	-3.41208	-3.00242	-1.28033
H	1.685755	-1.82481	0.939767
H	-1.24974	0.450445	2.503865

	Coordinates (Å)		
	X	Y	Z
H	-2.87898	3.97924	0.186363
H	-4.30506	-1.88135	0.752138
H	-3.93889	0.33824	1.339752
H	6.551484	1.436759	0.15469
H	2.705583	2.433712	-0.43512
H	2.595318	2.255729	1.298006
H	3.816619	-2.10874	-2.01875
H	3.813272	-2.33238	-0.27147
H	1.006357	0.523541	2.120717
H	0.365715	1.740924	1.042998
H	-2.81168	-1.4371	2.723452
H	-2.45664	-2.996	1.999437
H	4.621691	3.473183	2.185041
H	6.045922	3.581975	1.125216
H	4.482606	4.263334	0.622178
H	-3.11305	2.182413	1.540538
H	-1.39258	2.458575	1.512423
H	-3.3677	3.498551	-2.08923
H	-2.43439	2.005444	-2.06539
H	-4.89864	1.727453	-2.19089
H	-3.54772	-0.09348	-1.66181
H	-5.05246	-0.1935	-0.77082
H	-1.09107	4.076147	-1.38838
H	1.102786	0.088974	-2.18959
H	-0.33843	0.704827	-1.35789
H	1.093163	1.683136	-1.42334
H	-6.35329	1.945055	-0.25223
H	-5.65056	3.514568	-0.66355
H	-5.05886	2.617701	0.737364
H	-0.5917	-1.25082	3.741085
H	-0.31344	-2.87808	3.131416
H	0.881842	-1.61417	2.84776
H	-1.08773	-4.1453	0.817423
H	0.214382	-4.13114	-0.36562
H	0.576432	-3.8719	1.343267
H	-1.24476	-0.93929	-1.26132
H	-0.94381	-2.61487	-1.72859

	Coordinates (Å)		
Conformer 2*S*-**2**-5	X	Y	Z
C	5.578798	1.527399	0.075909
C	4.595458	2.423952	0.29593
C	3.141002	2.061372	0.109893
C	2.873969	0.54569	0.170762
C	3.874448	-0.19148	-0.747
C	5.319979	0.15687	-0.3794
C	1.384825	0.123569	-0.11011
C	1.318143	-1.43031	0.176214
C	2.212128	-2.13101	-0.84791
C	3.67283	-1.71782	-0.77745
C	0.46729	0.857088	0.901142
C	-0.9523	0.284863	1.117237
C	-0.83298	-1.21988	1.556268
C	-0.07699	-2.08316	0.457985
C	-2.25017	-1.77547	1.779146
O	6.224826	-0.66601	-0.49133
O	1.831912	-2.96251	-1.65678
C	4.883051	3.83351	0.725201
N	-1.86813	0.470972	-0.04162
C	-3.07786	-1.64171	0.504005
C	-1.95059	1.842858	-0.57332
C	-3.06453	2.770958	-0.03838
C	-4.33469	2.691725	-0.89614
C	-5.0881	1.343256	-0.88733
C	-4.1616	0.110277	-1.01857
C	-3.19945	-0.13412	0.167076
O	-2.50844	4.091749	-0.11566
H	3.092106	0.236539	1.204334
H	3.738924	0.19611	-1.76853
C	0.999978	0.479111	-1.56522
C	-6.04516	1.229122	0.313037
C	-0.13212	-1.27797	2.936969
C	0.19818	-3.51438	0.990449
C	-0.94959	-2.24639	-0.82833
C	-2.43406	-2.45245	-0.60782
O	-3.09259	-3.20205	-1.31575
H	1.871532	-1.54733	1.117825
H	-1.36681	0.817534	1.996889

	Coordinates (Å)		
	X	Y	Z
H	-3.28482	2.527814	1.012512
H	-4.08996	-2.0318	0.650742
H	-3.66318	0.294217	1.075053
H	6.62314	1.787829	0.227624
H	2.816386	2.478953	-0.85424
H	2.553358	2.586444	0.870395
H	4.088044	-2.14625	0.145153
H	4.211216	-2.1715	-1.6108
H	0.952208	0.834975	1.882884
H	0.419913	1.916592	0.642339
H	-2.73667	-1.20771	2.583388
H	-2.21147	-2.81522	2.118775
H	4.43943	4.034127	1.708428
H	5.955119	4.035444	0.777616
H	4.426446	4.547421	0.028313
H	-1.01299	2.357633	-0.3842
H	-2.04625	1.797824	-1.66548
H	-4.02862	2.936378	-1.92155
H	-5.03033	3.483251	-0.58329
H	-5.7193	1.338988	-1.78575
H	-4.80311	-0.77172	-1.11773
H	-3.58938	0.162915	-1.95176
H	-3.21292	4.71938	0.097033
H	1.55695	-0.10673	-2.30191
H	-0.06036	0.315955	-1.74488
H	1.196195	1.535694	-1.77036
H	-6.79664	2.024971	0.277266
H	-5.52872	1.314911	1.274871
H	-6.57383	0.269727	0.305753
H	-0.57827	-0.53733	3.609224
H	-0.27425	-2.25782	3.399488
H	0.942126	-1.08481	2.907534
H	-0.72729	-4.0365	1.249433
H	0.695428	-4.10501	0.219191
H	0.838769	-3.51052	1.876638
H	-0.89969	-1.33702	-1.43011
H	-0.5705	-3.06336	-1.44204

	Coordinates (Å)		
Conformer 2*S*-**2**-6	X	Y	Z
C	5.547175	1.25238	-0.16357
C	4.667011	2.145135	0.333748
C	3.179251	1.886216	0.305564
C	2.81689	0.395689	0.168356
C	3.633805	-0.23672	-0.97932
C	5.136405	-0.01294	-0.78221
C	1.27825	0.09915	0.033135
C	1.137091	-1.47581	0.099896
C	1.849577	-2.05974	-1.12155
C	3.331289	-1.73178	-1.19014
C	0.545227	0.719863	1.248299
C	-0.87166	0.184348	1.564821
C	-0.82009	-1.36401	1.750265
C	-0.25992	-2.0863	0.454657
C	-2.24891	-1.8321	2.073317
O	5.960284	-0.84454	-1.15379
O	1.317559	-2.74292	-1.98217
C	5.106772	3.450869	0.930082
N	-1.91088	0.693082	0.61131
C	-3.18853	-1.45886	0.932077
C	-1.94094	2.170594	0.745121
C	-3.18304	2.931539	0.257249
C	-3.52962	2.713492	-1.21365
C	-3.68787	1.226782	-1.58998
C	-4.24522	0.407821	-0.41113
C	-3.25745	0.083236	0.768942
O	-2.99312	4.320365	0.547766
H	3.143546	-0.08526	1.10284
H	3.382824	0.30186	-1.90641
C	0.732493	0.695276	-1.28707
C	-4.57229	1.071316	-2.83439
C	0.028451	-1.68093	3.00797
C	-0.04619	-3.59821	0.723442
C	-1.28128	-1.97616	-0.71978
C	-2.72591	-2.17996	-0.32627
O	-3.4914	-2.86363	-0.99173
H	1.787658	-1.76402	0.936625
H	-1.12489	0.585641	2.566221
H	-4.04312	2.652144	0.872932
H	-4.2085	-1.7996	1.136611
H	-3.71241	0.441446	1.711426
H	6.617722	1.437042	-0.12779
H	2.75922	2.472105	-0.52483
H	2.736271	2.306013	1.215001
H	3.729599	-2.08587	-2.14189
H	3.829758	-2.30723	-0.39791
H	1.147231	0.53698	2.145069
H	0.522022	1.805634	1.139378
H	-2.58493	-1.34422	2.997722
H	-2.27732	-2.90949	2.265633
H	4.818757	3.507119	1.987293
H	6.186991	3.593088	0.854315
H	4.607835	4.289392	0.428237
H	-1.81134	2.462202	1.802601
H	-1.0874	2.587371	0.197385
H	-4.46322	3.25374	-1.41709
H	-2.75605	3.178804	-1.84129
H	-2.69711	0.827852	-1.83202
H	-4.61316	-0.54177	-0.81054
H	-5.131	0.914858	-0.00936
H	-2.31359	4.651171	-0.05721
H	1.061999	0.142725	-2.17165
H	-0.35625	0.710872	-1.28237
H	1.062288	1.731449	-1.40876
H	-4.19224	1.676562	-3.66483
H	-5.59959	1.396799	-2.63032
H	-4.60962	0.028869	-3.16726
H	-0.28536	-1.04058	3.839002
H	-0.1277	-2.71567	3.322796
H	1.102767	-1.53907	2.8754
H	-0.97343	-4.08908	1.032925
H	0.292027	-4.09216	-0.18887
H	0.701632	-3.77749	1.500779
H	-1.25089	-0.97008	-1.1433
H	-1.02615	-2.67702	-1.51357

	Coordinates (Å)		
Conformer 2*S*-**2**-7	X	Y	Z
C	5.567302	1.116603	0.034337
C	4.688125	2.055911	0.440394
C	3.197606	1.854987	0.293603
C	2.797896	0.374387	0.155651
C	3.667171	-0.27797	-0.94589
C	5.153161	-0.14009	-0.60173
C	1.259668	0.117144	-0.05352
C	1.088987	-1.45069	-0.03954
C	1.806069	-1.9941	-1.27428
C	3.304504	-1.74279	-1.26064
C	0.493405	0.747532	1.148837
C	-0.87888	0.162258	1.582235
C	-0.82061	-1.40075	1.649913
C	-0.32283	-2.03176	0.283696
C	-2.22646	-1.92206	1.990638
O	5.960925	-1.02371	-0.87414
O	1.263275	-2.5757	-2.20007
C	5.131908	3.353772	1.050343
N	-2.02142	0.759284	0.800488
C	-3.22054	-1.47751	0.921616
C	-2.20825	2.178753	1.211223
C	-2.44548	3.133825	0.036174
C	-3.77405	2.939346	-0.70124
C	-4.07694	1.506544	-1.17572
C	-4.44219	0.57045	-0.00579
C	-3.32721	0.067427	0.968438
O	-1.34042	3.063513	-0.86732
H	3.061409	-0.10831	1.108661
H	3.535026	0.312357	-1.86537
C	0.826827	0.744826	-1.39878
C	-5.23239	1.525923	-2.19006
C	0.085818	-1.78727	2.845373
C	-0.16922	-3.57019	0.399603
C	-1.37174	-1.76047	-0.83624
C	-2.80543	-2.02262	-0.43552
O	-3.59971	-2.59075	-1.17112
H	1.721187	-1.78677	0.791921
H	-1.01578	0.477222	2.632136

	Coordinates (Å)		
	X	Y	Z
H	-2.4277	4.154661	0.438057
H	-4.22122	-1.86802	1.131539
H	-3.68472	0.26552	1.996125
H	6.637919	1.254405	0.161548
H	2.867982	2.43123	-0.5831
H	2.696156	2.313383	1.152571
H	3.729023	-2.05667	-2.21566
H	3.739088	-2.3892	-0.48624
H	1.140101	0.66839	2.030051
H	0.385424	1.818178	0.959454
H	-2.53925	-1.51366	2.960367
H	-2.22269	-3.01005	2.103387
H	4.758253	3.443677	2.077974
H	6.219787	3.44948	1.06434
H	4.713494	4.201507	0.493506
H	-3.03214	2.265057	1.937482
H	-1.31184	2.52544	1.728666
H	-4.5963	3.275947	-0.05352
H	-3.75838	3.616392	-1.56354
H	-3.19421	1.109567	-1.69371
H	-4.92179	-0.3171	-0.43143
H	-5.22039	1.06944	0.585475
H	-1.22363	2.119986	-1.04821
H	1.400022	0.355827	-2.24427
H	-0.21765	0.546317	-1.63302
H	0.959917	1.828823	-1.38015
H	-4.99745	2.173016	-3.04148
H	-6.1533	1.904293	-1.72932
H	-5.44013	0.522391	-2.57531
H	-0.2841	-1.30586	3.756342
H	0.060167	-2.8657	3.0189
H	1.131474	-1.4961	2.729106
H	-1.10514	-4.05483	0.689806
H	0.120985	-3.9839	-0.56719
H	0.594244	-3.85457	1.129134
H	-1.37242	-0.6981	-1.0883
H	-1.13149	-2.32408	-1.73766

	Coordinates (Å)		
Conformer 2*S*-**2**-8	X	Y	Z
C	5.58239	1.538317	-0.04927
C	4.611003	2.418698	0.267137
C	3.149391	2.057722	0.14777
C	2.887564	0.539887	0.157106
C	3.839022	-0.16046	-0.83696
C	5.302615	0.18701	-0.54685
C	1.386415	0.125925	-0.06691
C	1.329581	-1.4355	0.17211
C	2.184646	-2.10555	-0.9061
C	3.644971	-1.68683	-0.88945
C	0.518591	0.8303	1.004522
C	-0.88988	0.249002	1.285445
C	-0.77895	-1.26952	1.627993
C	-0.06075	-2.08753	0.470466
C	-2.20777	-1.78882	1.862515
O	6.203844	-0.62222	-0.75014
O	1.77605	-2.91784	-1.72077
C	4.919627	3.809296	0.741264
N	-1.86998	0.589392	0.210709
C	-3.04443	-1.61331	0.598148
C	-1.96859	2.070361	0.093739
C	-3.33568	2.791566	0.071876
C	-4.18201	2.573968	-1.18392
C	-4.89631	1.216236	-1.26798
C	-3.92974	0.029649	-1.10321
C	-3.17779	-0.10734	0.247906
O	-3.05579	4.190468	0.236039
H	3.157015	0.189525	1.165111
H	3.639087	0.249955	-1.83903
C	0.923733	0.535868	-1.48655
C	-6.10229	1.129814	-0.31666
C	-0.03936	-1.40959	2.982409
C	0.202708	-3.54713	0.923294
C	-0.96599	-2.16576	-0.79899
C	-2.42186	-2.44231	-0.51378
O	-3.0718	-3.26286	-1.14596
H	1.913436	-1.58519	1.090517
H	-1.22867	0.734237	2.222129

	Coordinates (Å)		
	X	Y	Z
H	-3.91005	2.530082	0.966058
H	-4.06037	-1.99218	0.747322
H	-3.80759	0.293893	1.061239
H	6.632986	1.797988	0.05191
H	2.771561	2.517103	-0.77711
H	2.603566	2.545879	0.962138
H	4.102051	-2.13108	0.005537
H	4.147596	-2.121	-1.75478
H	1.056472	0.802682	1.958917
H	0.439544	1.889027	0.750258
H	-2.66444	-1.22112	2.684158
H	-2.19943	-2.83658	2.179791
H	4.536174	3.962814	1.757676
H	5.99228	4.014783	0.737964
H	4.419639	4.5513	0.106258
H	-1.46419	2.529537	0.95007
H	-1.42098	2.4051	-0.8015
H	-4.92582	3.378577	-1.22216
H	-3.53097	2.701188	-2.06156
H	-5.29789	1.13786	-2.28726
H	-4.50852	-0.88649	-1.26459
H	-3.17767	0.058728	-1.90114
H	-2.63189	4.495383	-0.57962
H	1.351004	-0.0941	-2.27208
H	-0.16132	0.490624	-1.5678
H	1.212559	1.56865	-1.70274
H	-6.81816	1.931345	-0.52725
H	-5.818	1.215628	0.73731
H	-6.62362	0.174289	-0.43795
H	-0.47713	-0.72497	3.716268
H	-0.15169	-2.42134	3.379462
H	1.029983	-1.19371	2.934168
H	-0.72201	-4.05519	1.211811
H	0.641668	-4.11598	0.102247
H	0.889759	-3.59781	1.772367
H	-0.96365	-1.20301	-1.31421
H	-0.58613	-2.91644	-1.49066

	Coordinates (Å)		
Conformer 2*S*-**2**-9	X	Y	Z
C	5.543446	1.258653	-0.16532
C	4.661019	2.152016	0.326945
C	3.173923	1.889693	0.299845
C	2.814572	0.397894	0.168385
C	3.633735	-0.23745	-0.97607
C	5.135653	-0.01066	-0.77763
C	1.276559	0.098377	0.032566
C	1.137289	-1.47644	0.104574
C	1.852558	-2.06423	-1.11347
C	3.333663	-1.73359	-1.18189
C	0.542037	0.722048	1.245298
C	-0.87431	0.185552	1.562614
C	-0.82321	-1.36208	1.751469
C	-0.25985	-2.08722	0.458665
C	-2.25315	-1.82897	2.072449
O	5.961352	-0.84328	-1.14298
O	1.323223	-2.75298	-1.97129
C	5.097573	3.462032	0.916206
N	-1.91221	0.691779	0.608098
C	-3.19008	-1.46017	0.927193
C	-1.94115	2.168899	0.732001
C	-3.1864	2.929728	0.251651
C	-3.52907	2.720777	-1.21745
C	-3.67417	1.235289	-1.59927
C	-4.23627	0.40327	-0.43132
C	-3.25869	0.081245	0.757899
O	-2.93923	4.33016	0.423764
H	3.14118	-0.079	1.105034
H	3.383084	0.297563	-1.90526
C	0.731498	0.689666	-1.29004
C	-4.54637	1.080576	-2.85242
C	0.022705	-1.67614	3.011574
C	-0.04575	-3.59826	0.732054
C	-1.27907	-1.98092	-0.71815
C	-2.72328	-2.18805	-0.3258
O	-3.48488	-2.88108	-0.98593
H	1.786725	-1.76117	0.943438
H	-1.12806	0.588754	2.563616

	Coordinates (Å)		
	X	Y	Z
H	-4.05135	2.641685	0.863993
H	-4.21056	-1.80042	1.130056
H	-3.72126	0.441919	1.695964
H	6.613563	1.445924	-0.13005
H	2.752363	2.471598	-0.53245
H	2.730114	2.312212	1.207632
H	3.733345	-2.09052	-2.132
H	3.832385	-2.30556	-0.38727
H	1.143997	0.543232	2.14301
H	0.516479	1.807226	1.131505
H	-2.59141	-1.33773	2.994342
H	-2.28208	-2.90563	2.268737
H	4.808974	3.523559	1.972976
H	6.177497	3.606312	0.840053
H	4.596829	4.296453	0.409419
H	-1.80047	2.456266	1.792965
H	-1.09809	2.592795	0.178786
H	-4.46825	3.252643	-1.41746
H	-2.759	3.202629	-1.8323
H	-2.67886	0.844077	-1.83547
H	-4.59087	-0.54729	-0.84064
H	-5.13092	0.899426	-0.03552
H	-2.88844	4.506883	1.37324
H	1.063272	0.134874	-2.17238
H	-0.35714	0.703937	-1.28739
H	1.058773	1.72617	-1.41438
H	-4.16336	1.694124	-3.6753
H	-5.57789	1.397323	-2.65574
H	-4.57315	0.040089	-3.19242
H	-0.29397	-1.03523	3.841127
H	-0.13244	-2.71071	3.327408
H	1.097048	-1.53258	2.881104
H	-0.97293	-4.08854	1.042677
H	0.29307	-4.09471	-0.17861
H	0.701857	-3.77491	1.510194
H	-1.25017	-0.9754	-1.14304
H	-1.02065	-2.68258	-1.51014

	Coordinates (Å)				Coordinates (Å)		
Conformer 2*S*-**2**-10	X	Y	Z		X	Y	Z
C	5.645615	1.372152	0.025439	H	-2.65198	4.027687	-0.06501
C	4.725809	2.253772	0.467657	H	-4.12075	-1.83772	0.795973
C	3.246194	1.96792	0.369282	H	-3.74151	0.424876	1.310558
C	2.914821	0.470523	0.225995	H	6.709683	1.576538	0.112506
C	3.802172	-0.15877	-0.86964	H	2.85602	2.540881	-0.48421
C	5.288294	0.093052	-0.5977	H	2.753485	2.386668	1.253342
C	1.390217	0.148211	0.007857	H	3.992039	-2.02019	-2.00056
C	1.268869	-1.42663	0.085623	H	4.001586	-2.2177	-0.25088
C	2.059765	-2.0164	-1.08417	H	1.133959	0.586485	2.107511
C	3.536925	-1.66101	-1.07652	H	0.576027	1.85185	1.064371
C	0.585234	0.766774	1.176493	H	-2.63158	-1.3051	2.745202
C	-0.84847	0.228911	1.415427	H	-2.24612	-2.88131	2.075792
C	-0.78793	-1.31963	1.618038	H	4.771547	3.635427	2.111139
C	-0.13918	-2.04866	0.365154	H	6.192008	3.735158	1.045661
C	-2.226	-1.80867	1.857614	H	4.623369	4.397473	0.534831
O	6.144315	-0.72847	-0.91554	H	-2.28327	2.418705	1.57471
O	1.592436	-2.72664	-1.96033	H	-0.96717	2.595266	0.417895
C	5.112117	3.573674	1.069975	H	-4.58059	2.798067	0.85758
N	-1.83897	0.704056	0.393411	H	-4.89336	3.661133	-0.62875
C	-3.09555	-1.4844	0.647904	H	-5.15509	1.736932	-1.91649
C	-1.9605	2.167179	0.544311	H	-3.22996	0.490256	-1.65904
C	-2.85897	2.992807	-0.38811	H	-4.53021	-0.56796	-1.13755
C	-4.36962	2.78652	-0.2201	H	-2.93914	3.405702	-2.29517
C	-4.95195	1.513786	-0.86085	H	1.276272	0.153747	-2.20395
C	-3.96549	0.330888	-0.86709	H	-0.17854	0.757097	-1.39553
C	-3.17547	0.04816	0.435588	H	1.253102	1.756019	-1.47055
O	-2.40807	2.824183	-1.73389	H	-6.77782	0.320144	-0.72748
H	3.197514	0.002114	1.181106	H	-6.98366	2.00447	-0.22021
H	3.58966	0.364833	-1.81469	H	-6.15823	0.858422	0.841596
C	0.9087	0.724946	-1.34632	H	-0.3876	-0.97318	3.733056
C	-6.29388	1.152961	-0.20628	H	-0.17501	-2.64967	3.241533
C	-0.01418	-1.61518	2.928256	H	1.064189	-1.45913	2.861024
C	0.074725	-3.5548	0.665354	H	-0.86293	-4.05289	0.928172
C	-1.08618	-1.96398	-0.87316	H	0.469675	-4.05584	-0.21976
C	-2.539	-2.22744	-0.55778	H	0.779311	-3.71565	1.485983
O	-3.23441	-2.97559	-1.23002	H	-1.06595	-0.95267	-1.28547
H	1.873696	-1.69642	0.962289	H	-0.75605	-2.65206	-1.65019
H	-1.16064	0.635293	2.398516				

Conformer 2*R*-**2**-1	Coordinates (Å) X	Y	Z
C	5.614529	1.425261	0.004304
C	4.67264	2.305299	0.401062
C	3.199956	1.98888	0.292216
C	2.898622	0.481175	0.199053
C	3.812059	-0.16904	-0.86254
C	5.289448	0.118625	-0.57774
C	1.383535	0.121677	-0.02494
C	1.291123	-1.45155	0.104513
C	2.105951	-2.06366	-1.03695
C	3.576334	-1.68194	-1.02355
C	0.551778	0.765745	1.110563
C	-0.87236	0.207022	1.354096
C	-0.78475	-1.33234	1.609863
C	-0.10766	-2.09182	0.39054
C	-2.21535	-1.84247	1.850206
O	6.164111	-0.69865	-0.85279
O	1.66014	-2.80745	-1.89639
C	5.026238	3.652732	0.960835
N	-1.85997	0.627298	0.303005
C	-3.07874	-1.57502	0.622177
C	-2.02693	2.087797	0.405015
C	-2.84748	2.82185	-0.66908
C	-4.36286	2.691307	-0.49177
C	-4.96528	1.366059	-1.00109
C	-3.98293	0.177775	-0.94675
C	-3.18499	-0.05163	0.362247
H	-2.54444	2.472608	-1.66782
H	3.177479	0.05233	1.17361
H	3.604758	0.319306	-1.82744
C	0.90712	0.642246	-1.40302
C	-6.28451	1.058818	-0.27716
C	-0.02026	-1.56636	2.937799
C	0.133898	-3.58173	0.746428
C	-1.04252	-2.07336	-0.85995
C	-2.49693	-2.34352	-0.55519
O	-3.17568	-3.11407	-1.21887
H	1.890931	-1.67954	0.99619
H	-1.20558	0.6408	2.317292

	Coordinates (Å) X	Y	Z
O	-2.44508	4.189571	-0.49342
H	-4.09834	-1.94333	0.7724
H	-3.7591	0.344065	1.222547
H	6.673369	1.651825	0.099561
H	2.812441	2.523339	-0.58713
H	2.686553	2.430409	1.15305
H	4.048939	-2.06205	-1.93026
H	4.041034	-2.20301	-0.17506
H	1.093399	0.634222	2.053752
H	0.519953	1.845133	0.955806
H	-2.2163	-2.90782	2.102017
H	-2.6409	-1.31968	2.716936
H	6.103271	3.8333	0.945405
H	4.530057	4.44767	0.390137
H	4.669638	3.745077	1.994311
H	-1.04387	2.550942	0.351985
H	-2.45236	2.364071	1.389861
H	-4.58819	2.831226	0.573761
H	-4.85615	3.521862	-1.01426
H	-5.20305	1.50004	-2.0651
H	-3.25257	0.265713	-1.75824
H	-4.55857	-0.72793	-1.16645
H	1.309188	0.061661	-2.23845
H	-0.17981	0.627227	-1.4669
H	1.214365	1.682088	-1.54944
H	-6.9764	1.905743	-0.34393
H	-6.11677	0.856021	0.78735
H	-6.78052	0.184168	-0.71116
H	-0.16251	-2.5927	3.284931
H	1.055164	-1.38887	2.87666
H	-0.41653	-0.90535	3.715734
H	-0.79652	-4.0905	1.014587
H	0.551211	-4.10482	-0.11548
H	0.830725	-3.69885	1.580886
H	-1.03189	-1.08115	-1.31644
H	-0.6936	-2.78736	-1.60466
H	-2.98471	4.72876	-1.08706

Conformer 2*R*-**2**-2	Coordinates (Å) X	Y	Z
C	5.562395	1.094309	-0.08564
C	4.707199	1.990582	0.447919
C	3.211966	1.78347	0.392806
C	2.804768	0.313529	0.180084
C	3.608398	-0.279	-0.99888
C	5.115451	-0.1227	-0.77268
C	1.258344	0.069127	0.026864
C	1.074201	-1.49949	-2.7E-05
C	1.760519	-2.02249	-1.26273
C	3.253431	-1.74493	-1.31144
C	0.54143	0.654372	1.272792
C	-0.86679	0.118648	1.63454
C	-0.84233	-1.44736	1.693828
C	-0.33784	-2.08191	0.329679
C	-2.2646	-1.93991	2.008409
O	5.91363	-0.96466	-1.1756
O	1.19977	-2.61831	-2.16886
C	5.183671	3.248633	1.114304
N	-1.95095	0.702818	0.781828
C	-3.23644	-1.45526	0.936969
C	-2.06714	2.164015	0.987642
C	-2.16933	2.954541	-0.31998
C	-3.48132	2.764434	-1.08476
C	-3.93311	1.307872	-1.30128
C	-4.37307	0.654909	0.027903
C	-3.28255	0.091534	0.987057
H	-1.33597	2.640156	-0.96285
H	3.113667	-0.2257	1.088422
H	3.388806	0.327803	-1.891
C	0.743876	0.748453	-1.26339
C	-5.09386	1.263017	-2.30781
C	0.036004	-1.8696	2.898467
C	-0.17586	-3.61841	0.466127
C	-1.37585	-1.83454	-0.80819
C	-2.82992	-1.99969	-0.42205
O	-3.64865	-2.49695	-1.18288
H	1.720891	-1.86044	0.810738
H	-1.0519	0.442599	2.676893

	Coordinates (Å) X	Y	Z
O	-1.99743	4.328342	0.055455
H	-4.2506	-1.81417	1.138148
H	-3.62155	0.313364	2.018809
H	6.638159	1.239426	-0.02954
H	2.82012	2.420518	-0.41336
H	2.772069	2.175076	1.316089
H	3.639826	-2.04599	-2.28617
H	3.730757	-2.39043	-0.56146
H	1.169765	0.464176	2.150301
H	0.511565	1.742184	1.174217
H	-2.28809	-3.02977	2.102437
H	-2.57806	-1.54059	2.981835
H	6.268983	3.356944	1.05742
H	4.719689	4.128011	0.650268
H	4.884372	3.261622	2.169805
H	-1.19277	2.545924	1.517408
H	-2.92777	2.405599	1.631079
H	-4.26994	3.300979	-0.53817
H	-3.38157	3.263563	-2.05896
H	-3.09902	0.737706	-1.72868
H	-5.04911	-0.17466	-0.20374
H	-4.98271	1.387147	0.571255
H	1.093999	0.254075	-2.17387
H	-0.34325	0.765469	-1.28952
H	1.073449	1.790804	-1.30782
H	-5.39294	0.230433	-2.51448
H	-4.81353	1.730355	-3.25779
H	-5.97134	1.795732	-1.92095
H	-0.08588	-2.93469	3.109952
H	1.103275	-1.6815	2.766032
H	-0.28104	-1.32545	3.794154
H	-1.11463	-4.10409	0.746383
H	0.132467	-4.04208	-0.49082
H	0.576315	-3.88984	1.212005
H	-1.16109	-2.47094	-1.66646
H	-1.31309	-0.79876	-1.14668
H	-2.12421	4.865344	-0.73846

	Coordinates (Å)		
Conformer 2*R*-**2**-3	X	Y	Z
C	5.623621	1.422021	0.009362
C	4.681713	2.30688	0.394785
C	3.208809	1.993247	0.278065
C	2.904825	0.485514	0.194985
C	3.822208	-0.1738	-0.85773
C	5.298714	0.112477	-0.56665
C	1.39012	0.126261	-0.03233
C	1.296249	-1.44606	0.108332
C	2.113486	-2.06666	-1.02685
C	3.584389	-1.68721	-1.01101
C	0.553674	0.778982	1.09501
C	-0.87189	0.222087	1.335191
C	-0.78193	-1.31454	1.608058
C	-0.10277	-2.08471	0.397014
C	-2.21156	-1.82488	1.852453
O	6.172986	-0.7083	-0.83163
O	1.668876	-2.81509	-1.88272
C	5.035244	3.656633	0.949005
N	-1.85658	0.628413	0.275468
C	-3.07459	-1.56792	0.622359
C	-2.00833	2.095286	0.34368
C	-2.90224	2.827492	-0.68061
C	-4.39975	2.65897	-0.43404
C	-4.99018	1.348334	-0.98406
C	-3.99172	0.173245	-0.9531
C	-3.1834	-0.0467	0.350958
H	-2.64547	2.506541	-1.7011
H	3.178727	0.063831	1.173973
H	3.621301	0.309035	-1.82677
C	0.919614	0.636249	-1.41628
C	-6.30701	1.007653	-0.27145
C	-0.01878	-1.53362	2.939406
C	0.139864	-3.57099	0.767062
C	-1.03624	-2.07893	-0.85458
C	-2.49183	-2.34418	-0.54957
O	-3.17093	-3.11682	-1.21039
H	1.894238	-1.6676	1.002784
H	-1.21094	0.666324	2.291685

	Coordinates (Å)		
	X	Y	Z
O	-2.64396	4.231517	-0.53423
H	-4.09385	-1.93616	0.774974
H	-3.75014	0.355349	1.212848
H	6.682418	1.646309	0.110034
H	2.829293	2.520929	-0.60898
H	2.690586	2.442719	1.13194
H	4.059355	-2.07307	-1.91403
H	4.045458	-2.20436	-0.15819
H	1.090233	0.651636	2.041483
H	0.524775	1.85805	0.936061
H	-2.21077	-2.88815	2.112766
H	-2.63807	-1.29589	2.714919
H	6.112811	3.834201	0.939474
H	4.545283	4.449799	0.37045
H	4.672471	3.755904	1.979692
H	-1.01716	2.532212	0.214789
H	-2.35796	2.406861	1.347495
H	-4.57877	2.734274	0.646865
H	-4.91531	3.511305	-0.88896
H	-5.22615	1.511652	-2.04449
H	-3.26766	0.280776	-1.76793
H	-4.55179	-0.74102	-1.1769
H	1.329134	0.052608	-2.24582
H	-0.16687	0.614669	-1.48727
H	1.224778	1.676236	-1.56758
H	-7.00598	1.850268	-0.31349
H	-6.13846	0.774232	0.786742
H	-6.79487	0.142026	-0.73193
H	-0.1634	-2.55546	3.298628
H	1.057117	-1.35921	2.877649
H	-0.41475	-0.86274	3.709017
H	-0.79086	-4.07887	1.035736
H	0.561475	-4.1011	-0.08848
H	0.833385	-3.67966	1.605417
H	-1.0249	-1.09179	-1.322
H	-0.68733	-2.80121	-1.59129
H	-1.72305	4.39241	-0.78153

	Coordinates (Å)		
Conformer 2*R*-**2**-4	X	Y	Z
C	5.560186	1.091394	-0.09051
C	4.706339	1.987961	0.444698
C	3.211009	1.780765	0.393894
C	2.802976	0.310897	0.181869
C	3.603853	-0.28176	-0.99884
C	5.111417	-0.12599	-0.77559
C	1.256079	0.067272	0.032098
C	1.070363	-1.50119	0.004369
C	1.755141	-2.02484	-1.25907
C	3.24795	-1.74763	-1.31013
C	0.542986	0.650445	1.280829
C	-0.86721	0.11879	1.639925
C	-0.84742	-1.44731	1.698031
C	-0.34226	-2.0827	0.334366
C	-2.27189	-1.93527	2.010249
O	5.908413	-0.96873	-1.17927
O	1.193209	-2.62185	-2.16365
C	5.184426	3.246761	1.108502
N	-1.94778	0.705372	0.784548
C	-3.24068	-1.44812	0.937067
C	-2.05811	2.168376	0.983815
C	-2.15973	2.96662	-0.32553
C	-3.457	2.762415	-1.1119
C	-3.91027	1.304075	-1.31369
C	-4.36341	0.665393	0.018216
C	-3.28155	0.098798	0.984624
H	-1.31339	2.676254	-0.95409
H	3.113654	-0.22875	1.089382
H	3.382554	0.324963	-1.89058
C	0.738284	0.749086	-1.25561
C	-5.06193	1.251407	-2.33027
C	0.027767	-1.87305	2.903752
C	-0.18111	-3.61916	0.472571
C	-1.3791	-1.83645	-0.80497
C	-2.83398	-1.99675	-0.42022
O	-3.65318	-2.49395	-1.18052
H	1.717269	-1.86299	0.814657
H	-1.05388	0.442758	2.682074

	Coordinates (Å)		
	X	Y	Z
O	-1.94816	4.359377	-0.05829
H	-4.25642	-1.80315	1.137251
H	-3.62516	0.323496	2.014502
H	6.636084	1.236657	-0.03745
H	2.816833	2.418268	-0.4107
H	2.774017	2.172098	1.318697
H	3.632723	-2.0493	-2.2853
H	3.726254	-2.39293	-0.56058
H	1.170784	0.45356	2.157261
H	0.518218	1.73875	1.18699
H	-2.29906	-3.02505	2.104293
H	-2.58554	-1.5349	2.983226
H	6.269603	3.354961	1.048884
H	4.719365	4.125488	0.644346
H	4.887632	3.261107	2.164705
H	-1.18046	2.548123	1.509085
H	-2.9163	2.409783	1.63463
H	-4.26585	3.298659	-0.58939
H	-3.3393	3.257463	-2.08305
H	-3.0736	0.728268	-1.72779
H	-5.04693	-0.15918	-0.20928
H	-4.96849	1.407313	0.553955
H	1.08627	0.256438	-2.16783
H	-0.34894	0.765103	-1.27922
H	1.067522	1.791525	-1.29931
H	-5.36075	0.217293	-2.52949
H	-4.77212	1.709355	-3.28183
H	-5.94222	1.789039	-1.95658
H	-0.09994	-2.93728	3.116052
H	1.096119	-1.69088	2.771698
H	-0.28689	-1.32647	3.798824
H	-1.12014	-4.10392	0.753645
H	0.126765	-4.0441	-0.48394
H	0.571093	-3.89014	1.218569
H	-1.16514	-2.47612	-1.66096
H	-1.31298	-0.80234	-1.1477
H	-2.72576	4.682927	0.419009

	Coordinates (Å)		
Conformer 2*R*-**2**-5	X	Y	Z
C	5.618712	1.429885	-0.00062
C	4.675242	2.313453	0.384173
C	3.203007	1.995183	0.274047
C	2.90263	0.486478	0.194464
C	3.819647	-0.17288	-0.85852
C	5.295875	0.117207	-0.5703
C	1.3883	0.124034	-0.03021
C	1.296745	-1.4482	0.112597
C	2.114762	-2.06981	-1.0215
C	3.584759	-1.687	-1.00802
C	0.553269	0.776828	1.098106
C	-0.87102	0.218589	1.341714
C	-0.78275	-1.31833	1.611768
C	-0.10203	-2.08771	0.40087
C	-2.2136	-1.82739	1.853247
O	6.17153	-0.703	-0.83297
O	1.671513	-2.822	-1.87486
C	5.02661	3.666795	0.930964
N	-1.85628	0.628036	0.284797
C	-3.07433	-1.57061	0.621161
C	-2.01286	2.092617	0.361428
C	-2.88403	2.834508	-0.67447
C	-4.39165	2.665324	-0.4718
C	-4.9769	1.344357	-1.00771
C	-3.97613	0.172112	-0.96369
C	-3.18129	-0.04951	0.348593
H	-2.59443	2.533786	-1.68715
H	3.179064	0.06688	1.173734
H	3.615932	0.307592	-1.82809
C	0.915238	0.632115	-1.41403
C	-6.29591	1.007369	-0.29722
C	-0.02148	-1.53963	2.943721
C	0.14039	-3.57409	0.770769
C	-1.03406	-2.08162	-0.85183
C	-2.48936	-2.34848	-0.5486
O	-3.16719	-3.12272	-1.20883
H	1.894696	-1.66805	1.007568
H	-1.20795	0.660965	2.30007

	Coordinates (Å)		
	X	Y	Z
O	-2.52905	4.223877	-0.59551
H	-4.09412	-1.9383	0.771683
H	-3.7567	0.352007	1.205405
H	6.677142	1.657829	0.095683
H	2.817846	2.520861	-0.61159
H	2.686932	2.444703	1.129179
H	4.059507	-2.07415	-1.91063
H	4.047843	-2.20123	-0.15451
H	1.092391	0.652059	2.043618
H	0.520956	1.855085	0.935758
H	-2.21438	-2.89056	2.113964
H	-2.64136	-1.29773	2.714745
H	6.103647	3.84739	0.91728
H	4.53243	4.455527	0.35003
H	4.666559	3.770053	1.962211
H	-1.03057	2.54758	0.252453
H	-2.38692	2.385244	1.36592
H	-4.6019	2.758476	0.604165
H	-4.89539	3.508068	-0.95786
H	-5.20907	1.496761	-2.07035
H	-3.24328	0.281859	-1.77035
H	-4.53297	-0.74225	-1.19512
H	1.321939	0.045791	-2.24313
H	-0.17131	0.613206	-1.48246
H	1.220097	1.671486	-1.56824
H	-6.99778	1.846826	-0.35221
H	-6.13159	0.786031	0.764245
H	-6.77853	0.135052	-0.75043
H	-0.16466	-2.56257	3.300313
H	1.054104	-1.36286	2.883563
H	-0.41963	-0.87128	3.714409
H	-0.79012	-4.08146	1.041213
H	0.560384	-4.1045	-0.08531
H	0.835294	-3.6829	1.607944
H	-1.02253	-1.09413	-1.31843
H	-0.68343	-2.80278	-1.58878
H	-2.86543	4.558503	0.248699

	Coordinates (Å)		
Conformer 2*R*-**2**-6	X	Y	Z
C	5.561651	1.087697	-0.09025
C	4.708311	1.986305	0.44192
C	3.212574	1.781528	0.386975
C	2.802765	0.31162	0.178771
C	3.604804	-0.28523	-0.99912
C	5.112288	-0.13003	-0.77473
C	1.255717	0.068538	0.02766
C	1.070172	-1.49998	0.005474
C	1.755633	-2.02787	-1.256
C	3.248607	-1.75183	-1.30646
C	0.540293	0.657	1.272998
C	-0.86896	0.124499	1.636487
C	-0.84699	-1.44147	1.698982
C	-0.34236	-2.08006	0.336642
C	-2.27054	-1.93068	2.013453
O	5.909068	-0.97359	-1.17663
O	1.194089	-2.62701	-2.15926
C	5.187174	3.244788	1.10582
N	-1.9519	0.708247	0.782716
C	-3.24054	-1.44857	0.939002
C	-2.07268	2.170403	0.983451
C	-2.17047	2.96899	-0.32646
C	-3.46371	2.762198	-1.11137
C	-3.91185	1.303354	-1.31307
C	-4.36696	0.662345	0.016903
C	-3.28478	0.098192	0.983945
H	-1.32119	2.679412	-0.95972
H	3.111729	-0.22527	1.088407
H	3.384838	0.318823	-1.89302
C	0.739618	0.74497	-1.2637
C	-5.06221	1.250365	-2.33126
C	0.029612	-1.86228	2.905368
C	-0.18204	-3.61636	0.477573
C	-1.37898	-1.83478	-0.80288
C	-2.83321	-1.9991	-0.41727
O	-3.65065	-2.50244	-1.1753
H	1.716738	-1.85916	0.817127
H	-1.053	0.450705	2.678406

	Coordinates (Å)		
	X	Y	Z
O	-2.10885	4.373111	-0.03981
H	-4.25544	-1.80524	1.14039
H	-3.628	0.323464	2.01351
H	6.637656	1.231063	-0.03492
H	2.822677	2.41622	-0.42205
H	2.773411	2.176486	1.309301
H	3.634225	-2.05641	-2.28036
H	3.725766	-2.39524	-0.55459
H	1.168908	0.468336	2.150624
H	0.511987	1.744991	1.172295
H	-2.29558	-3.02012	2.111401
H	-2.58459	-1.52749	2.985102
H	6.272748	3.350266	1.049517
H	4.726336	4.124398	0.639013
H	4.887399	3.261031	2.161154
H	-1.20111	2.543353	1.528687
H	-2.93729	2.413177	1.621017
H	-4.26212	3.305287	-0.5859
H	-3.34456	3.25456	-2.08377
H	-3.07484	0.727367	-1.7266
H	-5.0476	-0.16412	-0.21265
H	-4.97427	1.402543	0.552
H	1.080588	0.242824	-2.1733
H	-0.34754	0.771065	-1.28403
H	1.078285	1.784139	-1.31727
H	-5.36108	0.216354	-2.53097
H	-4.77118	1.708433	-3.28242
H	-5.94259	1.788511	-1.95876
H	-0.09372	-2.92673	3.118899
H	1.097273	-1.67584	2.773591
H	-0.28771	-1.31606	3.799692
H	-1.12122	-4.10006	0.759713
H	0.12523	-4.04323	-0.47823
H	0.570191	-3.8865	1.223837
H	-1.16355	-2.47348	-1.65921
H	-1.3153	-0.79999	-1.14407
H	-1.22609	4.566576	0.303929

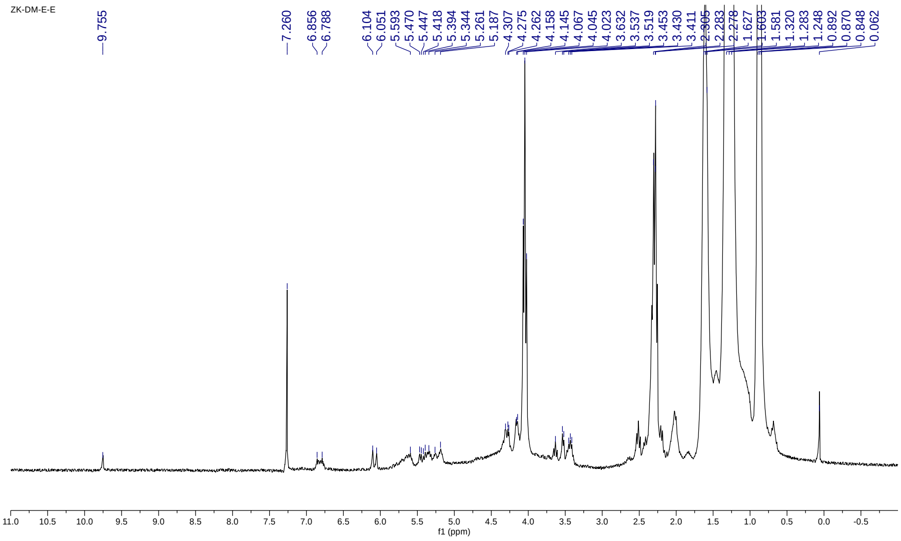

Figure S1. ^{1}H-NMR spectrum of fraction EF at 600 MHz in C_5D_5N

Figure S2. ^{1}H-NMR spectrum of norzoabenzaldehyde (**1**) at 600 MHz in C_5D_5N

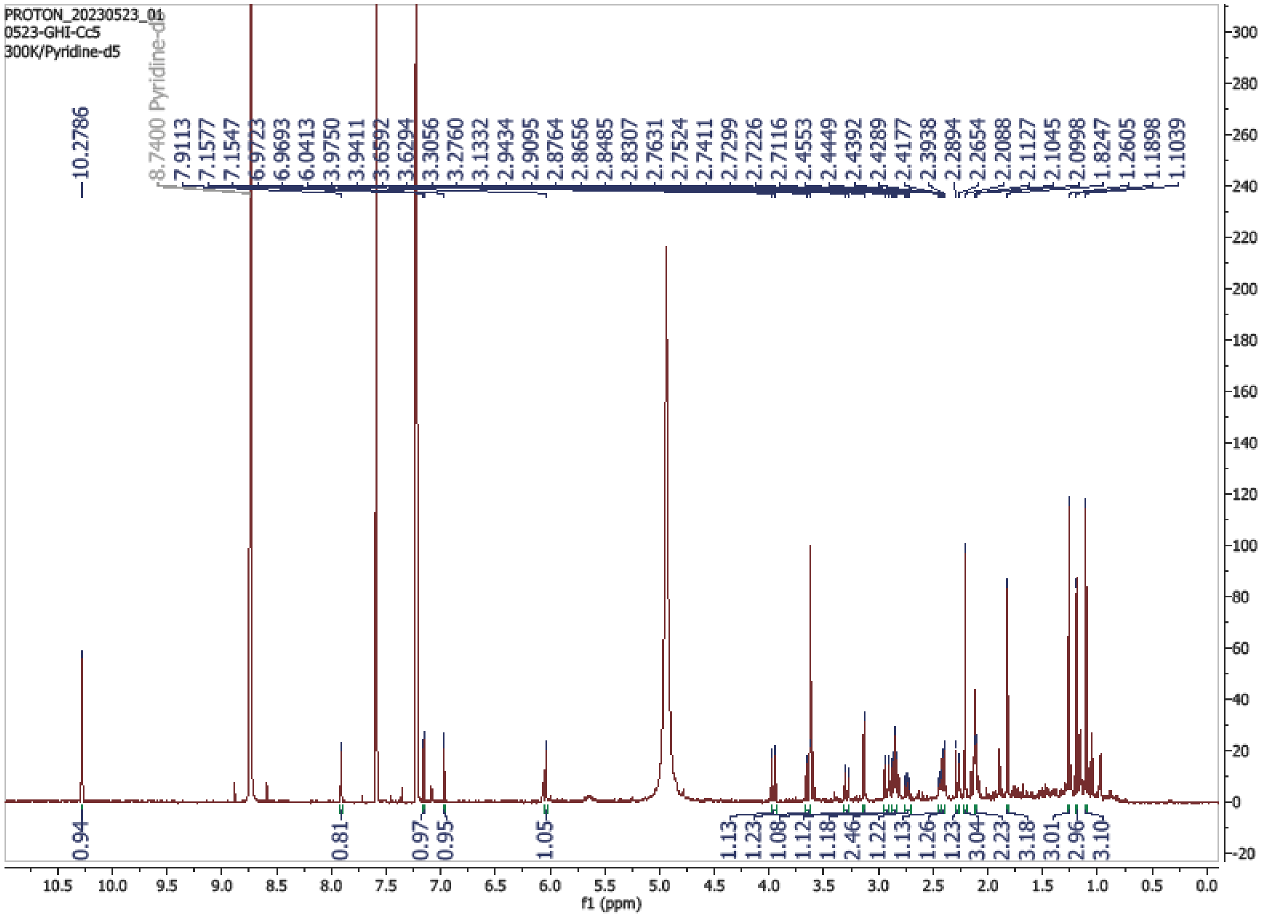

Figure S3. ^{1}H-NMR spectrum of norzoabenzaldehyde (6.94-7.20 ppm) at 600 MHz in C_5D_5N

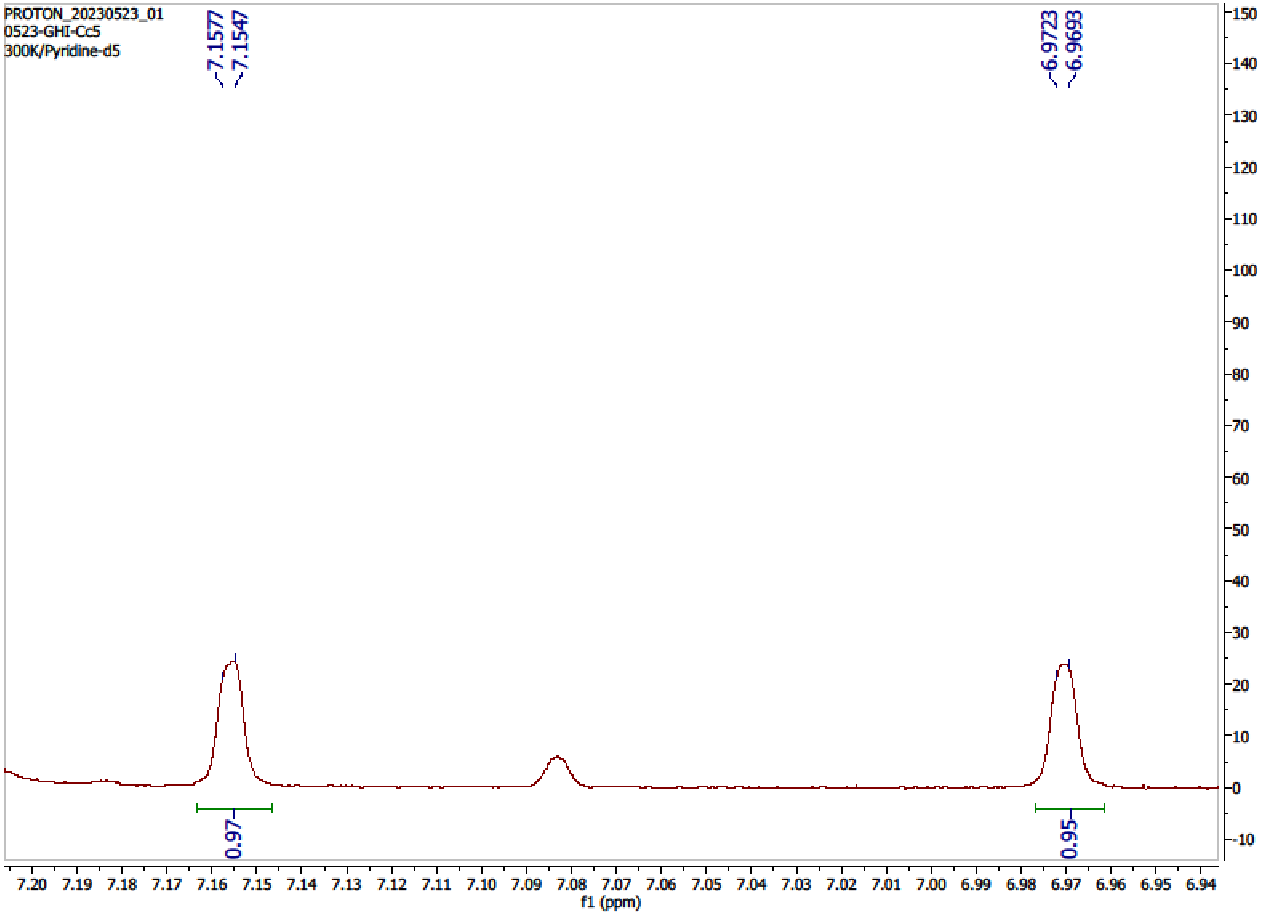

Figure S4. ^{1}H-NMR spectrum of norzoabenzaldehyde (3-4 ppm) at 600 MHz in C_5D_5N

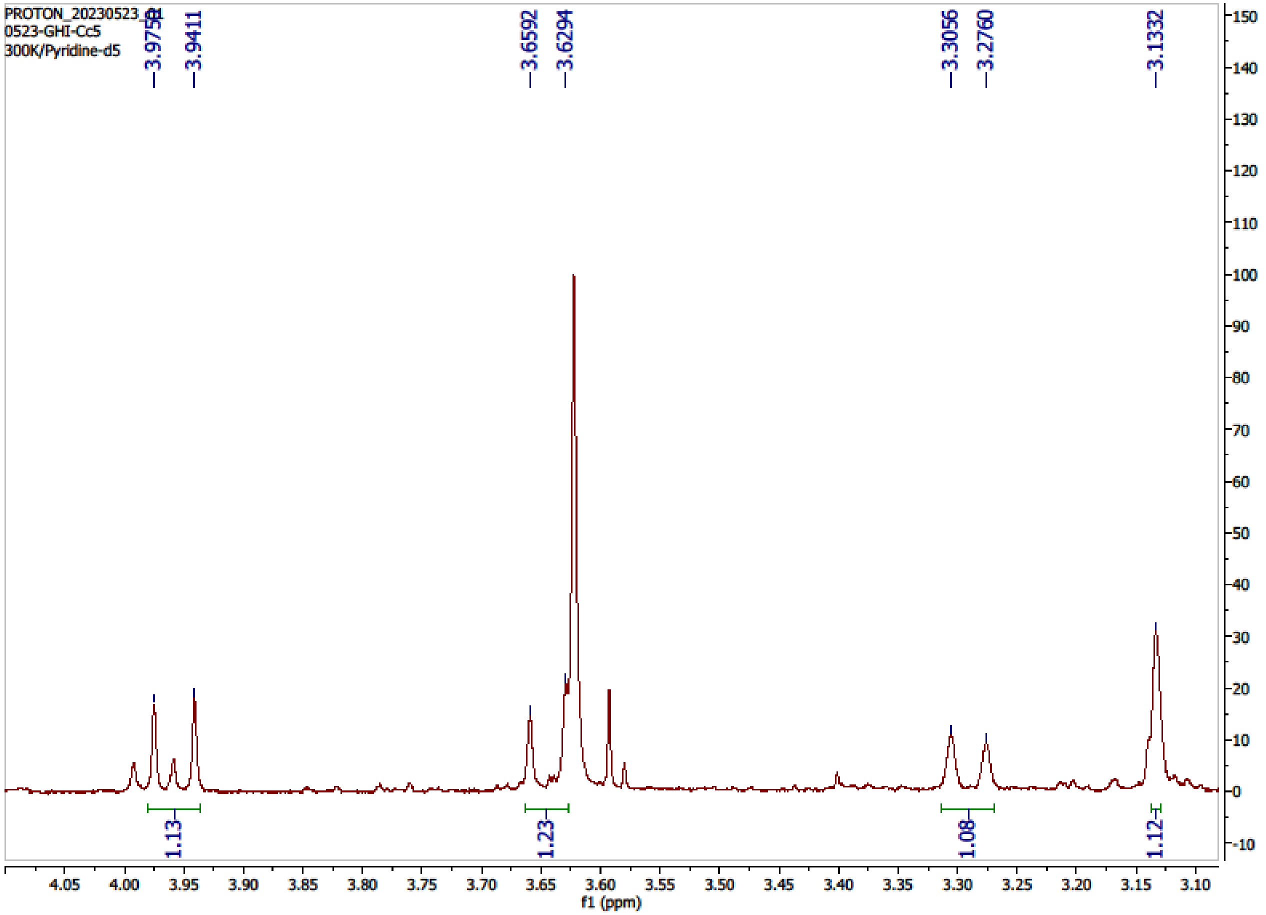

253 **Figure S5**. ^{1}H-NMR spectrum of norzoabenzaldehyde (1-3 ppm) at 600 MHz in C_5D_5N

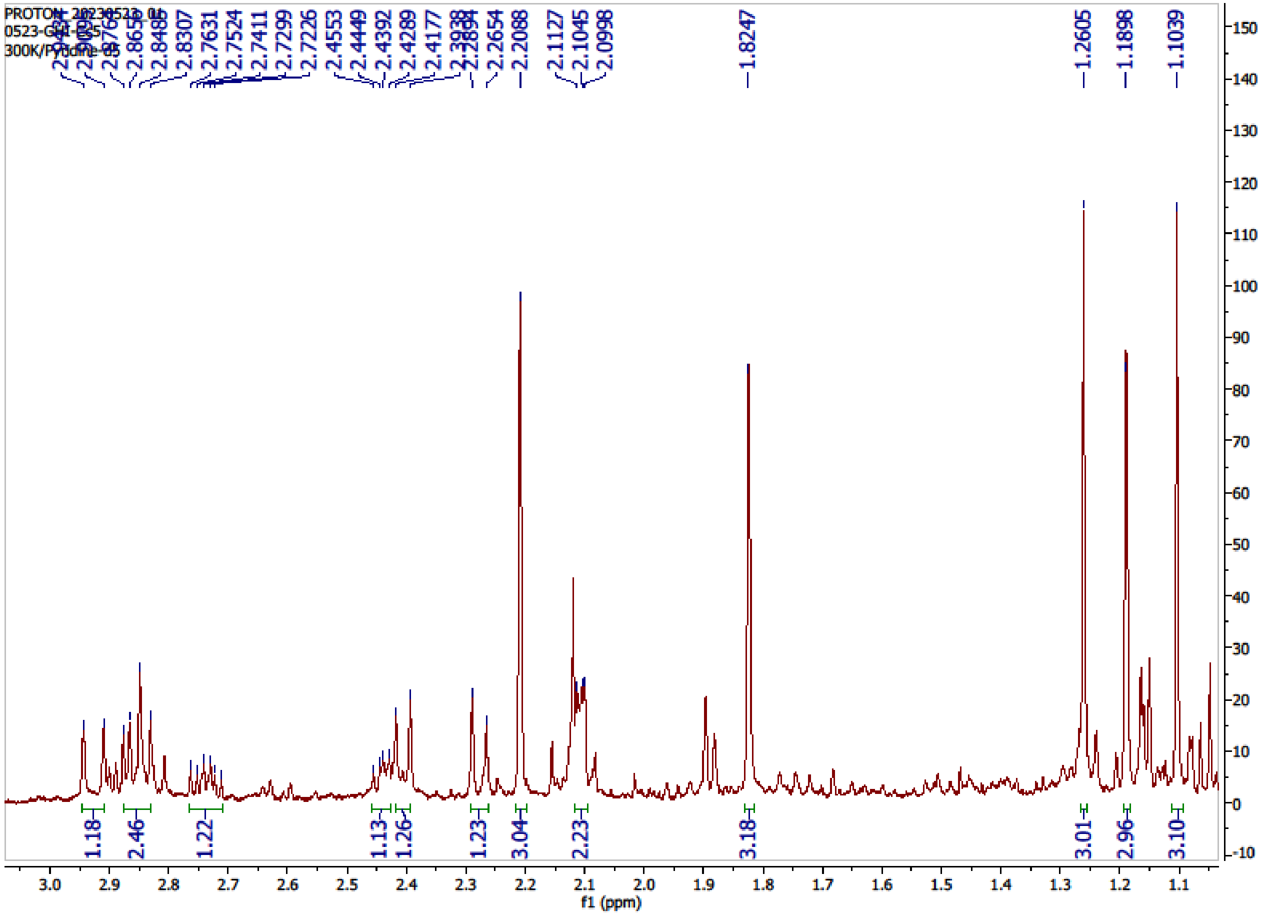

Figure S6. ^{13}C-NMR spectrum of norzoabenzaldehyde (**1**) at 150 MHz in C_5D_5N

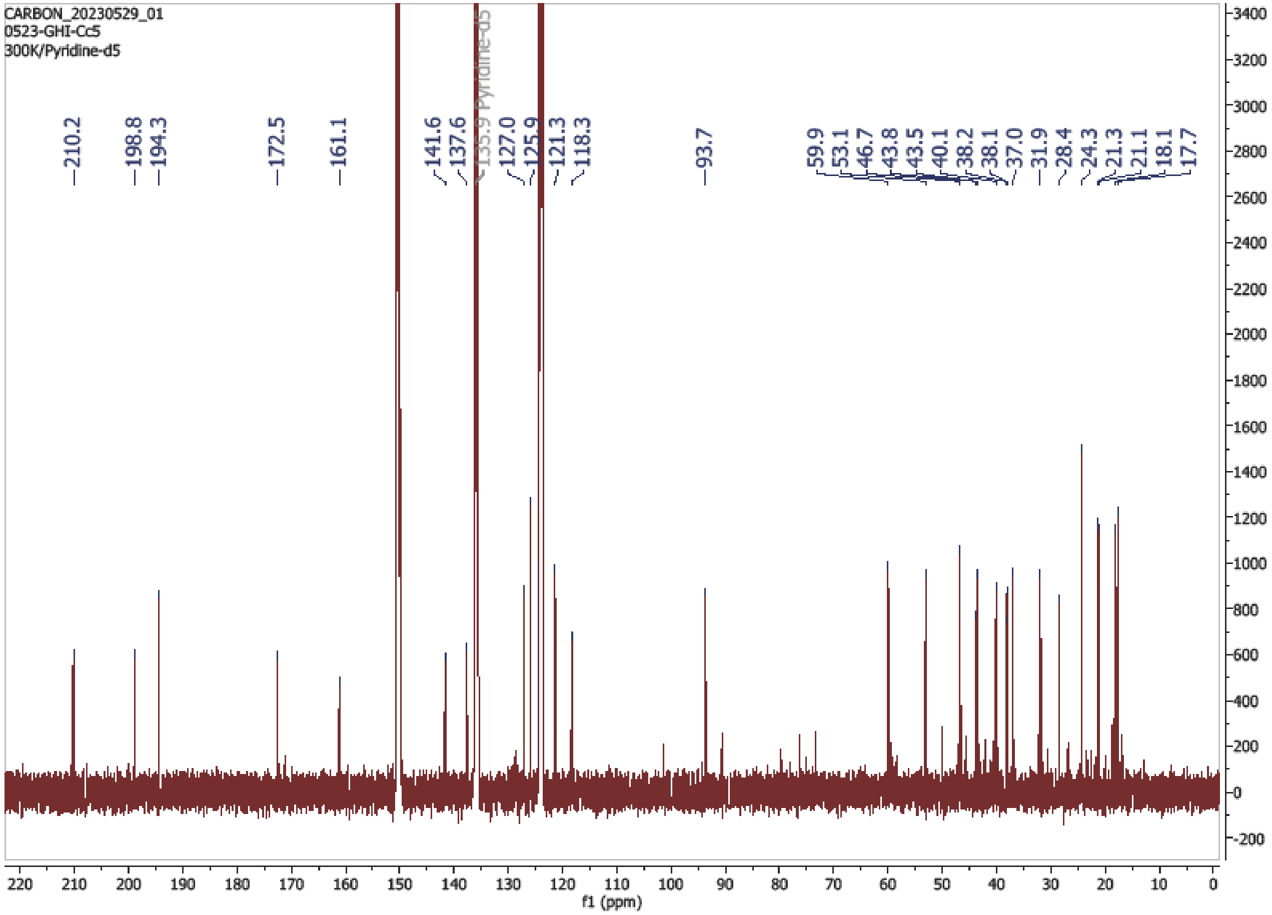

Figure S7. ^{13}C-NMR spectrum of norzoabenzaldehyde (115-210 ppm) at 150 MHz in C_5D_5N

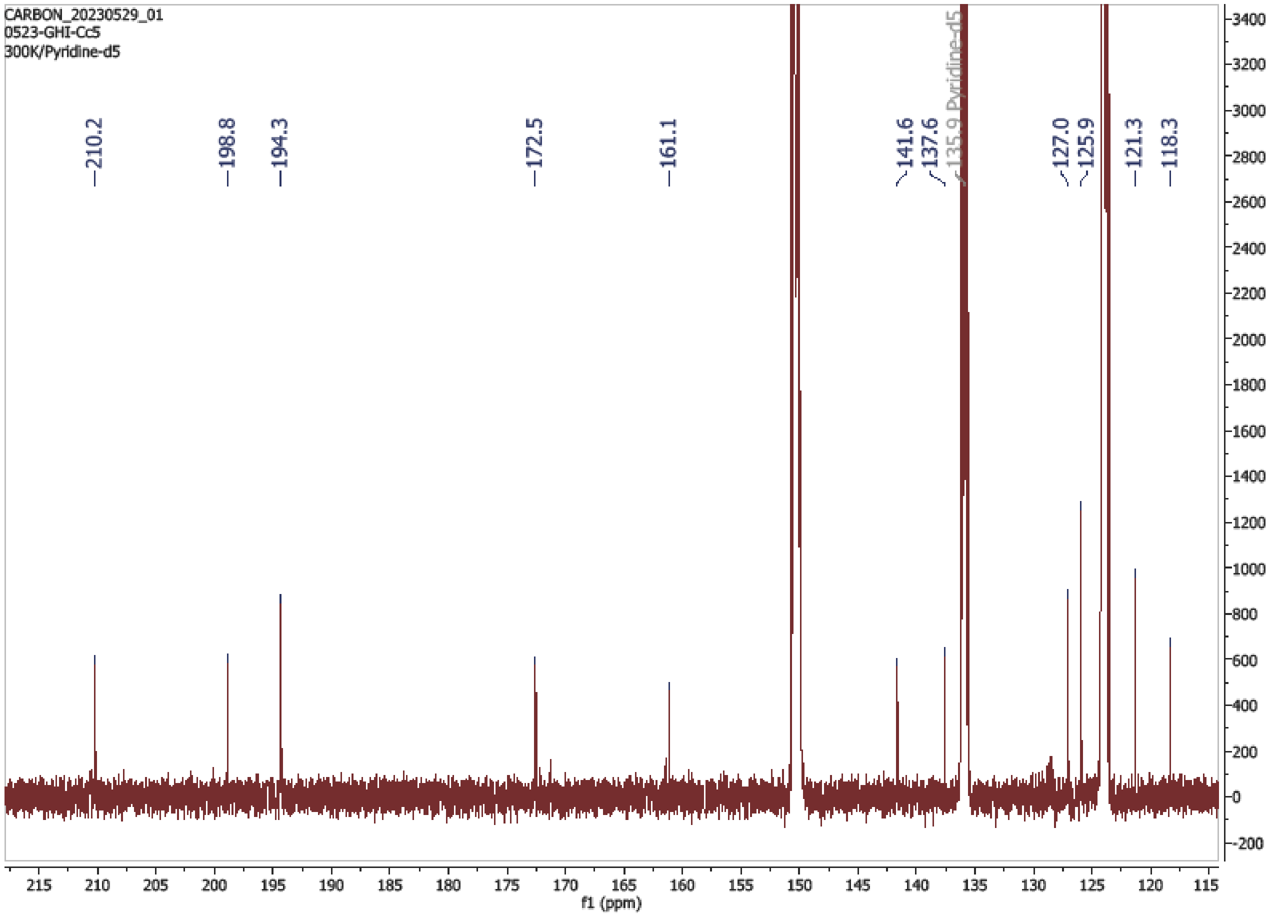

Figure S8. ^{13}C-NMR spectrum of norzoabenzaldehyde (15-95 ppm) at 150 MHz in C_5D_5N

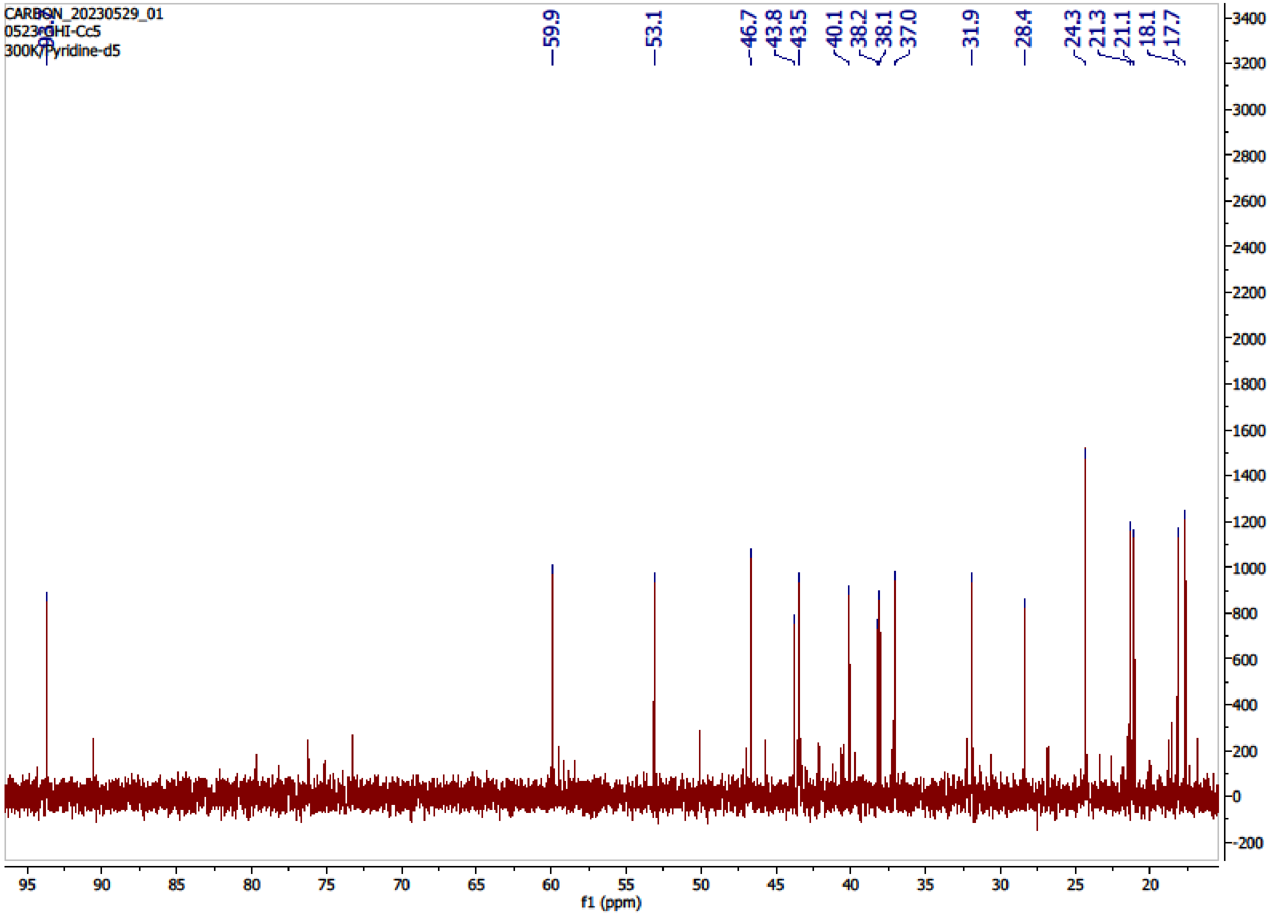

Figure S9. COSY spectrum of norzoabenzaldehyde (**1**) at 600 MHz in C_5D_5N

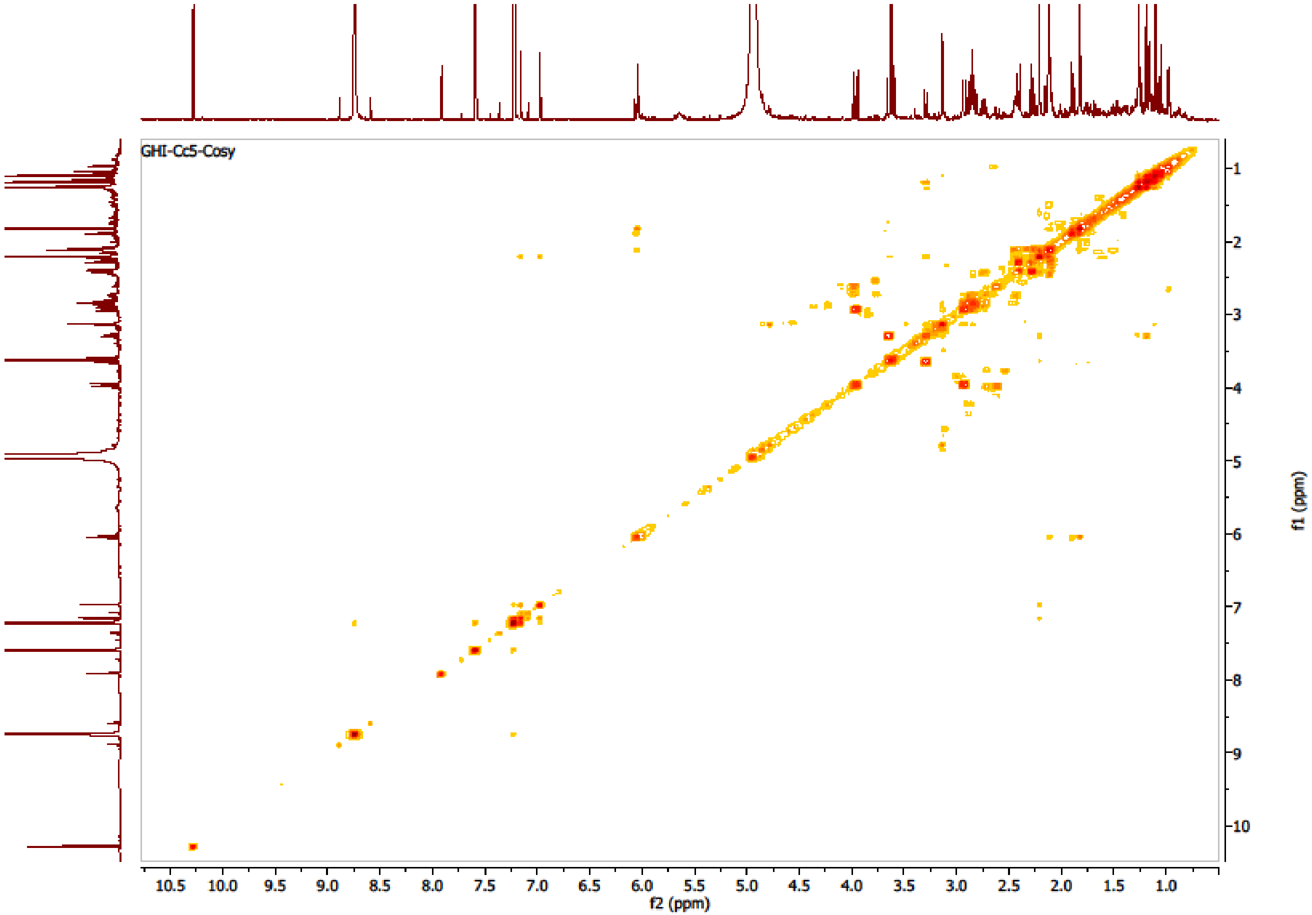

266 **Figure S10**. COSY spectrum of norzoabenzaldehyde (2.25-2.66 ppm) at 600 MHz in C_5D_5N

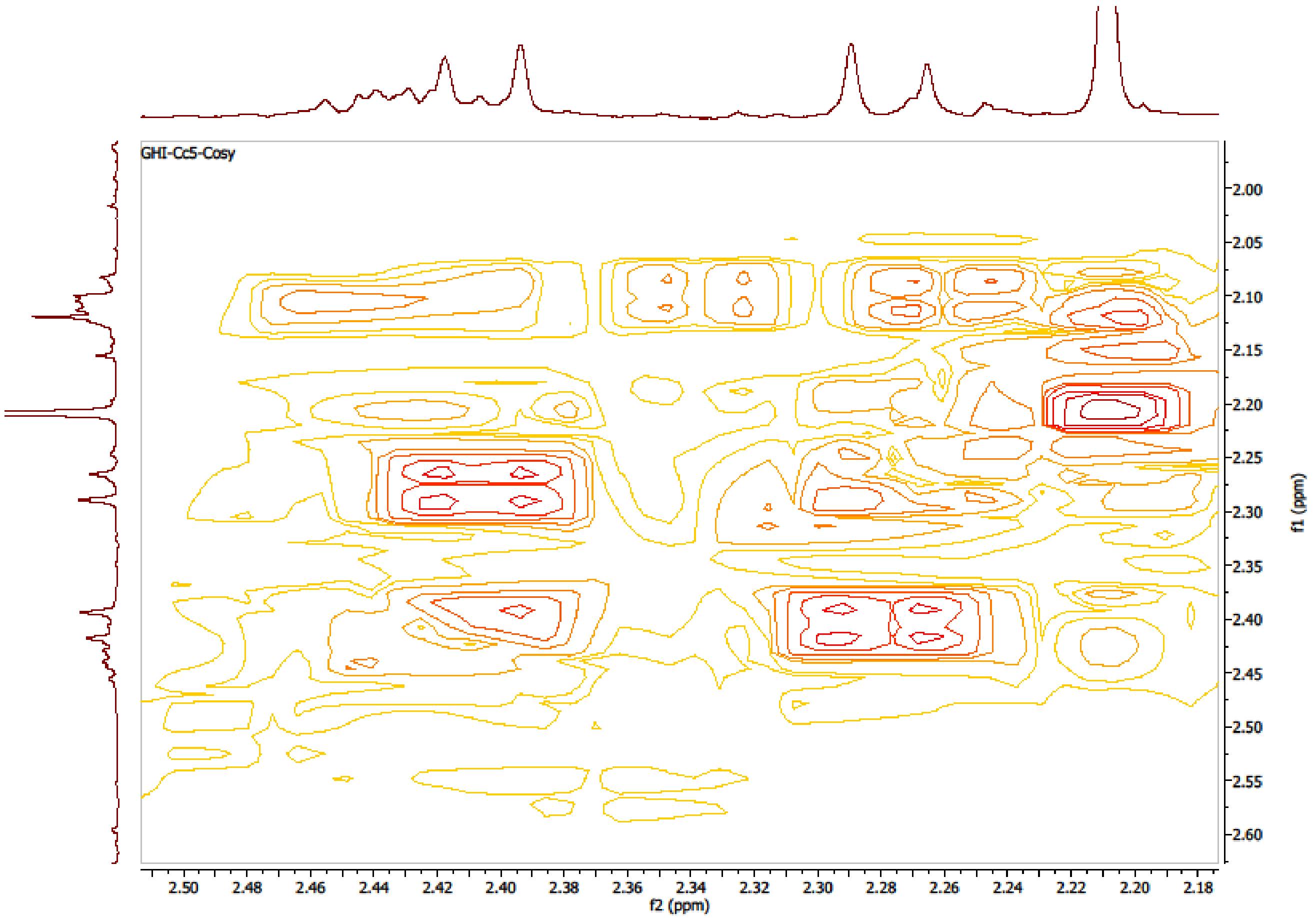

267

268

Figure S11. COSY spectrum of norzoabenzaldehyde (2.68-2.98 ppm) at 600 MHz in C_5D_5N

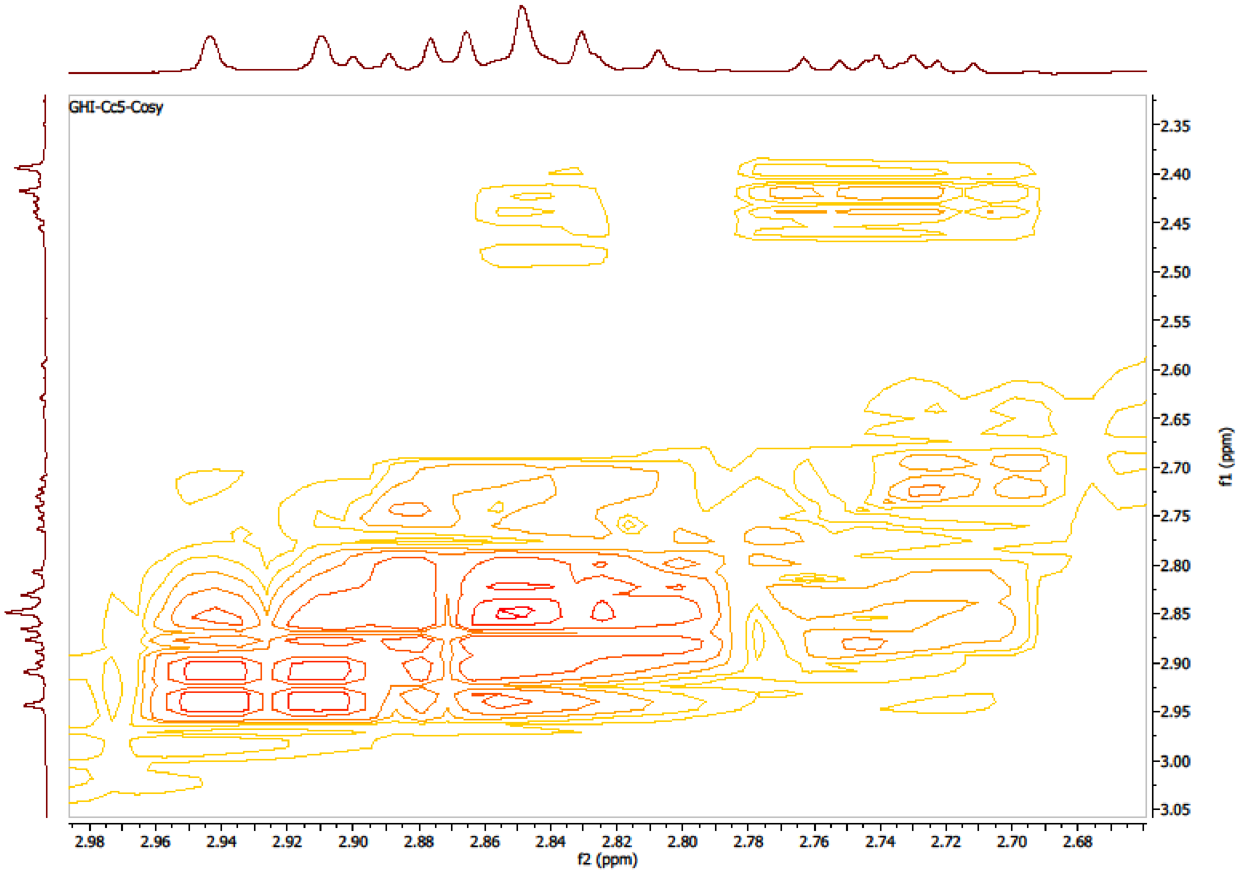

Figure S12. HSQC spectrum of norzoabenzaldehyde (**1**) at 600 and 150 MHz in C_5D_5N

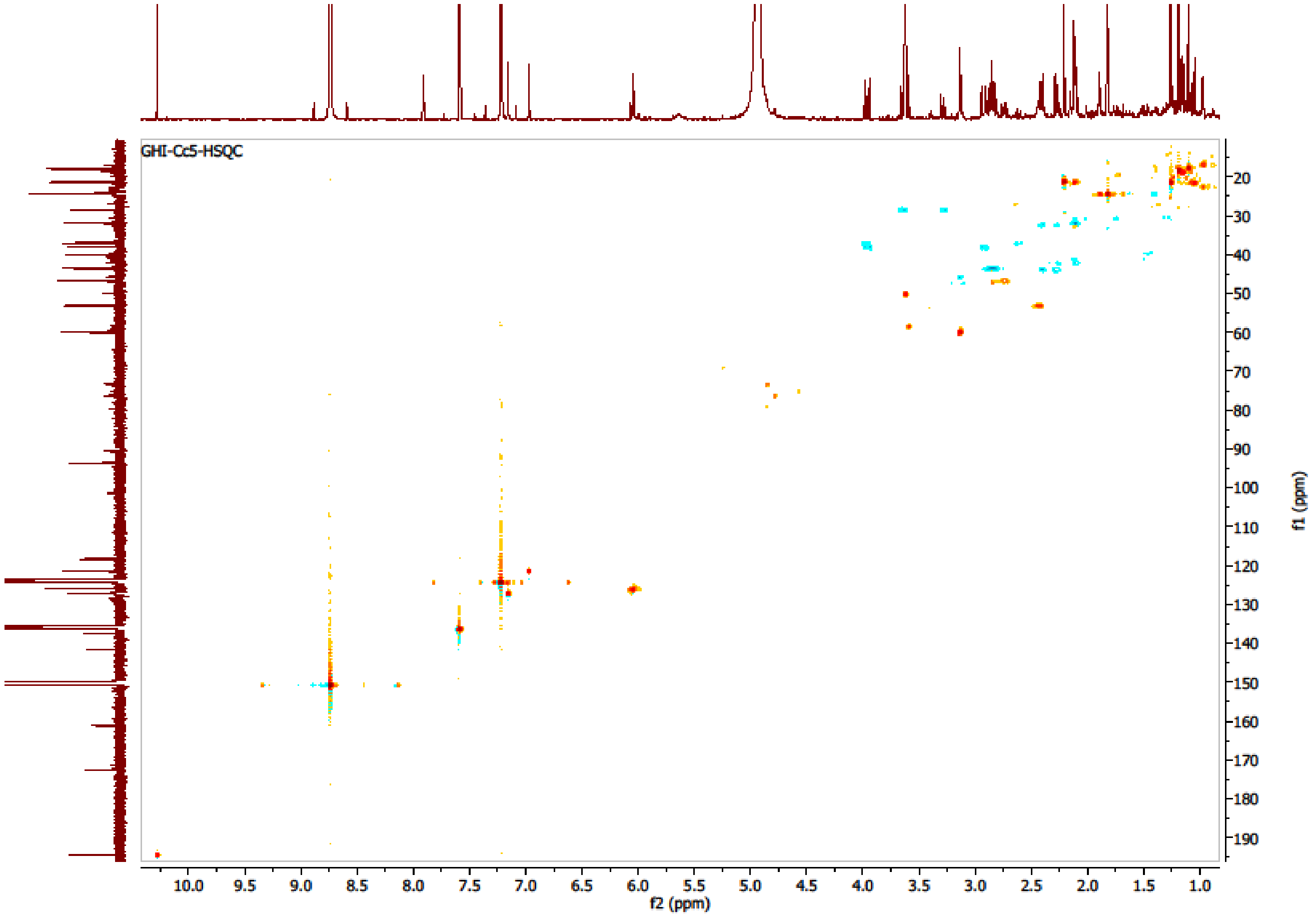

Figure S13. HSQC spectrum of norzoabenzaldehyde (9.75-10.40 ppm) at 600 and 150 MHz in C_5D_5N

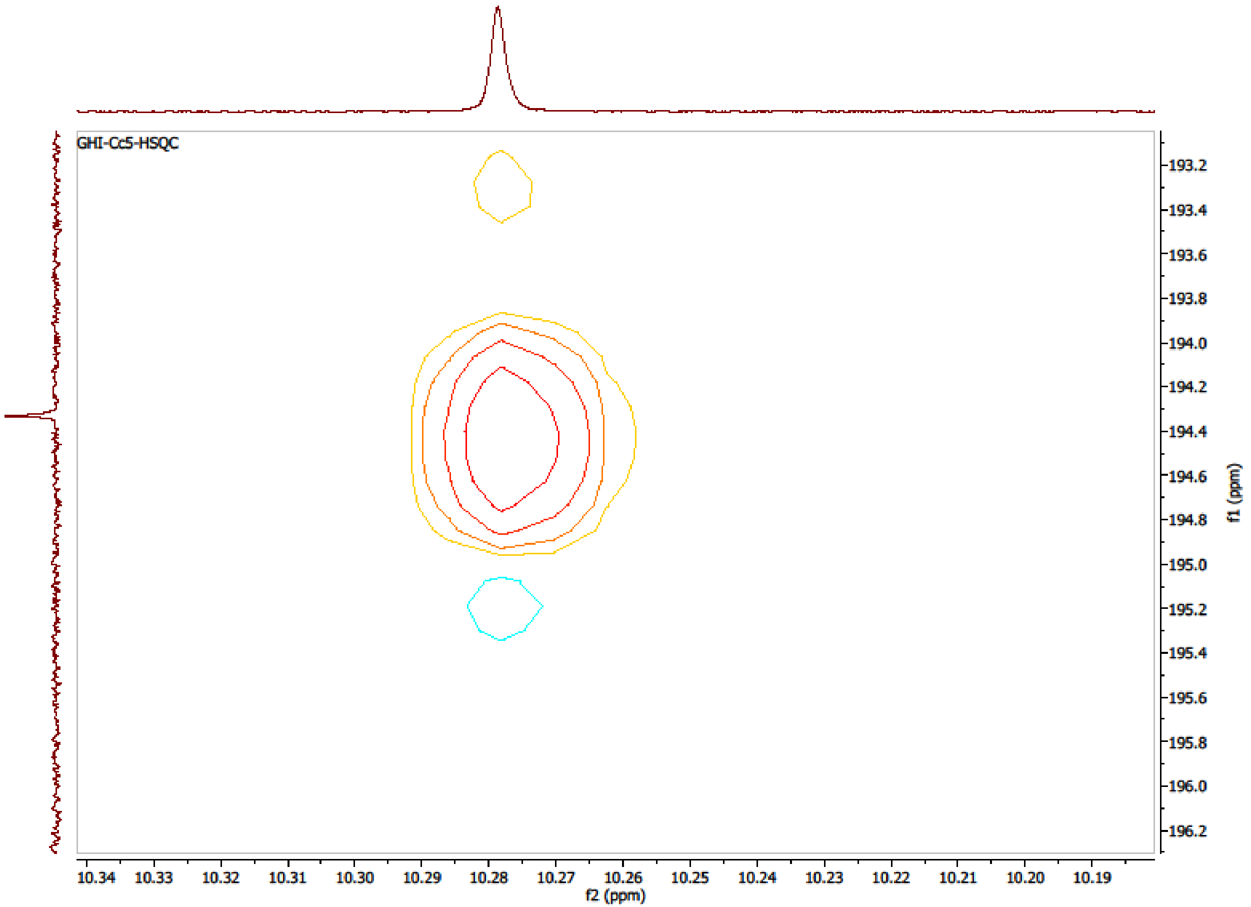

Figure S14. HSQC spectrum of norzoabenzaldehyde (6.0-7.6 ppm) at 600 and 150 MHz in C_5D_5N

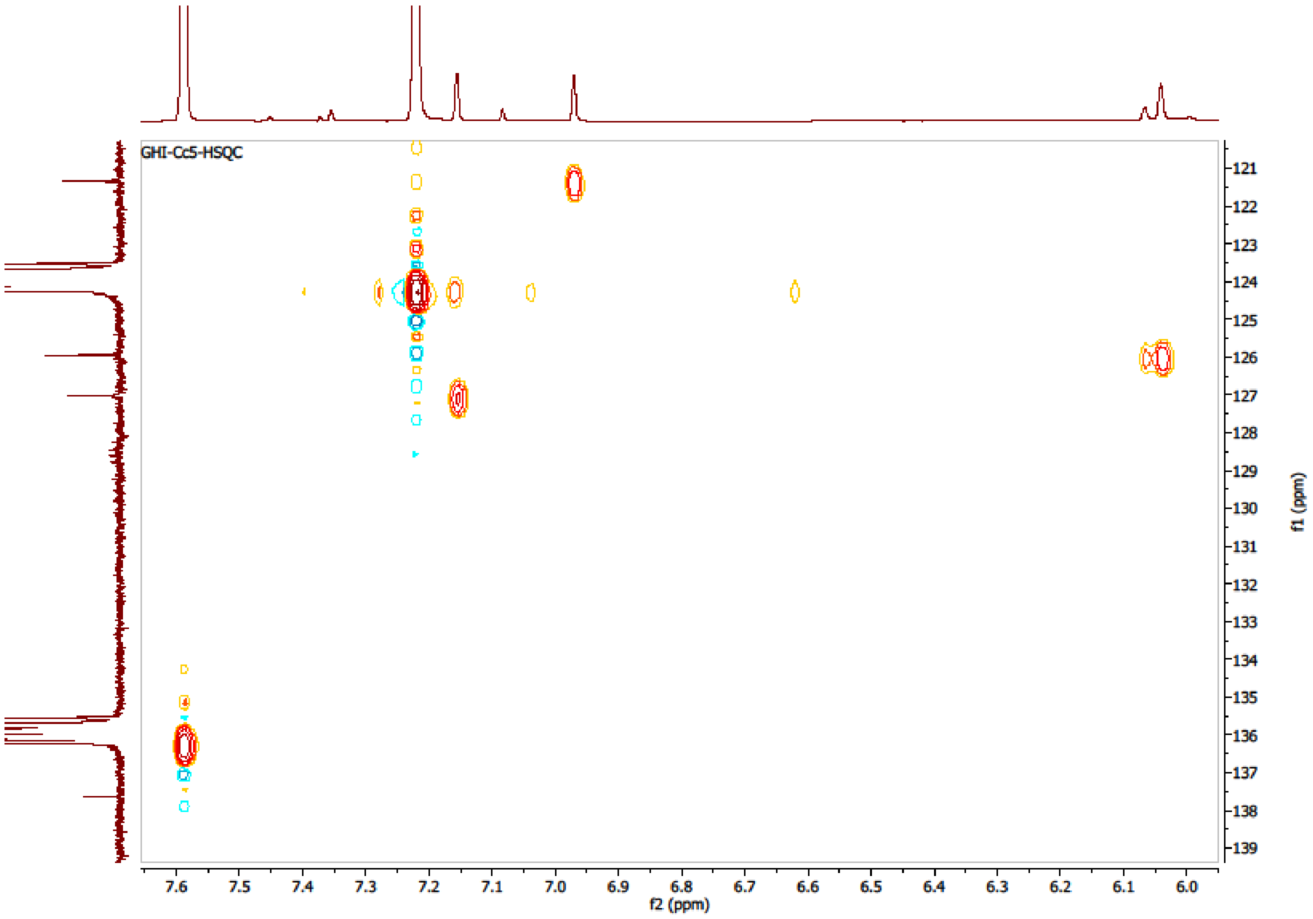

Figure S15. HSQC spectrum of norzoabenzaldehyde (0.9-4.0 ppm) at 600 and 150 MHz in C_5D_5N

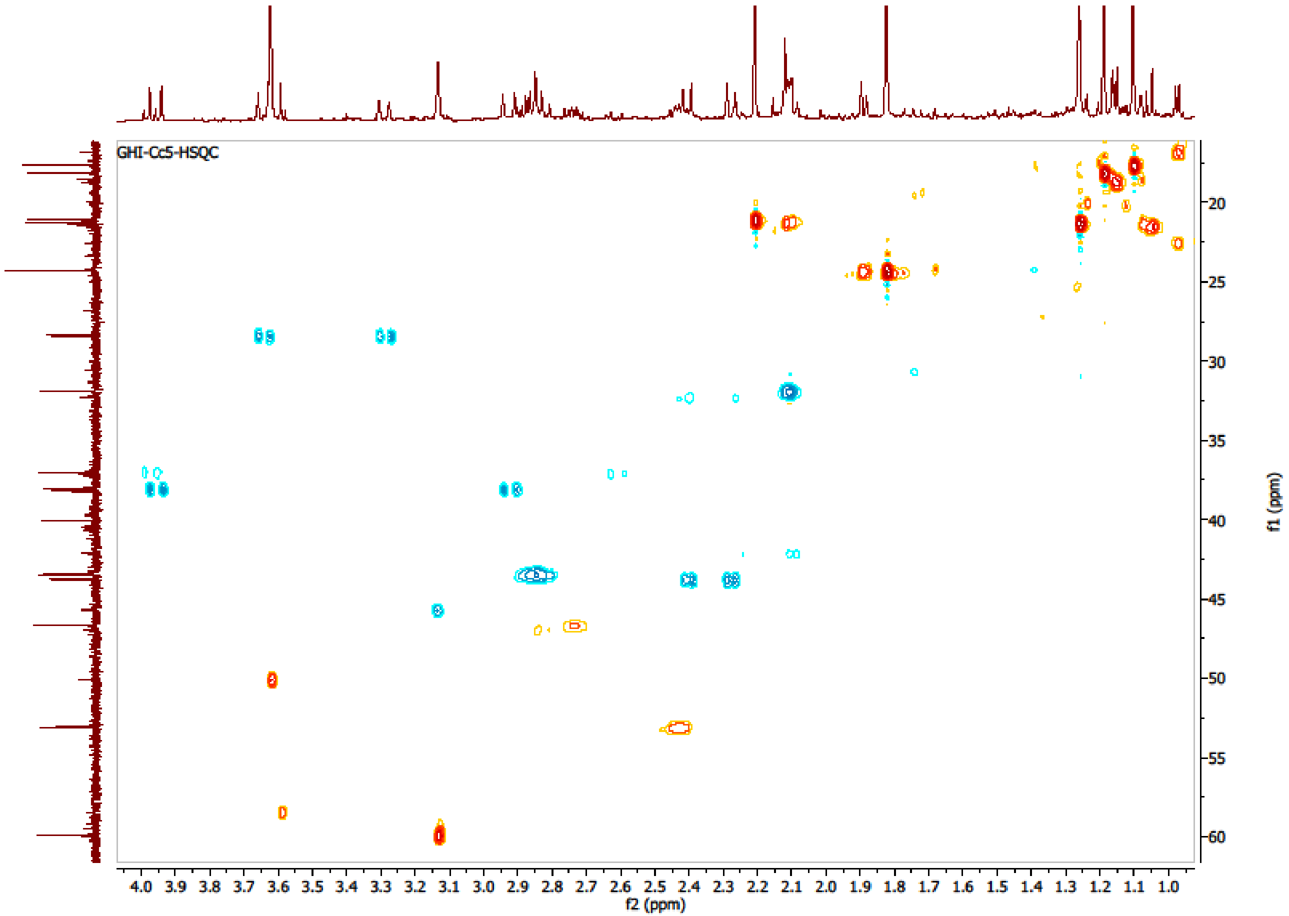

Figure S16. HMBC spectrum of norzoabenzaldehyde (**1**) at 600 and 150 MHz in C_5D_5N

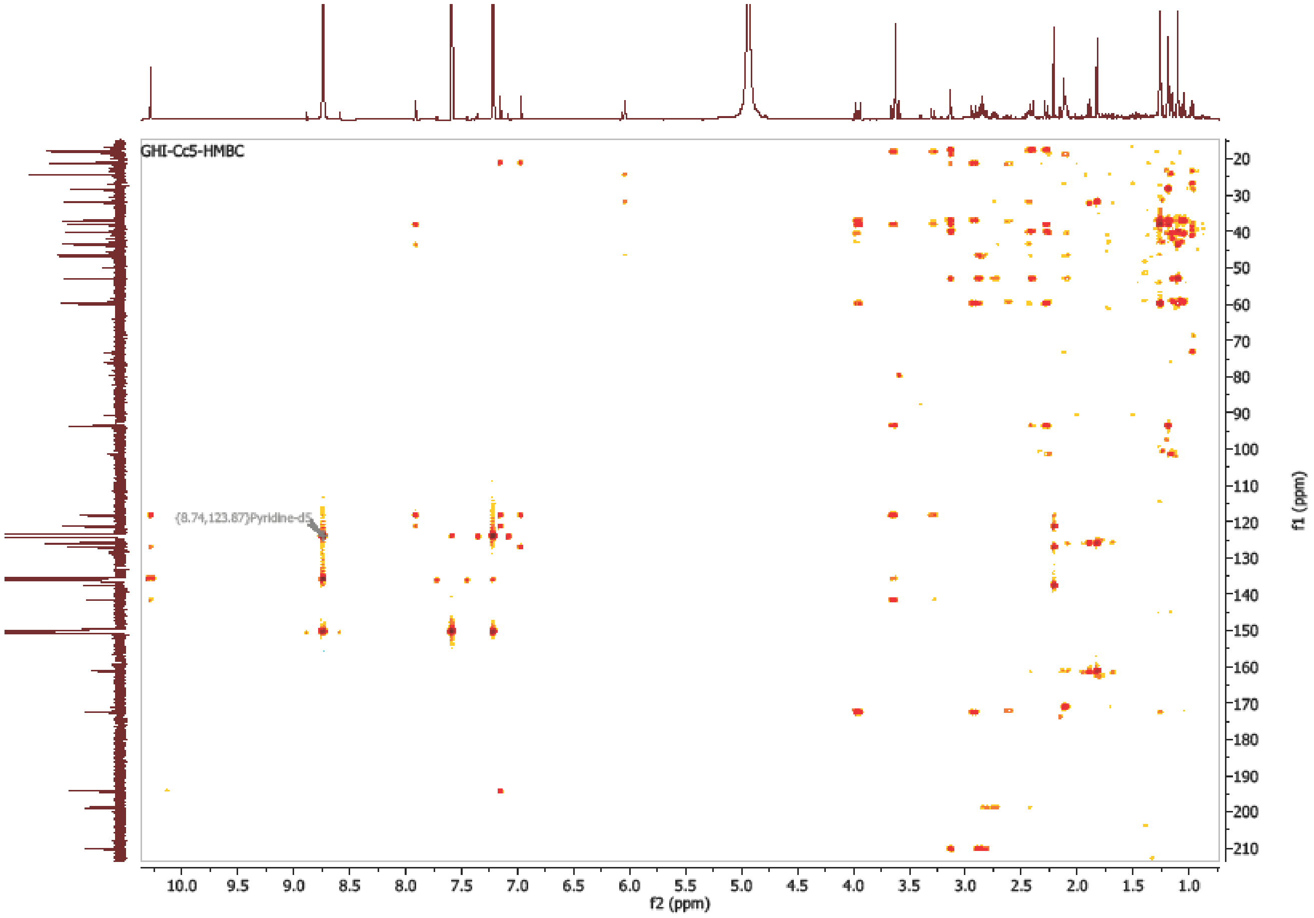

Figure S17. HMBC spectrum of norzoabenzaldehyde (10.10-10.50 ppm) at 600 and 150 MHz in C_5D_5N

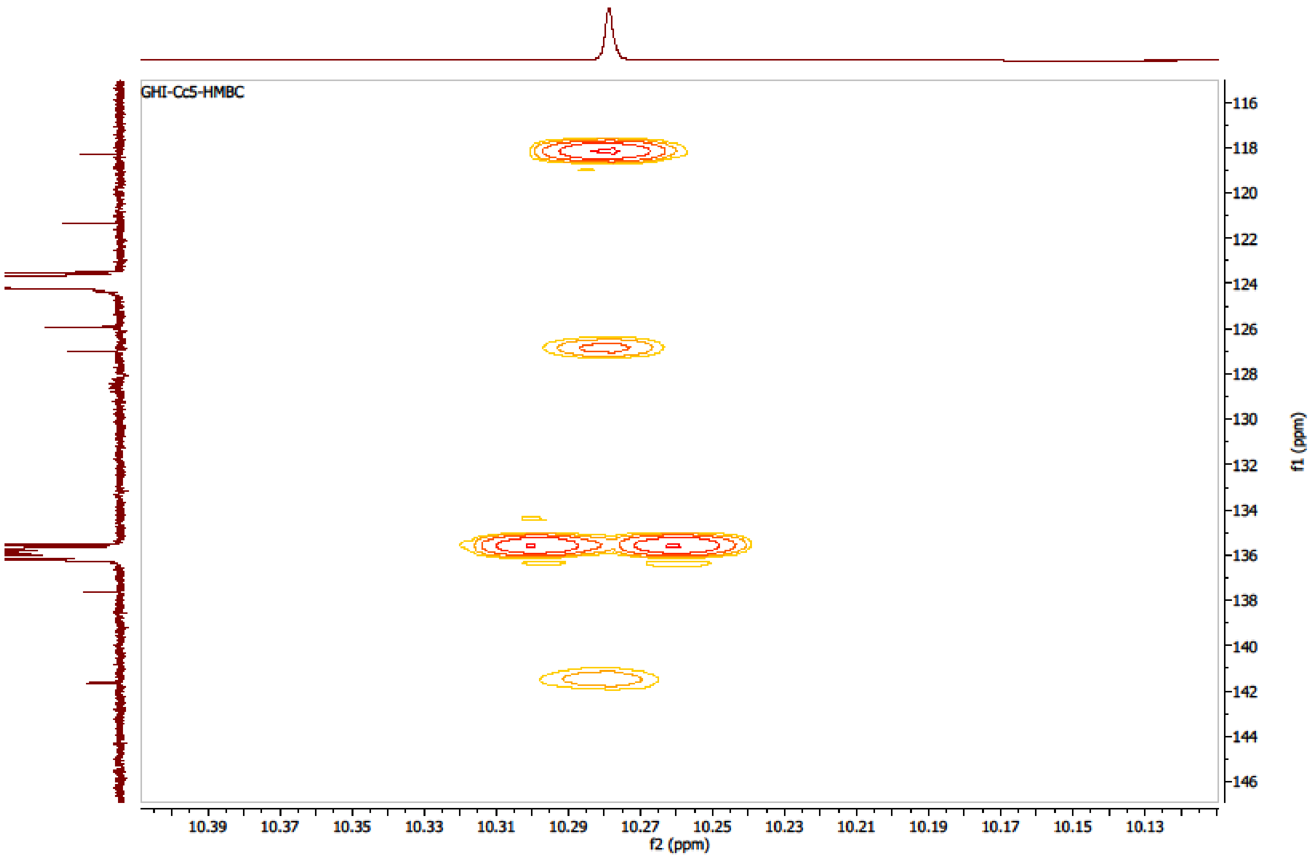

Figure S18. HMBC spectrum of norzoabenzaldehyde (6.85-7.95 ppm) at 600 and 150 MHz in C_5D_5N

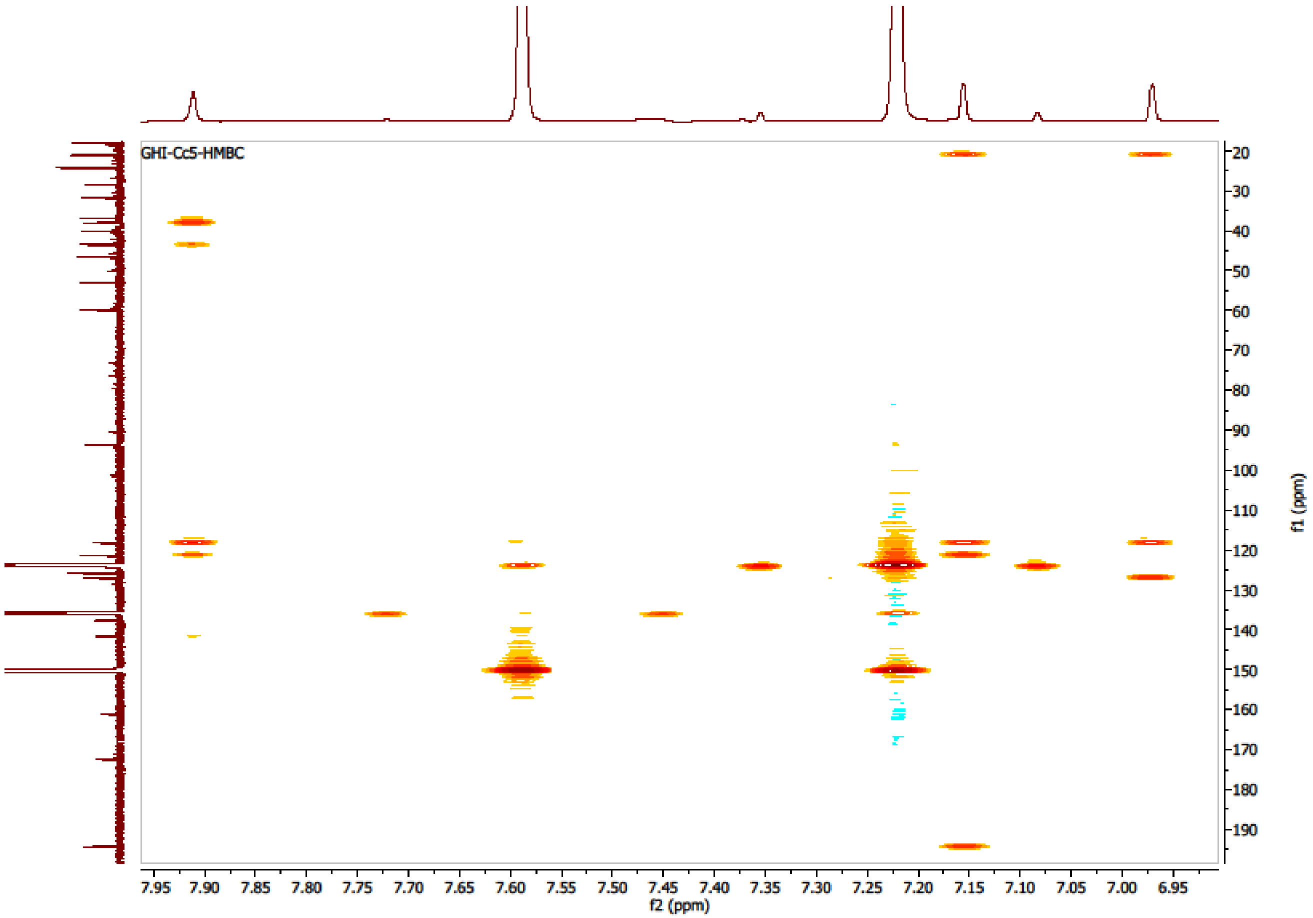

Figure S19. HMBC spectrum of norzoabenzaldehyde (2.40-4.20 ppm) at 600 and 150 MHz in C_5D_5N

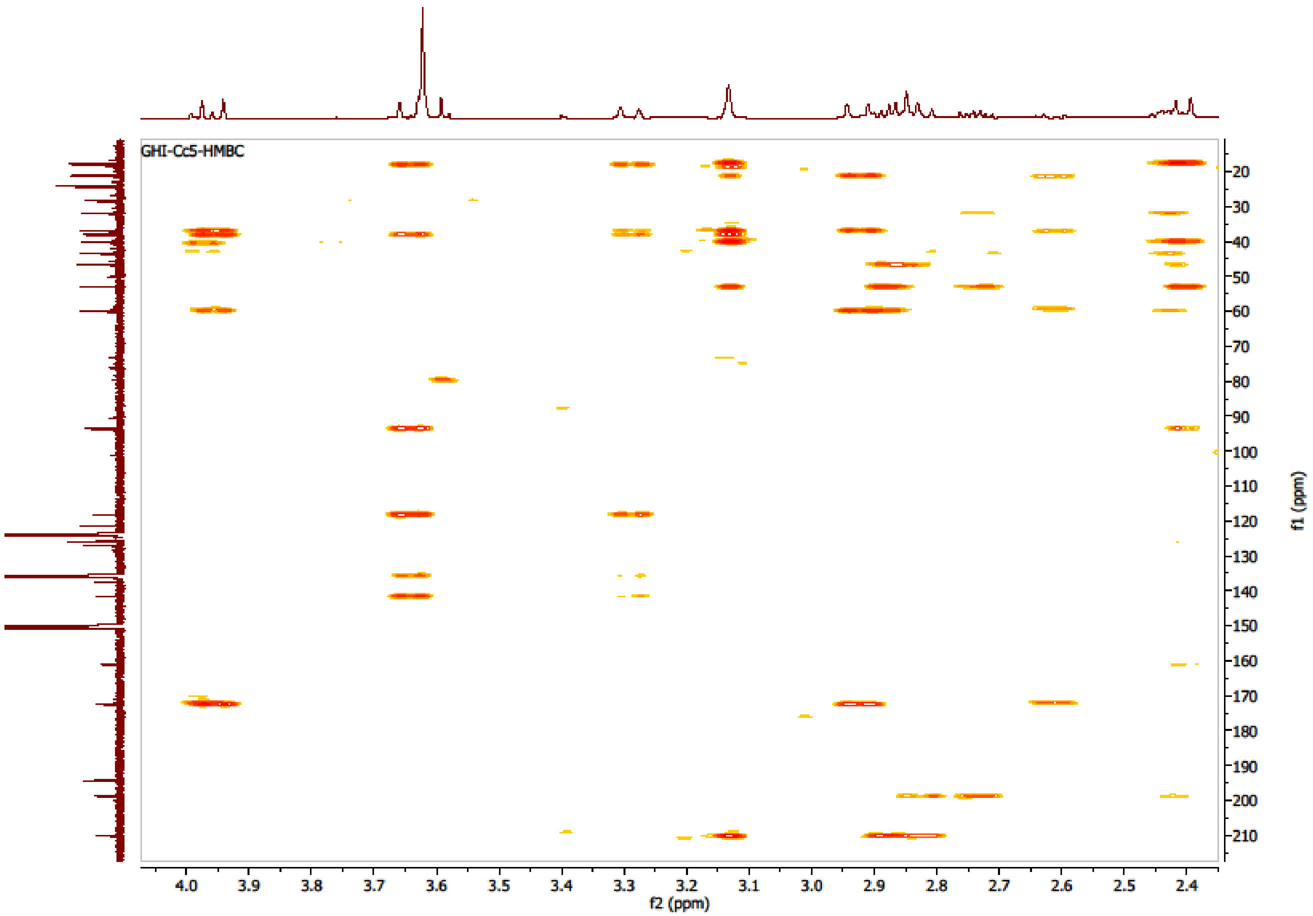

Figure S20. HMBC spectrum of norzoabenzaldehyde (0.90-2.30 ppm) at 600 and 150 MHz in C_5D_5N

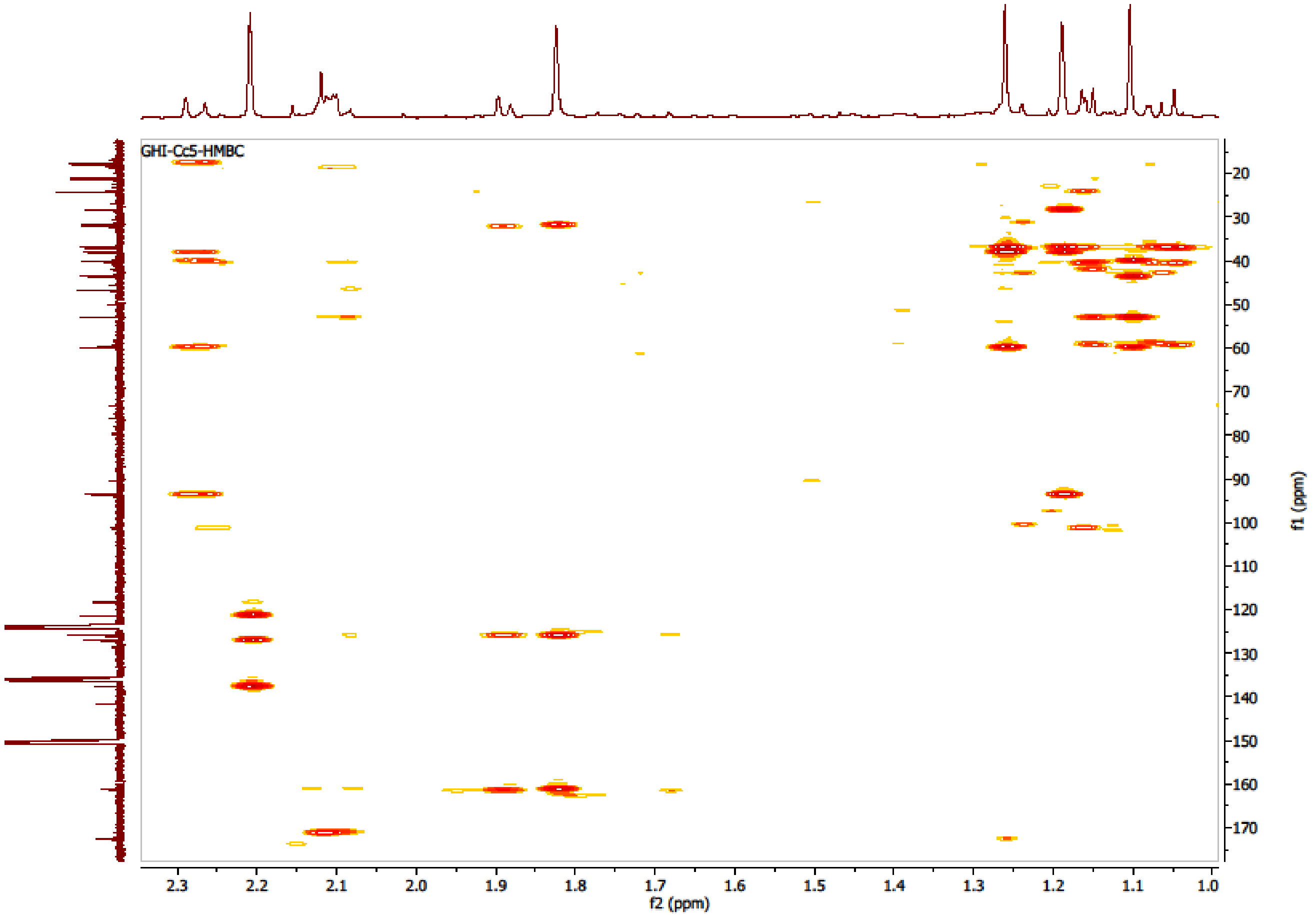

Figure S21. NOESY spectrum of norzoabenzaldehyde (**1**) at 600 MHz in C_5D_5N

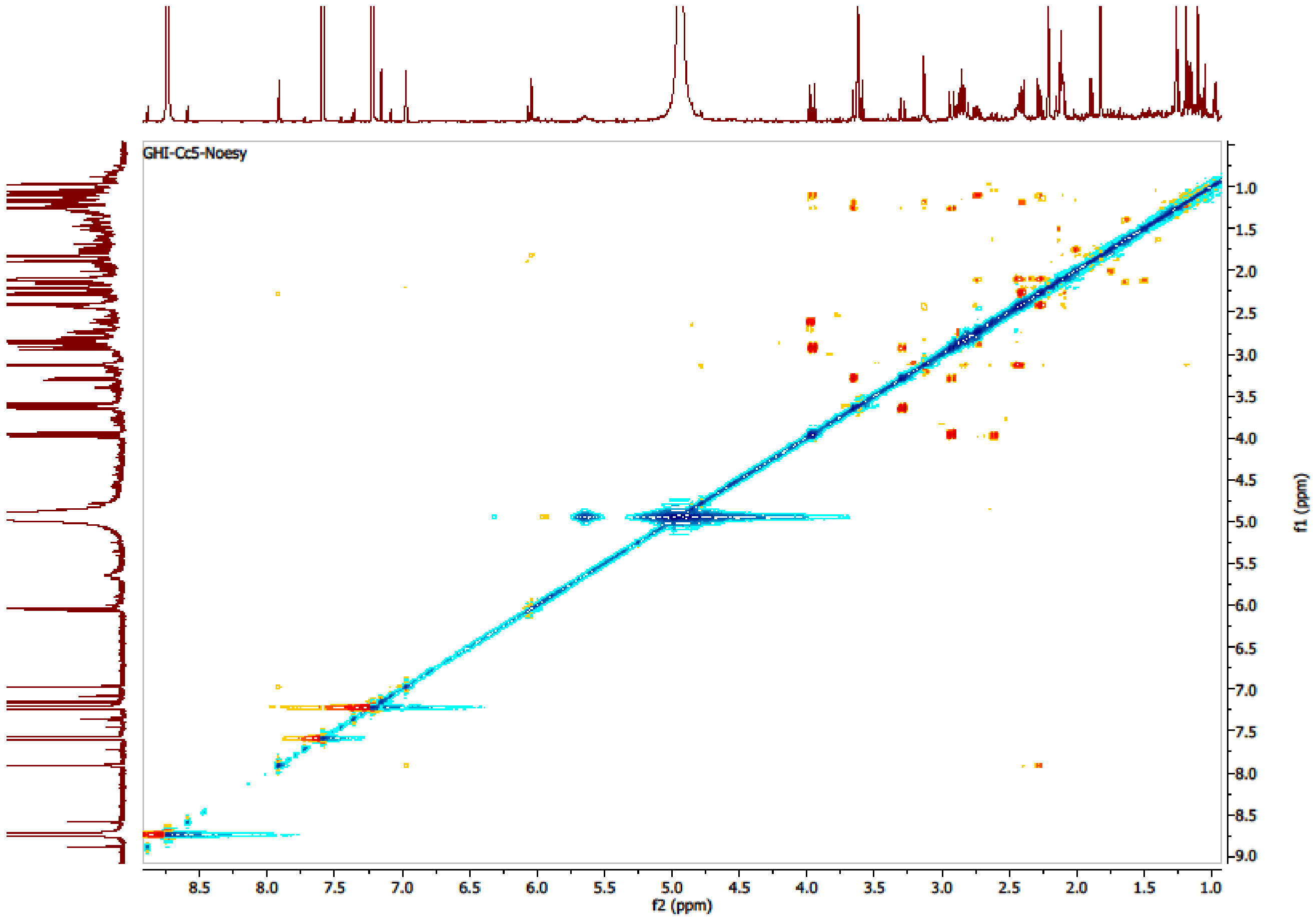

Figure S22. NOESY spectrum of norzoabenzaldehyde (7.82-8.00 ppm) at 600 MHz in C_5D_5N

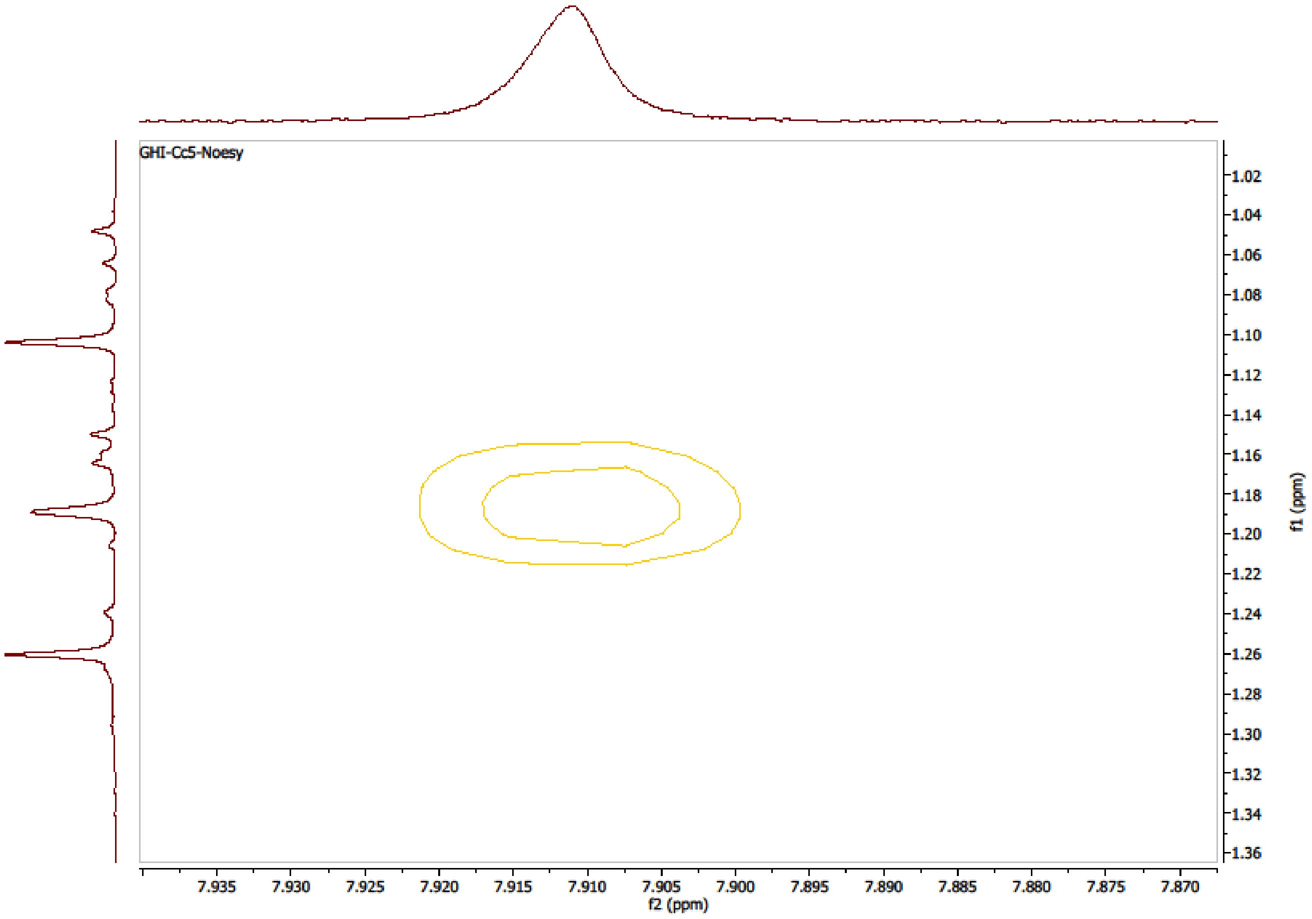

302 **Figure S23**. NOESY spectrum of norzoabenzaldehyde (3.02-3.22 ppm) at 600 MHz in C_5D_5N

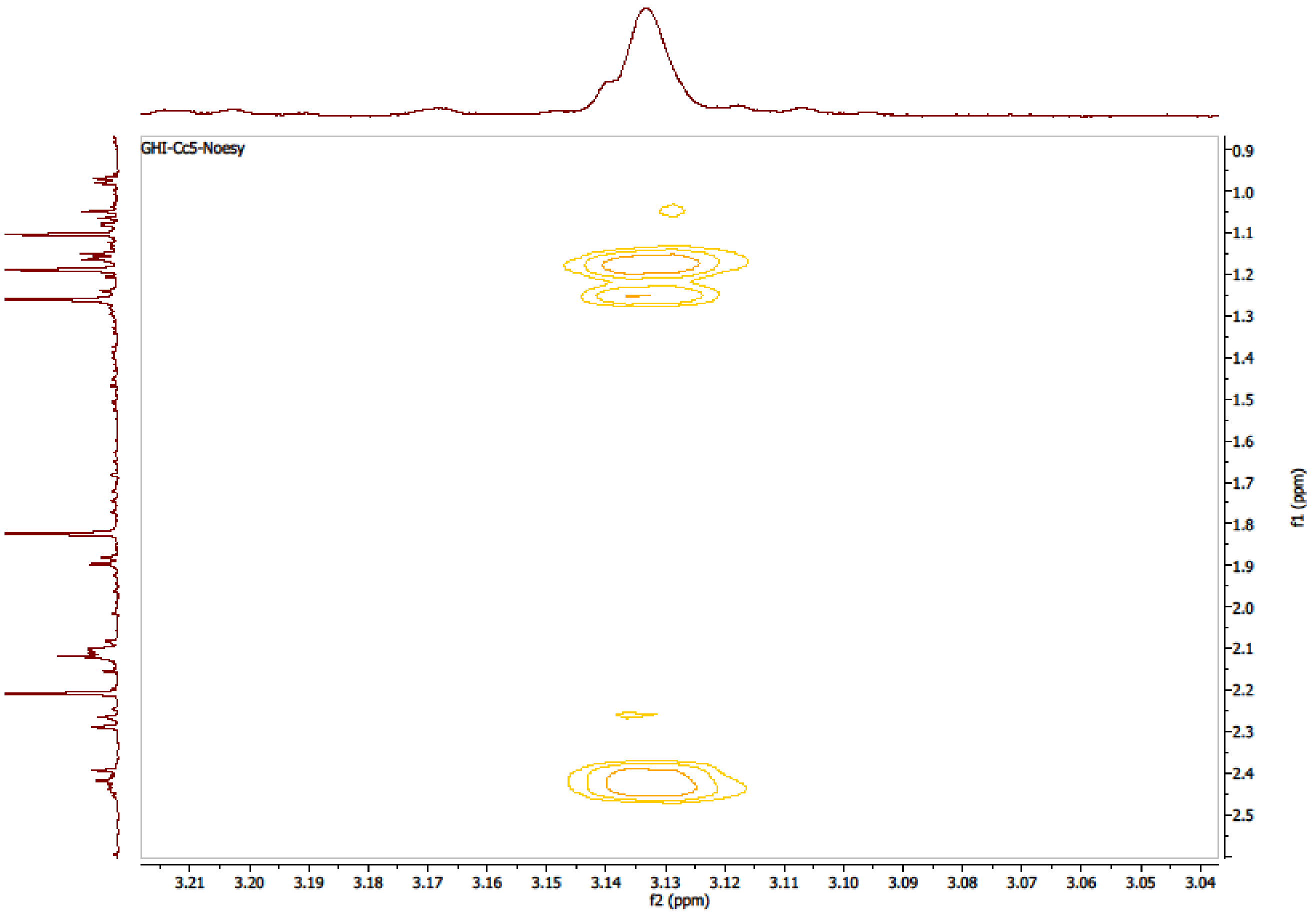

303

Figure S24. NOESY spectrum of norzoabenzaldehyde (0.99-1.18 ppm) at 600 MHz in C_5D_5N

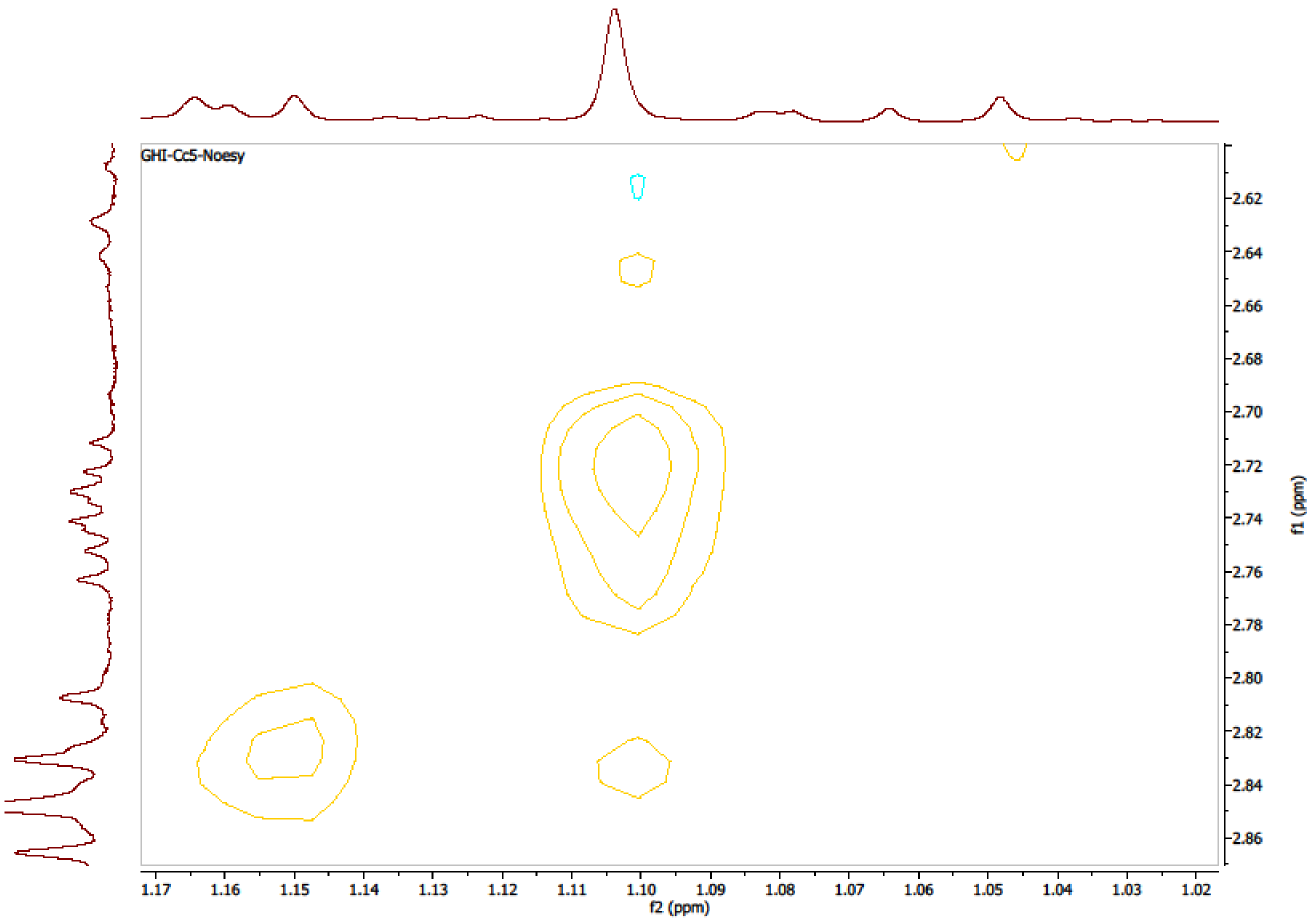

Figure S25. MS spectrum of norzoabenzaldehyde (**1**)

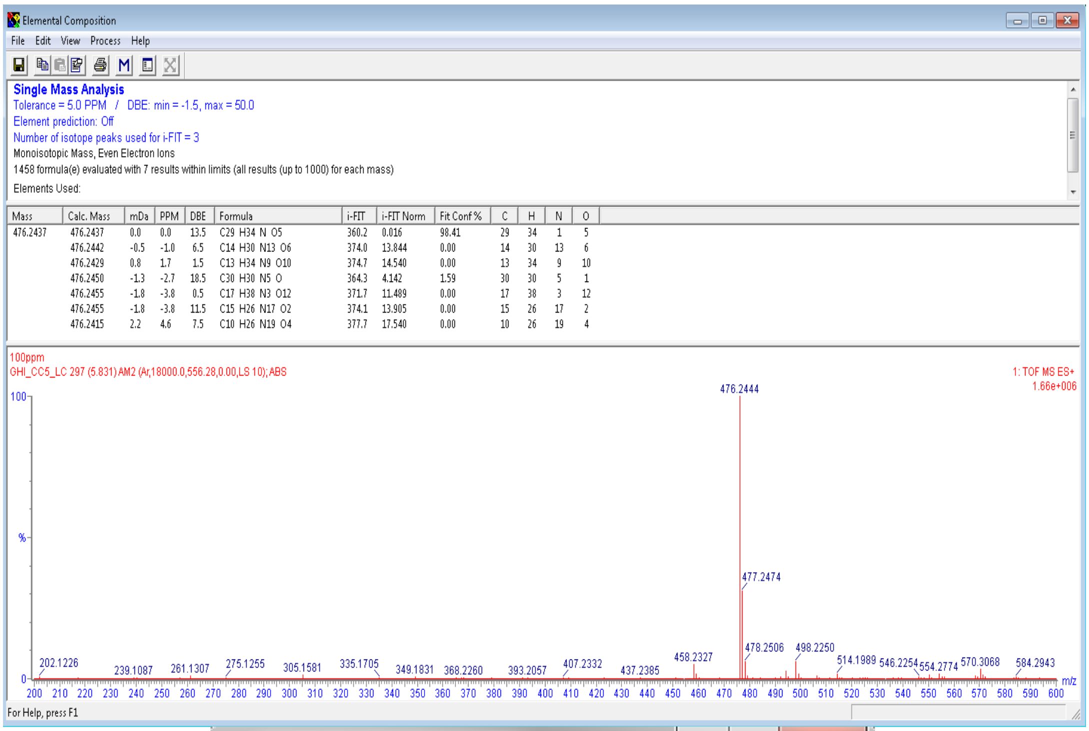

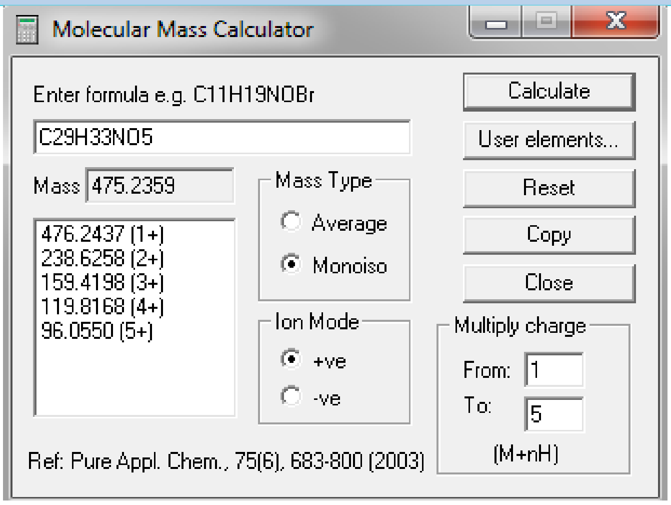

Figure S26. UV spectrum of norzoabenzaldehyde (**1**)

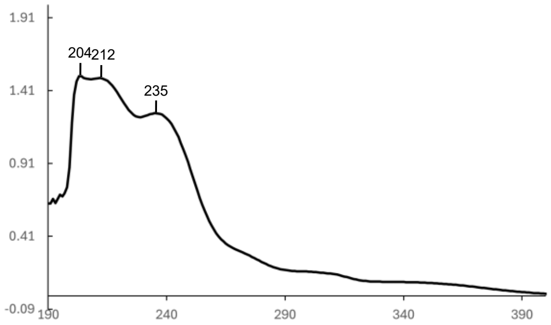

Figure S27. IR (ATR) spectrum of norzoabenzaldehyde (**1**)

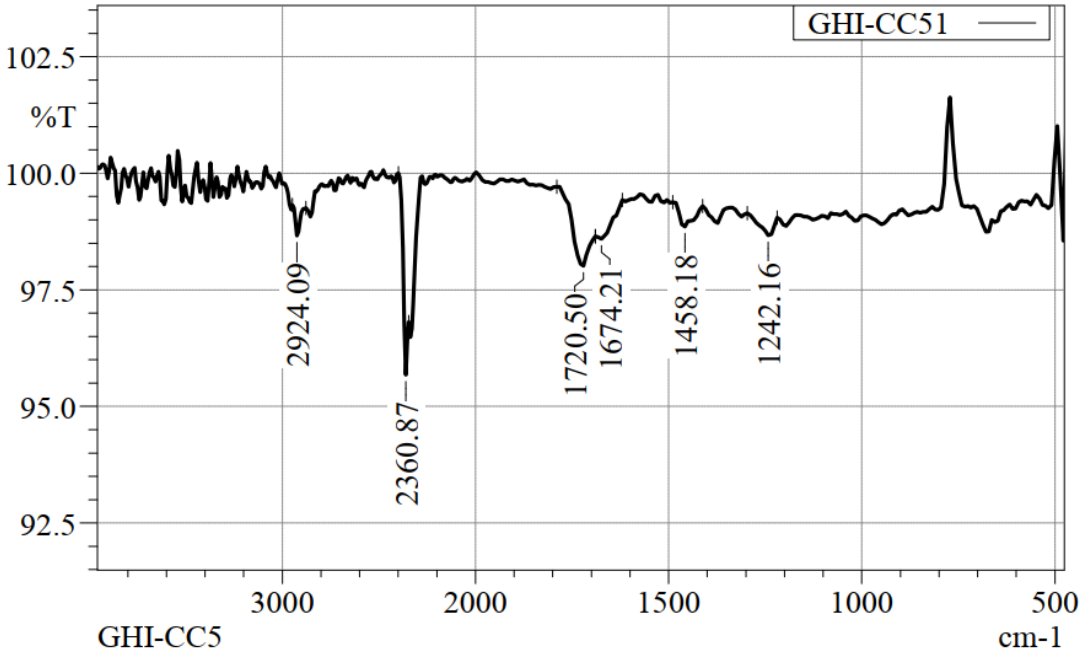

Figure S28. ^{1}H-NMR spectrum of norzoazepanol (**2**) at 600 MHz in $CDCl_3$

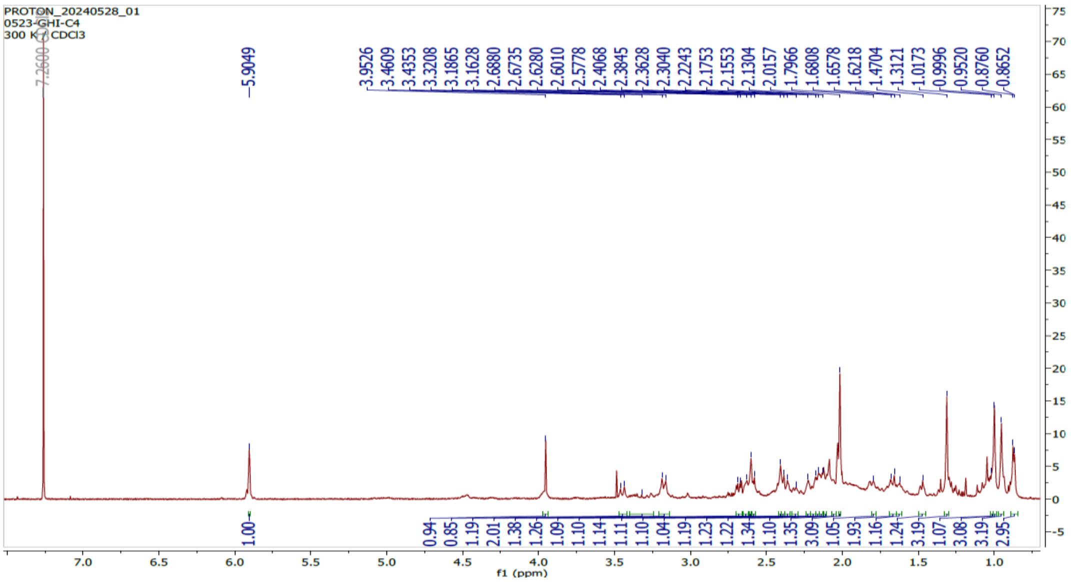

Figure S29. ^{1}H-NMR spectrum of norzoazepanol (3.0-6.1 ppm) at 600 MHz in $CDCl_3$

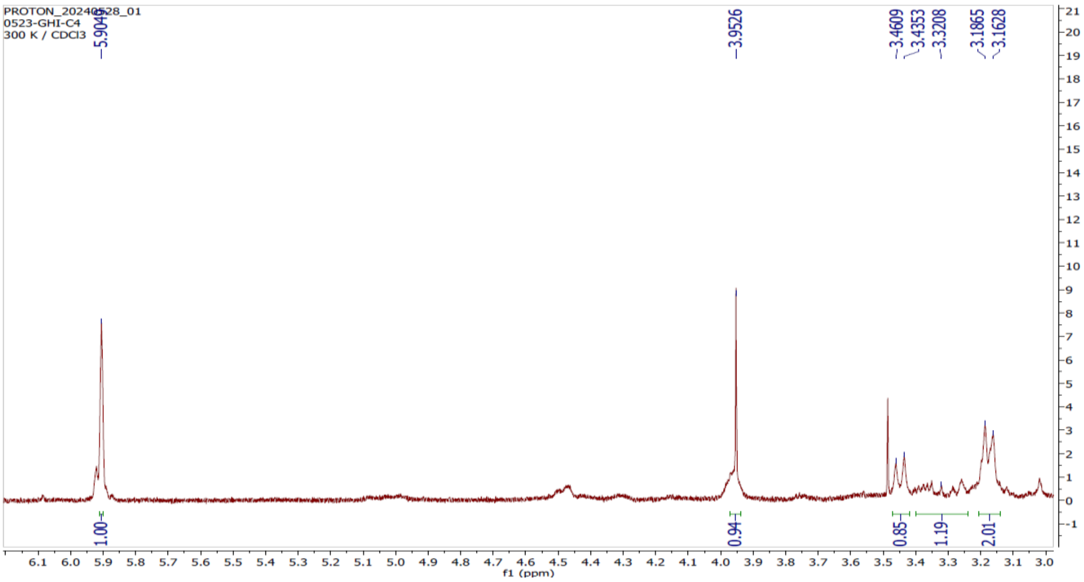

Figure S30. ^{1}H-NMR spectrum of norzoazepanol (0.8-2.9 ppm) at 600 MHz in $CDCl_3$

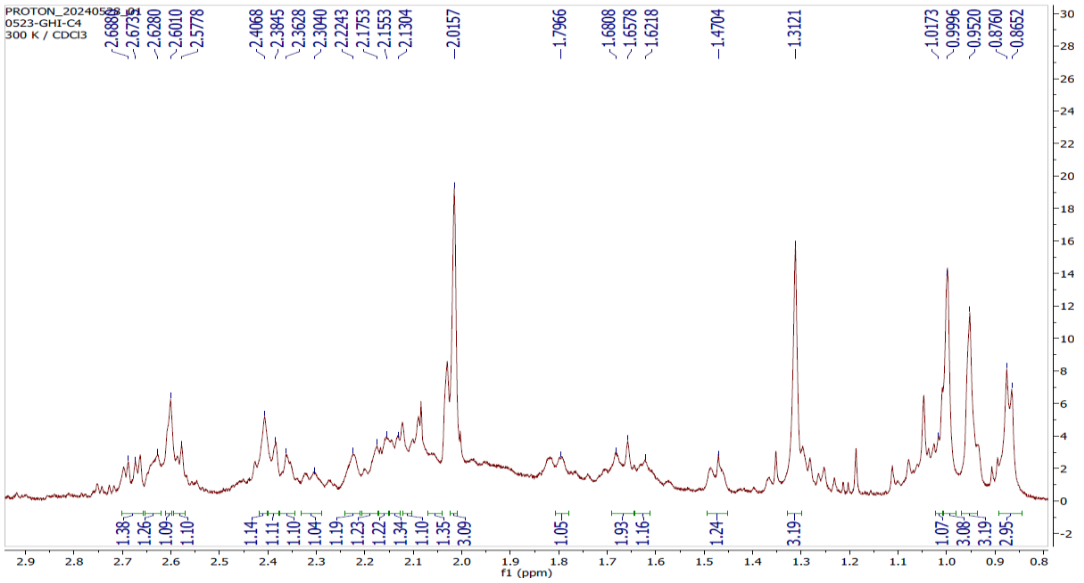

Figure S31. ^{13}C-NMR spectrum of norzoazepanol (**2**) at 150 MHz in $CDCl_3$

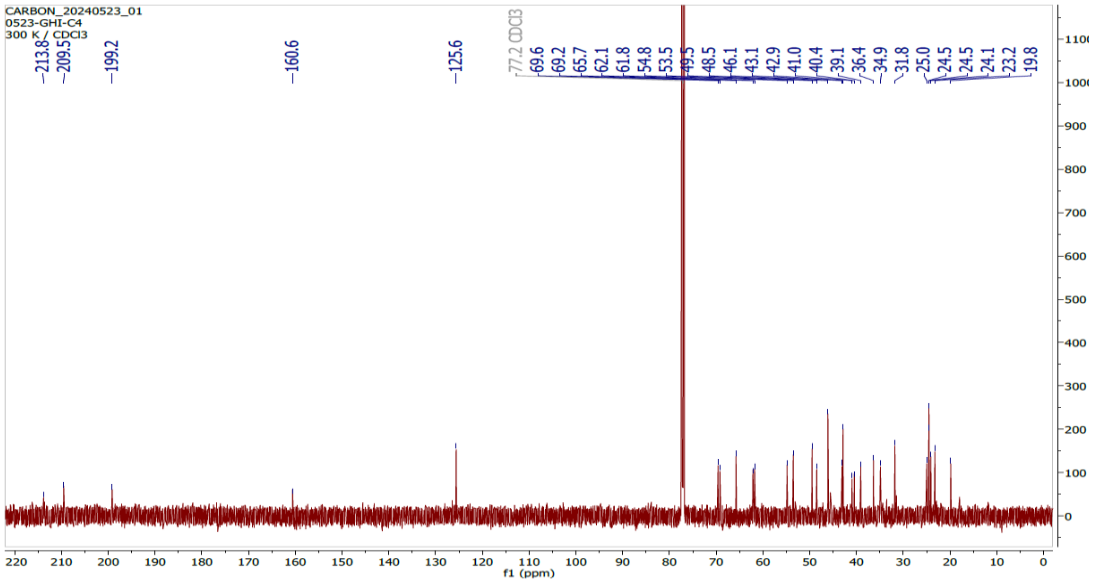

Figure S32. ^{13}C-NMR spectrum of norzoazepanol (120-220 ppm) at 150 MHz in $CDCl_3$

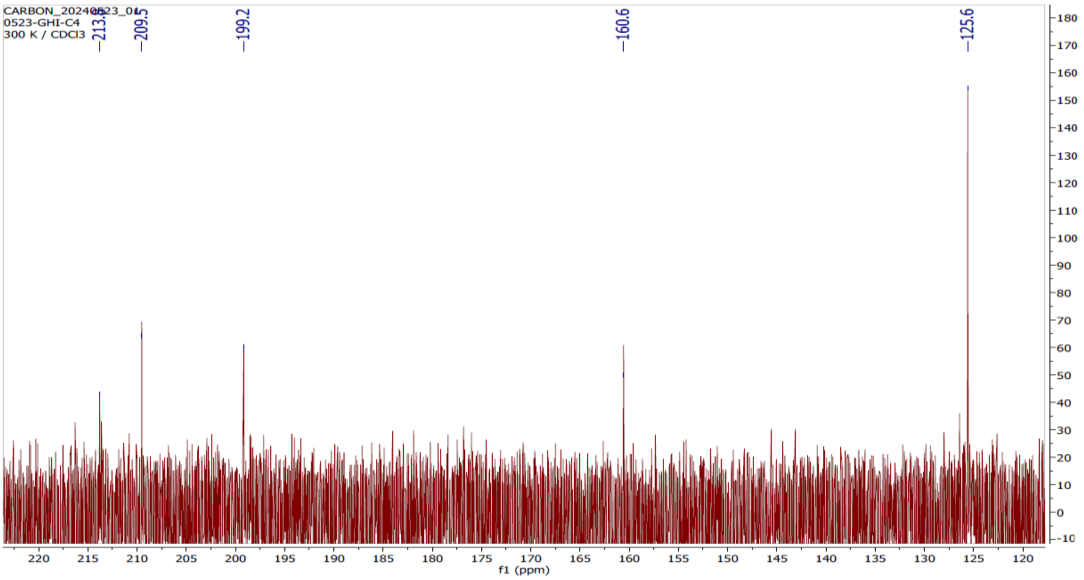

Figure S33. ^{13}C-NMR spectrum of norzoazepanol (20-74 ppm) at 150 MHz in $CDCl_3$

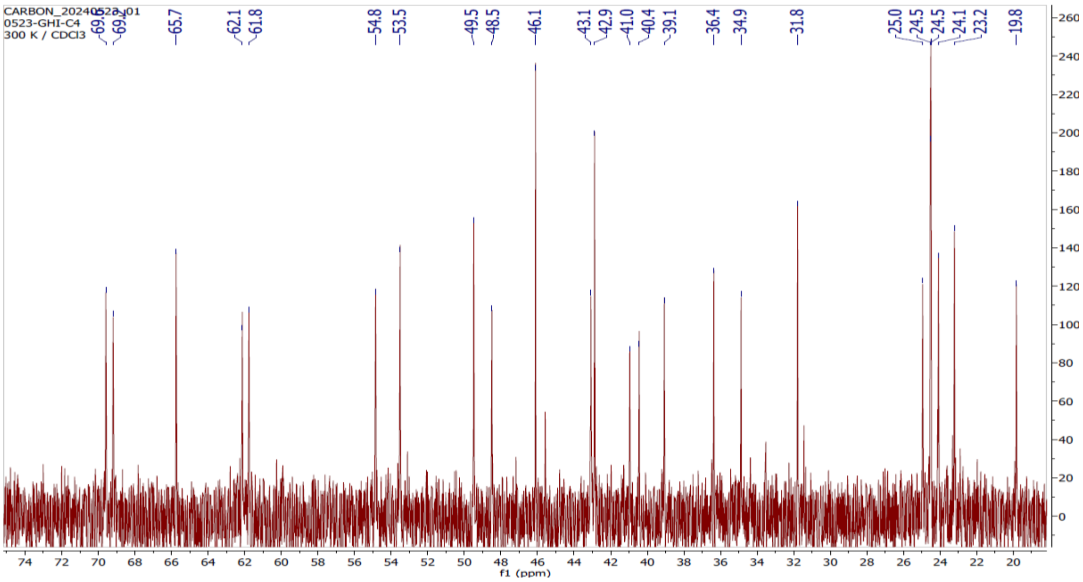

Figure S34. COSY spectrum of norzoazepanol (**2**) at 600 MHz in $CDCl_3$

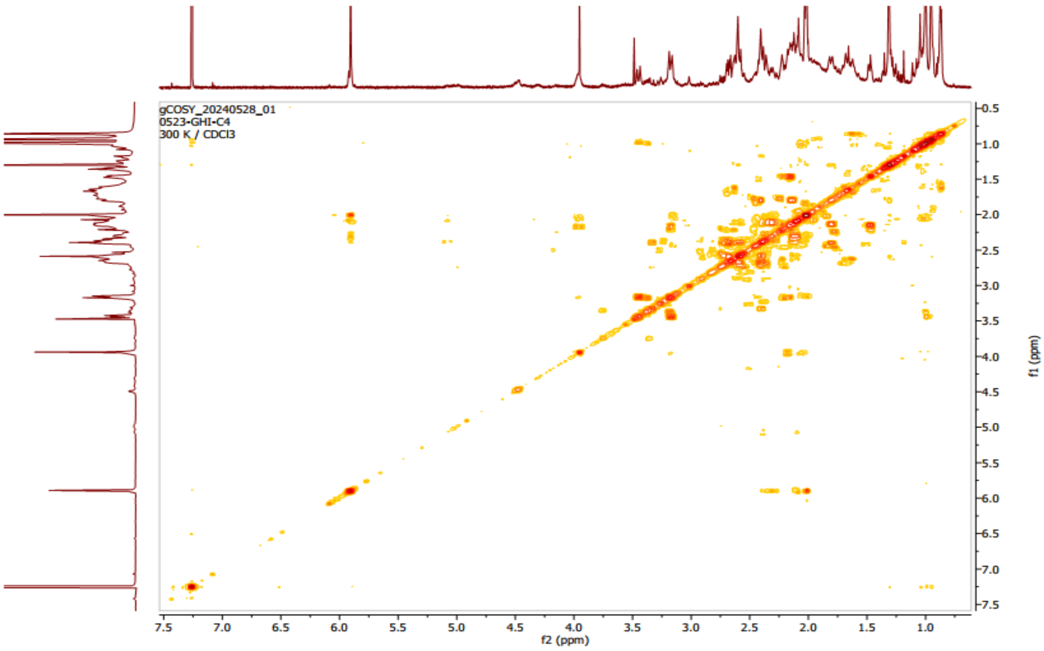

Figure S35. COSY spectrum of norzoazepanol (2.9-4.3 ppm) at 600 MHz in $CDCl_3$

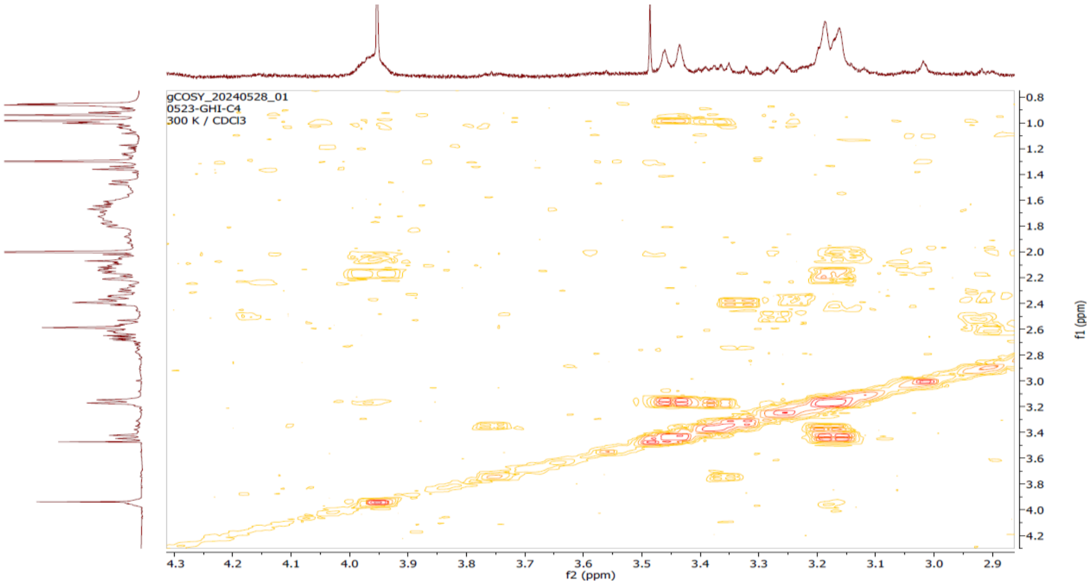

Figure S36. COSY spectrum of norzoazepanol (0.9-2.8 ppm) at 600 MHz in $CDCl_3$

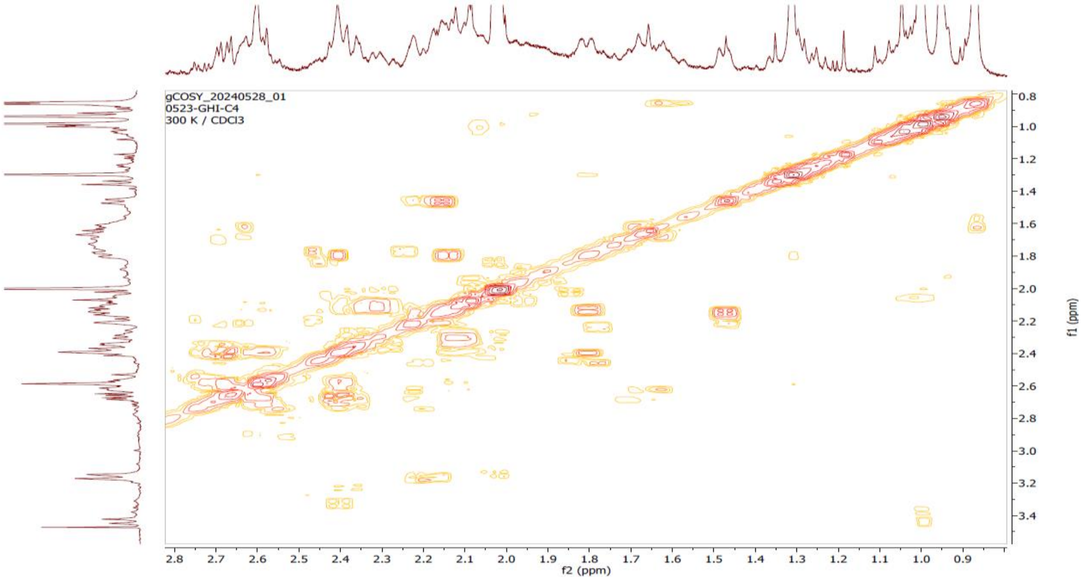

Figure S37. HSQC spectrum of norzoazepanol (**2**) at 600 and 150 MHz in $CDCl_3$

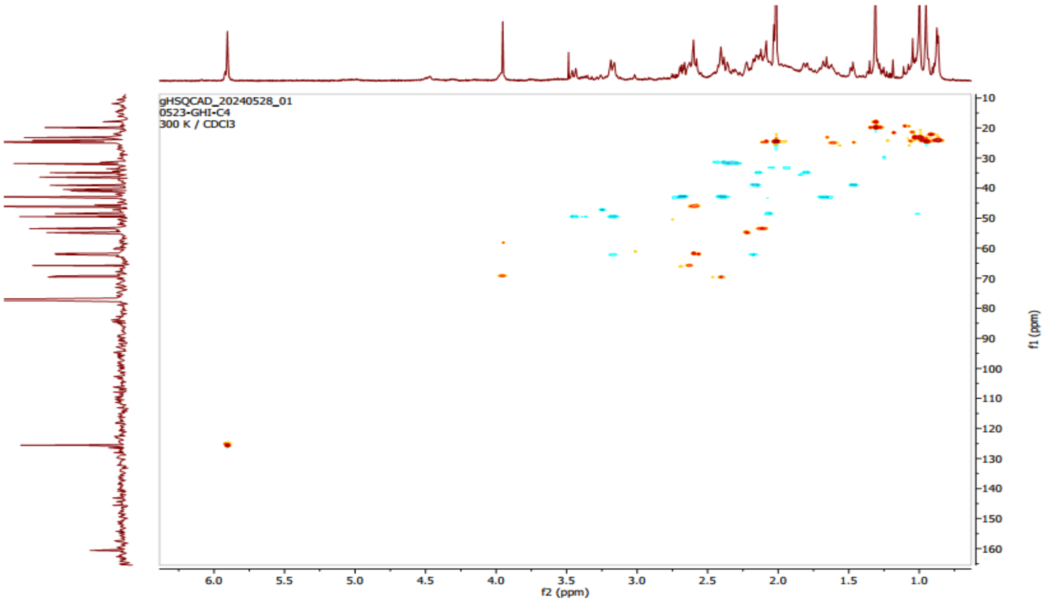

Figure S38. HSQC spectrum of norzoazepanol (3.80-6.10 ppm) at 600 and 150 MHz in $CDCl_3$

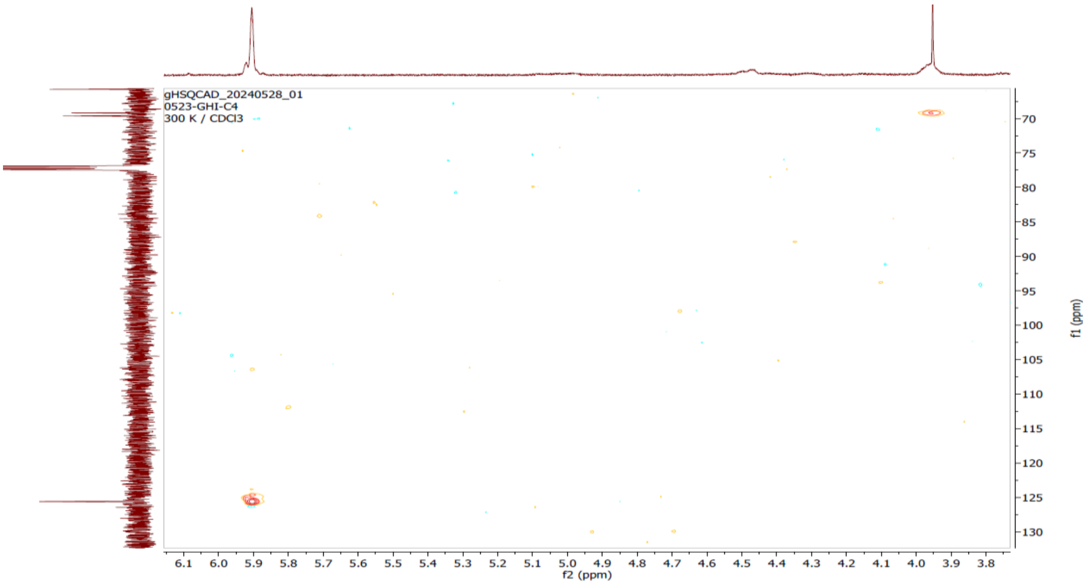

Figure S39. HSQC spectrum of norzoazepanol (0.80-3.50 ppm) at 600 and 150 MHz in $CDCl_3$

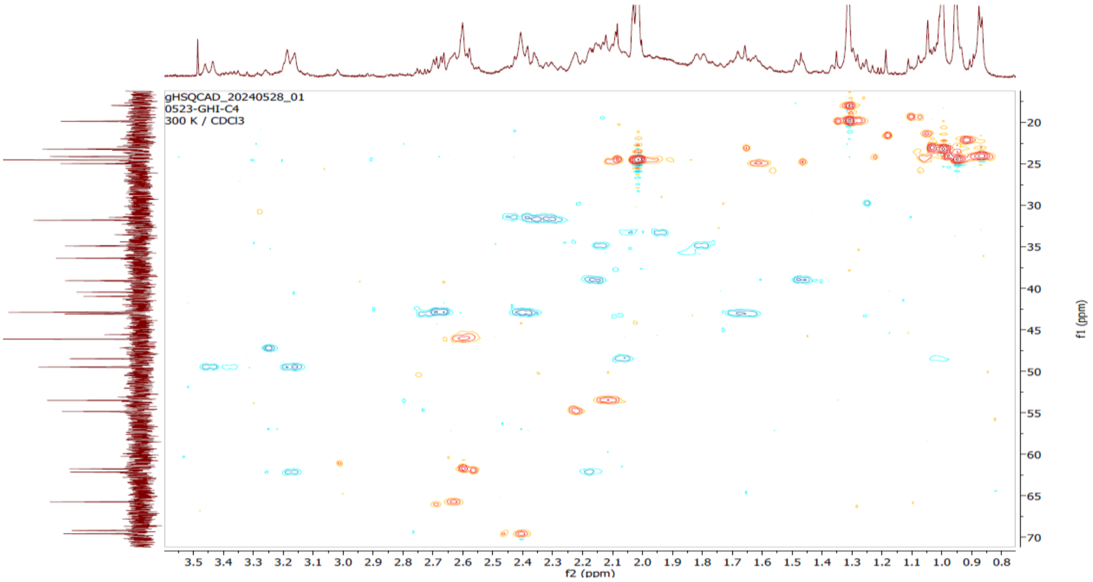

Figure S40. HMBC spectrum of norzoazepanol (**2**) at 600 and 150 MHz in $CDCl_3$

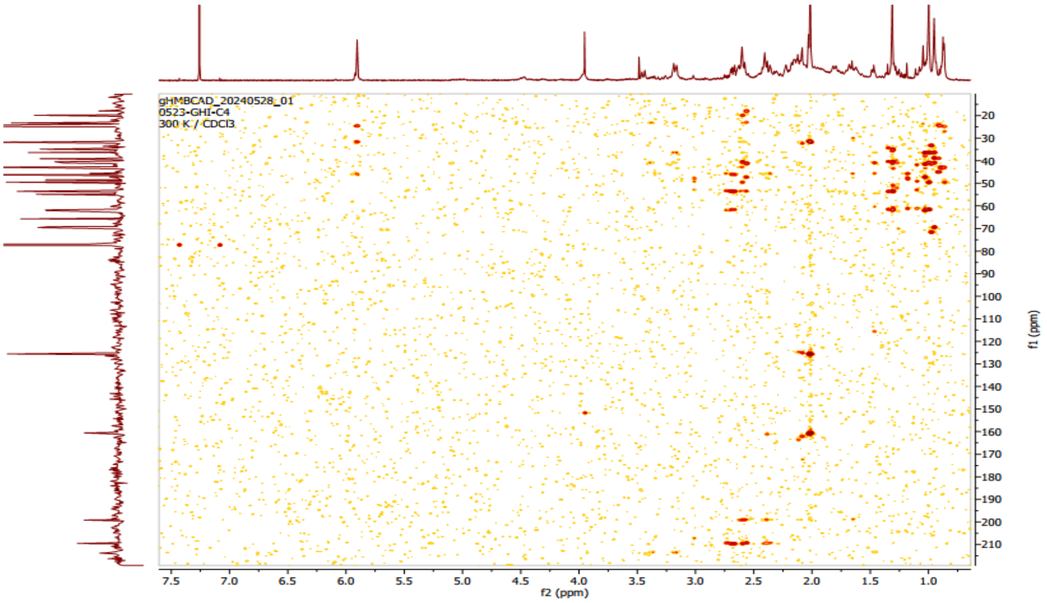

Figure S41. HMBC spectrum of norzoazepanol (5.78-6.03 ppm) at 600 and 150 MHz in $CDCl_3$

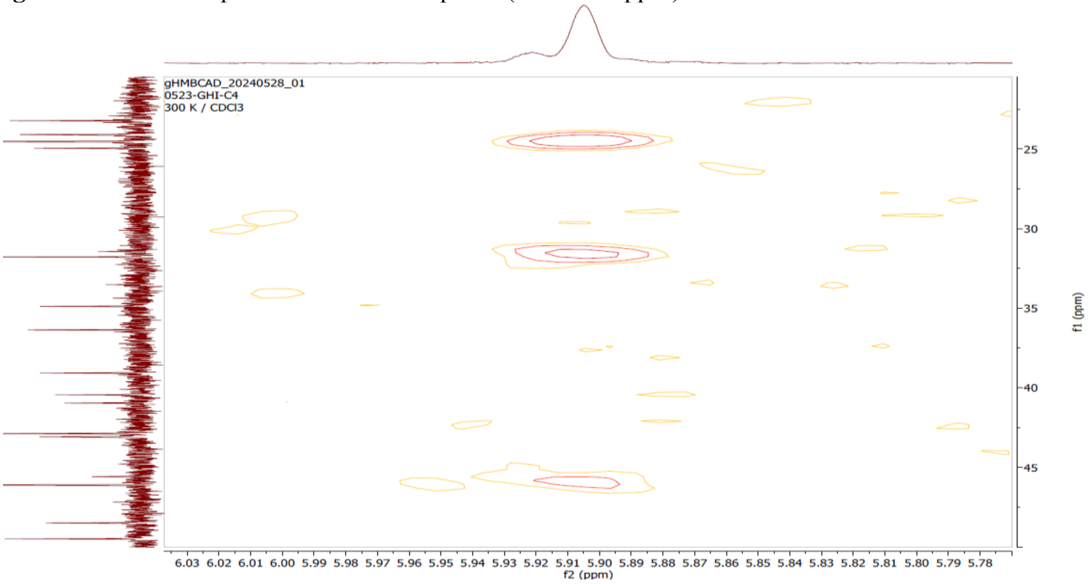

Figure S42. HMBC spectrum of norzoazepanol (2.0-3.2 ppm) at 600 and 150 MHz in $CDCl_3$

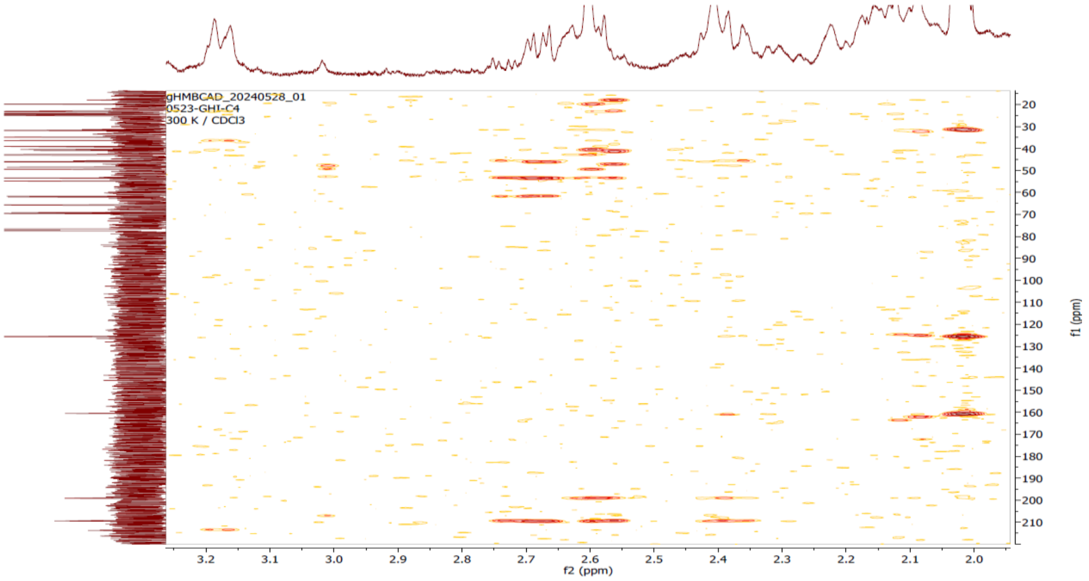

Figure S43. HMBC spectrum of norzoazepanol (0.85-1.45 ppm) at 600 and 150 MHz in $CDCl_3$

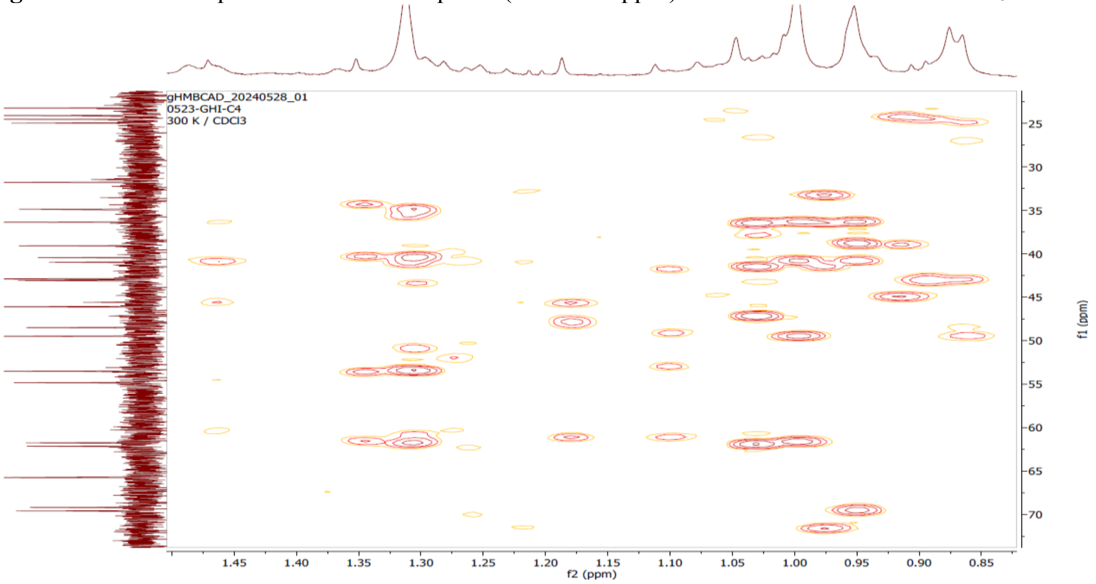

Figure S44. NOESY spectrum of norzoazepanol (**2**) at 600 MHz in $CDCl_3$

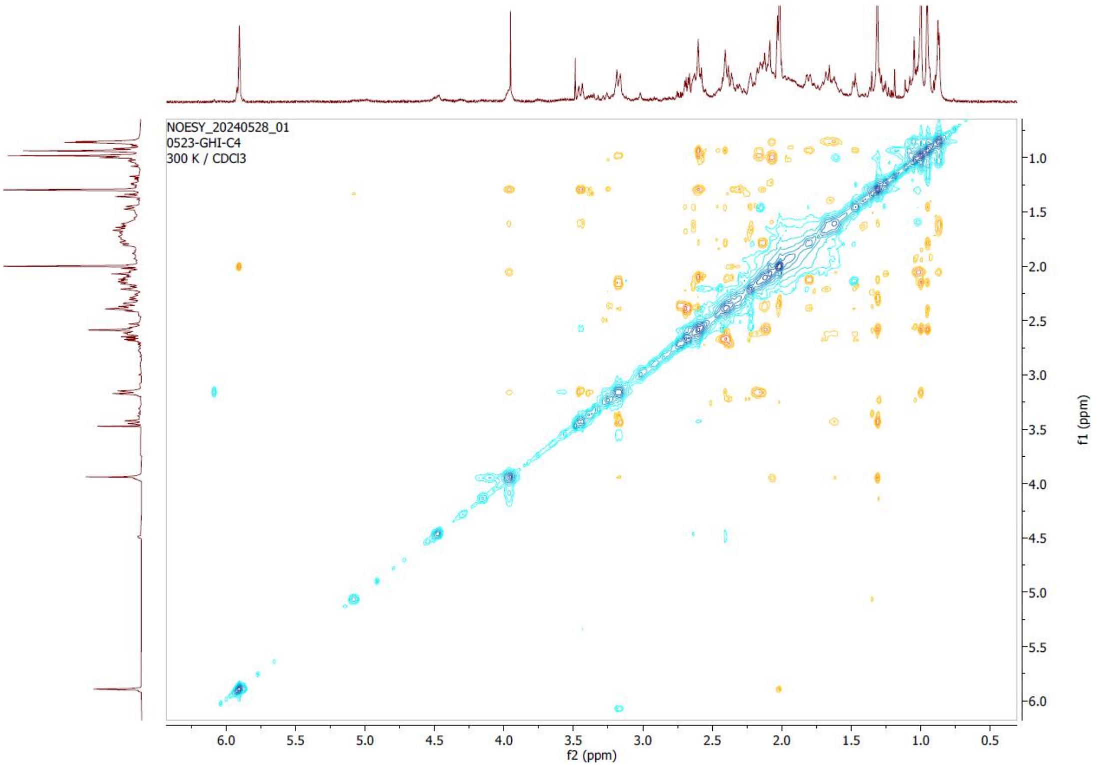

Figure S45. NOESY spectrum of norzoazepanol (2.01-2.29 ppm) at 600 MHz in $CDCl_3$

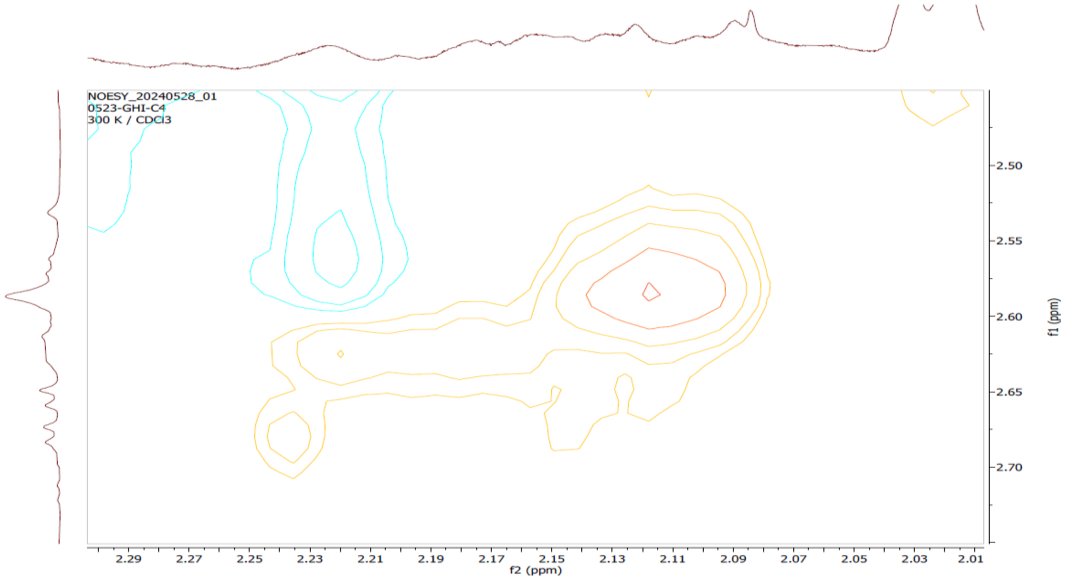

Figure S46. NOESY spectrum of norzoazepanol (1.4-1.56 ppm) at 600 MHz in $CDCl_3$

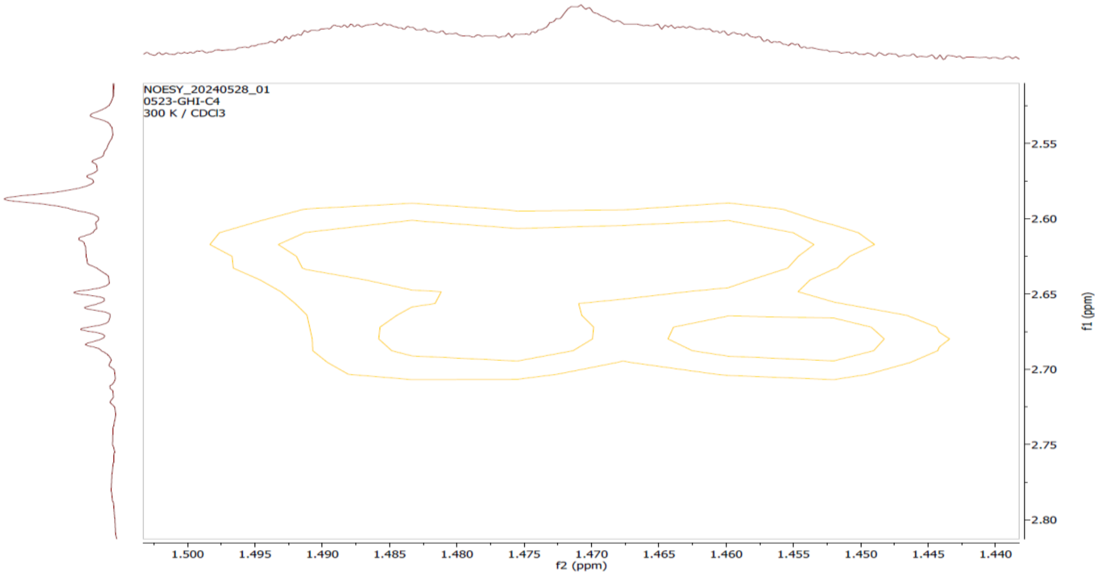

Figure S47. NOESY spectrum of norzoazepanol (1.45-1.50 ppm) at 600 MHz in $CDCl_3$

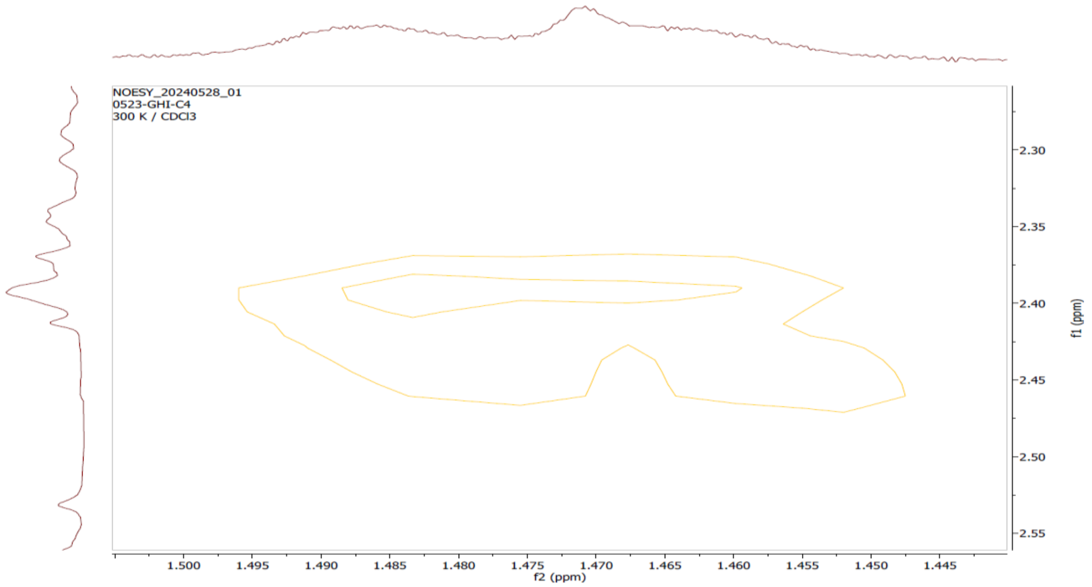

Figure S48. NOESY spectrum of norzoazepanol (0.83-1.08 ppm) at 600 MHz in $CDCl_3$

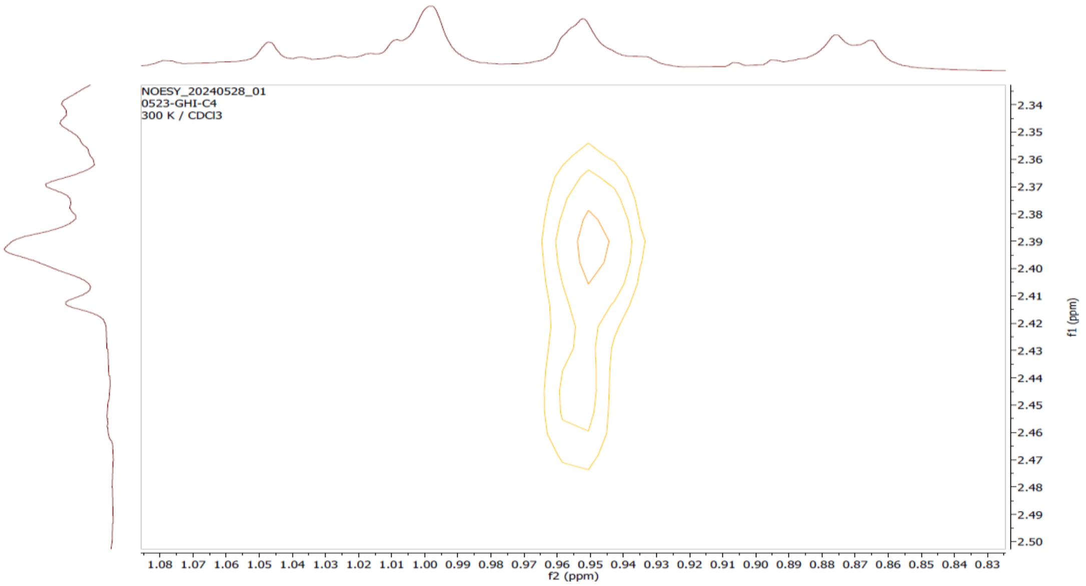

Figure S49. NOESY spectrum of norzoazepanol (2.36-2.76 ppm) at 600 MHz in $CDCl_3$

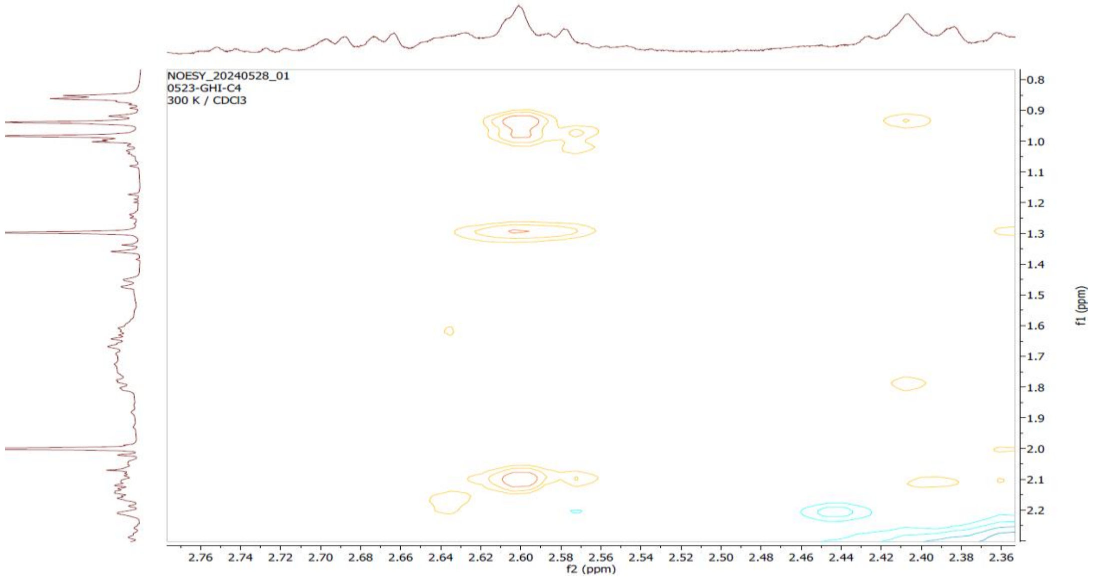

Figure S50. MS spectrum of norzoazepanol (**2**)

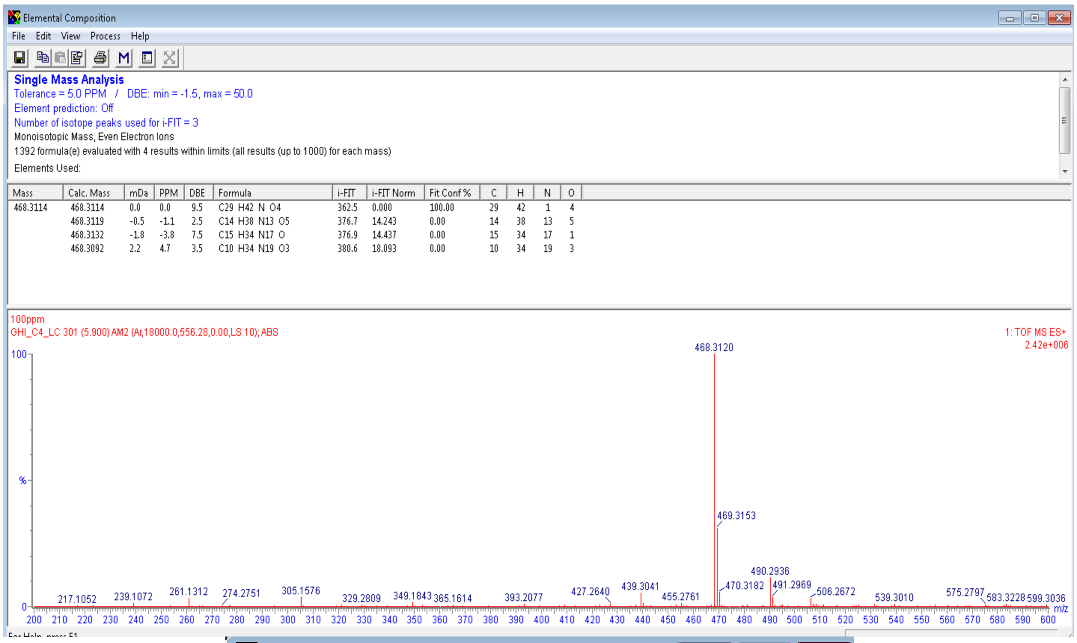

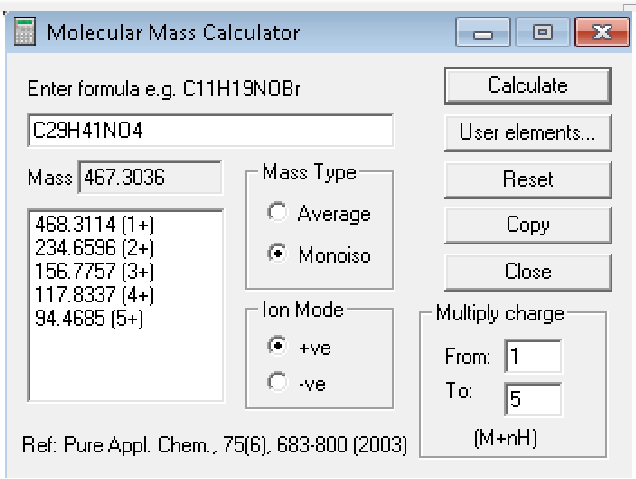

Figure S51. UV spectrum of norzoazepanol (**2**)

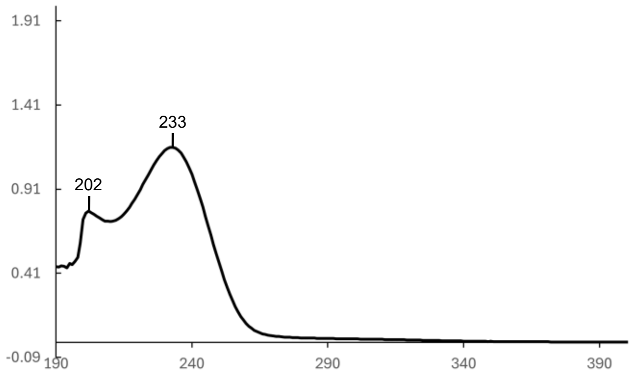

Figure S52. IR (ATR) spectrum of norzoazepanol (**2**)

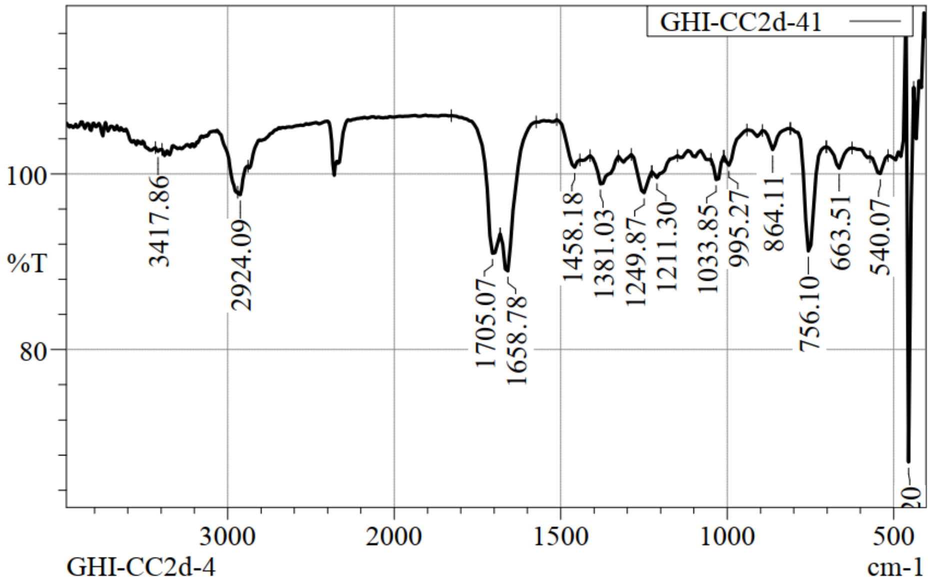

Figure S53. DP4+ results obtained using experimental data of compound **2** *versus* isomers 2*S*-**2** (isomer 1) and 2*R*-**2** (isomer 2)

Functional		Solvent?		Basis Set		Type of Data	
mPW1PW91		PCM		6-311+G(d,p)		Unscaled Shifts	
		DP4+	0.00%	100.00%	-	-	-
Nuclei	sp2?	Experimental	Isomer 1	Isomer 2	Isomer 3	Isomer 4	Isomer 5
C		62.1	61.1	58.8			
C		69.2	67.9	67.4			
C		48.5	41.2	44.7			
C		25	24.9	23.5			
C		42.9	39.2	39.3			
C		65.8	62.6	64.0			
C		54.9	53.9	54.3			
C		39.1	35.6	35.9			
C		36.4	36.2	36.2			
C		69.6	68.3	67.9			
C		34.9	31.9	31.7			
C		40.5	42.50	42.00			
C		53.5	51.90	51.70			
C		31.8	28.90	28.80			
C	x	160.6	170.20	170.20			
C	x	125.6	126.00	126.00			
C	x	199.2	203.30	203.30			
C		46.1	45.40	45.30			
C		42.8	41.80	41.80			
C	x	209.5	219.10	219.10			
C		61.8	60.10	60.30			
C		41	42.40	42.10			
C		49.5	47.60	48.20			
C	x	213.8	221.40	221.60			
C		23.2	17.40	17.80			
C		24.5	21.5	21.6			
C		19.8	14.9	15.2			
C		24.5	20.2	20			
C		24.1	17.8	19.1			
H		3.17	3.21	3.14			
H		2.17	2.41	2.05			
H		3.95	3.93	4.04			
H		2.07	1.94	2.01			
H		1.02	1.35	1			
H		1.62	1.9	1.67			
H		1.67	1.63	1.66			
H		1.67	1.56	1.58			
H		2.63	2.63	2.67			
H		2.22	2.15	2.09			
H		2.17	2.04	2.17			
H		1.47	1.65	1.49			
H		2.41	2.39	2.4			
H		2.13	2.26	2.18			
H		1.8	1.76	1.78			
H		2.1	2.15	2.17			
H		2.38	2.46	2.46			
H		2.28	2.42	2.43			
H		5.9	6.29	6.3			
H		2.58	2.69	2.69			
H		2.39	2.4	2.38			
H		2.68	2.7	2.71			
H		2.61	2.8	2.82			
H		3.45	3.42	3.41			
H		3.15	3.42	3.41			
H		0.99	1.01	1.03			
H		2.02	2.16	2.17			
H		1.32	1.08	0.99			
H		0.95	0.95	0.92			
H		0.86	0.93	0.9			

Functional	Solvent?		Basis Set		Type of Data	
mPW1PW91	PCM		6-311+G(d,p)		Unscaled Shifts	
	Isomer 1	Isomer 2	Isomer 3	Isomer 4	Isomer 5	Isomer 6
sDP4+ (H data)	0.03%	99.97%	-	-	-	-
sDP4+ (C data)	0.94%	99.06%	-	-	-	-
sDP4+ (all data)	0.00%	100.00%	-	-	-	-
uDP4+ (H data)	0.12%	99.88%	-	-	-	-
uDP4+ (C data)	13.84%	86.16%	-	-	-	-
uDP4+ (all data)	0.02%	99.98%	-	-	-	-
DP4+ (H data)	0.00%	100.00%	-	-	-	-
DP4+ (C data)	0.15%	99.85%	-	-	-	-
DP4+ (all data)	0.00%	100.00%	-	-	-	-

Figure S54. The cartesian coordinates of the dominant conformers for 2*S*-**2** and 2*R*-**2**

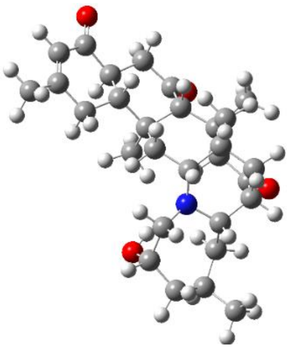

2*S*-**2**-1 (30.13%)

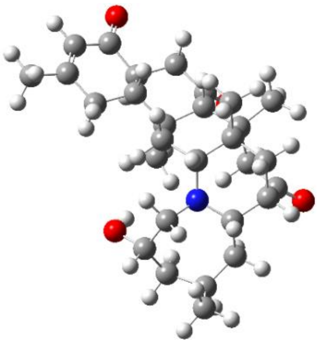

2*S*-**2**-2 (13.82%)

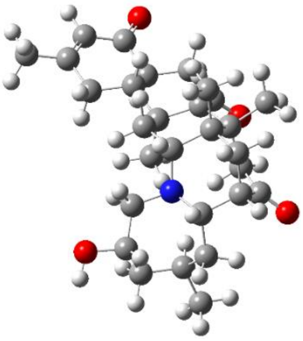

2*S*-**2**-3 (1.54%)

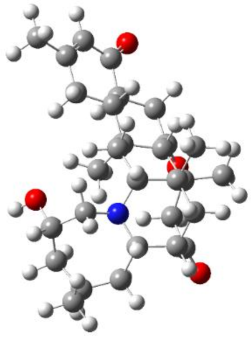

2*S*-**2**-4 (18.07%)

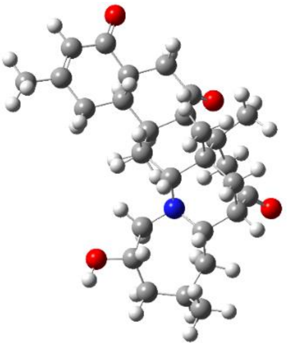

2*S*-**2**-5 (1.65%)

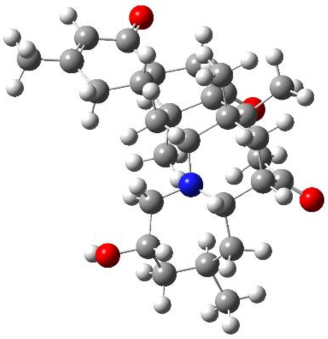

2*S*-**2**-6 (1.48%)

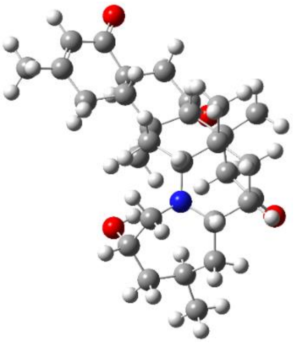

2*S*-**2**-7 (22.76%)

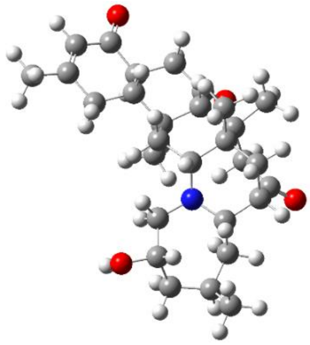

2*S*-**2**-8 (1.22%)

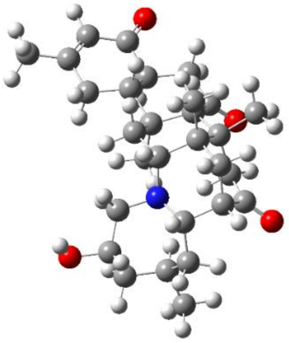

2*S*-**2**-9 (1.36%)

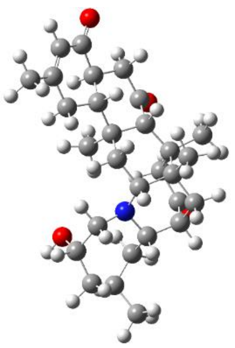

2*S*-**2**-10 (7.98%)

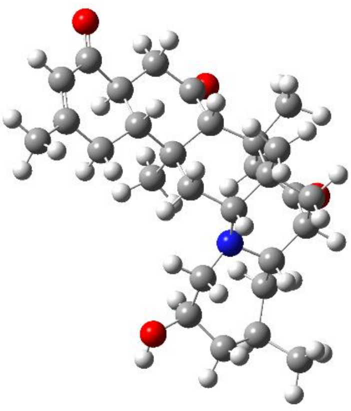

2*R*-**2**-1 (6.15%)

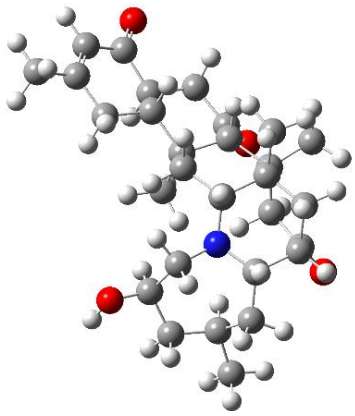

2*R*-**2**-2 (29.42%)

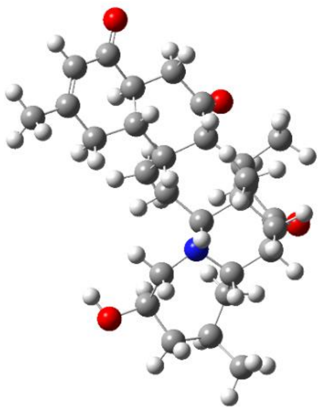

2*R*-**2**-3 (4.53%)

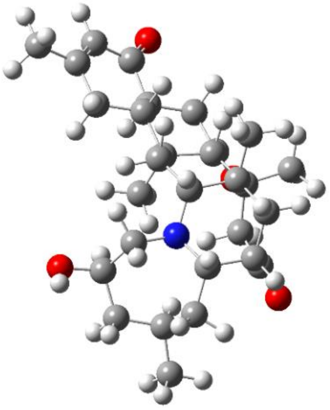

2*R*-**2**-4 (35.68%)

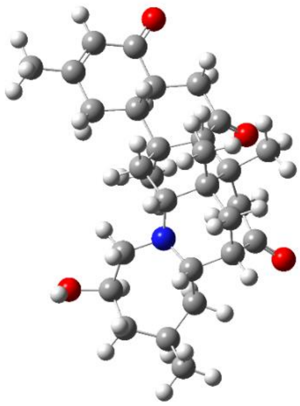

2*R*-**2**-5 (8.59%)

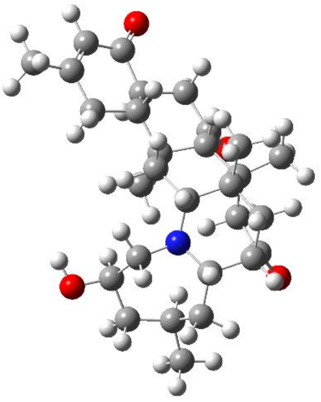

2*R*-**2**-6 (14.81%)

Figure S55. ^{1}H-NMR spectrum of 3-acetoxynorzoanthaminone (**3**) at 600 MHz in $CDCl_3$

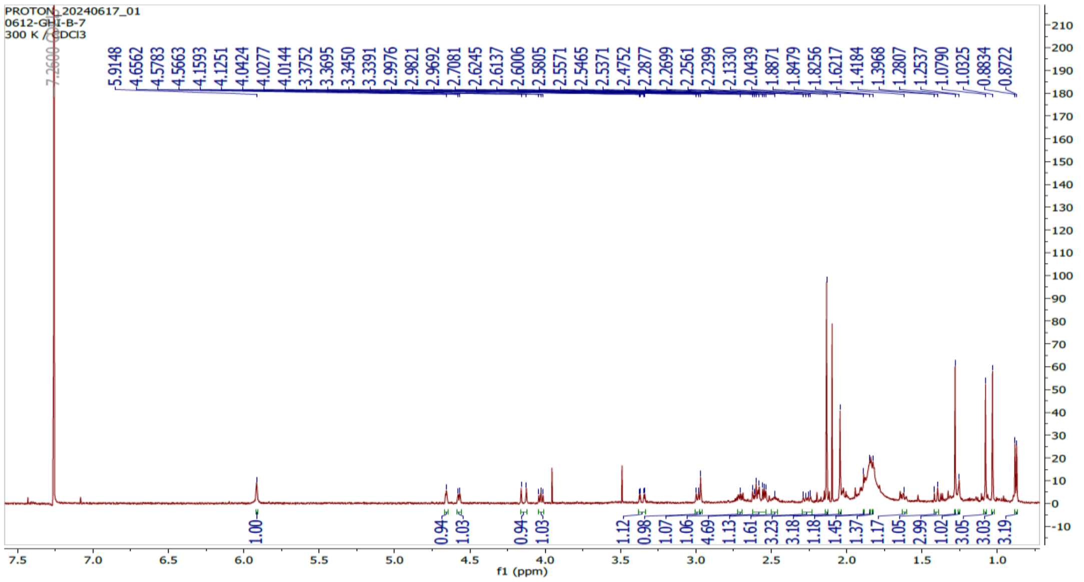

Figure S56. ^{1}H-NMR spectrum of 3-acetoxynorzoanthaminone (2.8-6.2 ppm) at 600 MHz in $CDCl_3$

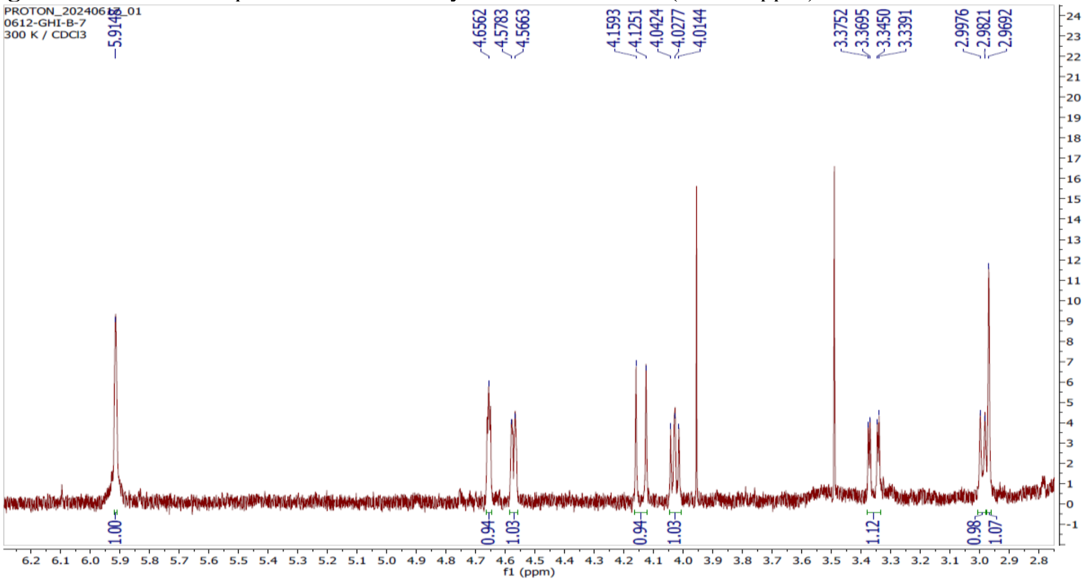

Figure S57. ^{1}H-NMR spectrum of 3-acetoxynorzoanthaminone (0.8-2.9 ppm) at 600 MHz in $CDCl_3$

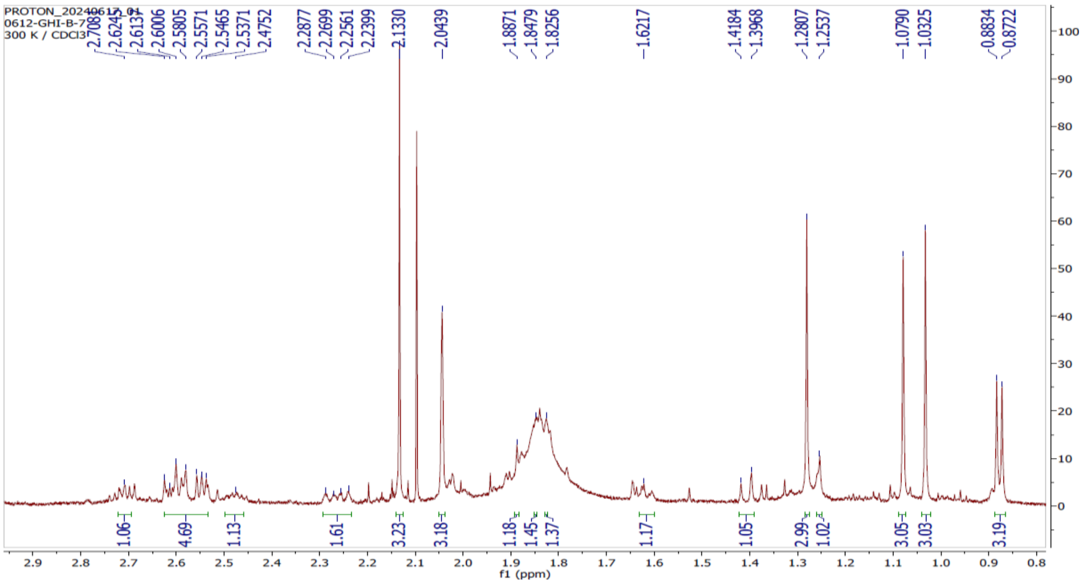

Figure S58. ^{13}C-NMR spectrum of 3-acetoxynorzoanthaminone (**3**) at 150 MHz in $CDCl_3$

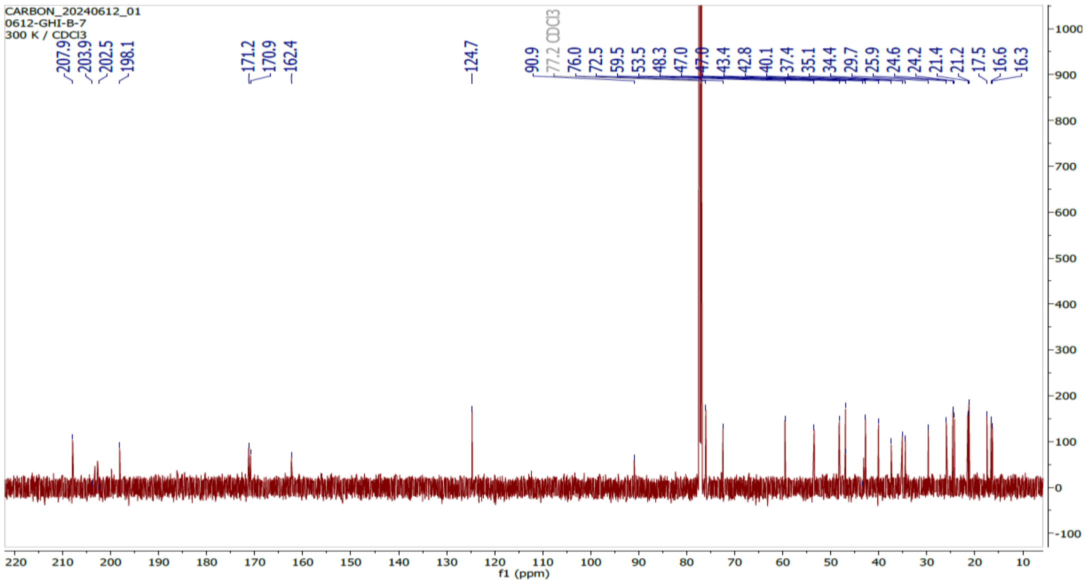

Figure S59. ^{13}C-NMR spectrum of 3-acetoxynorzoanthaminone (90-215 ppm) at 150 MHz in $CDCl_3$

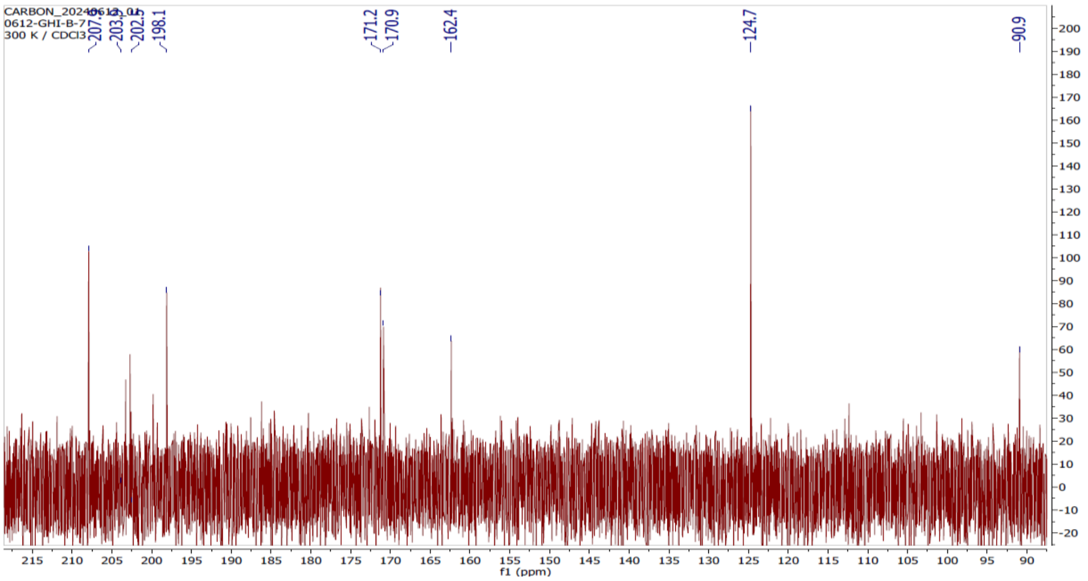

Figure S60. ^{13}C-NMR spectrum of 3-acetoxynorzoanthaminone (15-80 ppm) at 150 MHz in $CDCl_3$

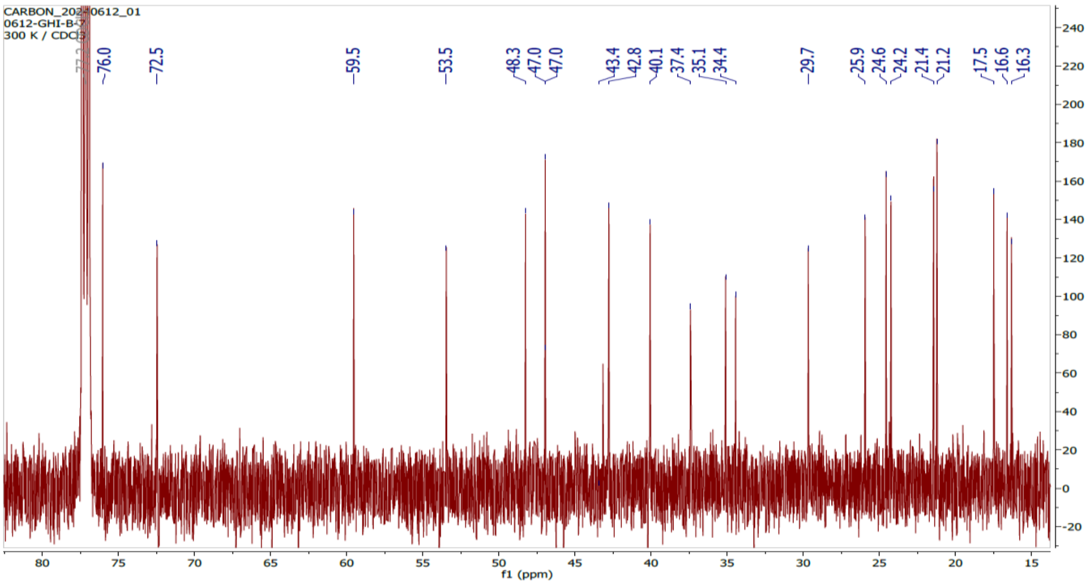

Figure S61. COSY spectrum of 3-acetoxynorzoanthaminone (**3**) at 600 MHz in $CDCl_3$

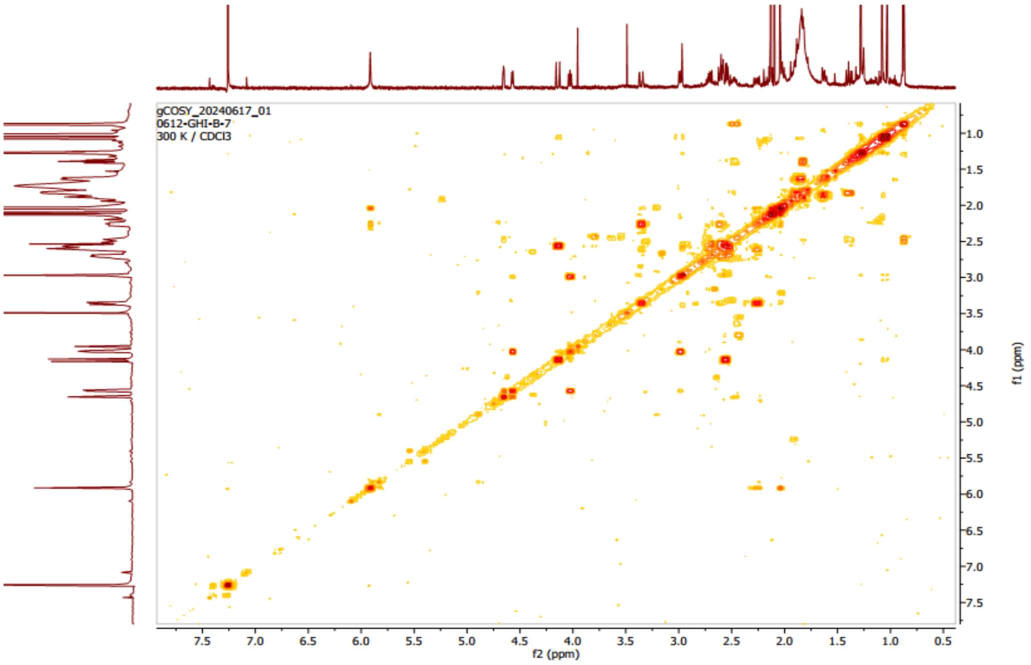

Figure S62. COSY spectrum of 3-acetoxynorzoanthaminone (0.8-4.8 ppm) at 600 MHz in $CDCl_3$

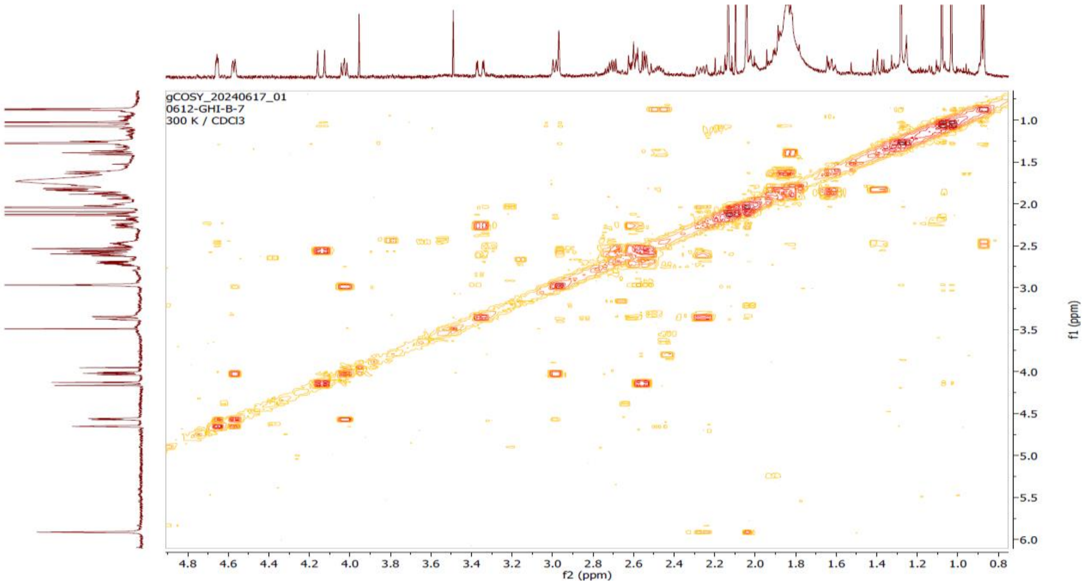

Figure S63. HSQC spectrum of 3-acetoxynorzoanthaminone (**3**) at 600 and 150 MHz in $CDCl_3$

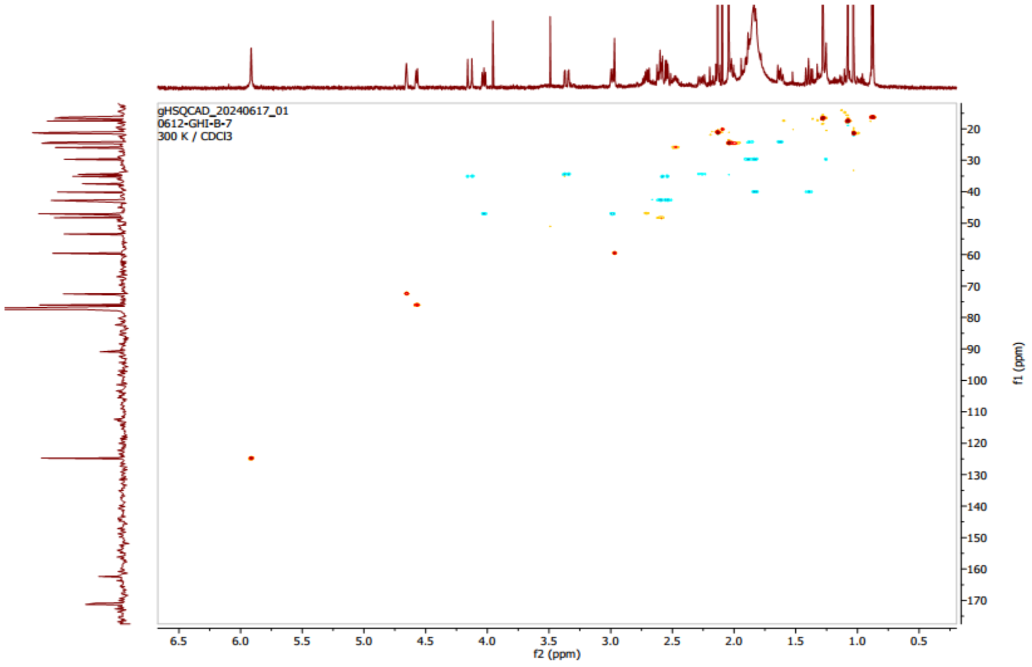

Figure S64. HSQC spectrum of 3-acetoxynorzoanthaminone (4.4-6.1 ppm) at 600 and 150 MHz in $CDCl_3$

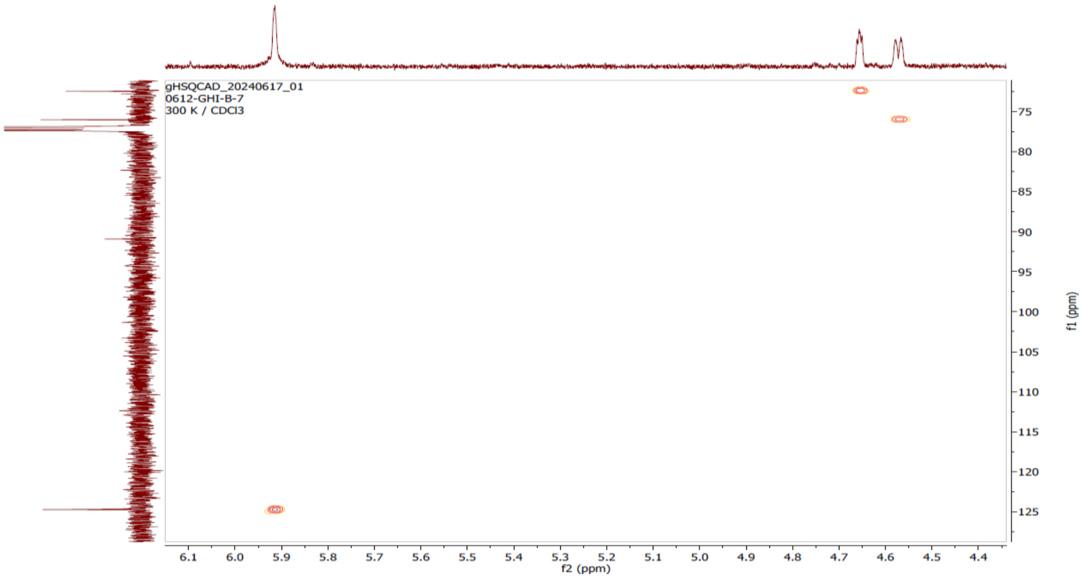

Figure S65. HSQC spectrum of 3-acetoxynorzoanthaminone (0.8-4.2 ppm) at 600 and 150 MHz in $CDCl_3$

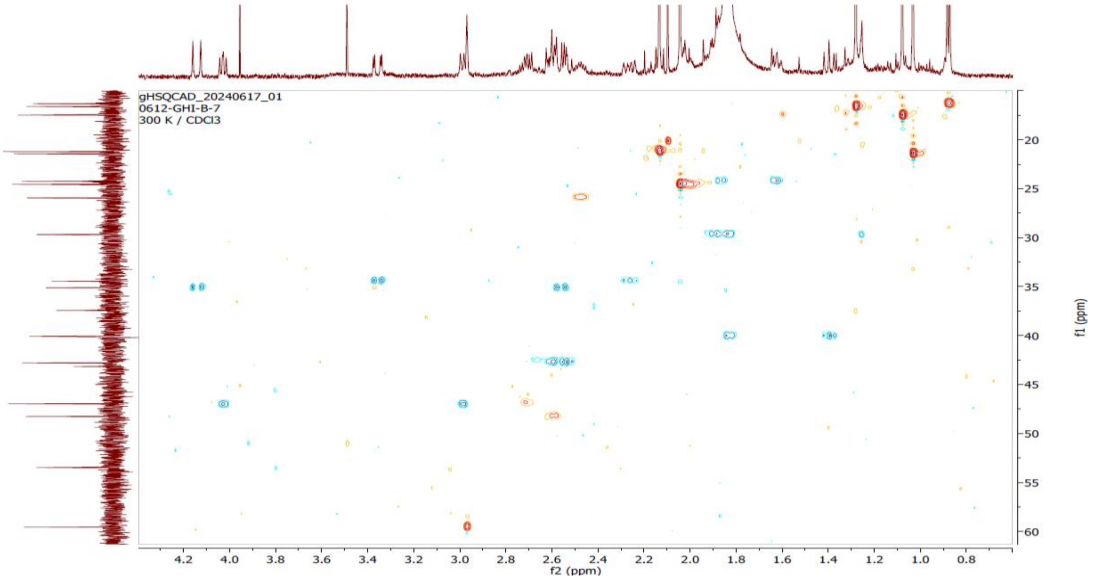

Figure S66. HMBC spectrum of 3-acetoxynorzoanthaminone (**3**) at 600 and 150 MHz in $CDCl_3$

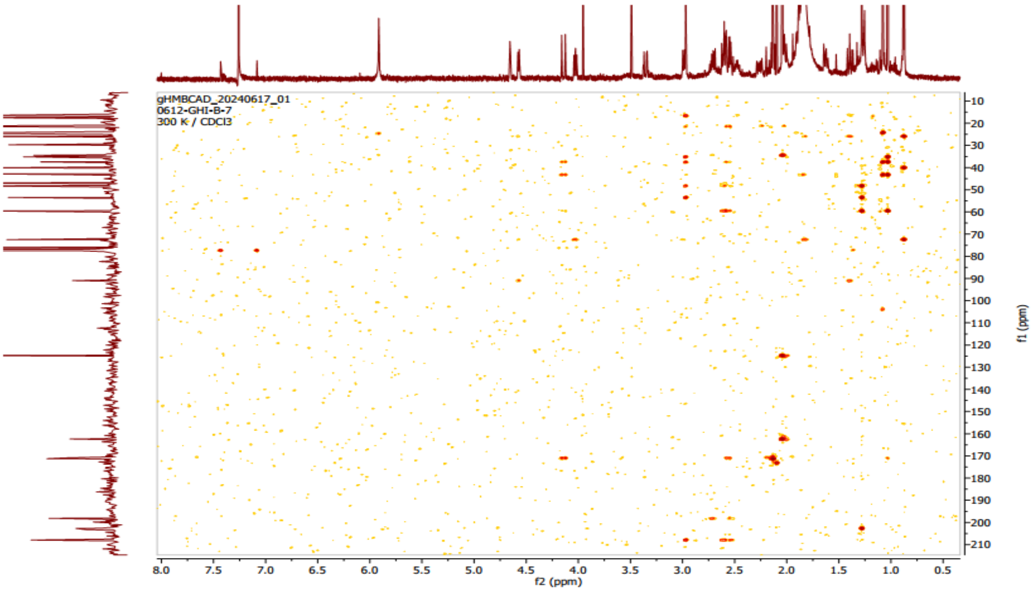

Figure S67. HMBC spectrum of 3-acetoxynorzoanthaminone (3.90-4.28 ppm) at 600 and 150 MHz in $CDCl_3$

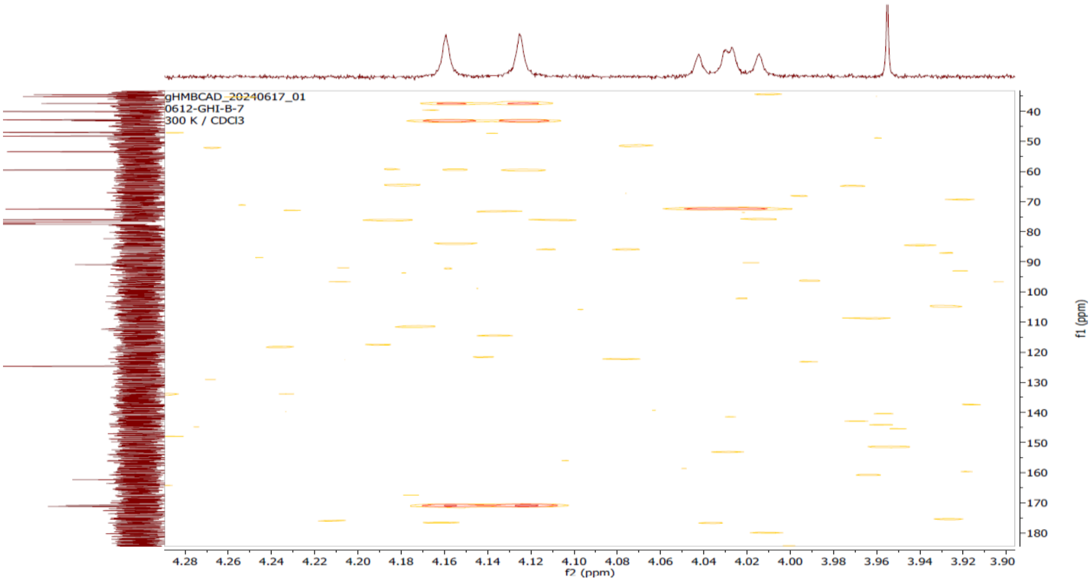

Figure S68. HMBC spectrum of 3-acetoxynorzoanthaminone **(**0.7-3.2 ppm) at 600 and 150 MHz in $CDCl_3$

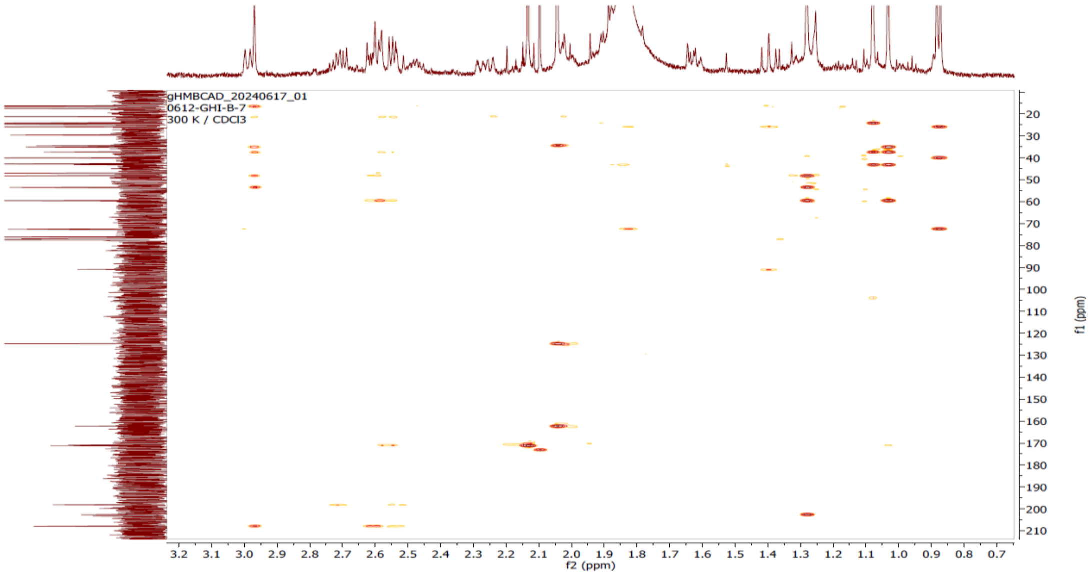

Figure S69. NOESY spectrum of 3-acetoxynorzoanthaminone (**3**) at 600 MHz in $CDCl_3$

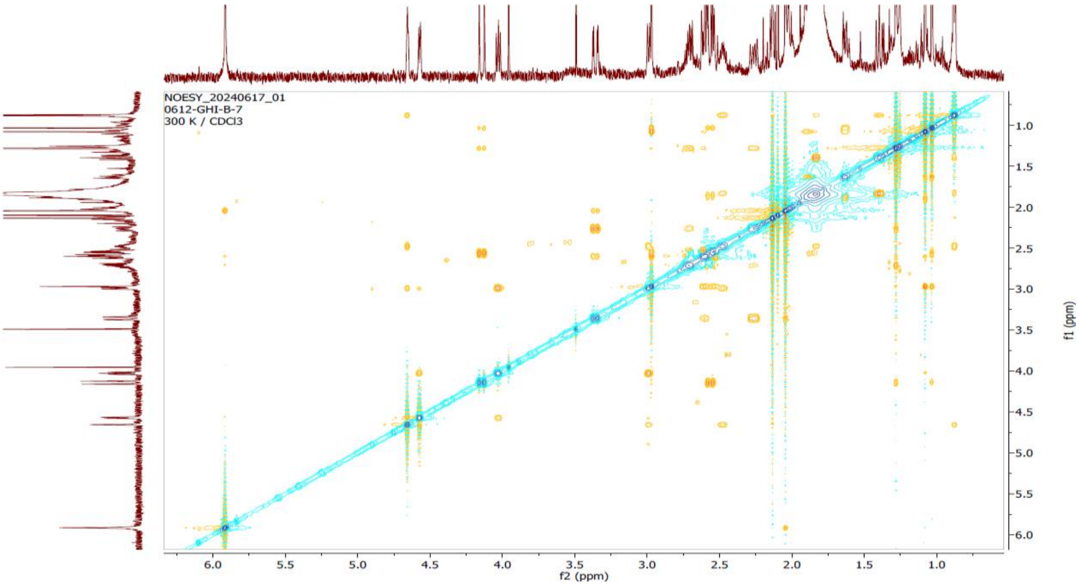

Figure S70. NOESY spectrum of 3-acetoxynorzoanthaminone (2.65-2.77 ppm) at 600 MHz in $CDCl_3$

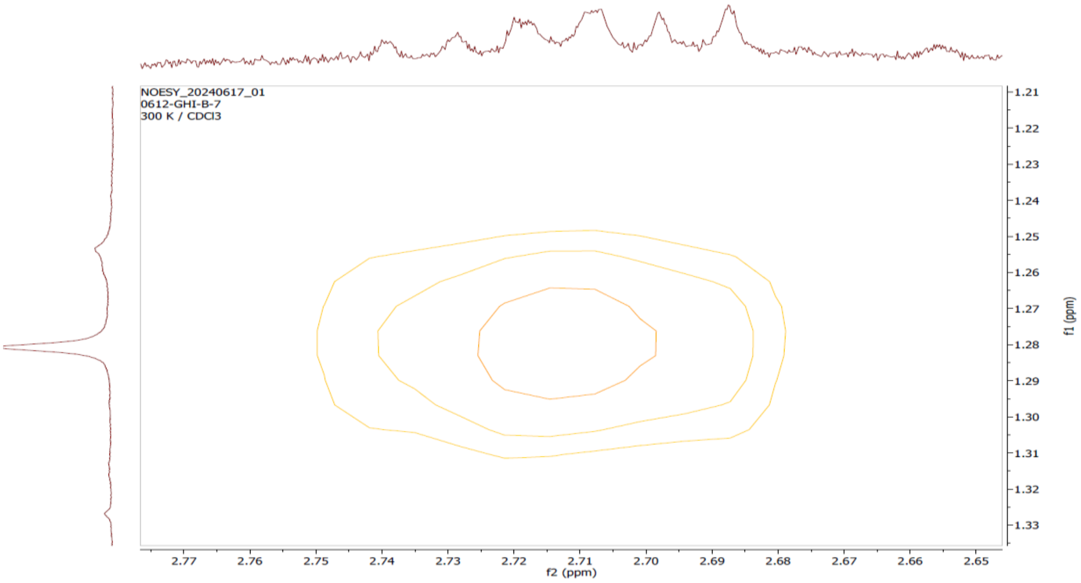

Figure S71. NOESY spectrum of 3-acetoxynorzoanthaminone (2.93-3.03 ppm) at 600 MHz in $CDCl_3$

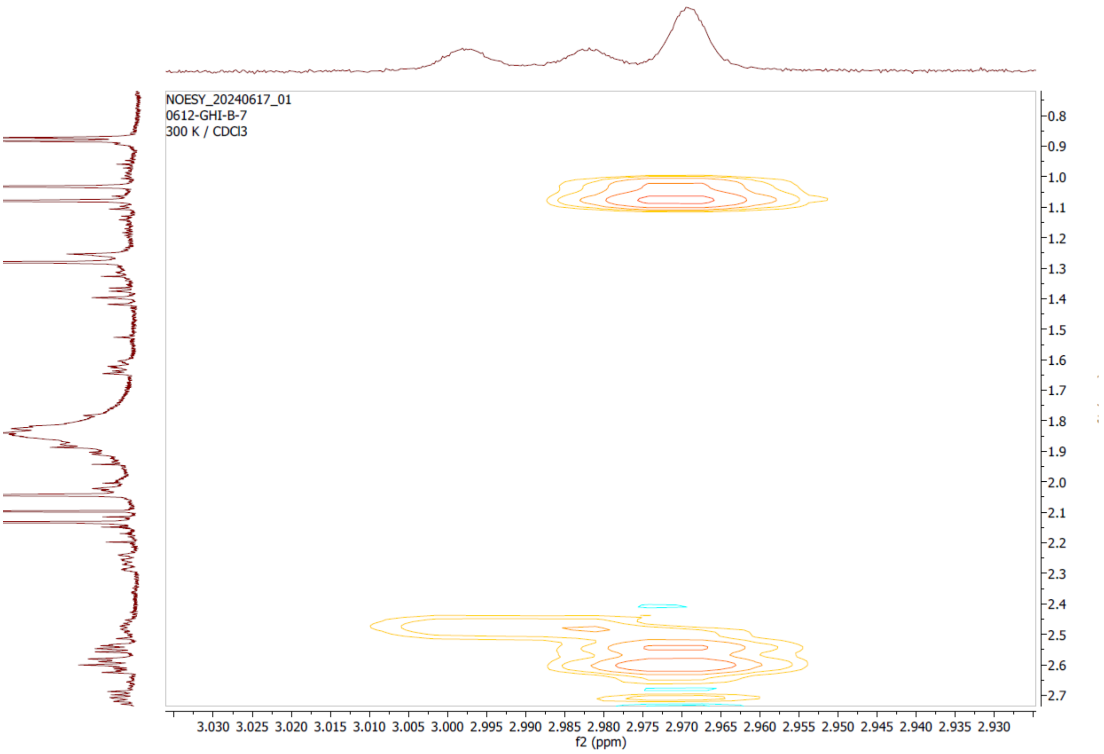

Figure S72. NOESY spectrum of 3-acetoxynorzoanthaminone (2.93-3.03 ppm) at 600 MHz in $CDCl_3$

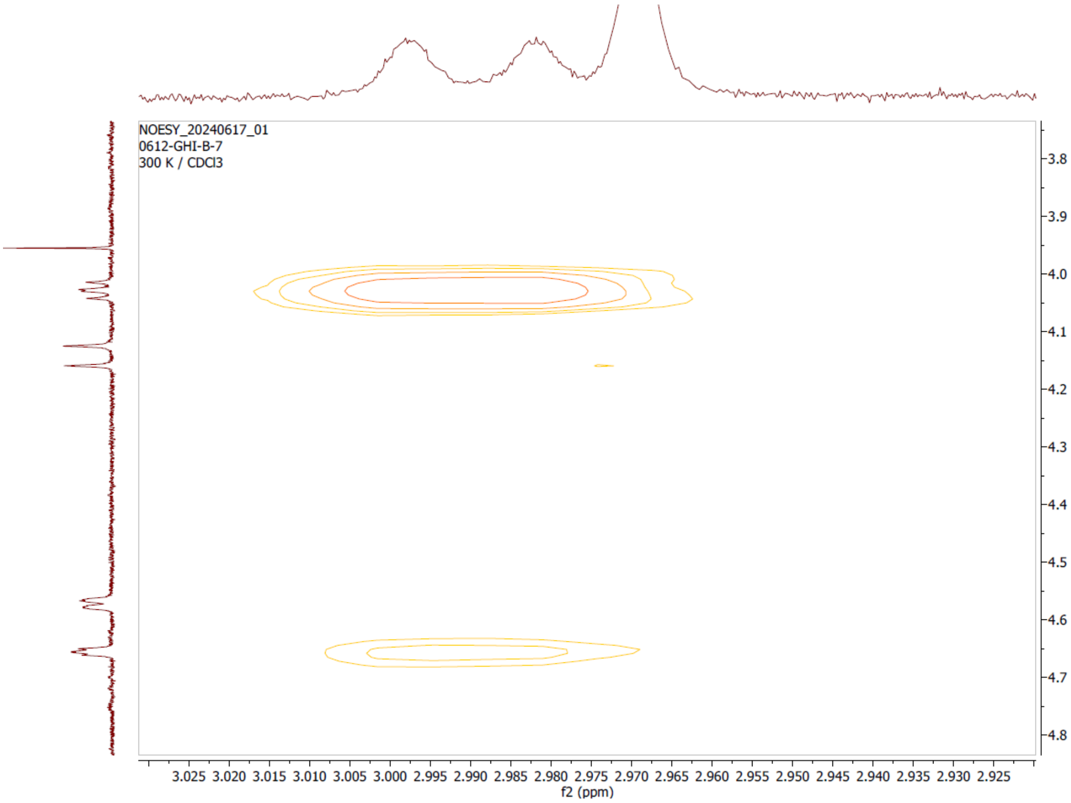

Figure S73. MS spectrum of 3-acetoxynorzoanthaminone (**3**)

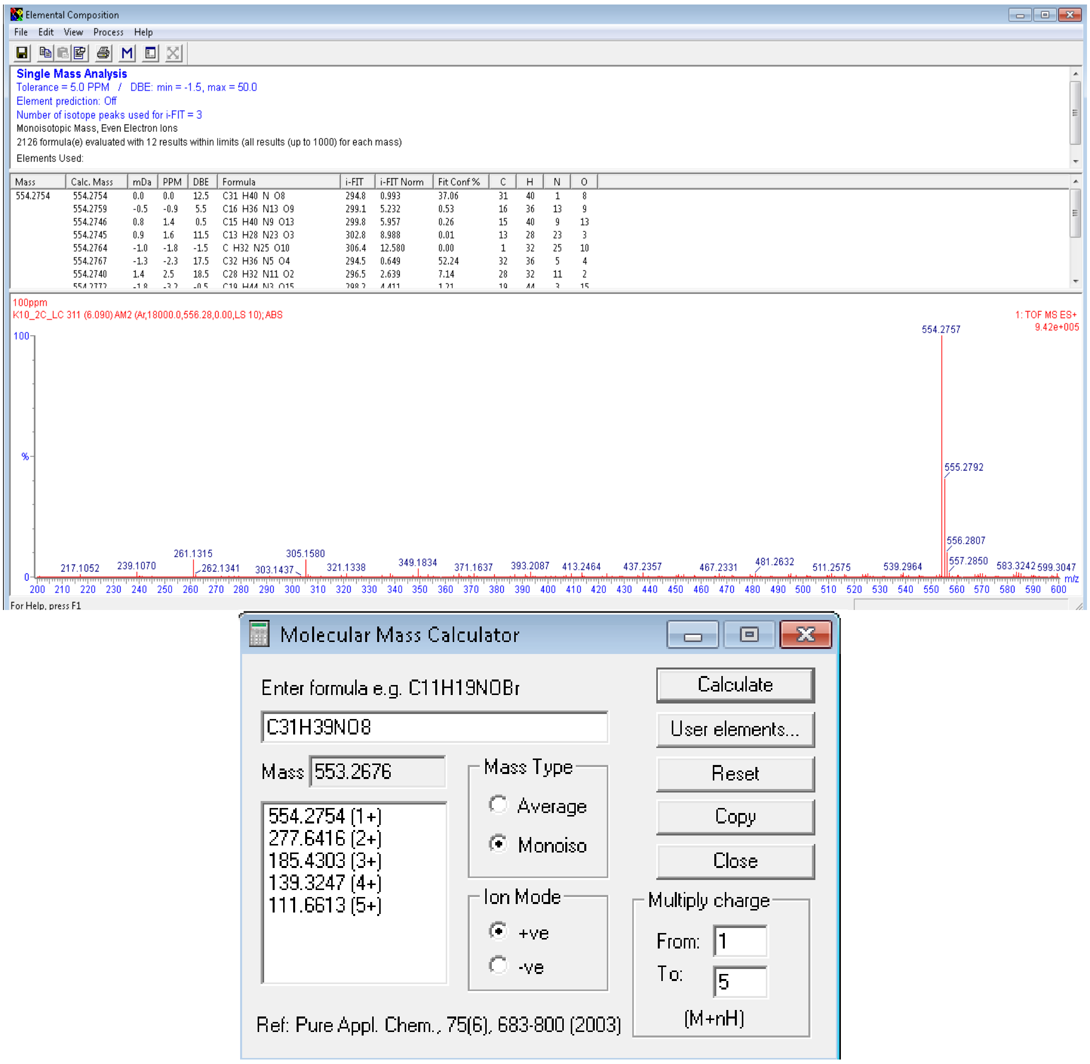

Figure S74. UV spectrum of 3-acetoxynorzoanthaminone (**3**)

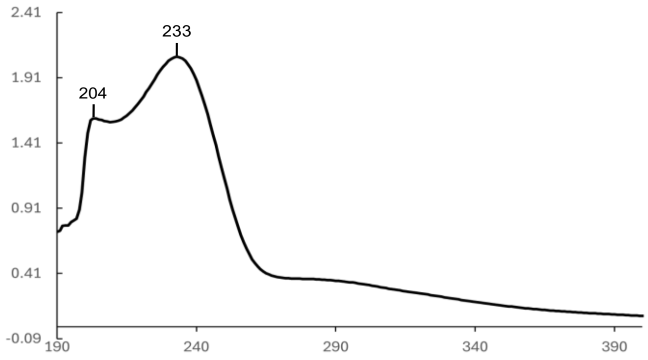

Figure S75. IR (ATR) spectrum of 3-acetoxynorzoanthaminone (**3**)

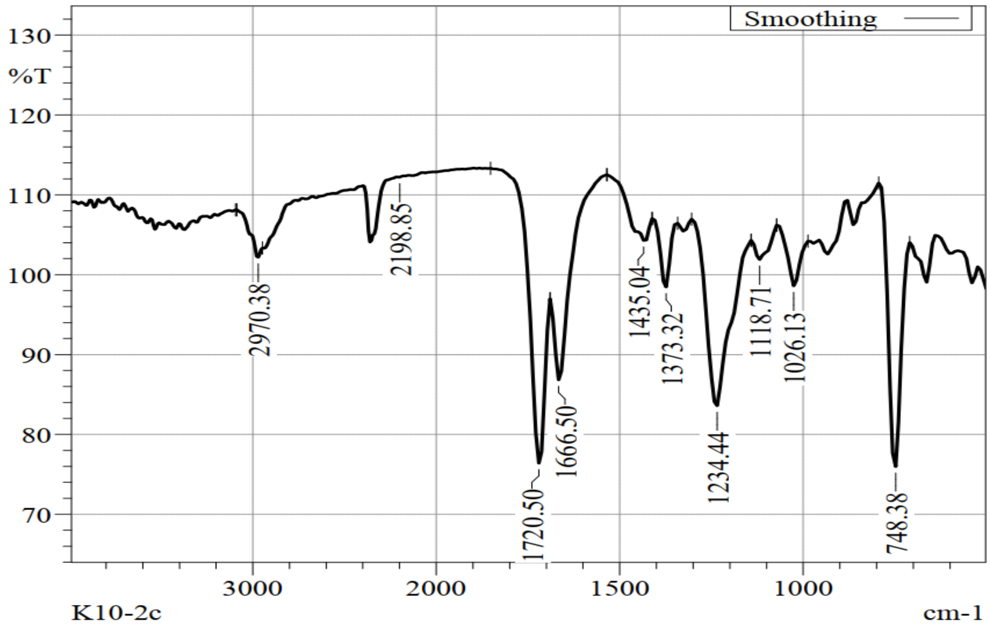

Figure S76. ^{1}H-NMR spectrum of 11-hydroxynorzoanthamide B (**4**) at 600 MHz in $CDCl_3$

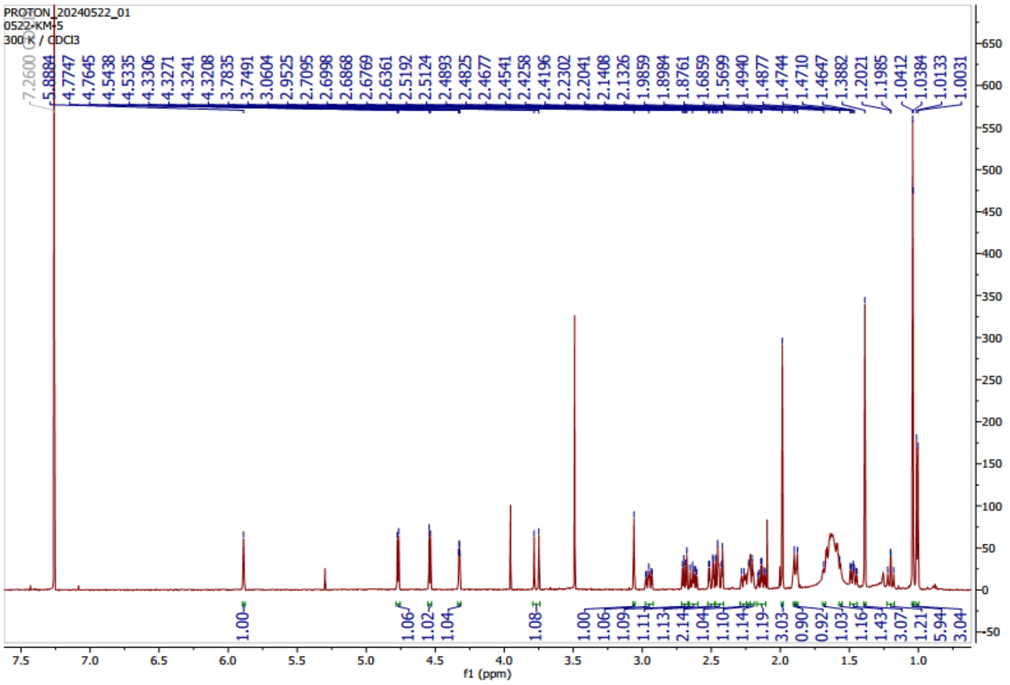

Figure S77. ^{1}H-NMR spectrum of 11-hydroxynorzoanthamide B (3.60-6.30 ppm) at 600 MHz in $CDCl_3$

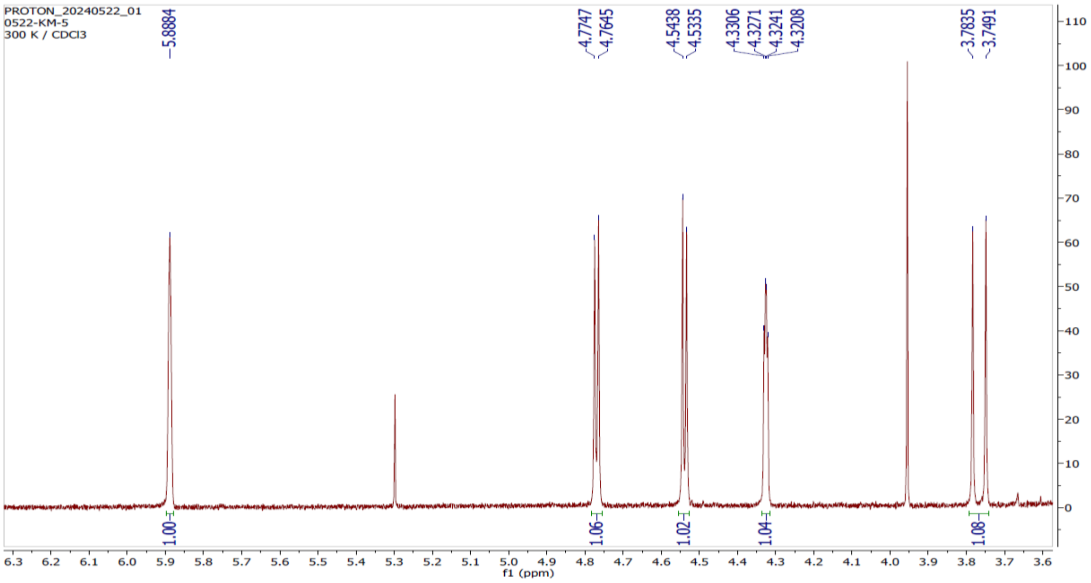

Figure S78. ^{1}H-NMR spectrum of 11-hydroxynorzoanthamide B (0.80-3.20 ppm) at 600 MHz in $CDCl_3$

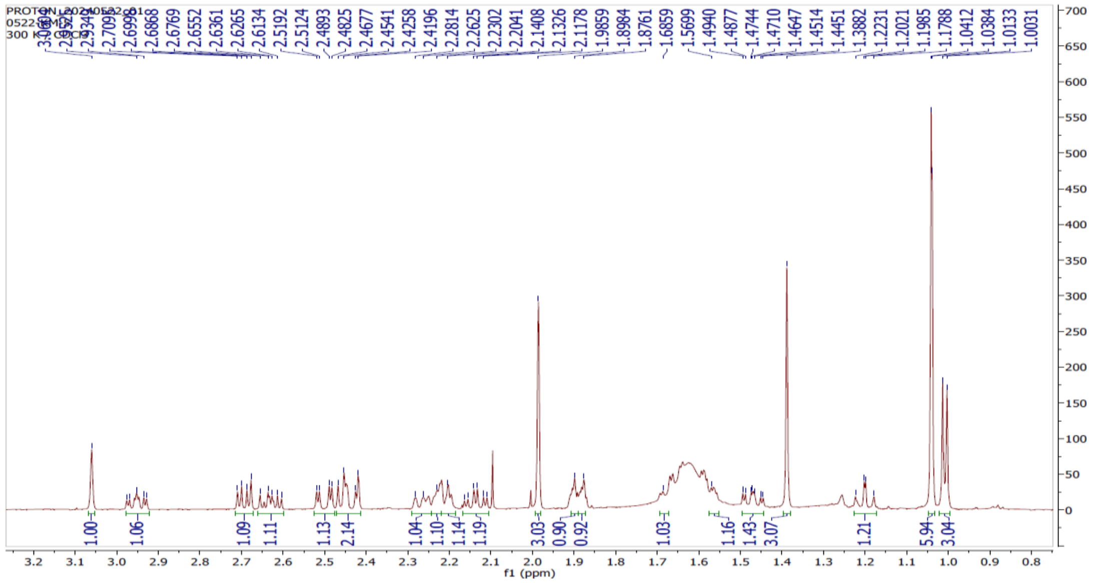

Figure S79. ^{13}C-NMR spectrum of 11-hydroxynorzoanthamide B (**4**) at 150 MHz in $CDCl_3$

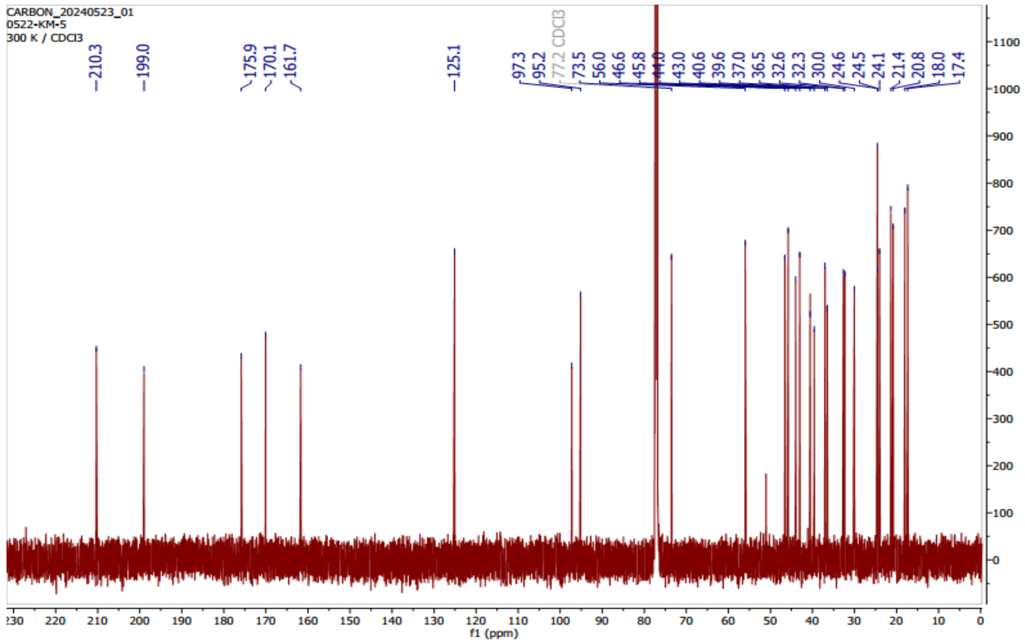

Figure S80. ^{13}C-NMR spectrum of 11-hydroxynorzoanthamide B (105-225 ppm) at 150 MHz in $CDCl_3$

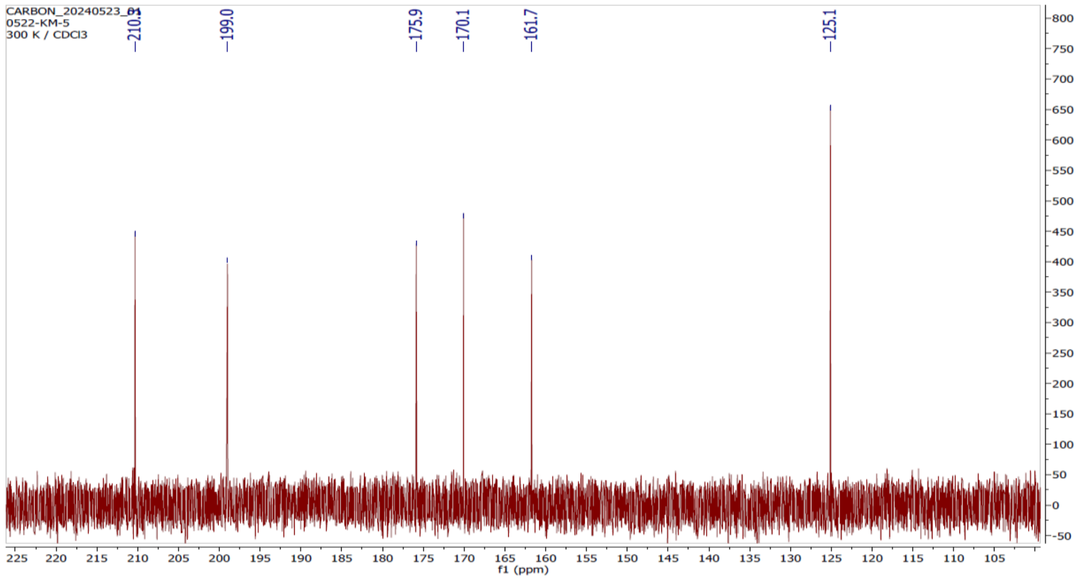

Figure S81. ^{1}C-NMR spectrum of 11-hydroxynorzoanthamide B (15-100 ppm) at 150 MHz in $CDCl_3$

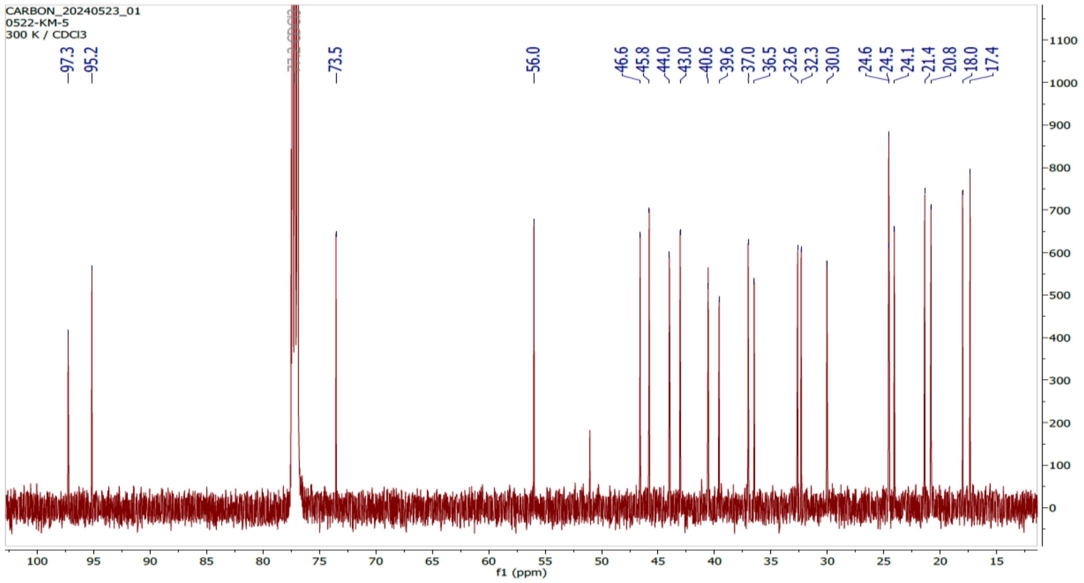

Figure S82. COSY spectrum of 11-hydroxynorzoanthamide B (**4**) at 600 MHz in $CDCl_3$

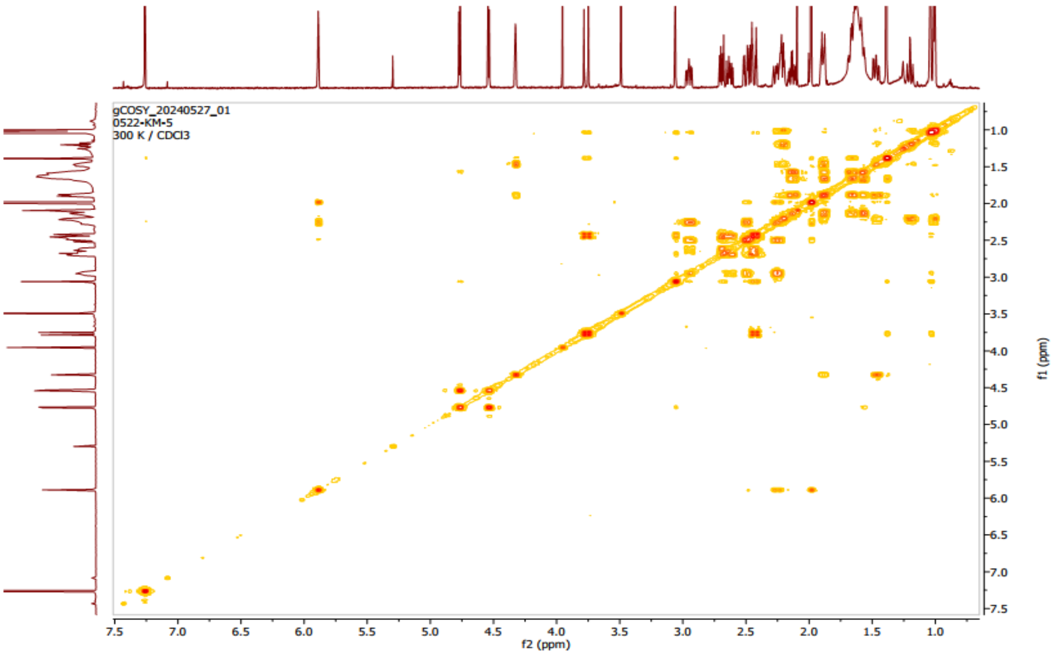

Figure S83. COSY spectrum of 11-hydroxynorzoanthamide B (3.70-4.90 ppm) at 600 MHz in $CDCl_3$

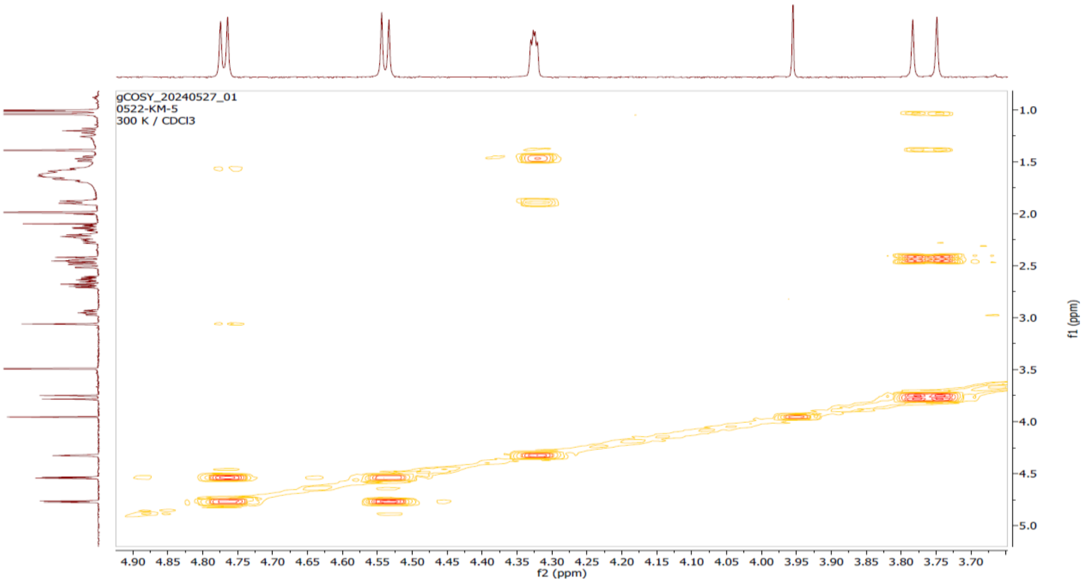

Figure S84. COSY spectrum of 11-hydroxynorzoanthamide B (3.70-4.90 ppm) at 600 MHz in $CDCl_3$

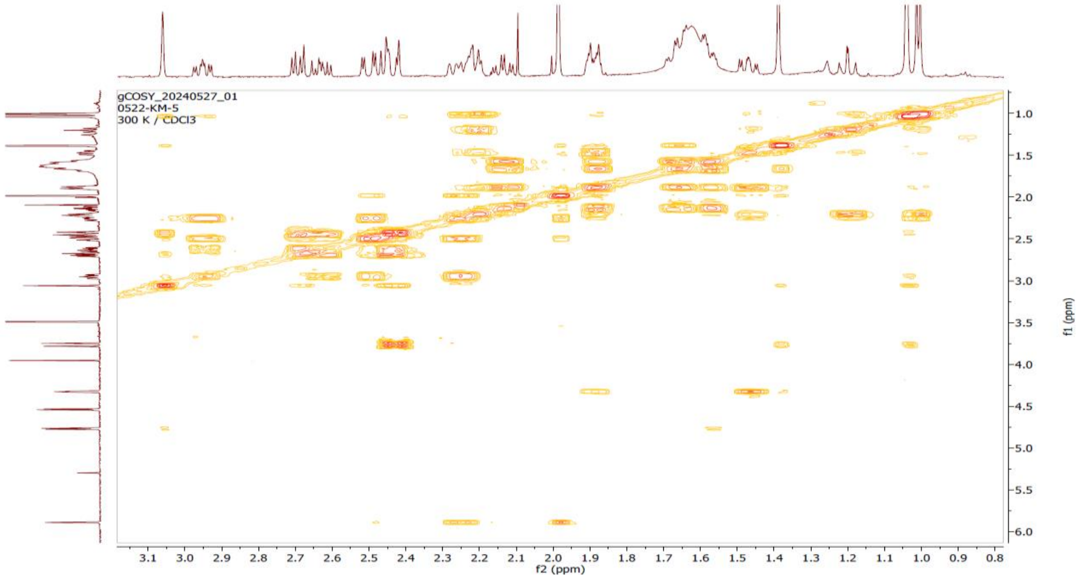

Figure S85. HSQC spectrum of 11-hydroxynorzoanthamide B (**4**) at 600 and 150 MHz in $CDCl_3$

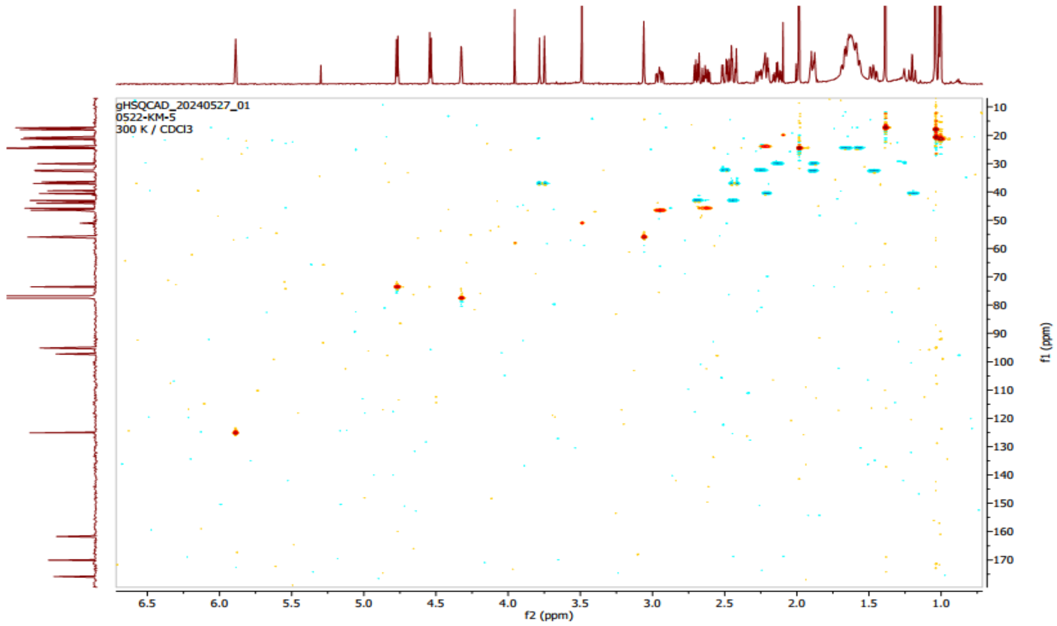

Figure S86. HSQC spectrum of 11-hydroxynorzoanthamide B (4.2-6.1 ppm) at 600 and 150 MHz in $CDCl_3$

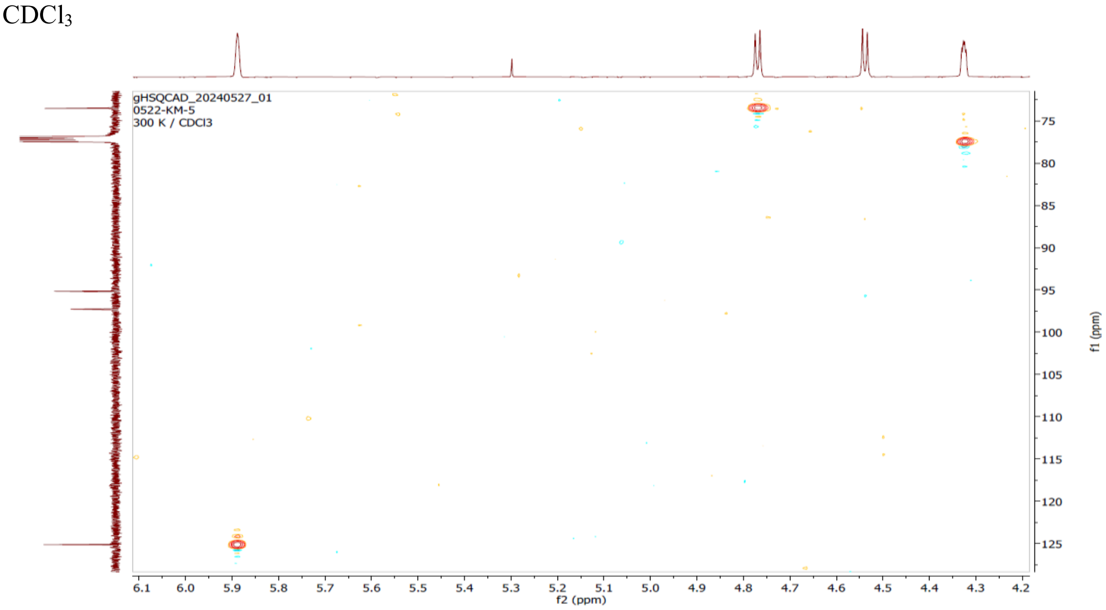

Figure S87. HSQC spectrum of 11-hydroxynorzoanthamide B (0.9-4.0 ppm) at 600 and 150 MHz in $CDCl_3$

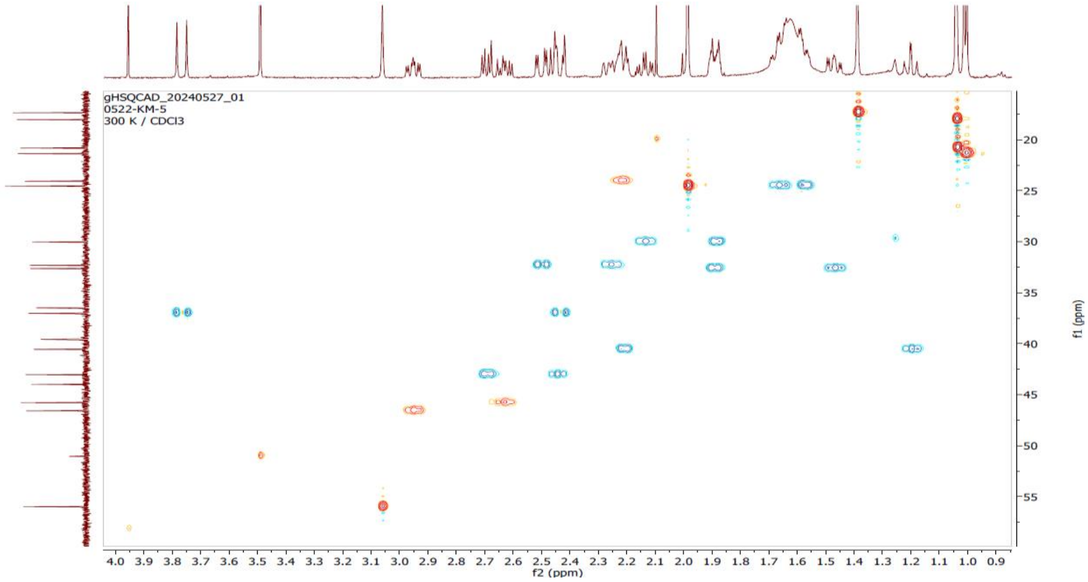

Figure S88. HMBC spectrum of 11-hydroxynorzoanthamide B (**4**) at 600 and 150 MHz in $CDCl_3$

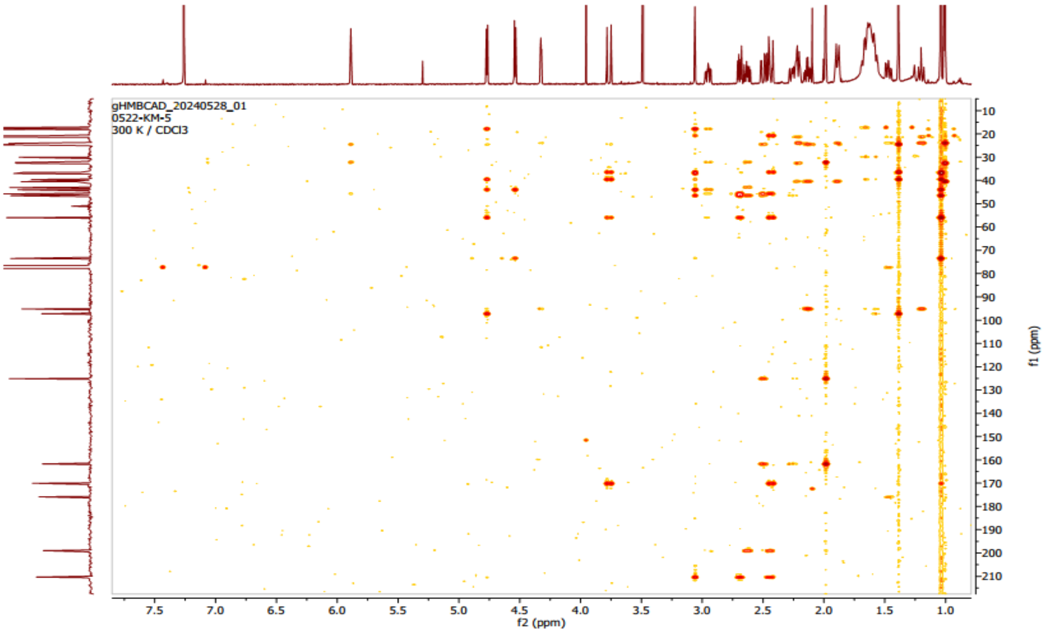

Figure S89. HMBC spectrum of 11-hydroxynorzoanthamide B (4.3-6.0 ppm) at 600 and 150 MHz in $CDCl_3$

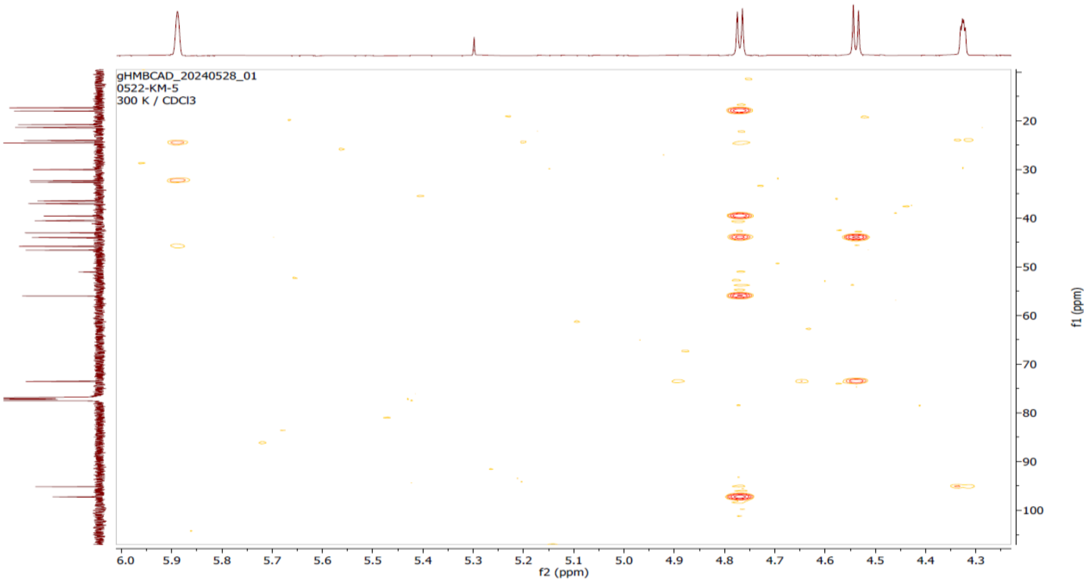

Figure S90. HMBC spectrum of 11-hydroxynorzoanthamide B (1.0-4.0 ppm) at 600 and 150 MHz in $CDCl_3$

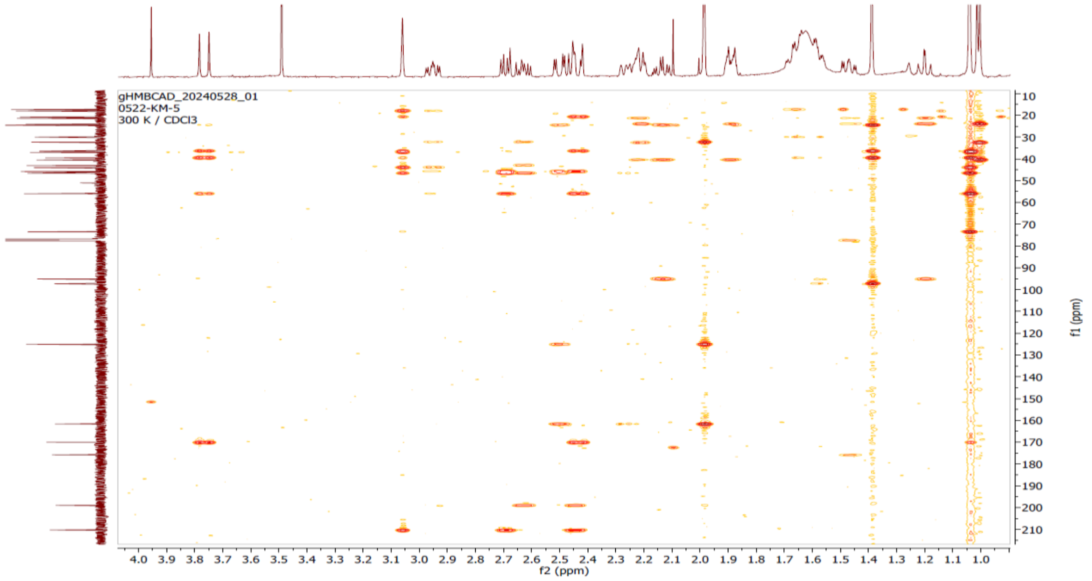

Figure S91. NOESY spectrum of 11-hydroxynorzoanthamide B (**4**) at 600 MHz in $CDCl_3$

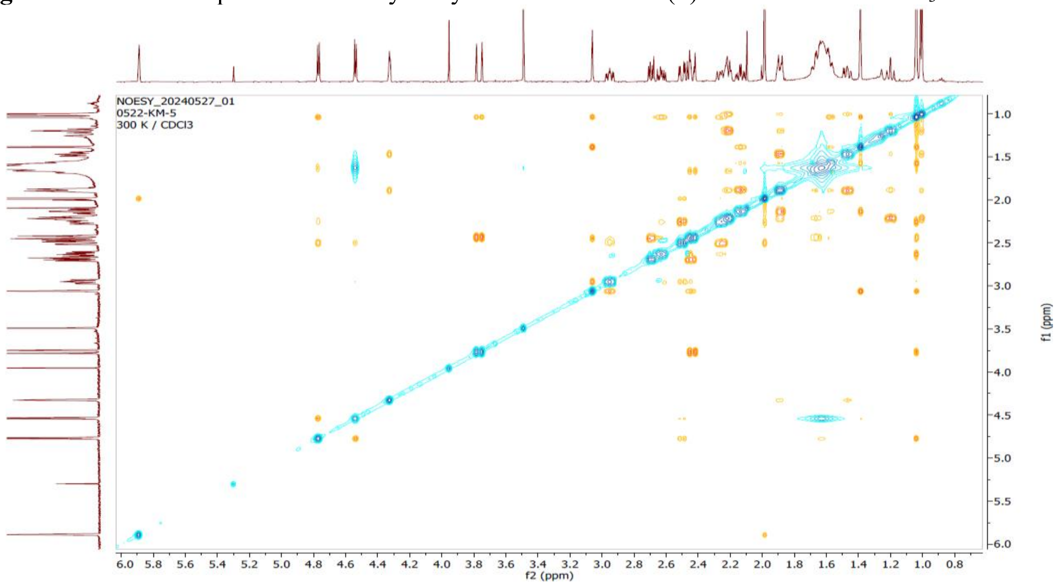

Figure S92. NOESY spectrum of 11-hydroxynorzoanthamide B (4.7-4.8 ppm) at 600 MHz in $CDCl_3$

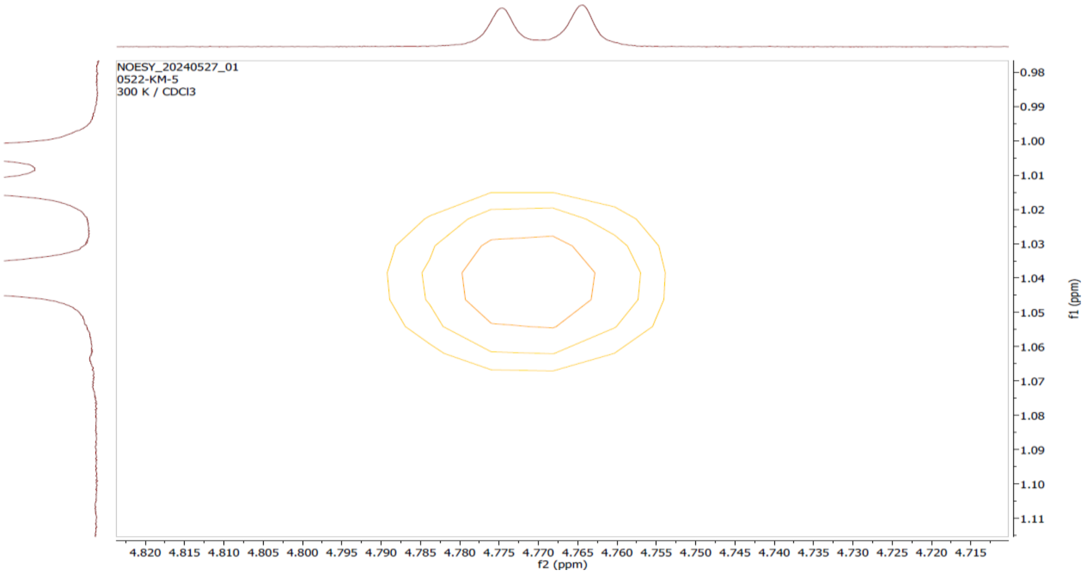

Figure S93. NOESY spectrum of 11-hydroxynorzoanthamide B (3.0-3.2 ppm) at 600 MHz in $CDCl_3$

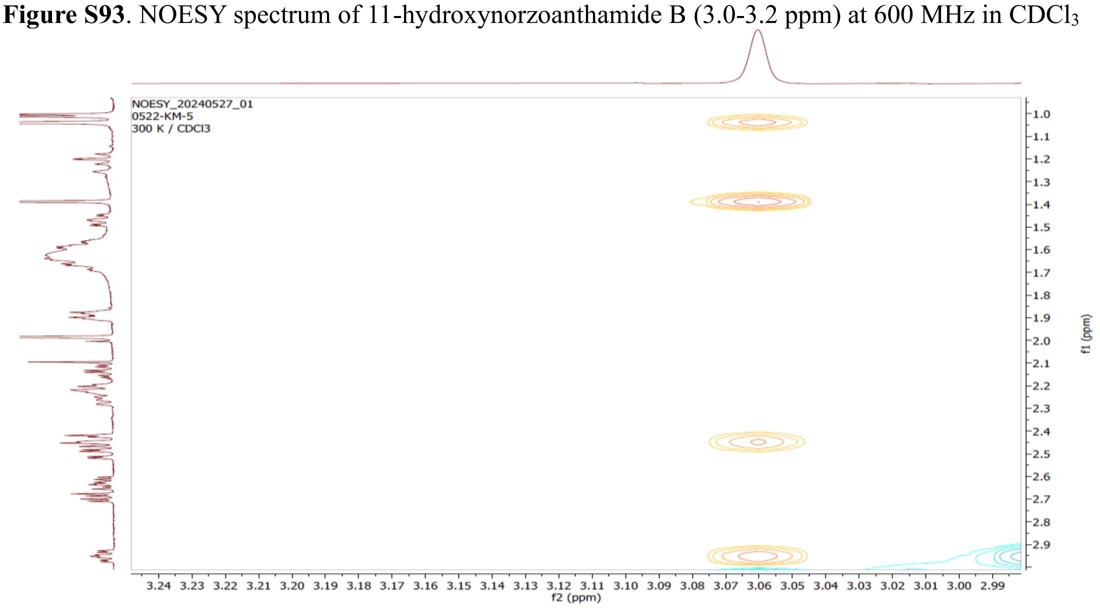

Figure S94. NOESY spectrum of 11-hydroxynorzoanthamide B (1.0-1.17 ppm) at 600 MHz in $CDCl_3$

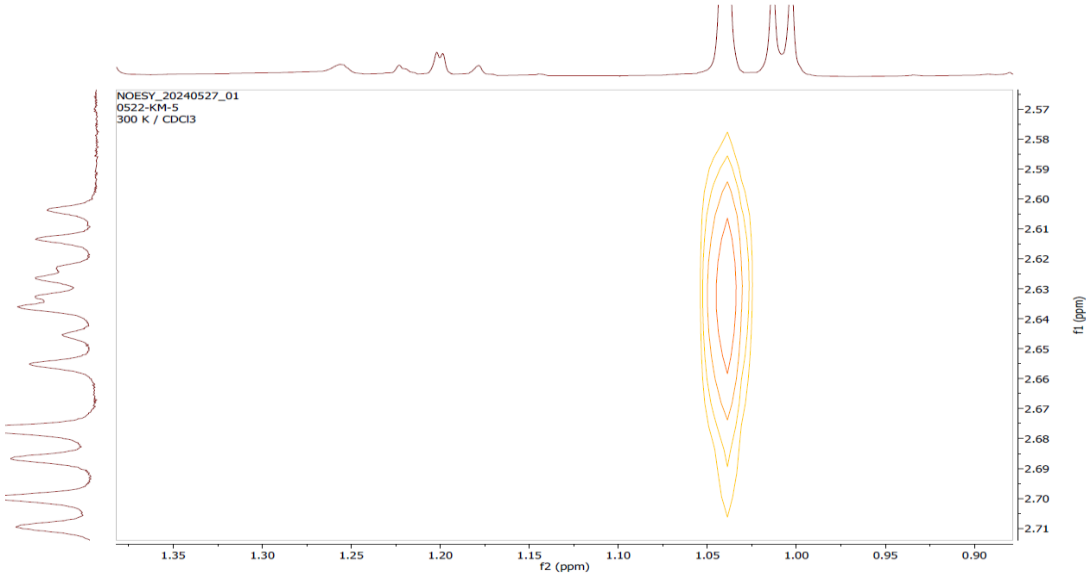

Figure S95. MS spectrum of 11-hydroxynorzoanthamide B (**4**)

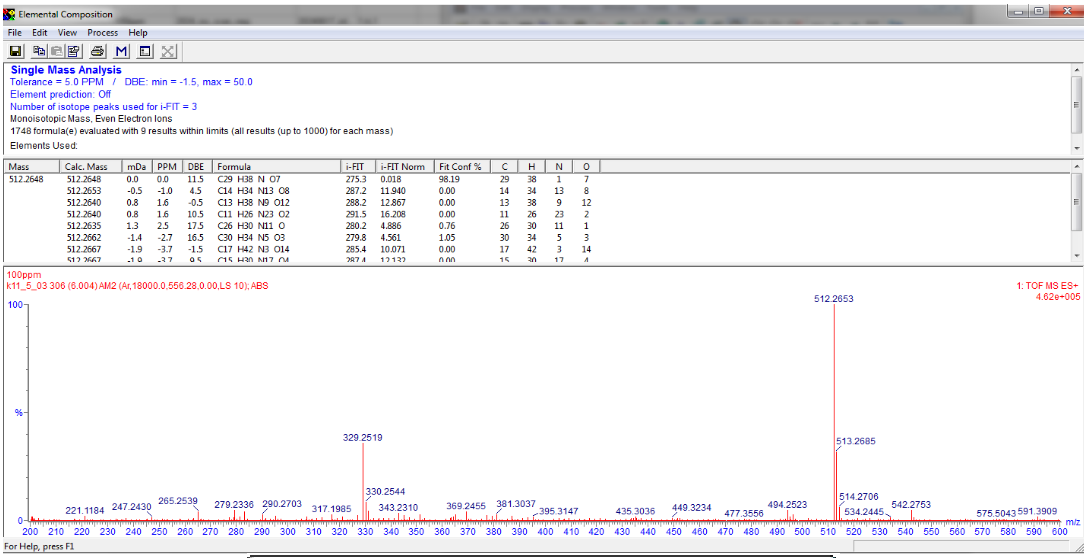

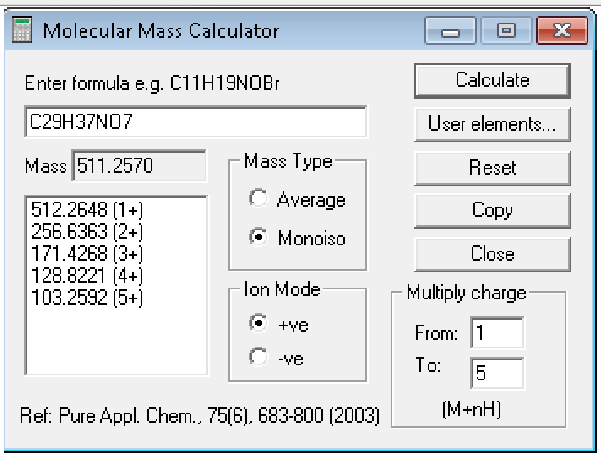

Figure S96. UV spectrum of 11-hydroxynorzoanthamide B (**4**)

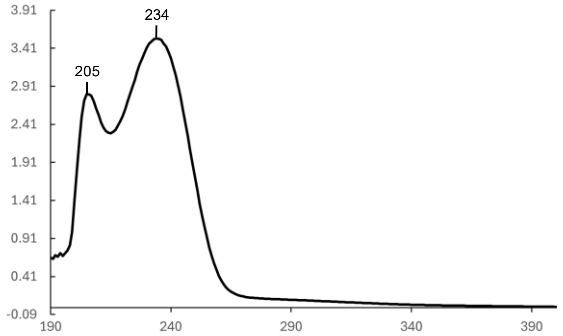

Figure S97. IR (ATR) spectrum of 11-hydroxynorzoanthamide B (**4**)

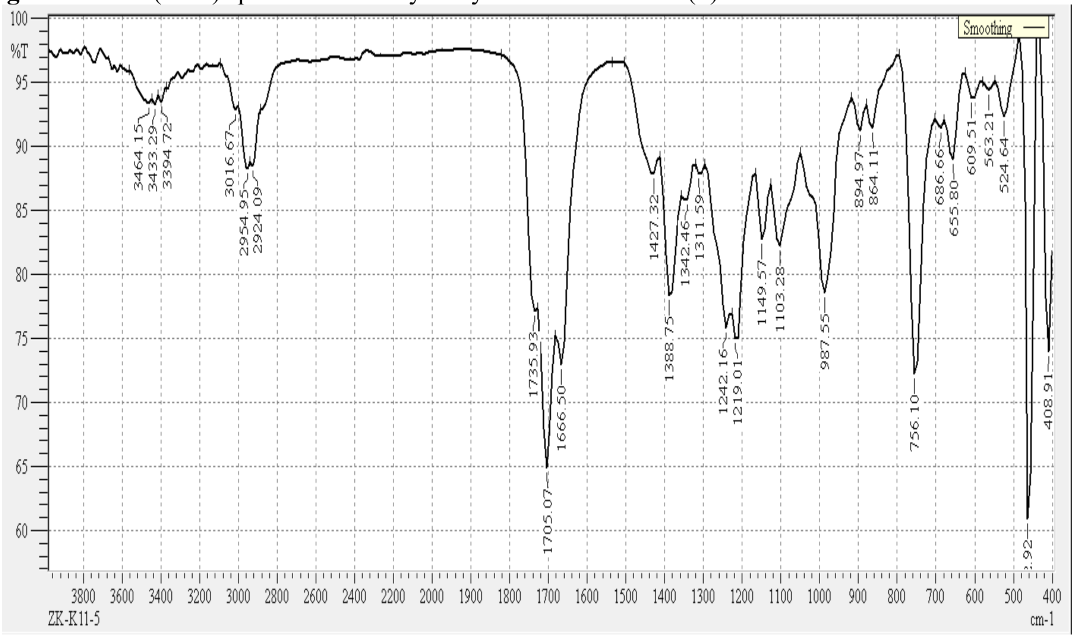

Figure S98. ^{1}H-NMR spectrum of 11-hydroxyzoanthamide B (**5**) at 800 MHz in $CDCl_3$

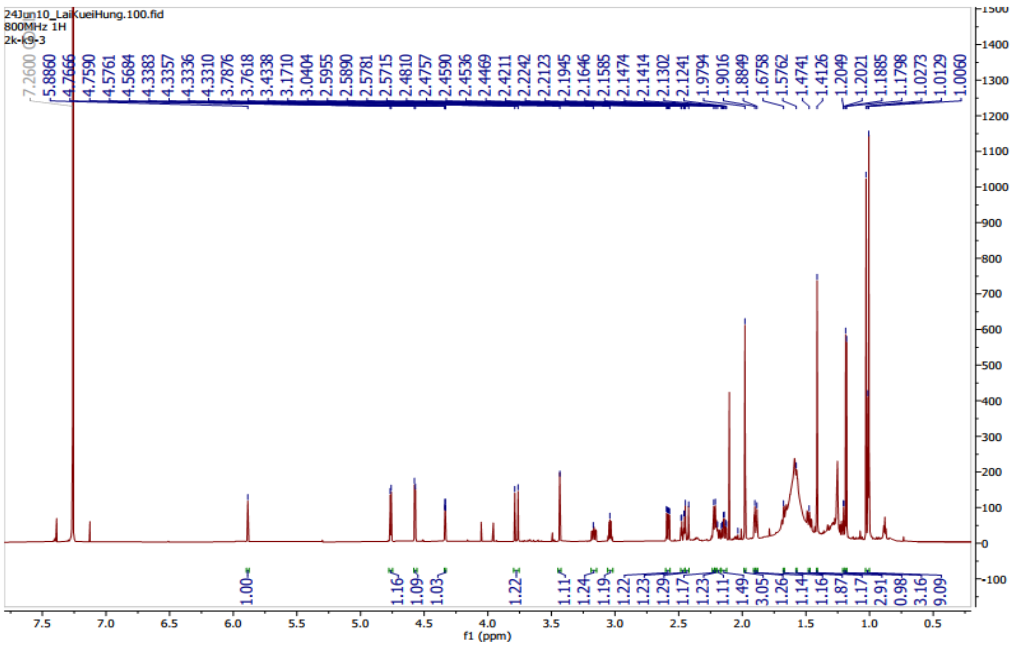

Figure S99. ^{1}H-NMR spectrum of 11-hydroxyzoanthamide B (3.0-6.2 ppm) at 800 MHz in $CDCl_3$

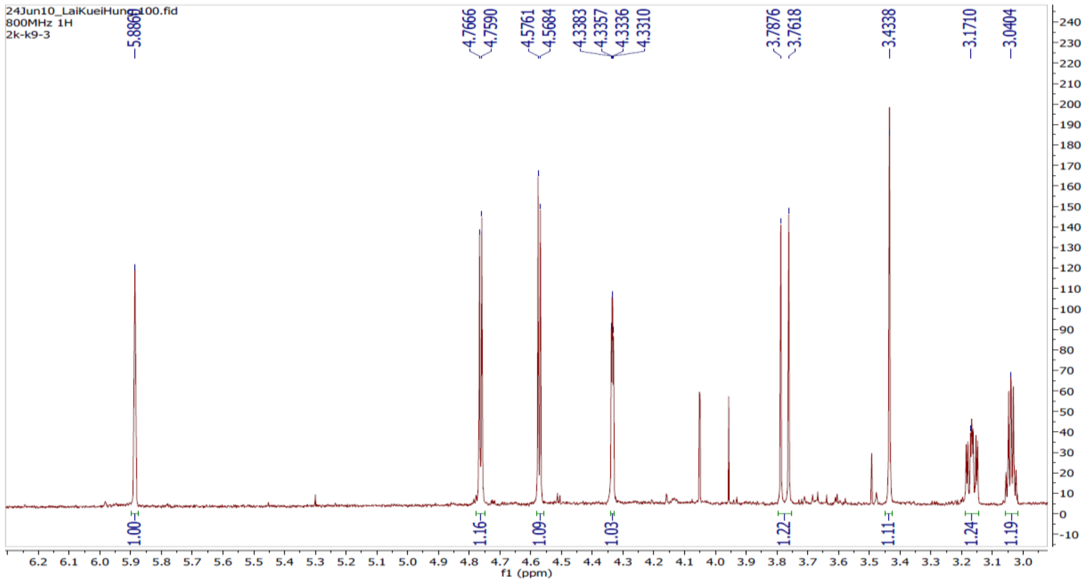

Figure S100. ^{1}H-NMR spectrum of 11-hydroxyzoanthamide B (1.0-2.7 ppm) at 800 MHz in $CDCl_3$

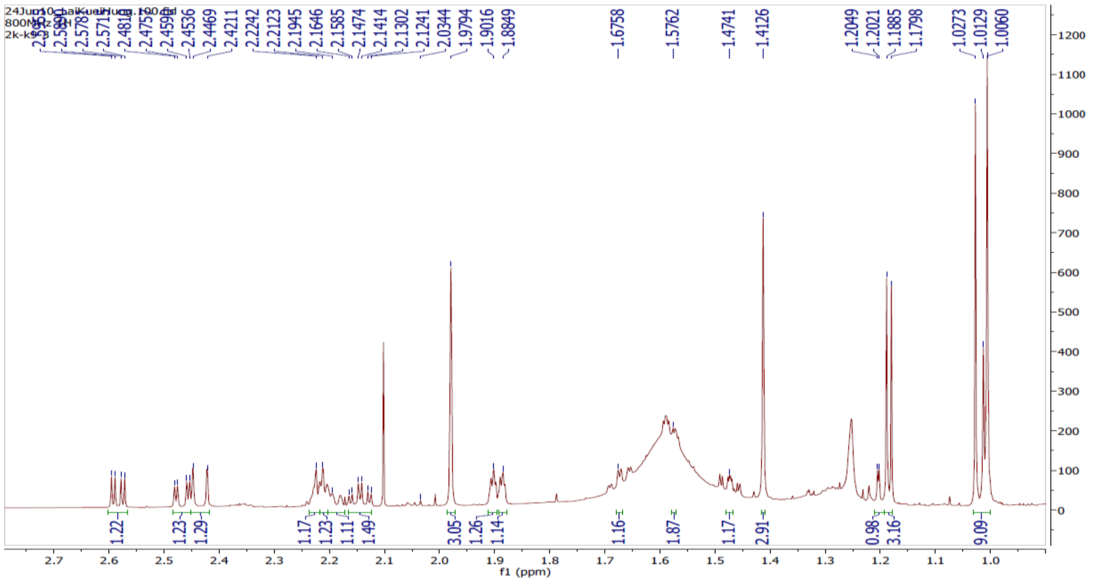

Figure S101. ^{13}C-NMR spectrum of 11-hydroxyzoanthamide B (**5**) at 200 MHz in $CDCl_3$

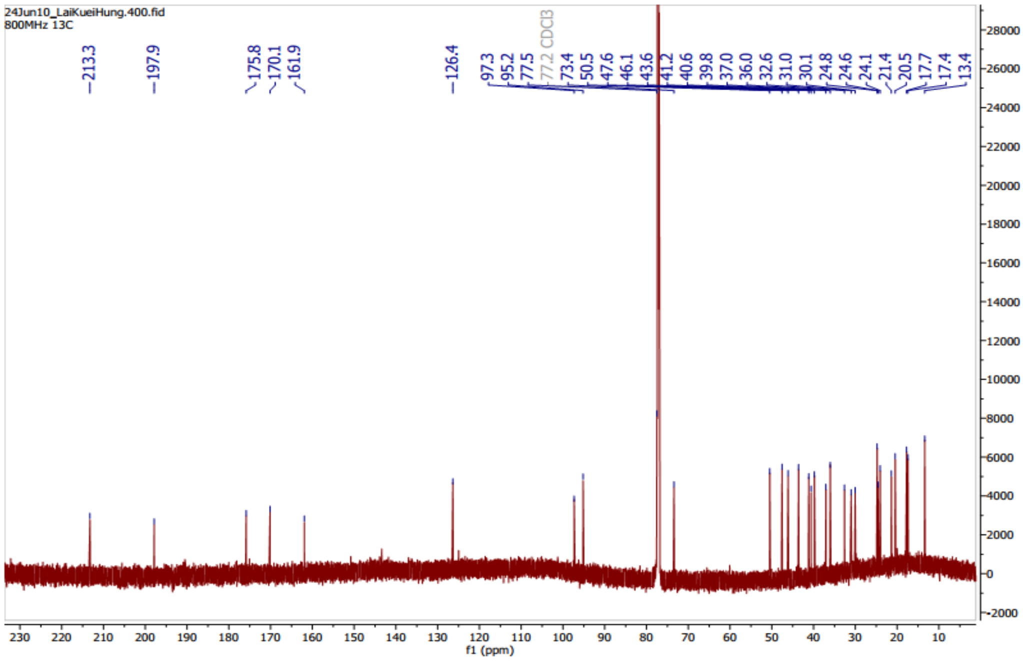

Figure S102. ^{13}C-NMR spectrum of 11-hydroxyzoanthamide B (95-220 ppm) at 200 MHz in $CDCl_3$

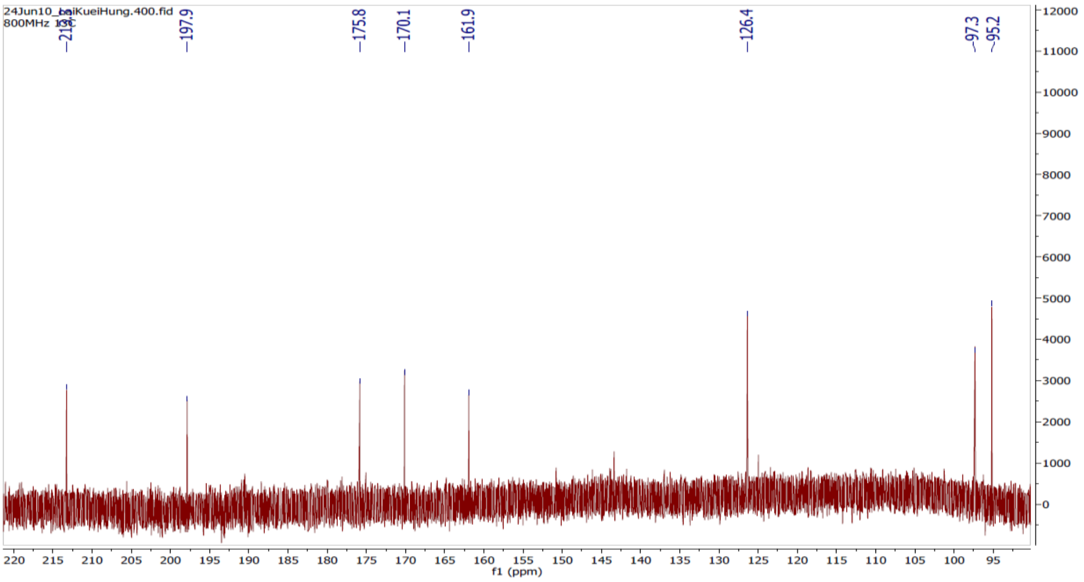

Figure S103. ^{13}C-NMR spectrum of 11-hydroxyzoanthamide B (10-80 ppm) at 200 MHz in $CDCl_3$

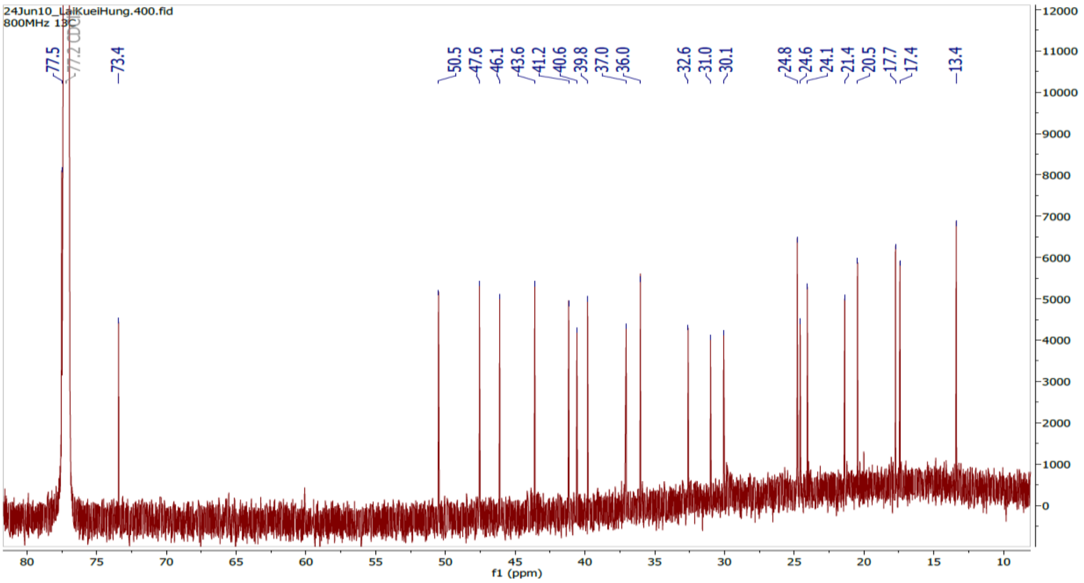

Figure S104. COSY spectrum of 11-hydroxyzoanthamide B (**5**) at 800 MHz in $CDCl_3$

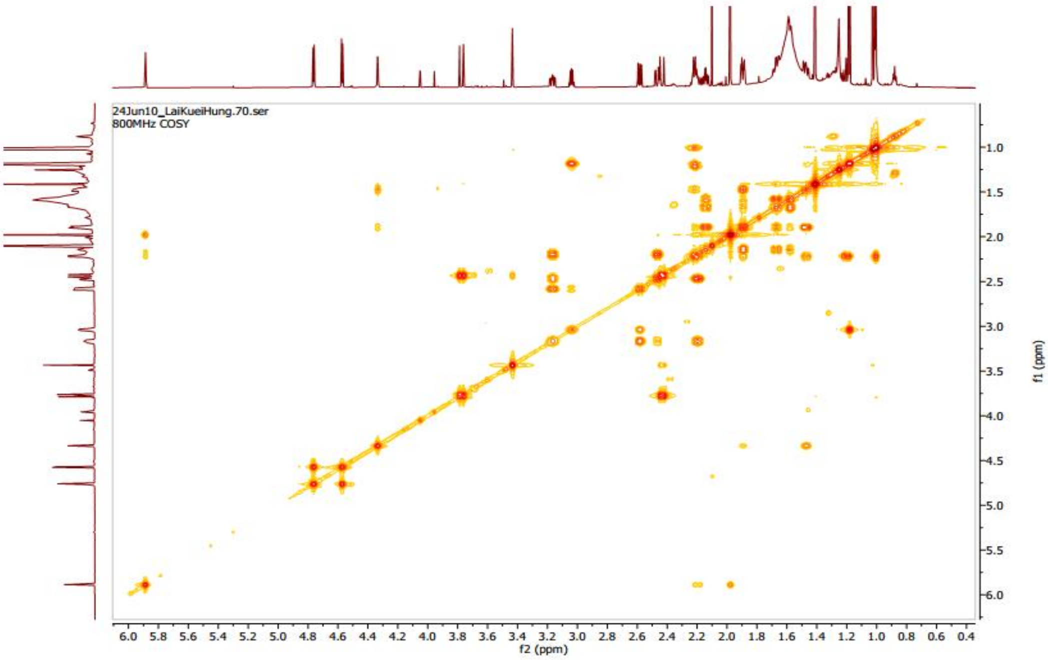

Figure S105. COSY spectrum of 11-hydroxyzoanthamide B (2.9-4.9 ppm) at 800 MHz in $CDCl_3$

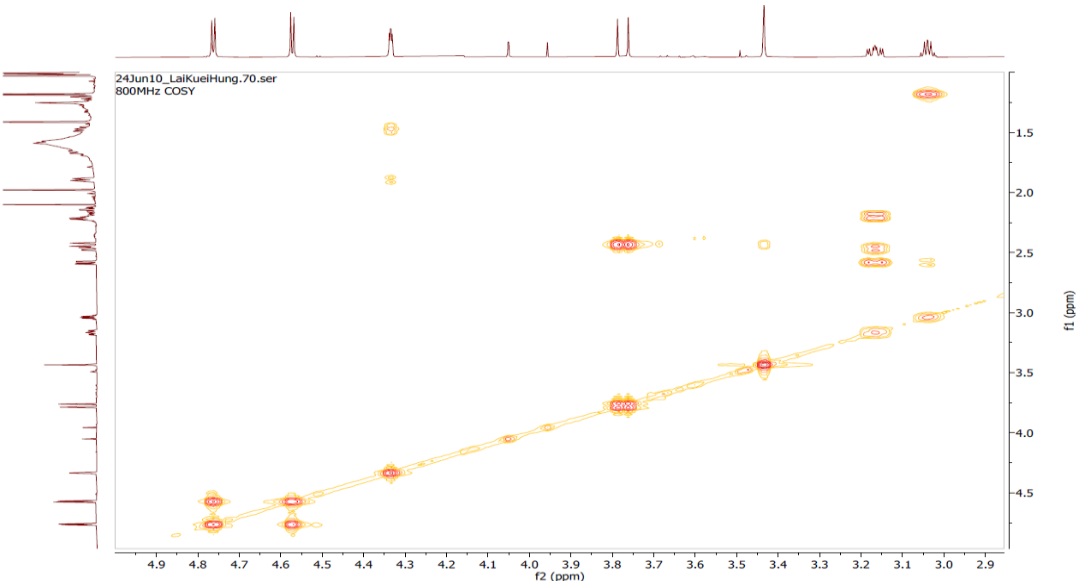

Figure S106. COSY spectrum of 11-hydroxyzoanthamide B (0.6-2.6 ppm) at 800 MHz in $CDCl_3$

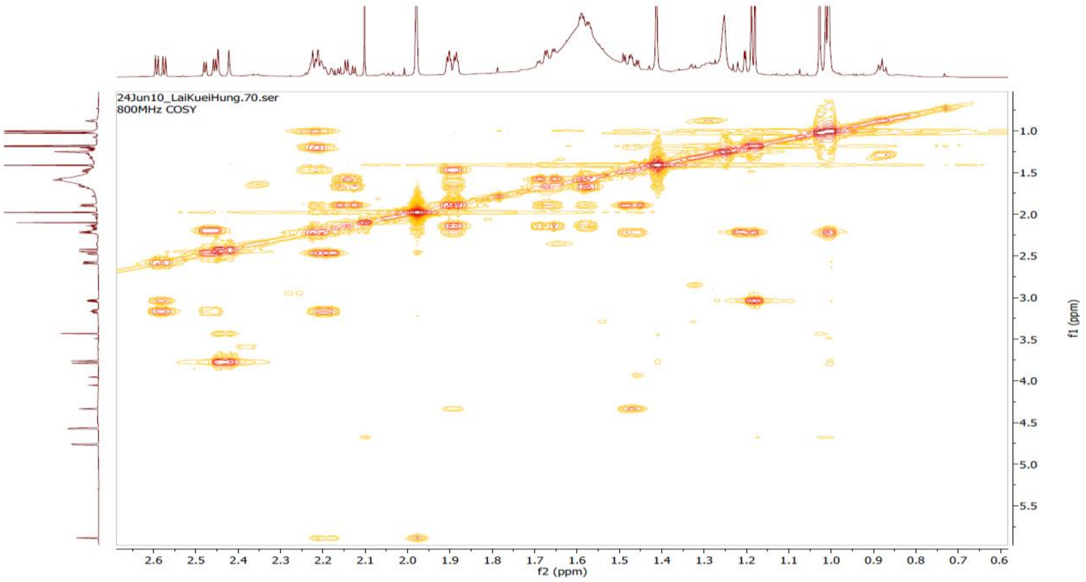

Figure S107. HSQC spectrum of 11-hydroxyzoanthamide B (**5**) at 800 and 200 MHz in $CDCl_3$

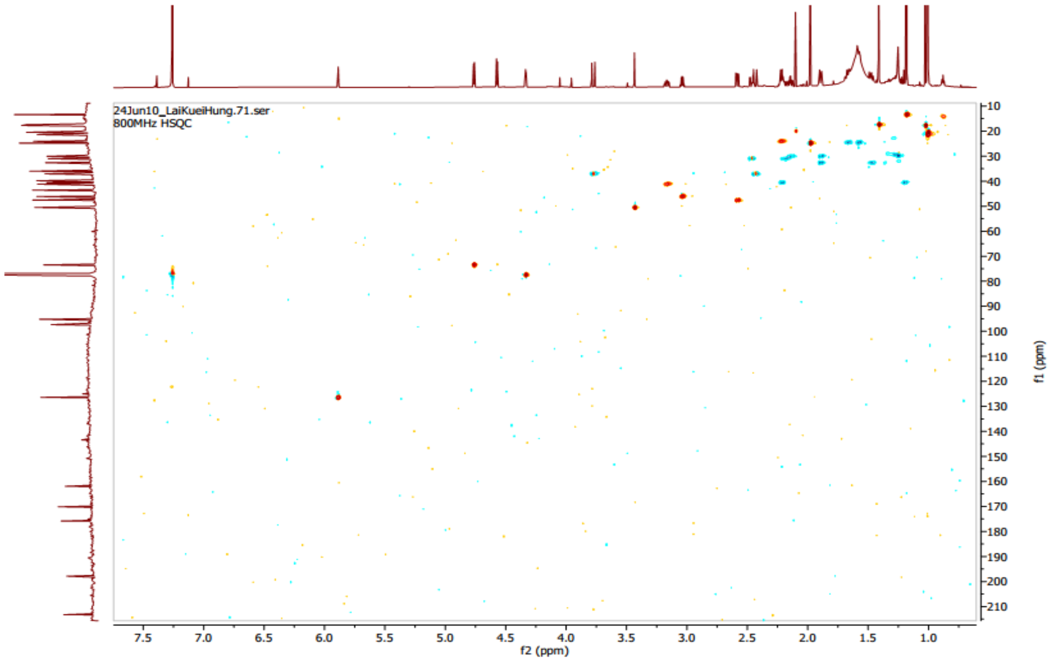

Figure S108. HSQC spectrum of 11-hydroxyzoanthamide B (4.3-5.9 ppm) at 800 and 200 MHz in $CDCl_3$

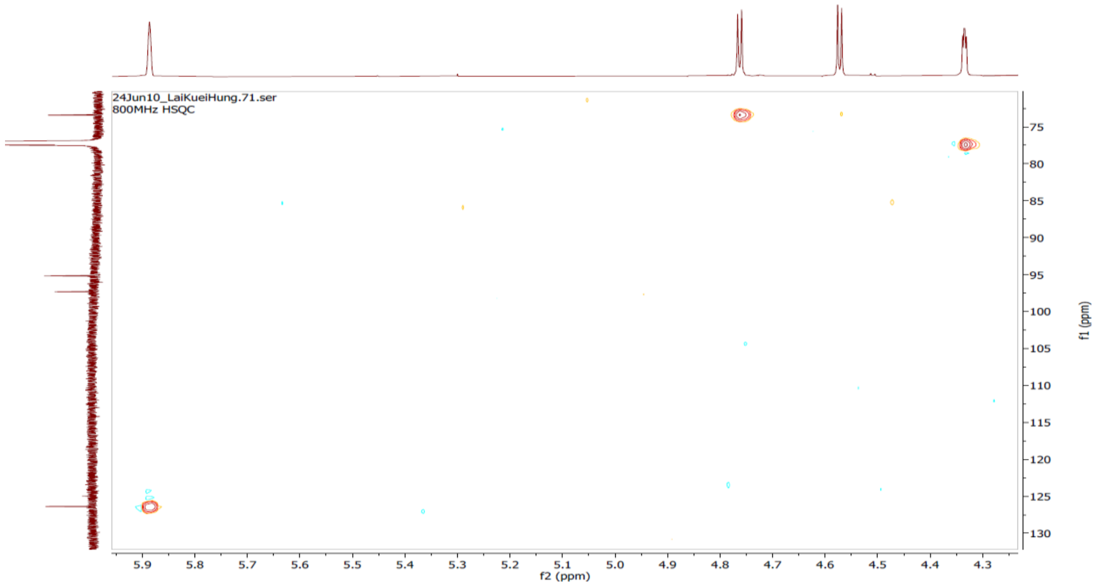

Figure S109. HSQC spectrum of 11-hydroxyzoanthamide B (4.3-5.9 ppm) at 800 and 200 MHz in $CDCl_3$

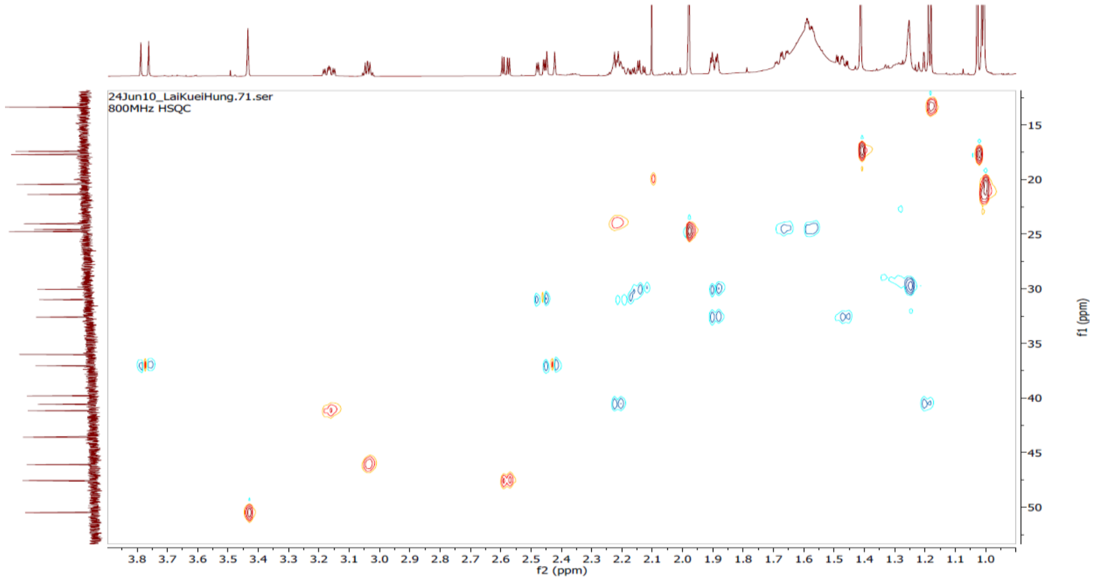

Figure S110. HMBC spectrum of 11-hydroxyzoanthamide B (**5**) at 800 and 200 MHz in $CDCl_3$

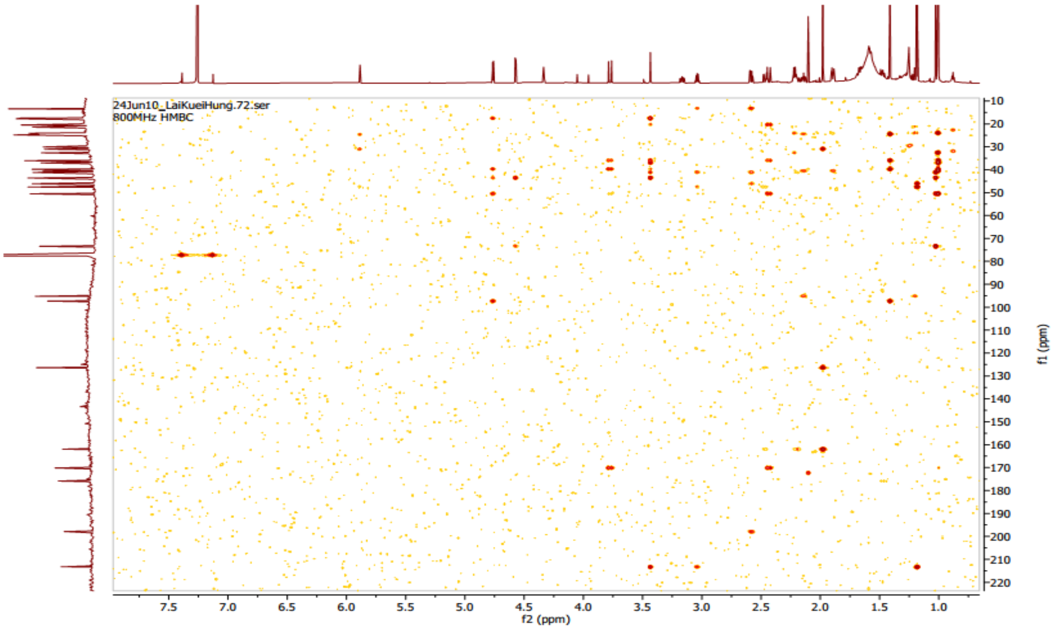

Figure S111. HMBC spectrum of 11-hydroxyzoanthamide B (4.5-5.9 ppm) at 800 and 200 MHz in $CDCl_3$

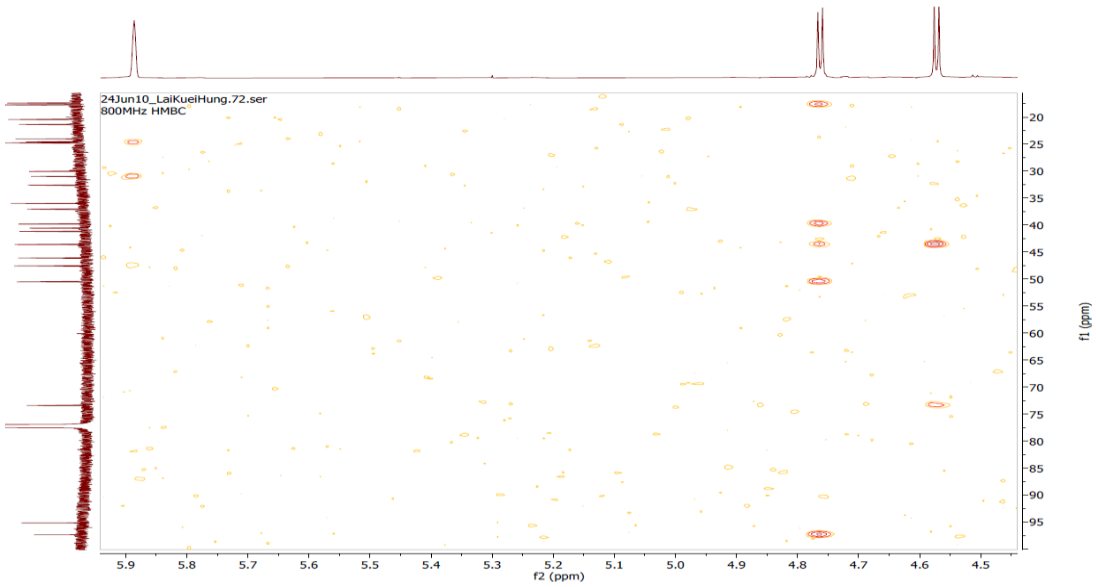

Figure S112. HMBC spectrum of 11-hydroxyzoanthamide B (1.8-3.8 ppm) at 800 and 200 MHz in $CDCl_3$

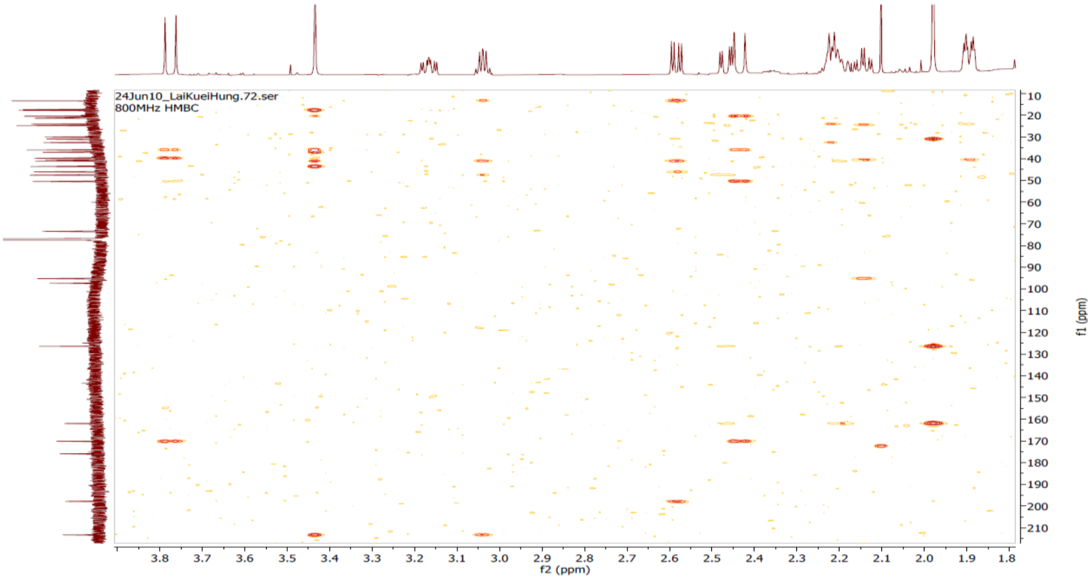

Figure S113. HMBC spectrum of 11-hydroxyzoanthamide B (0.96-1.46 ppm) at 800 and 200 MHz in $CDCl_3$

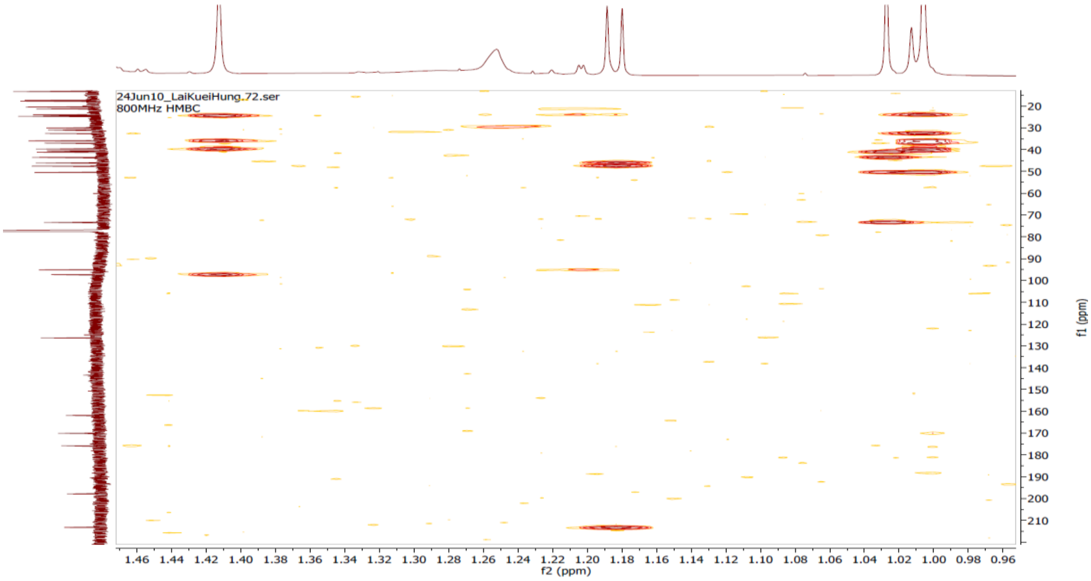

Figure S114. NOESY spectrum of 11-hydroxyzoanthamide B (**5**) at 800 MHz in $CDCl_3$

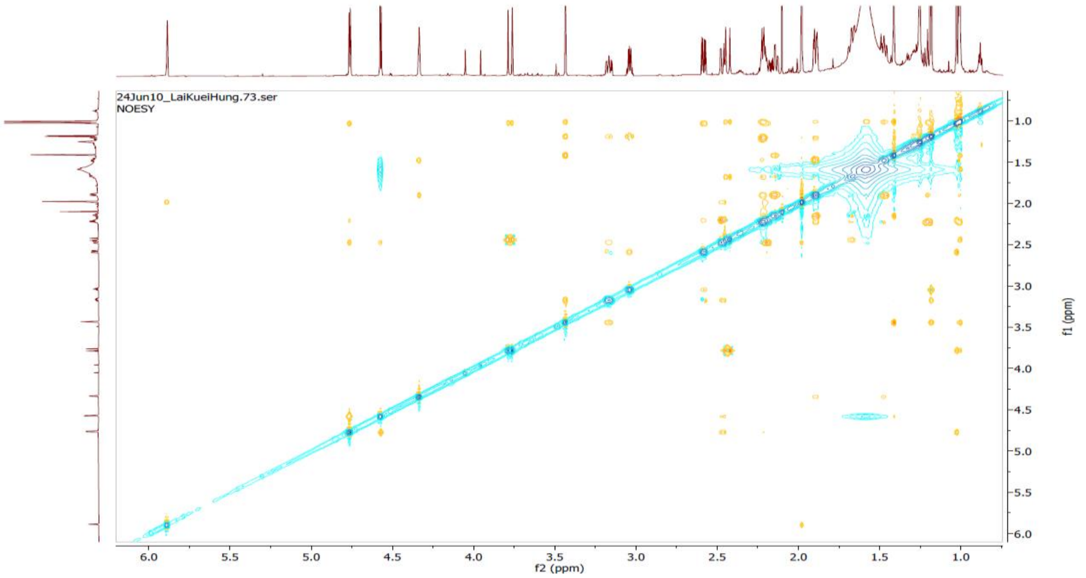

Figure S115. NOESY spectrum of 11-hydroxyzoanthamide B (4.68-4.84 ppm) at 800 MHz in $CDCl_3$

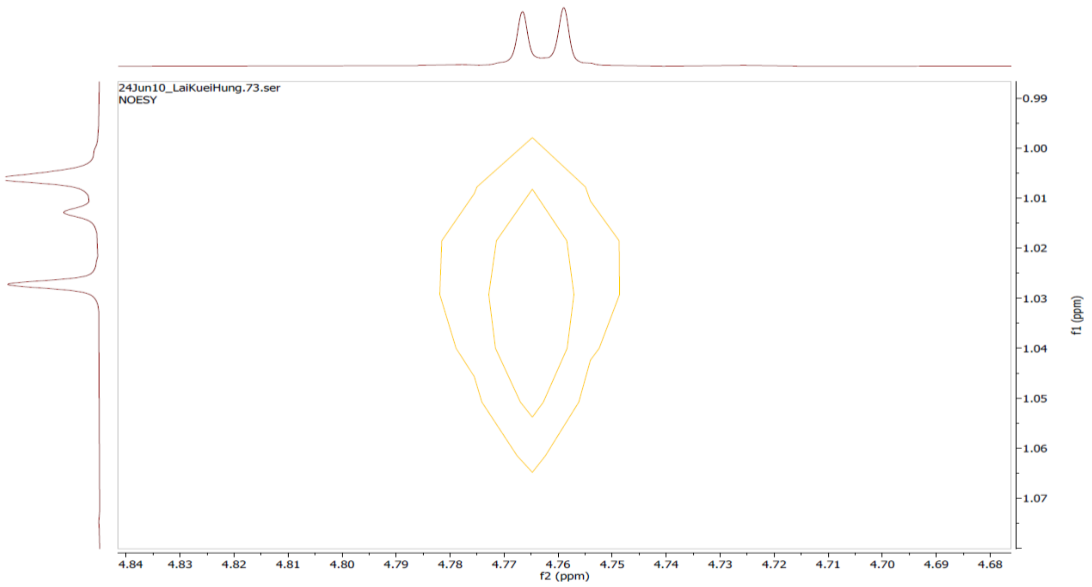

Figure S116. NOESY spectrum of 11-hydroxyzoanthamide B (3.38-3.48 ppm) at 800 MHz in $CDCl_3$

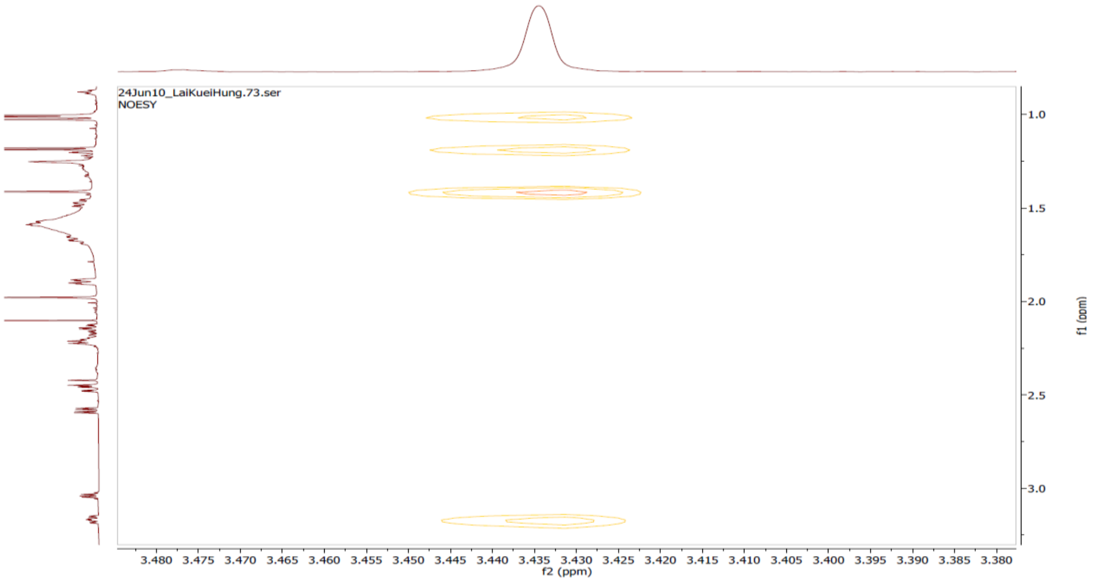

Figure S117. NOESY spectrum of 11-hydroxyzoanthamide B (2.54-2.63 ppm) at 800 MHz in $CDCl_3$

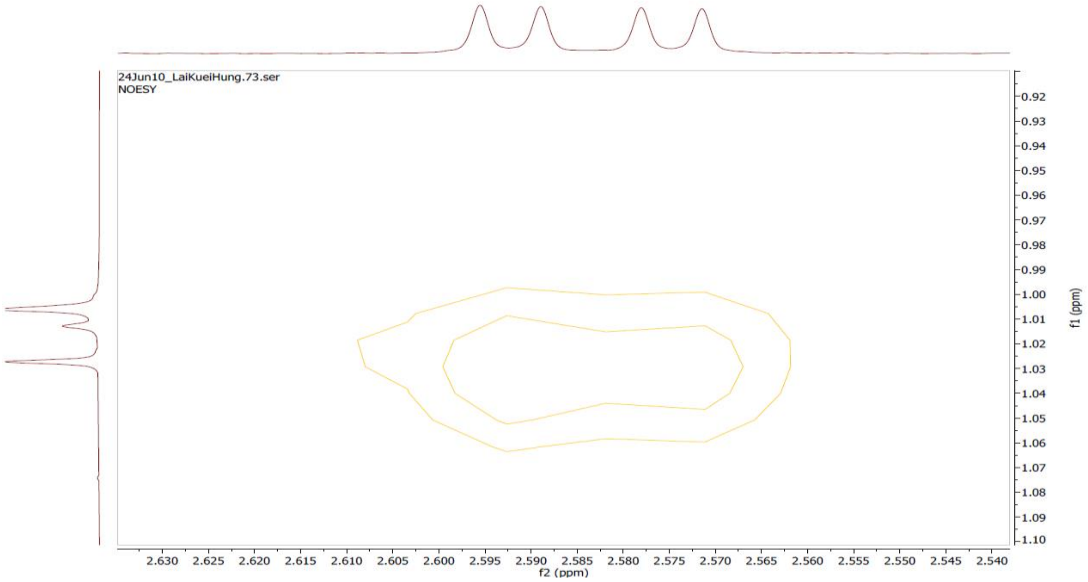

Figure S118. MS spectrum of 11-hydroxyzoanthamide B (**5**)

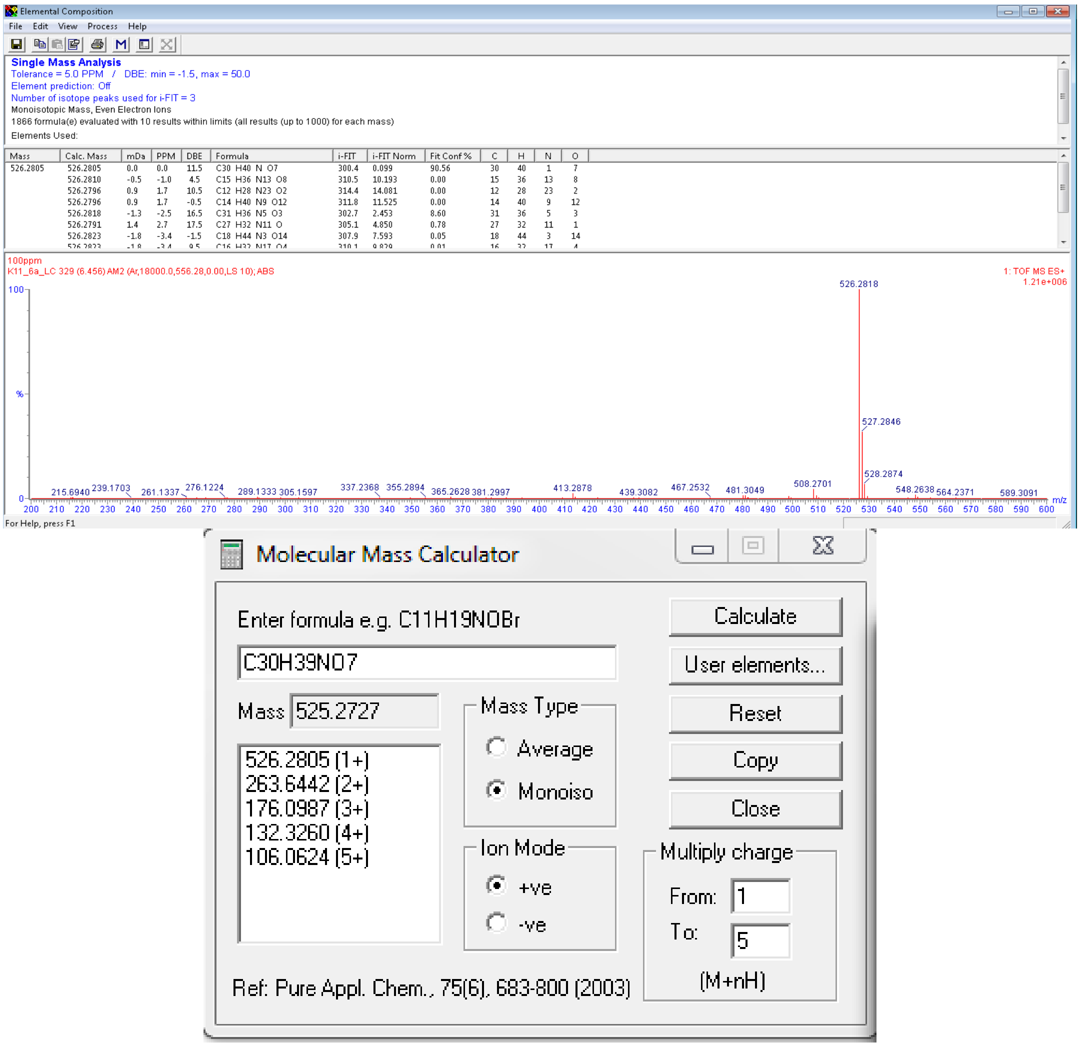

Figure S119. UV spectrum of 11-hydroxyzoanthamide B (**5**)

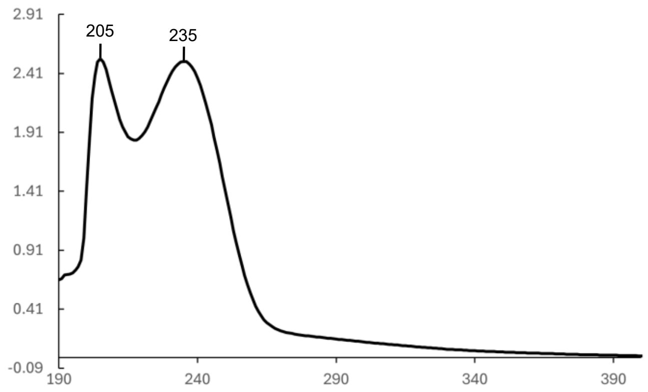

Figure S120. IR (ATR) spectrum of 11-hydroxyzoanthamide B (**5**)

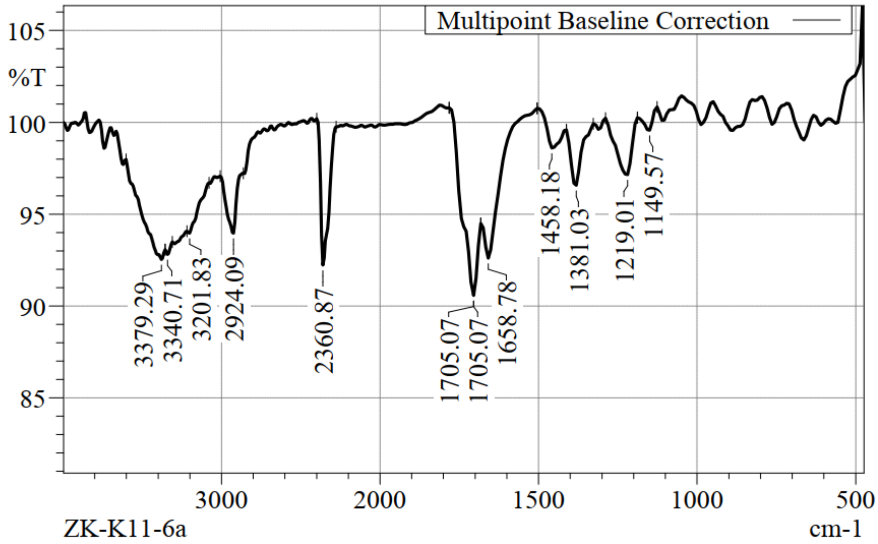

Figure S121. ^{1}H-NMR spectrum of norzoanthaminone (**6**) at 600 MHz in $CDCl_3$

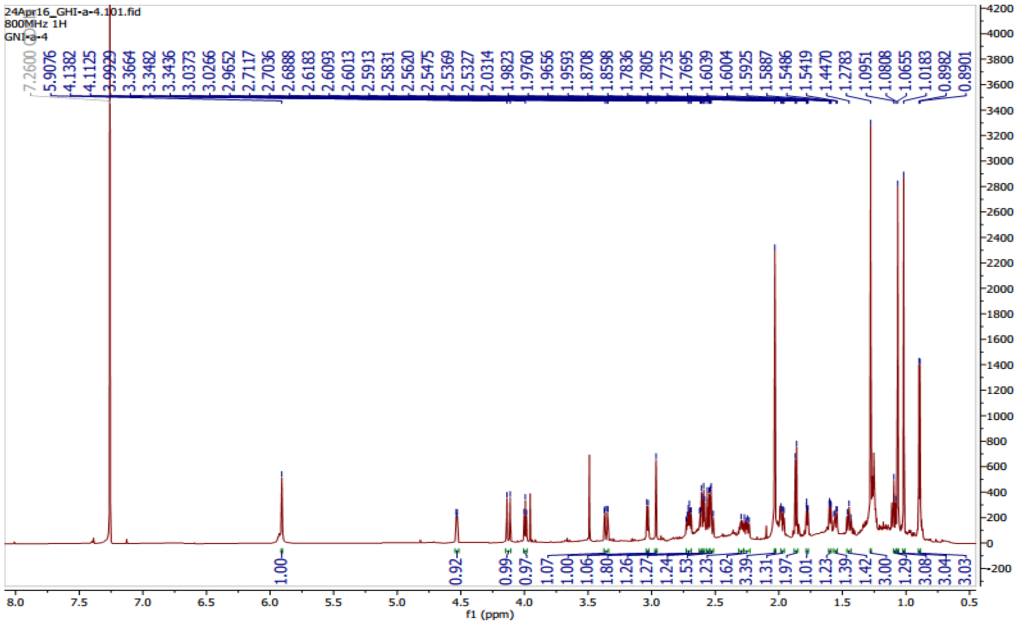

Figure S122. ^{13}C-NMR spectrum of norzoanthaminone (**6**) at 150 MHz in $CDCl_3$

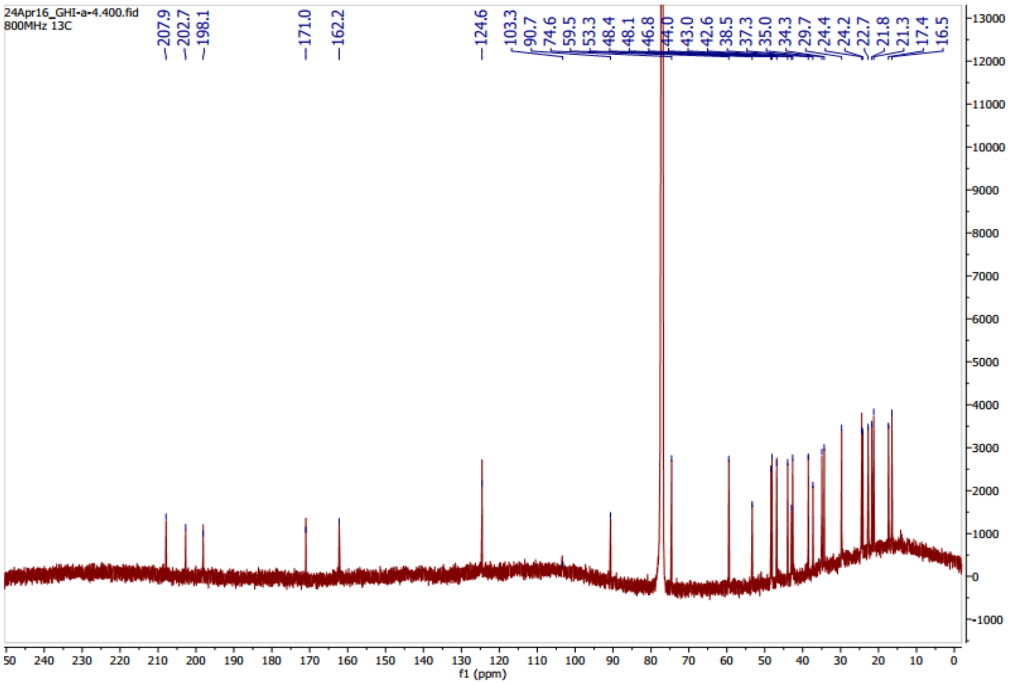

Figure S123. ^{1}H-NMR spectrum of 3-acetoxynorzoanthamine (**7**) at 600 MHz in $CDCl_3$

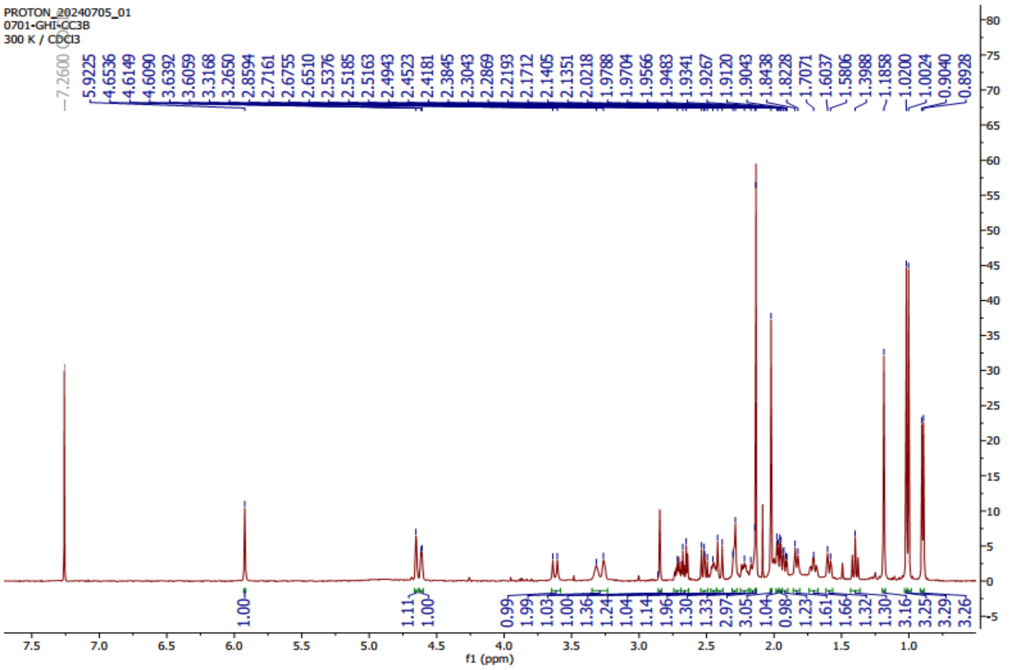

Figure S124. ^{13}C-NMR spectrum of 3-acetoxynorzoanthamine (**7**) at 150 MHz in $CDCl_3$

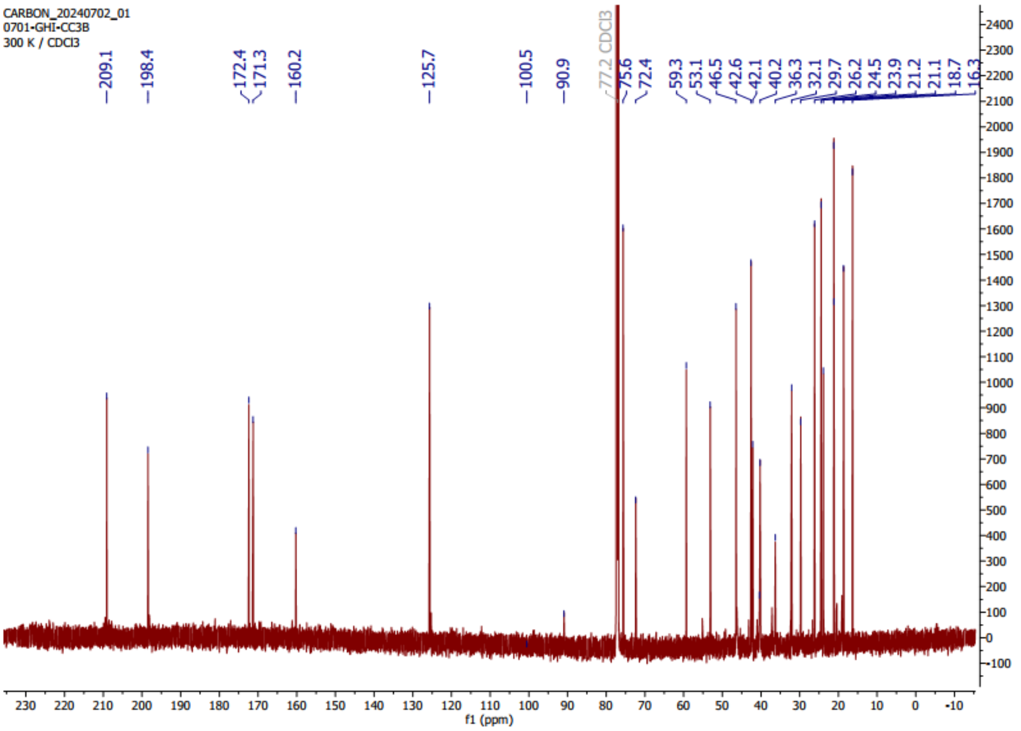

Figure S125. ^{1}H-NMR spectrum of 3-acetoxyzoanthamine (**8**) at 600 MHz in $CDCl_3$

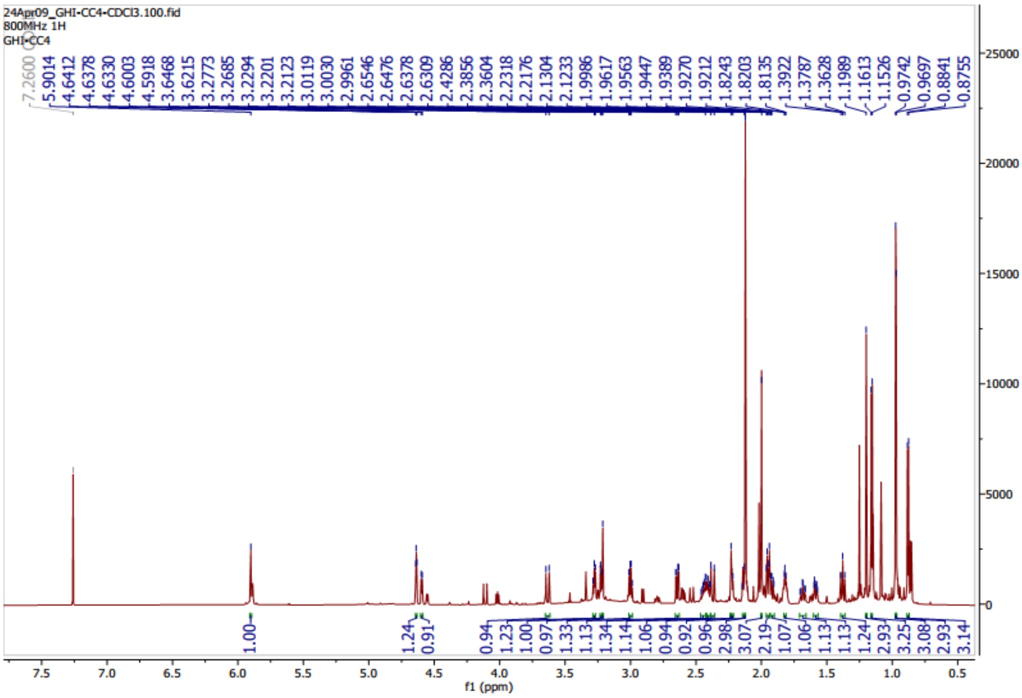

Figure S126. ^{13}C-NMR spectrum of 3-acetoxyzoanthamine (**8**) at 150 MHz in $CDCl_3$

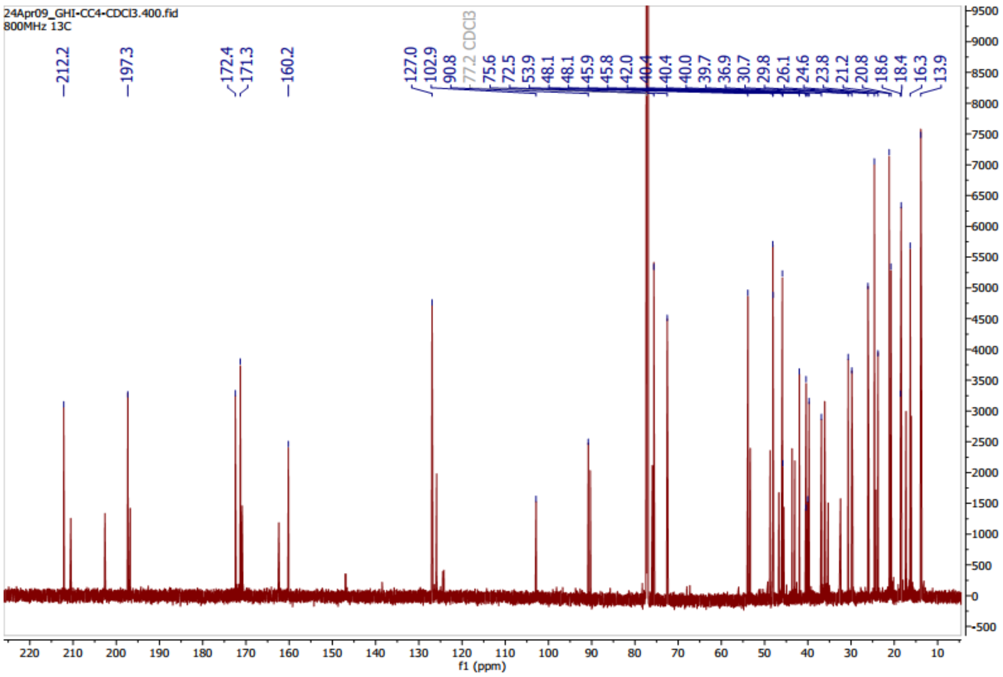

Figure S127. ^{1}H-NMR spectrum of 3-hydroxynorzoanthamine (**9**) at 600 MHz in $CDCl_3$

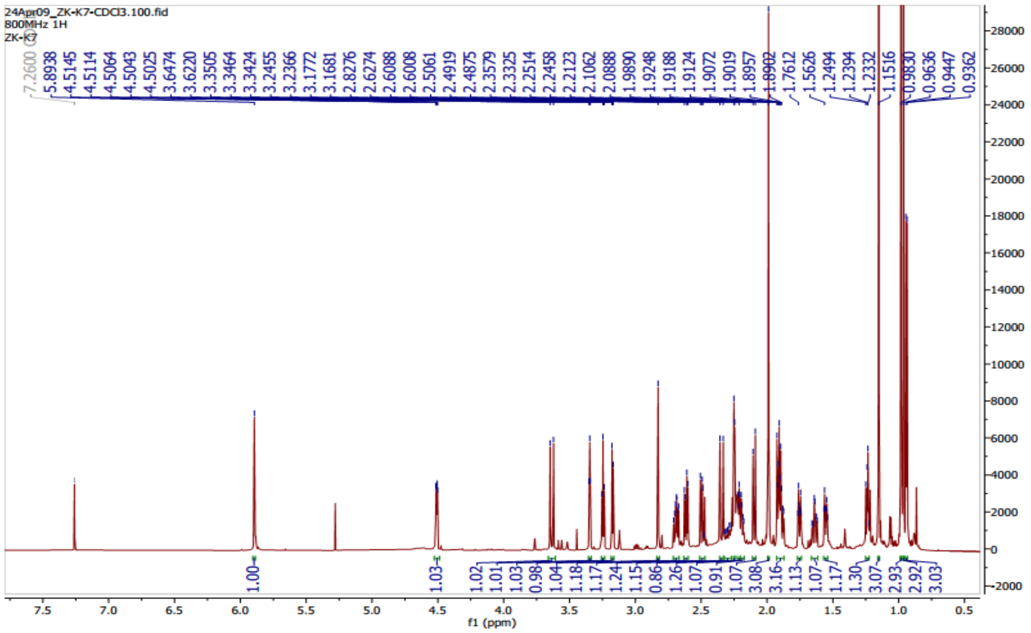

Figure S128. ^{13}C-NMR spectrum of 3-hydroxynorzoanthamine (**9**) at 150 MHz in $CDCl_3$

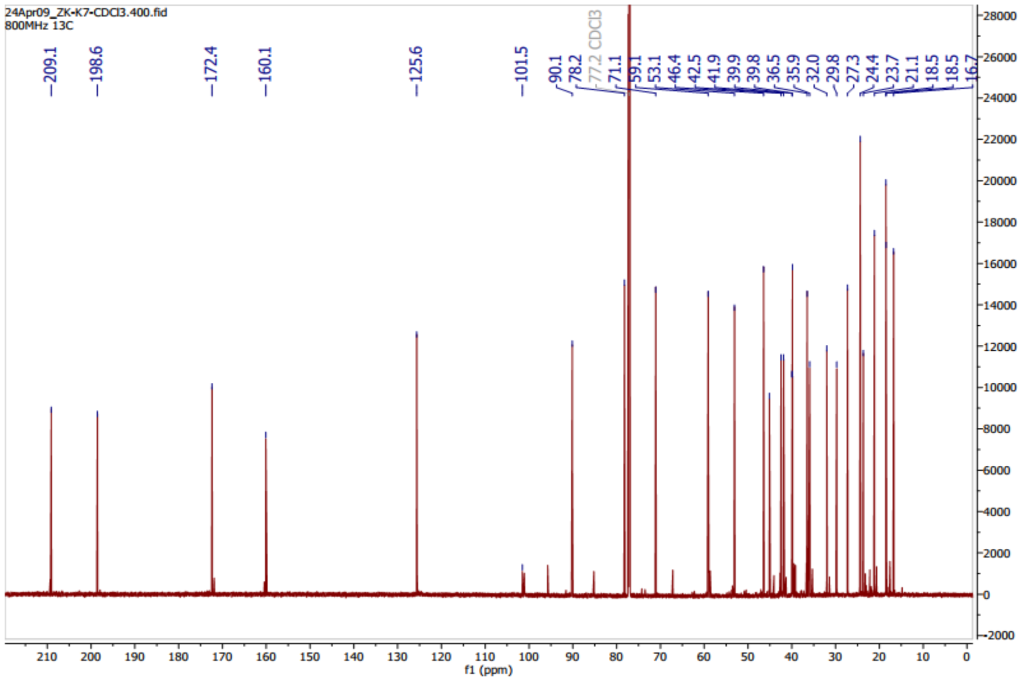

1. Cen-Pacheco, F., et al., *New oxidized zoanthamines from a Canary Islands Zoanthus sp.* Mar Drugs, 2014. **12**(10): p. 5188-96.
2. Guillen, P.O., et al., *Zoanthamine Alkaloids from the Zoantharian Zoanthus cf. pulchellus and Their Effects in Neuroinflammation.* Marine Drugs, 2018. **16**(7): p. 242.
3. Hsu, Y.M., et al., *Zoanthamine-Type Alkaloids from the Zoanthid Zoanthus kuroshio Collected in Taiwan and Their Effects on Inflammation.* J Nat Prod, 2016. **79**(10): p. 2674-2680.
4. Takahashi, Y., et al., *Total synthesis of zoanthenol.* Angewandte Chemie, 2009. **48 47**: p. 8905-8.
5. Costa-Lotufo, L.V., et al., *Chemical profiling of two congeneric sea mat corals along the Brazilian coast: adaptive and functional patterns.* Chemical Communications, 2018. **54**(16): p. 1952-1955.

Figure S129. ^{1}H-NMR spectrum of norzoabenzaldehyde (**1**) at 600 MHz in C_5D_5N after repurification

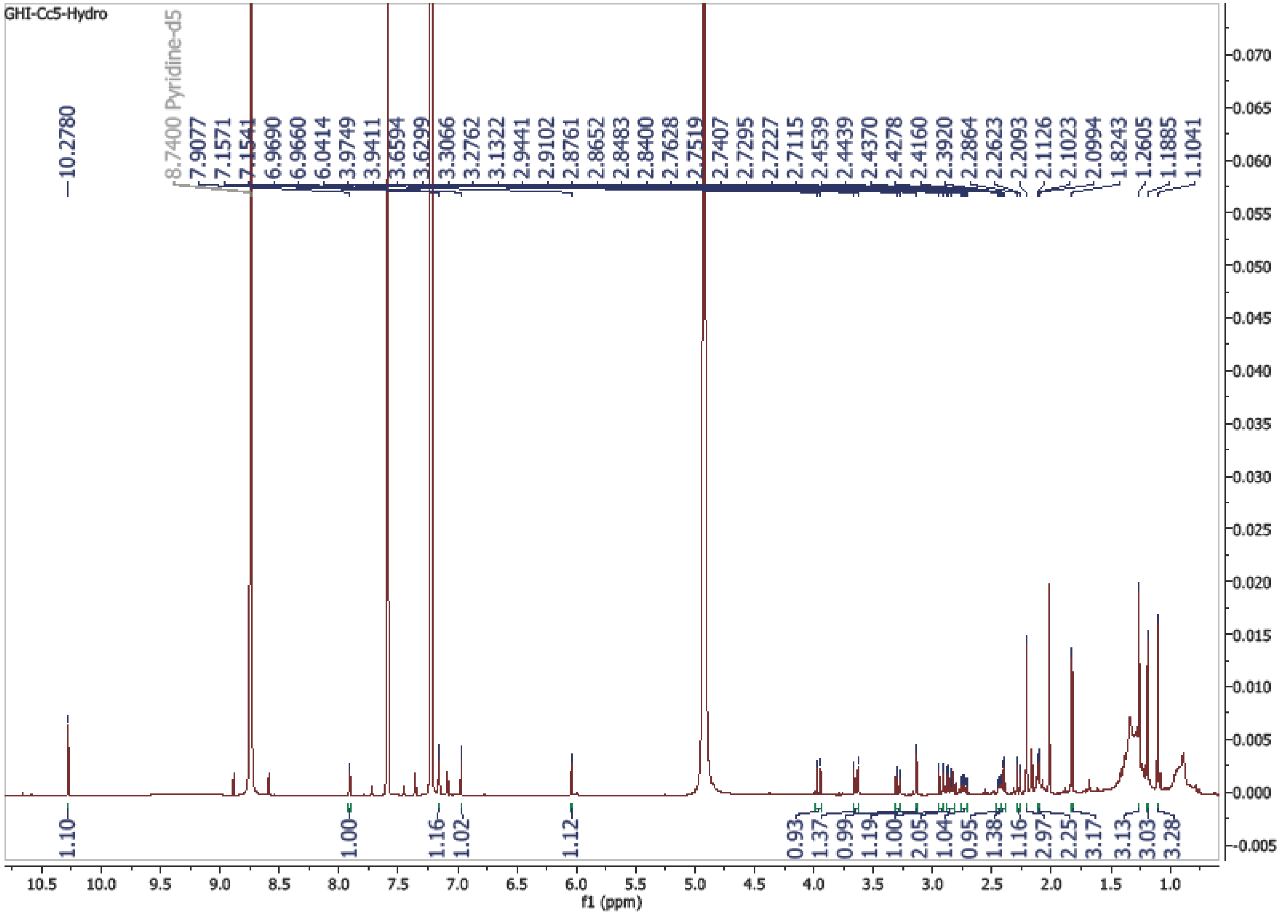

Figure S130. ^{13}C-NMR spectrum of norzoabenzaldehyde (**1**) at 150 MHz in C_5D_5N after repurification

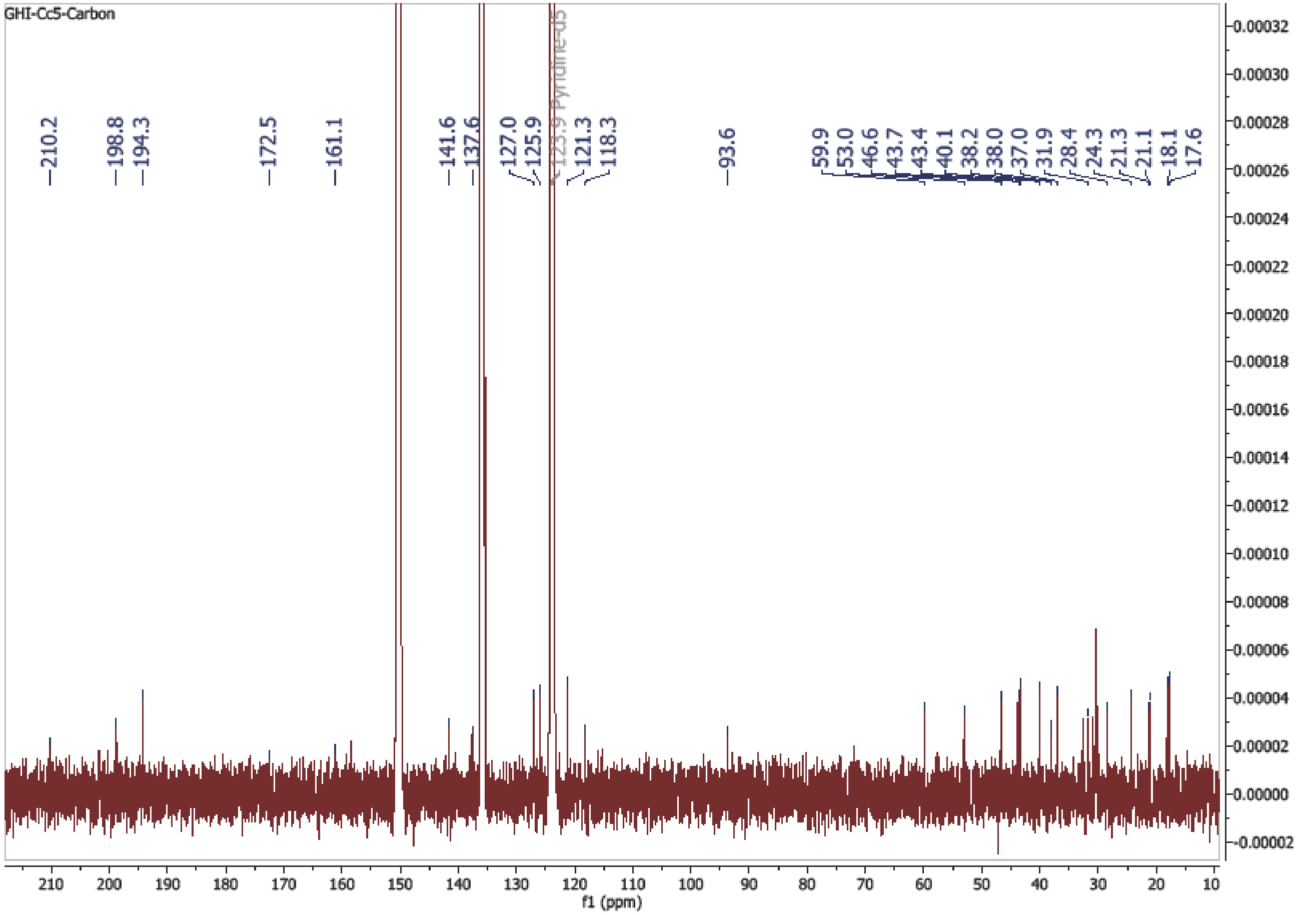

Supplement: Supplementary file 1 [file np5c00457_si_001.pdf]
